# An Integrated Biorefinery Proof of Concept: The Synthesis of Fully Bio-Based, Functional Lignin Polyester Copolymers of Cyclic Anhydrides and Epoxides Towards Polyol Applications and Tunable Bio-Derived Materials

**DOI:** 10.3390/polym17202806

**Published:** 2025-10-21

**Authors:** Oliver J. Driscoll, Daniel J. van de Pas, Kirk M. Torr, Hayden P. Thomas, Richard Vendamme, Elias Feghali

**Affiliations:** 1 Sustainable Polymer Technologies Team, Flemish Institute for Technological Research (Vito N.V.), Boeretang 200, 2400 Mol, Belgium; richard.vendamme@vito.be; 2 Scion Group, Bioeconomy Science Institute, Titokorangi Drive, Private Bag 3020, Rotorua 3046, New Zealand; daniel.vandepas@scionresearch.com (D.J.v.d.P.); hayden.thomas@scionresearch.com (H.P.T.); 3 Chemical Engineering Program, Notre Dame University–Louaize, Zouk Mosbeh 1211, Lebanon

**Keywords:** lignin, depolymerized lignin, cyclic anhydride, epoxide, polyester, polyol, bioplastic, polyurethane, ROCOP, copolymerization, polymer, biorefinery, ROCOP valorization, lignin valorization

## Abstract

A versatile, sustainable feedstock pathway to bio-based polymeric materials was developed utilizing lignin biomass and the ring-opening copolymerization (ROCOP) of cyclic anhydrides and epoxides to synthesize functional, lignin-derived, fully bio-based polyester polyols. The initial goal was to make the ROCOP reaction more applicable to bio-derived starting materials and more attractive to commercialization by conducting the polymerization under less constrained and industrially relevant conditions in air and without the extensive purification of reagents, catalysts, or solvents, typically used in the literature. A refined ROCOP system was applied as a powerful tool in lignin valorization by successfully synthesizing the lignin-derived copolyester prepolymers from lignin models and depolymerized native lignin sourced from the reductive catalytic fractionation of *Pinus radiata* wood biomass. After mechanistic studies based on NMR characterization, an alternative ROCOP-style mechanism was proposed. This was found to be (1) contributing to the acceleration of the observed reaction rates with added [PPNCl] organo-catalyst and (2) ‘self-initiation/self-promoted’ ROCOP without any added external [PPNCl] catalyst, likely due to the presence of inherent [OH] groups/ species in the lignin-derived glycidyl ether monomer promoting reactivity. As a final goal, the potential of these lignin-derived polyesters as intermediate polyols was demonstrated by applying them in the synthesis of polyurethane (PU) film materials with a high biomass content of 75–79%. A dramatic range of thermomechanical properties was observed for the resulting materials, demonstrating how the ROCOP reaction can be used to tailor the properties of the functional polyester and PU material based on the nature of the epoxide and anhydride substrates used. These findings help endeavors towards predicting the relationship between chemical structure and material thermomechanical properties and performance, relevant for industrial applications. Overall, this study demonstrated the proof of concept that PU materials can be prepared from lignocellulosic biomass utilizing industrially feasible ROCOP of bio-derived cyclic anhydrides and epoxides.

## 1. Introduction

With the plastics economy’s overreliance on diminishing petrochemical derivatives, ca. 98% in 2017 (352.8 million metric tons), the ecological damage caused at both ends of the linear plastic lifecycle, and the ensuing environmental concern, there has been attention on the use of biomass derived resources and the utilization of waste streams to produce alternative, sustainable biomaterials [1,2,3,4,5,6]. However, in the polymer sector, this currently only accounts for 2% of the global production being obtained from renewable sources (ca. 7.2 million metric tons in 2017), and therefore, a drastic, responsive shift is needed [1,3,4]. With the application of a circular, bio-economic approach, the aim is that these sustainable alternatives will help alleviate the environmental strain and replace fossil-based polymers and plastics.

Polyesters are ideal candidates for future commercial biomaterials due to their general biocompatibility, benign and facile end-of-life (EOL) hydrolytic degradation, and potential sourcing from renewable feedstocks [7,8,9,10,11,12]. The traditional, industrial method for their synthesis is via the step-growth, self-condensation polymerization of diols and diacids. However, there are many limitations with this method, including the requirement of precise monomer stoichiometry to achieve high-molecular-weight polyesters (>10,000 g·mol^−1^); relatively demanding conditions such as high temperatures, reduced pressures, and neat conditions; and the complete removal of produced water to access useful, high-molecular-weight polymer [4,12,13,14]. The other critical disadvantage is the lack of control in the overall reaction and the broad molecular weight distributions generally obtained [12,13]. An industrially preferred route to aliphatic polyesters has been the ring-opening polymerization (ROP) of cyclic esters, such as lactide and *ε*–caprolactone, because of the superior control in the chain-growth polymerization. However, these aliphatic polyesters are non-aromatic and less rigid. As a result, while high melting temperatures can be reached, unsuitable and inferior thermal properties can be displayed, such as low glass transition temperatures (*T*_g_), which limits their use in certain applications and suitability in replacing fossil-based polymers [13,15,16,17,18,19,20,21,22,23,24,25,26]. In addition, there is a lack of available cyclic monomers that can be polymerized [12,14]. Another alternative, promising, and versatile method is the ring-opening copolymerization (ROCOP) of cyclic anhydride and epoxide reagents [13]. This process is attractive and a potentially powerful tool because it enables access to the extensive range of commercially available anhydrides and epoxides, with certain monomers for both reagents having the potential to be sourced renewably, and more rigid aromatic/semi-aromatic polymer backbones. This can result in more varied and superior thermomechanical properties [20]. Therefore, with the fine-tuning of the anhydride/epoxide combination sets, there is potential to control the properties of the polyester material for a desired polymeric application.

High success has been achieved with anhydride/epoxide ROCOP using a range of metal initiators based on elements such as aluminum(III), chromium(III), cobalt(III), magnesium(II), zinc(II), and iron(III) [13,14,18,27,28,29,30,31,32,33]. In the majority of cases, homogeneous binary or multifunctional catalytic systems have been employed, with the metal behaving as a Lewis acid catalyst and an anionic, nucleophilic, Lewis basic co-catalytic species, such as *bis*(triphenylphosphoranylidene)iminium chloride (PPNCl) or 4– (dimethylamino)pyridine (DMAP), or functional groups tethered on the metal, to cooperatively aid the ring opening of the substrates [12,13,14,18].

While the application of different catalytic systems has been heavily explored, this has mainly been using a limited substrate scope on petrochemically derived monomers [33]. There has been strong emphasis in this area of late to explore substrates that have the potential to be sourced renewably from biomass sources such as sugars, terpenes and plant oils [2,7,33,34,35,36,37,38,39]. Liu et al. synthesized eugenol glycidyl ether (EGE) via the reaction of epichlorohydrin with eugenol; both reagents are renewably produced from glycerol waste [40,41,42,43] and plant oils such as clove oil [44,45], respectively, and coupled the epoxide with a range of cyclic anhydrides with a cobalt(III)–chloride complex/[PPNCl] binary catalytic system [36]. A new class of alternating polyester was afforded and, in particular, succinic anhydride (SA), which can be produced from succinic diacid—sourced via sugar fermentation [46,47,48] or, more recently, reported via the catalytic carbonylation [49] of bio-sourced acrylic acid, observed high reactivity to form the fully bio-based, linear poly(SA–*alt*–EGE) polymer (*M*_n_ = 5600–8500 g·mol^−1^, Đ = 1.86–2.06) [36]. However, a major, understated drawback in the current state-of -the-art for anhydride/epoxide ROCOP is with the conditions of the polymerization reactions and the purity of the reagents employed. To achieve higher-molecular-weight polymers, it is standard protocol for this ROCOP area to employ heavily purified reagents, heavily distilled epoxides, recrystallized or sublimed cyclic anhydrides, and even recrystallized or sublimed catalyst and co-catalysts, and rigorously eliminate air and moisture by conducting the reactions under dry, inert atmospheres in attempts to remove adventitious contaminants [7,12,27,28,29,30,32,33,35,36,37,38,50,51,52,53,54,55,56,57,58]. This includes hydrolyzed diols (residing from the epoxides), diacids (residing from the cyclic anhydrides), and residual moisture, which all participate as co-initiators and in chain-transfer side reactions lowering the molecular weight of the final polymer product [30,51,52,59]. Reports have already discussed the effect of the purity of reagents [12,50,51,52].

Recently, in contrast to this, North and co-workers synthesized functionalized, bio-based, low-molecular-weight polyesters under more ‘standard laboratory’ conditions: reactions conducted without rigorous inert, anhydrous conditions, without the need of a glovebox or specialized equipment, and instead flushing with a nitrogen atmosphere before heating. Polymerization was achieved using chromium(III) or aluminium(III)–chloride complexes or dicyclohexylurea organic compound as the catalyst and [PPNCl] as the co-catalyst (*M*_n_ = 990–2230 g·mol^−1^, Đ = 1.01–1.64) [40,56]. Epichlorohydrin (ECH) was employed as the epoxide substrate and SA or itaconic anhydride (IA) as the cyclic anhydride substrate; IA can be produced from itaconic diacid biomass sourced via the fungal fermentation of sugar or the pyrolysis of citric triacid [40,60,61,62,63,64].

For the ROCOP area to appear more attractive for commercialization, the current highly purified and inert, academic reaction protocols are impractical when operating industrially and employing lower-purity bio-derived starting materials, specifically those derived from biomass. In such situations, we believe the value of ROCOP lies in the functionality and novel properties that can be introduced into the polyester through the control and tailoring of the ROCOP. While circumventing the purity and inert sensitivity will undoubtfully result in expected deceases in the molecular weight of the polymer, this brings the potential for the ROCOP polyester to be exploited as a low-molecular-weight prepolymer, where a secondary polymerization reaction can be applied to make a polymeric material, offering a second opportunity to tailor functionality/performance further. This rationale is explored in this paper using ROCOP polyesters as a bio-based polyols for the synthesis of PU films as a proof of concept. This opens up a new versatile, *sustainable feedstock pathway* to new bio-based polymeric materials that are not attainable via self-condensation and ROP reaction routes.

In addition to a circular, bio-economic approach, the biorefinery concept will be at the core of future sustainable approaches. However, one of the greatest challenges for the scientific community has been making the process economically viable, as well as the valorization of lignin [65,66,67]. To achieve this, efforts have been focused on converting this often regarded low-value, underutilized by-product, to more sustainable, higher value chemical applications and macromolecular materials (lignin-to-chemicals and lignin-to-materials) [1,65,66,67,68,69,70]. Reductive catalytic fractionation (RCF) of wood, such as via metal-catalyzed hydrogenolysis, is a ‘lignin-first’ biorefinery approach that has recently gained attention as an attractive method for depolymerizing native lignin to produce lignin hydrogenolysis oil (LHO) products that have been employed as polyols for a range of material applications [1,65,67,68,71,72,73,74]. As far as we are aware, lignin has never been applied as a monomer to ROCOP, nor has any lignin model compound that is prominently abundant in LHO. Recently, Rao et al. grafted ROCOP reactions onto enzymatic hydrolytic lignin under rigorously anhydrous conditions. However, the lignin moiety was utilized as a macro-initiator and not as a monomer and therefore not polymerized. Instead, a range of cyclic anhydride/epoxide copolymers were synthesized on a lignin core, and the lignin was thus present in a low wt% (~10%) [75].

**Figure 1:**
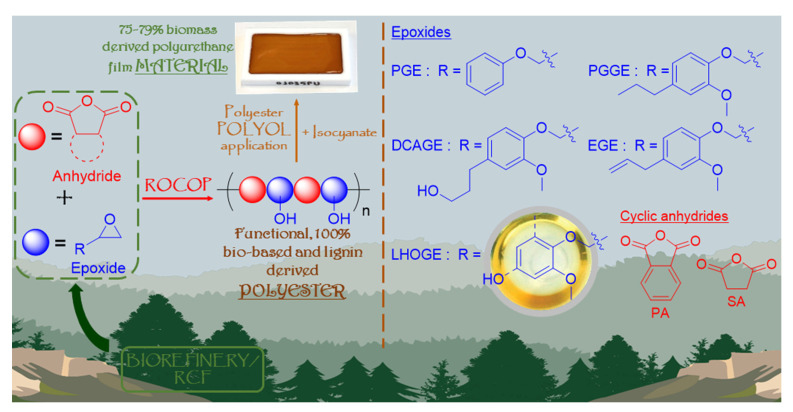
An overview of this proof-of-concept work: (1) the organo-catalytic ROCOP of lignin- derived epoxides, from both glycidylated lignin models and glycidylated depolymerized native lignin; lignin hydrogenolysis oil sourced from the reductive catalytic fractionation (RCF) of *Pinus radiata* wood biomass, and cellulosic/hemicellulosic derived cyclic anhydrides to synthesize functional, 100% bio-content polyesters. (2) The application of the DCAGE and LHOGE functional polyesters as polyols in the production of a range of polyurethane (PU) film materials with a high biomass content (75–79%).

As illustrated by Figure 1, in this proof-of-concept study, we successfully combined the contemporary, green chemistry themes of lignin valorization, anhydride/epoxide ROCOP, and organocatalysis to synthesize functional, lignin-derived, *fully bio-based* polyesters, from both glycidylated lignin models and glycidylated depolymerized native lignin; lignin hydrogenolysis oil (LHO) sourced from the RCF of *Pinus radiata* wood biomass. The polymerizations were conducted using more robust and industrially relevant conditions with no purification of any of the commercially received reagents, catalyst or solvent, and conducted in the majority of cases in air. Cyclic anhydrides can be derived from the furanics sourced from the cellulosic and hemicellulosic, plant biomass [40,46,48,61,76,77,78,79]. Hence, the biorefinery products from both the lignin and the polysaccharide components of biomass can be coupled together in one polymerization reaction via ROCOP. The value and potential of these lignin-derived, bio-based polyesters were demonstrated by exploiting their characteristic functionality and applying them as intermediate, lower-molecular-weight polyols in the synthesis of polyurethane materials where the thermomechanical performance can be tuned based on the chemical structure of the starting epoxides and anhydrides.

## 2. Results and Discussion

### 2.1. Development of a Catalytic ROCOP System for Lignin Model Epoxide Compounds and Phthalic Anhydride

Initial investigations were conducted using the chromium(III)salen–chloride complex, reported by Coates, bearing a 1,2–diaminocyclohexane (Jacobsen) ligand backbone, because of its reported robustness, high tolerance to both air and moisture sensitivity, and high activity for ROCOP (see ESI, Table S1) [56,80]. The complex demonstrates cooperativity and enhanced reactivity when employed in conjunction with a nucleophilic co-catalyst, such as PPNCl, and for this reason, a binary catalytic system was selected [30,40]. To probe if ROCOP were possible, the polymerization would be first proven using glycidylated lignin model compounds that resemble the structures found in glycidylated lignin hydrogenolysis oil. PGE was chosen as the epoxide substrate because it was the closest commercial substrate that held resemblance to a lignin moiety. Commercial PA was chosen as it was hypothesized that the aromaticity would enhance the anhydride’s tolerance and robustness to moisture, discouraging formation of the diacid and for ease of characterization [36,55]. In addition, it has been well reported that PA can be bio-based and produced from renewable biomass via furans, from the cellulosic fraction of lignocellulose, and Diels–Alder chemistry [19,76,77,78,79,81,82,83].

As part of our rationale and desire for more robust and industrially relevant reaction conditions, the polymerizations were conducted completely in air in standard 20 mL vials without the use of inert gases or a glovebox, and no purification of any of the commercially received reagents, catalysts, or solvents. In both solvent (standard-grade toluene solvent) and neat (solvent-free) polymerizations, the Cr(III)/PPNCl catalytic system demonstrated remarkable activity (TOF = 49–1744 h^−1^), as well as the control, at 60–110 °C, with varying monomer-to-catalyst ratios ([PA]:[PGE]:[Cr(III)]:[PPNCl] = [250–1000]:[250–1000]:[1]:[0–1]) for PA/PGE ROCOP (see ESI, Table S1). In addition, 4–propyl guaiacol (PG); a prominent monomer in depolymerized lignin hydrogenolysis oils [84,85] was glycidylated, and the resultant propyl guaiacol glycidyl ether (PGGE) successfully polymerized (see ESI, Table S2). However, despite extensive efforts, purification issues were encountered with the removal of the toxic [Cr] metal residue and its associated color from the product, despite only 1.6–6.3 mg of the catalyst being added for polymerization. This finding was unexpected as we employed the standard cleanup protocol in the literature, involving dissolving the product in dichloromethane and precipitating with acidified methanol [32,36,40,50,55]. Complete cleavage of the [Cr(III)] catalyst from the polymer chain was unsuccessful with the solid products isolated retaining a pale pink color (See ESI, Figure S1). It has been suggested in the literature that the purification procedure may not be optimal and catalysts based on metals other than chromium are preferred so colored metal residue do not remain in the polymer product [40,55]. In response to these findings, it was decided to attempt the ROCOP in the absence of the [Cr(III)] catalyst and, simply, with only the organic [PPNCl] as the catalyst, as part of a single-component catalytic system. This is because it was theorized the catalytic PPN^+^ and Cl^-^ ions would be easier to remove during later purification and afford polymer containing no metal residue [52]. It has already been reported in cases that [PPNCl] alone is able to catalyze ROCOP [12,20,30,40,50,52,58,59,86,87,88]. Furthermore, the application of metal-free, organic compounds as catalysts in the area of polymerization chemistry has recently attracted attention and is certainly in its infancy for ROCOP [12,40,50,75,89]. As well- reviewed by Le Bideau and co-workers, there are a number of advantages which make it potentially industrially relevant [12].

**Figure 2:**
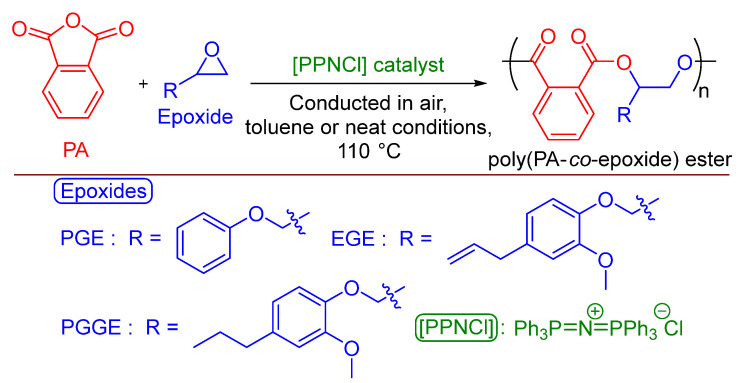
ROCOP of commercial PA and commercial PGE, synthesized EGE or PGGE using commercial [PPNCl] organo-catalyst in more robust, industrially relevant reaction conditions in air.

Organo-catalyzed PA/PGE ROCOP (Figure 2) was conducted employing, as before, more robust conditions in air and [PPNCl], with or without toluene solvent (see ESI, Table S3). For the neat polymerizations, at 110 °C and a [PA]:[PGE]:[PPNCl] ratio of [500]:[500]:[1], no reactivity was observed after 30 min, but a high conversion was afforded when increasing to 120 min, with a moderate molecular weight and narrow distribution obtained (*M_n_* = 4250 g·mol^−1^, Ð = 1.12). Unlike that for the [Cr(III)]/[PPNCl] catalytic system, all purified PA/PGE polymeric products isolated were non-colored and white (See ESI, Figure S2). The polymerization of synthesized PGGE (Figure 2) was successful under these neat reaction conditions with an extra 30 min needed to reach a moderate molecular weight (*M*_n_ = 3200 g·mol^−1^), possibly because of the extra steric hindrance from the methoxy group on the aromatic ring. Synthesized eugenol glycidyl ether (EGE) also reacted, giving a product of lower molecular weight (*M*_n_ = 1950 g·mol^−1^) after 2 h. The polymerization was controlled with the addition of half the amount of [PPNCl] catalyst ([PA]:[PGE]:[PPNCl] = [1000]:[1000]:[1]) requiring double the reaction time, but the molecular weight did not increase and remained constant at 4300 g·mol^−1^. However, decreasing the amount of [PPNCl] further ([PA]:[PGE]:[PPNCl] = [2000]:[2000]:[1]) did increase the molecular weight to 5000 g·mol^−1^, and the dispersity remained narrow (Ð = 1.19). As would be expected, under the same reaction conditions, using [PPNCl] as the sole catalyst was less active than the [Cr(III)]/[PPNCl] binary system, but reactivity was still observed under the robust conditions (for example: [Cr(III)]/[PPNCl], Table S1, entry 9, TOF = 1417 h^−1^ vs. [PPNCl] catalyst, Table S3, entry 3, TOF = 193 h^−1^).

Despite the lower activities, as solution polymerizations were observed to result in higher-molecular-weight polymer earlier with the [Cr(III)] system (see ESI, Table S1), standard-grade toluene (1.0 mL) was added to the polymerization here for PA/PGE ROCOP with [PPNCl] organo-catalyst (Table S3, entry 8, [PA]:[PGE]:[PPNCl] = [500]:[500]:[1]). The reaction time of 24 h was too long, but complete conversion was observed, and the molecular weight was unexpectedly lower than the respective neat polymerization (Table S3; entry 8, *M_n_* = 3950 g·mol^−1^ vs. entry 3, *M_n_* = 4250 g·mol^−1^). This may relate to the reaction time being too long and possible detrimental side reactions, such as chain-transfer with moisture [40] occurring at the high conversions and lowering the molecular weight of the polymer; the increasing dispersities observed may be indicative that this was potentially occurring (Table S3, entry 8, Ð = 1.29). In response to this, the solution polymerizations were repeated for 24 h with lower [PPNCl] catalyst amounts ([PA]:[PGE]:[PPNCl] = [1000]:[1000]:[1] and [2000]:[2000]:[1]), in an effort to further increase the molecular weight. Toluene addition was scaled relatively, to the amount of monomer, to ensure solubility of the crude mixture occurred because of the solvent, and the monomers were not merely melted, with the reactions partly in both neat and solution conditions. After 24 h reaction time, this was successful and both solution polymerizations afforded the higher molecular weights of 5250 g·mol^−1^ and 5700 g·mol^−1^ ([PA]:[PGE]:[PPNCl] = [1000]:[1000]:[1] and [2000]:[2000]:[1], respectively) compared to the respective neat polymerizations (Table S3, entries 9 and 10 vs. entries 6 and 7).

Using these findings, and because entry 10 of Table S3 afforded the highest molecular weight for PGE, it was decided to explore these optimized conditions further. It was observed that if the vial was flushed with argon and anhydrous toluene was employed (the ‘standard laboratory conditions’, employed by North), the molecular weight could be further increased to 6850 g·mol^−1^ (Table 1, entry 1) [40]. Our desire was to remain using conditions as robust and industrially relevant as possible, but as this was the highest *M*_n_ value observed and to expand the scope and to prove it was possible, these conditions were employed for the ROCOP of PA with synthesized PGGE, EGE and dihydroconiferyl alcohol glycidyl ether (DCAGE) (Figure 2); dihydroconiferyl alcohol (DCA) is another prominent monomer that can be present in high concentrations in depolymerized LHOs [71,90,91,92]. These glycidylated lignin models, except EGE, have never been trialed for ROCOP, and none have been trialed using [PPNCl] as the sole catalyst. Therefore, as a proof of concept, we wanted to demonstrate the versatility of the reaction and that reasonable-molecular-weight polymers could be obtained with all these three substrates; reasonable in terms of molecular weights sufficient and commonly desired for polyol applications (~2000–4000 g·mol^−1^) as was the final goal of this study.

High conversion was observed for PGGE and EGE after 24 h at this [epoxide]:[PA]:[PPNCl] ratio of [2000]:[2000]:[1] with low catalyst loading (Table 1, entries 2 and 3). Reasonable-molecular-weight polymer was observed for both (*M*_n_ = 3400 and 3600 g·mol^−1^ for PGGE and EGE substrate, respectively), while dispersity was narrow (Ð = 1.31–1.36). Interestingly, the DCAGE substrate demonstrated remarkable activity, and the ROCOP was considerably faster compared to the PGE, PGGE, and EGE substrates (Table 1). It was postulated at the time that the aliphatic OH side chains in DCAGE, not present in PGE, PGGE, and EGE, are acting as a co-catalyst, rather than behaving as spectator groups, and cooperating with the [PPNCl] in accelerating the polymerization. This was exemplified by the slight loss of control with the broader molecular weight distribution (Ð = 1.96). This was an important observation, as DCA monomers and oligomers are major components of the lignin hydrogenolysis oil (LHO) produced from pinewood using a Pd/C catalyst [90,91]. Despite the high activity, a polymer (not oligomer) was observed for this DCAGE polymerization (*M*_n_ = 2700 g·mol^−1^). The crude products of the organo- catalyzed ROCOP of PGGE, EGE, and DCAGE with PA were pale yellow or orange in color because of the inherent colors of the starting epoxides. This color was removed upon purification and thorough drying to yield near-white solid polymer products (see ESI, Figure S3).

### 2.2. Exploring Organo-Catalyzed PA/DCAGE ROCOP with Views to Further Material Applications

After demonstrating the ROCOP of glycidylated lignin model compounds, with phthalic anhydride, was feasible, our attention shifted to what potential future applications may the product polyesters behold. Recently, our research institutes demonstrated the synthesis of polyurethane (PU) foam and adhesive materials using LHO, from depolymerized native lignin sourced from *Pinus radiata*, as a polyol [72,93]. Therefore, with the inherent OH functionality of polyesters, we wanted to explore the possibility of using these product polyesters, derived from lignin models, as polyols for PU synthesis proof of concept. The OH content, measured using phosphitylation and ^31^P NMR spectroscopy (see ESI), was low for the polyesters using PGE, PGGE, and EGE substrates. For example, the poly(PA–*co*–PGE) from entry 2 of Table S3 observed a low total OH content of 0.45 mmol/g (aliphatic OH = 0.23 mmol/g and carboxylic acid OH = 0.22 mmol/g), which made it unsuitable as a polyol. On the other hand, the polyester produced from DCAGE was more promising with a higher OH content as a result of the presence of aliphatic OH arms. For example, a total OH content value of 3.06 mmol/g (aliphatic OH = 2.07 mmol/g and carboxylic acid OH = 0.99 mmol/g) was measured for Table 1, entry 4. Hence, this polyester product was functional and had the potential to behave as a polyol and couple with isocyanate for PU. In addition, because of the unexpected, remarkable activity for PA/DCAGE ROCOP, in comparison to the other lignin models, it was decided to further investigate the reaction conditions to improve the polyol properties of the polyester and better understand what was occurring in the polymerization.

The more robust solvent-free, neat reaction conditions in air, without the use of inert gases or a glovebox, were employed for PA/DCAGE ROCOP (Table 2) as was conducted earlier for PGE (Table S3). At a [PA]:[DCAGE]:[PPNCl] ratio of [500]:[500]:[1], high conversions were observed after only six and ten minutes; at ten minutes, the stirring had completely stopped due to the increase in viscosity of the crude mixture (Table 2, entries 1 and 2). These activities for DCAGE were vastly superior to those for the PGE substrate at the same conditions (DCAGE, 88% conversion after six minutes, Table 2, entry 1 vs. PGE, 77% conversion after two hours, Table S3, entry 3). Despite the high activity, reasonable molecular weights were observed (*M*_n_ = 2650–2800 g·mol^−1^) and, unexpectedly, these were higher than that for the solution polymerization conducted at a [PA]:[DCAGE]:[PPNCl] ratio of [2000]:[2000]:[1] (Table 1, entry 4). At the time, we thought this was potentially because the reaction time of eight hours for the solution polymerization was too long, and possible detrimental chain-transfer side reactions occurred.

Unpredictably, the molecular weight remained constant despite increasing the amount of [PPNCl] ([PA]:[DCAGE]:[PPNCl] = [250]:[250]:[1], *M*_n_ = 2650 g·mol^−1^ and [100]:[100]:[1], *M*_n_ = 2800 g·mol^−1^). This trend deviated when scaling up the reaction for entry 5 of Table 2, where the molecular weight noticeably decreased (*M*_n_ = 2050 g·mol^−1^) when employing higher quantities of the monomer substrates. There was a smaller decrease in the molecular weight when the reaction was scaled up even further (Table 2, entry 6). In terms of color, all the DCAGE-derived polymer products isolated, upon purification and drying, from Table 2, were visibly non-colored and near-white (Figure S4). There were initial concerns that some degree of cross-linking was occurring or the OH arms were reacting and directly inserting into the ringed monomer substrates. This would cause a loss in the control of the polymerization, thermosetting and may explain the slightly broader dispersities observed in this study (Ð = 1.51–1.79). However, all crude mixtures and polymers isolated were soluble for NMR spectroscopy and GPC analysis so remained thermoplastic. ^31^P NMR spectroscopy and GPC analysis was evaluated to aid this query. For example, focusing on entry 2 of Table 2, the total content was measured to be 3.13 mmol/g and the *M*_n_ observed to be 2800 g·mol^−1^ for the polymer. Thus, this determined a functionality of 8.8 for the poly(PA-*co*-DCAGE) {ƒ_OH_ = (3.13 mmol/g × 2800 g·mol^−1^)/1000}. This logically agrees with the degree of polymerization calculated to be 7.2 from GPC analysis (*M*_n_/*M*_R, PA and DCAGE_ = 2800 g·mol^−1^/388 g·mol^−1^ = 7.2) and hence an understandable functionality of ~1.5 for the mixed end groups (ƒ_OH, end groups_ ≈ 8.8 − 7.2 = 1.6). This suggests that a linear, thermoplastic, alternating, and functional polyester was formed with seven aliphatic OH side chains aligned along the polymer chain, giving rise to the high functionality (Figure S5); the functional polymer being thermoplastic currently but post-modification, using these aliphatic OH arms, would rapidly result in an insoluble thermosetting material. This demonstrated nicely the exceptional control and how powerful a tool ROCOP can offer, with the OH aliphatic arms not inserting into the polymer or cross-linking, and the driving force for the reaction being solely the ring opening of both the ringed reagents. It is doubtful theoretically that this control would be observed if the alternative self-condensation reaction of DCA and phthalic diacid were used, where both phenolic and aliphatic OH groups would likely react to form ester moieties and a thermoset polymer. This analysis, using GPC and ^31^P NMR spectroscopy, confirmed that the aliphatic OH arms were present and free to behave as catalytic species with the [PPNCl] for the PA/DCAGE ROCOP reactions. Therefore, unlike the polyesters synthesized using PGE, PGGE, and EGE substrates containing low OH contents, these aliphatic OH arms in the poly(PA–*co*–DCAGE) had the potential to perform as polyols for PU thermoset material synthesis via post-modification.

### 2.3. Blank Control ROCOP Reactions, Chain-Transfer Reactions, Self-Catalysis/Promoted ROCOP and [OH] Species

Unlike those observed for PA/PGE ROCOP, the results were unexpected that for PA/DCAGE ROCOP (Table 2): (1) the reaction was remarkably more active with the presence of OH side chain moieties cf. PA/PGE, PGGE, EGE ROCOP; (2) increasing the amount of [PPNCl] organic catalyst did not lower the molecular weight of the isolated polymer, and the observed *M*_n_ values could not be controlled by [PPNCl] concentration. Compared to the other PGE, PGGE, and EGE substrates (*conducted under the same reaction conditions*), it was earlier postulated that the remarkable activity observed using the DCAGE substrate was as a result of the aliphatic OH side chains, but defining these groups as a co-catalyst does not support these findings (Table 2), so more was at play than initially thought. Despite no control of the *M*_n_ values with [PPNCl] concentration, the polymerization remained relatively controlled with regards to the measured *T*_g_ increasing upon subtle increases in the molecular weight (*T*_g_ = 29.5–37.9 °C). However, the dispersities did broaden slightly (Ð = 1.51–1.79). Furthermore, upon increasing the scale of the reaction, the molecular weight decreased.

The blank PA/DCAGE control reactions, without added [PPNCl], revealed the aliphatic OH side chains were able to initiate the polymerization; possibly via H-bonding interactions following the classical ROCOP mechanism as an initial theory, albeit with slower activity and affording a lower-molecular-weight polymer (see ESI, Table S4, entries 1 and 2) [12,13,50,94]. The fact polymerization was able to initiate itself, with *structural functional groups inherent on one of the monomers* (not externally added chemicals such alcohols or diols), introduced the theme of ‘autocatalysis’. To our knowledge, the term ‘autocatalysis’ is strictly employed when the product contributes to the catalysis, but here it is more likely all [OH] containing species, particularly the reactant, not the less mobile polymer. As far as we are aware, we could not find an example of a reaction or polymerization in the ROCOP literature where a functional group on the reactant was mainly responsible for catalysis or reactivity. Therefore, we defined these observations as ‘self-catalyzed ROCOP’ or ‘self-promoted ROCOP’. Furthermore, the DCAGE could be applied catalytically when spiked into a PA/PGE ROCOP mixture without [PPNCl]. Impressively, reactivity was observed at concentrations as low as 0.1–0.2 mol% for the [DCAGE] or [OH side chains] (0.003–0.007 mmol OH/g) after 22–28 h (Table S6). At this point, the concentration was not lowered further because the blank PA/PGE ROCOP observed low conversion, likely through diol or diacid impurities after 24 h, and extending the time period could lead to false interpretations [51,52]. These were important findings considering the [OH] species [71,90,91] in lignin hydrogenolysis oil glycidyl ether (LHOGE) and would also therefore be predicted to promote initiation for ROCOP.

Depending on the definition of the [OH] catalytic species (mol% or wt%) or the catalytic group ([OH] group, [CH_2_CH_2_CH_2_OH], or [DCAGE]), in molar equivalents, there was actually a stoichiometric amount of the monomer’s inherent structural OH side chains in the PA/DCAGE ROCOP (Table 2). For example, 2.5 mmol [DCAGE] meant there was 2.5 mmol [OH side chains] in the polymerization. Therefore, it was proposed that the aliphatic OH side chains may have been behaving as a chain transfer agent (CTA) and not a catalytic species when in combination with the [PPNCl] catalyst. This may explain the accelerated rate and increased *M*_n_ values when still used in combination with [PPNCl], the broader dispersities, and the [PPNCl] not controlling the molecular weights [95,96,97]. There are examples of externally added CTAs in ROCOP, termed reversible-deactivation ring-opening copolymerization (RD-ROCOP), and these generally entail a sophisticated metal-based catalyst with the addition of a protic CTA such as alcohol, amine or carboxylic acid [14,29,97,98,99,100]. However, there are examples of organo-catalysts that can undergo immortal polymerization for other ring-opening polymerization reactions [96]. However, in our case, there is not an exogenous amount of alcoholic CTA added because it was structurally inherent at high concentrations in the lignin-derived epoxide monomer.

It was also found that, after conducting other blank ROCOP reactions under the same conditions (see ESI), the initiation from OH groups for DCAGE may not have been fully derived from the aliphatic OH side chains. Unexpectedly, slower polymerization was still observed without the presence of these moieties for synthesized PGE (cf. to commercial PGE, Table S5), PGGE, and EGE (Tables S4 and S5), and this was attributed to traces of [OH] containing impurities from the glycidylation protocol, such as the α–chlorohydrin species. These species would also be present in the synthesized DCAGE and thus may have had minor contributions to the accelerated reaction times with the high concentrations of OH side chains [71,95]. With molecular weight dependent on the total [OH], this may account for the decreasing *M*_n_ values upon increasing the scale of polymerization in Table 2. Despite this, in all cases for all synthesized lignin model epoxides, the ROCOP was accelerated with the addition of [PPNCl]. For more information, see the ESI.

### 2.4. Mechanistic Considerations: Why the Increase in Rate with the Presence of [OH] Groups, the Role of the [OH] Groups, and an Alternative ROCOP Mechanism Proposed Inherent for Lignin

**Figure 3:**
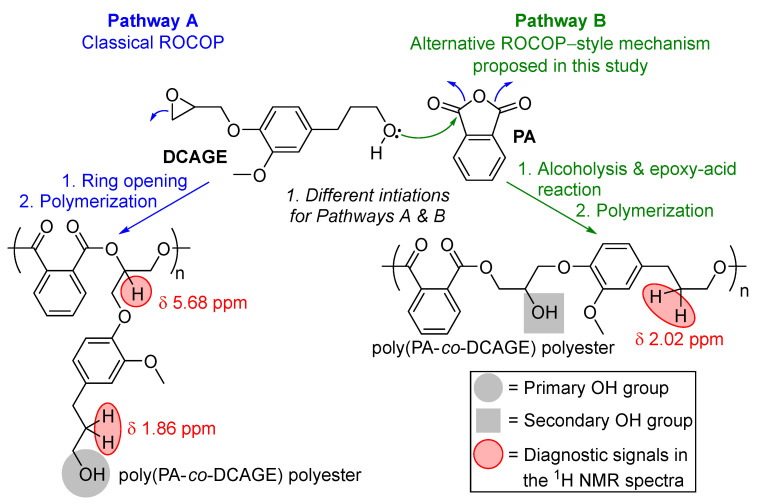
An illustration of the two proposed mechanistic reaction pathways in operation for the ROCOP of lignin-derived epoxides with cyclic anhydrides (PA shown here) in this study: Pathway (A) classical ROCOP, and Pathway (B) alcoholysis, epoxy–acid reactions and ROCOP (see ESI, Figures S11 and S12 for more details).

Efforts were made to further deduce the unexplained observations made and the specific role of the aliphatic [OH] side–chain groups, which likely caused the increase in reactivity for DCAGE because the situation was complex. For the PGE, PGGE, and EGE substrates, as these contained no [OH] moieties, these could only proceed via the classical ROCOP mechanism generally reported with PA [13,18]. For DCAGE, it was initially proposed earlier that the increase in rate could be as a result of the aliphatic OH side chains participating in H-bonding interactions via a classical ROCOP mechanism, labelled here as ‘*Pathway A*’ (Figure 3 and see ESI, Figures S11 and S12 for more details) [12,13,50,94]. However, the possibility was theoretically explored that a change of mechanism and a different ROCOP-derived mechanism was operating to explain the faster rate with [PPNCl] and ‘self-initiation/self-promoted ROCOP’ without the presence of [PPNCl]. After further in-depth evaluation of the ^31^P NMR spectra of phosphitylated poly(PA–*co*–DCAGE) products (Table 2), the majority of the aliphatic OH groups were, in fact, distinguished as secondary alcohols and not primary alcohol groups inherent with a classical ROCOP polyester product (Pathway A, Figure 3, and see ESI). Therefore, to explain this, an alternative ROCOP-style mechanism was proposed considering the aliphatic OH group on the DCAGE reacting with the cyclic anhydride, PA, and ring opening. This was termed as ‘*Pathway B*’ (Figure 3 and see ESI) and involved (1) an alcoholysis reaction of the cyclic anhydride, which has been previously suggested in the literature for lignin [75,101,102]; (2) the carboxylic acid reacting with the epoxide of another molecule via an epoxy–acid reaction [103]; (3) these two steps repeating and ROCOP to obtain a polyester with a secondary aliphatic OH functionality. Thus, the OH groups would still be initiating the polymerization, but instead of hydrogen-bonding, they are ring opening the two co-monomers. Therefore, there were two proposed routes to polyester products for DCAGE (Figure 3). Both routes are able to produce linear, thermoplastic, functional polyester for future polyol applications, with aliphatic OH groups running along the polymer backbone and indistinguishable via total OH content and GPC analysis. In addition, tertiary amines have been shown to catalyze rapid alcoholysis reactions, and the addition of [PPNCl] could accelerate both the rate of reaction for Pathway B as well as Pathway A [101]. However, as well as the polymerization following either route, the situation was complex with the possibility of both pathways being in operation (a mixed mechanism) and two different polyester products formed.

Further NMR spectroscopic studies, using a combination of techniques (such as ^1^H, ^13^C, DEPT135, HSQC, and HMBC spectroscopy), were employed on the polyester samples, and selective Total Correlation Spectroscopy (TOCSY) NMR was performed to aid NMR signal resolution and distinguish overlapping signals (see ESI for details). This revealed: (1) The confirmation that PA/PGGE ROCOP followed Pathway A solely because of the absence of OH groups. (2) The presence of diagnostic signals in the ^1^H spectra for Pathway A and Pathway B for poly(PA–*co*–DCAGE) (Figure 3) was in agreement with the predictive NMR calculations. (3) Both mechanisms were in operation for PA/DCAGE ROCOP. For example, using the distinct, diagnostic signals in the ^1^H NMR spectra (Figure 3), it was possible to quantify the different types of copolymer units for the poly(PA–*co*–DCAGE) products {[Pathway A]:[Pathway B] for Table 2, entry 4 = 1:4, Table 2, entry 6 = 1:2.5 and Table S4, entry 2 = 1:3 (without [PPNCl]}. It is common to apply MALDI–ToF analysis for ROCOP-derived polyesters in the literature [50,55]; however, this was ***not*** possible in this study employing a high content of lower purity, lignin-derived glycidyl ethers and polyesters. ESI–MS was attempted, but the analysis was complicated, and efforts are still ongoing. Other evidence for the existence of Pathway B include the carboxylic acid OH values determined for the poly(PA–*co*–DCAGE) polyesters (see ESI), supporting the attack and opening of the anhydride by the aliphatic OH group of DCAGE [102]. In addition, spiking a PA/commercial PGE ROCOP reaction with an external, stochiometric amount (100 mol%) of commercial 3–phenyl–1–propanol mimicked the structural moiety of the DCAGE side chain (without the glycidyl ether functional group), and no [PPNCl] observed full conversion but did not induce polymerization with GPC analysis confirming no build-up of molecular weight supporting Pathway B (Figure 3, step 1) (Table S7). Furthermore, as was mentioned earlier, when DCAGE could be applied catalytically when spiked into a PA/PGE ROCOP mixture without [PPNCl], it was observed that molecular weight increased as the [DCAGE] decreased in the ROCOP mixture, also implying support of Pathway B (Table S6). Overall, all these findings with the lignin model compounds were important to consider before the application of lignin hydrogenolysis oil glycidyl ether (LHOGE) to ROCOP (and, with a view to later proof-of-concept material synthesis); with DCA, PG, and aliphatic OH side chains being prominent in LHO, the traces of [OH] contain impurities from the glycidylation protocol in LHOGE, and the LHO biomass contains a mixture of many [OH], containing monomer, dimer, and oligomer species [71,104].

### 2.5. Organo-Catalyzed ROCOP with PA and LHOGE Sourced from the RCF of Pinus Radiata Biomass and Glycidylation

**Figure 4:**
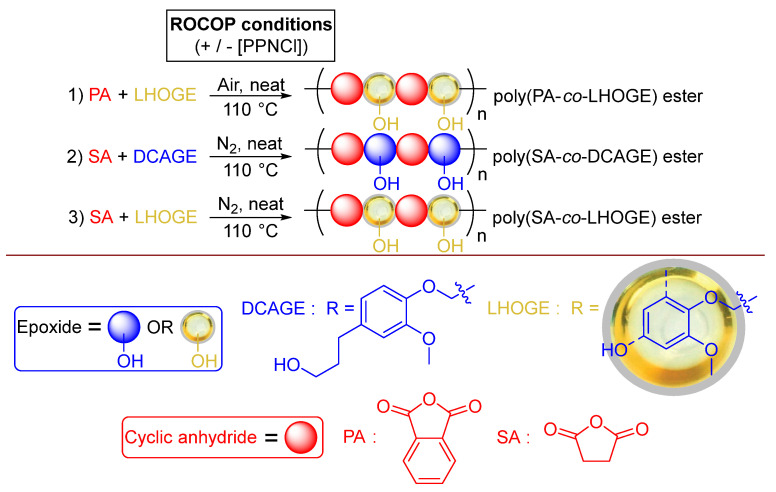
The organo-catalyzed ROCOP of synthesized LHOGE; sourced from the reductive catalytic fractionation (RCF) of native *Pinus radiata* wood, in the laboratory at pilot scale, and commercial PA or SA with/without commercial [PPNCl] under solvent-free, neat, more robust, and industrially relevant reaction conditions.

Having proven that ROCOP could be successfully applied to the lignin model compounds under robust conditions in air, the focus shifted to the application of actual lignin from biomass using LHOGE (Figure 4 and Table S8). This was sourced from the native lignin in *Pinus radiata* wood via RCF using a Pd/C catalyst to produce LHO in the laboratory at the pilot scale, followed by glycidylation as previously reported [71,93]. The LHO, denoted LHO4 in the previous study [71], was composed of a mixture of monomers, dimers and oligomers of predominantly DCA and PG. The monomers DCA and PG made up 16.1 wt.% and 4.7 wt.% of the LHO, respectively [71]. Two PA/LHOGE ROCOP polymerizations (Figure 4) were initially conducted with and without external [PPNCl] organic catalyst added to the mixtures (Table S8, entries 1 and 2).

This was a check to detect if there was possible activity from the species containing unglycidylated OH groups in the LHOGE mixture, the previously discussed ‘self- catalyzed ROCOP’ and Pathway A/B discussion (Figure 3), and to probe the effect of the added [PPNCl] on the polymerization. After 15 min, there was polymerization observed without the need for added [PPNCl] (38% PA conversion, *M*_n_ = 2000 g·mol^−1^). However, as was the case with the lignin models, the activity was enhanced with the presence [PPNCl] and resulted in an increased molecular weight (63% PA conversion, *M*_n_ = 2600 g·mol^−1^). Both the conversion and molecular weight were further increased when extending the reaction time to 20 min with [PPNCl] (Table S8, entry 3, 83% PA conversion, *M*_n_ = 2800 g·mol^−1^). Similar to that for DCAGE (Table 2), there was a minimal degree of molecular weight control of the polymerization via variation of the amount of [PPNCl] organo-catalyst added for LHOGE (*M*_n_ = 2750–2950 g·mol^−1^). However, reactivity was influenced by the [PPNCl] concentration with an increased [PPNCl] loading (from [PA]:[LHOGE]:[PPNCl] = [250]:[500]:[1] to [100]:[100]:[1]), increasing the reactivity (78% PA conversion after 15 min). While a decrease in the [PPNCl] loading (from [PA]:[LHOGE]:[PPNCl] = [250]:[250]:[1] to [500]:[500]:[1]), decreased the reactivity (79% PA conversion after 30 min). Increasing the scale of the polymerization from 1.25 mmol to 6.81 mmol epoxide content in the [LHOGE] decreased the molecular weight (Table S8; entry 6, *M*_n_ = 2450 g·mol^−1^ vs. entry 4, *M*_n_ = 2750 g·mol^−1^), as would be expected from that observed using the DCAGE substrate (Table 2). Both the OH content and *T*_g_ values observed from the isolated poly(PA–co–LHOGE) products displayed minimal variation at around 3 mmol total OH/g and 57–61 °C, respectively (Table S8, and for a full breakdown of the OH contents for all DCAGE/LHOGE polyesters, see Table S11). Due to the broad nature of the ^1^H NMR signals in the spectra for the poly(PA–*co*–LHOGE) products, it was not possible to determine the ratio of polyester structures from Pathway A vs. B as was tried for poly(PA–*co*–DCAGE); however, the ^31^P NMR spectra again implied Pathway B was the preferred route. Fundamentally, this proof of concept demonstrated that functional lignin-derived polyester could be synthesized from glycidylated depolymerized lignin; lignin hydrogenolysis oil (LHO) sourced from the RCF of native lignin in *Pinus radiata* wood in air (Figure 4). Furthermore, as cyclic anhydrides can be derived from cellulosic derived furanics and bio-based epichlorohydrin (employed for the glycidylation) already produced industrially [40,41,42,43], these lignin-derived polyesters can therefore be fully bio-based (100% bio-content).

### 2.6. Organo-Catalyzed ROCOP of DCAGE and LHOGE with Succinic Anhydride

To expand the scope of anhydride substrates, efforts were focused on succinic anhydride (SA) as an aliphatic cyclic anhydride that can be sourced from biomass that is structurally different from the rigid, aromatic PA. It was envisaged that this would produce polyester and PU materials with contrary thermomechanical properties and therefore was considered interesting to study. The ROCOP of SA with DCAGE (Figure 4 and Table S9, see ESI) was conducted under the same conditions used for PA with unpurified commercial reagents and [PPNCl]. However, with SA being aliphatic rather than aromatic, it was expected that the SA would be less stable than the PA under heating. As a precaution to reduce the likelihood of potential undesired side-reactions, the reaction vial was flushed briefly with nitrogen before polymerization; *akin* to North et al. [40] At two different loadings of [PPNCl] (Table S9, entries 1 and 2), after only 15 min at 110 °C, the resonance signal at δ 3.00 ppm in the ^1^H NMR spectrum for SA reagent had disappeared for the crude polymerization mixtures (see ESI), indicating complete reactivity. After purification, as would be expected, the lower organo-catalyst loading afforded the slightly higher-molecular-weight polymer ([SA]:[epoxide]: [PPNCl] = [500]:[500]: [1], *M_n_* = 2450 g·mol^−1^ vs. ([SA]:[epoxide]: [PPNCl] = [100]:[100]:[1], *M_n_* = 2350 g·mol^−1^). Both polymerizations observed the same dispersity (Đ = 1.75–1.78). FT–IR analysis indeed confirmed the polymer was poly(SA–*co*–DCAGE) polyester and, as a result of the similar *M*_n_ values, the total OH content was determined to be identical for both products (2.56–2.57 mmol total OH/g). The undisturbed aliphatic OH side-chains (2.18 mmol aliphatic OH/g), along with expected carboxylic acid OH groups (0.38–0.39 mmol carboxylic acid OH/g), were observed following the polymerization. When the scale of the polymerization was increased (Table S9, entry 3) to produce more thermoplastic polyester product for PU material synthesis, the molecular weight was reduced (*M*_n_ = 1100 g·mol^−1^). As a result, the measured OH content was increased to 3.11 mmol total OH/g with a reduction in the chemical functionality (ƒ = 3.4) closer to that required to synthesize thermoplastic PU material (ƒ = 2). The difference in chemical structure between SA and PA was evident in these reactions as solid polymer products were not obtained in these instances. Instead, the poly(SA–*co*–DCAGE) polyesters existed as viscous oils with DSC analysis confirming *T*_g_ values observed to below 0 °C (Table S9, entries 1–3).

After proving polyester from SA could be produced, LHOGE was then employed as the epoxide using the same identical reaction conditions (Figure 4). SA/LHOGE ROCOP polymerization was conducted without [PPNCl] organic catalyst to check if there was possible activity from species in the LHOGE mixture or ‘self-catalysis’ (Table S9, entry 4). There was near complete SA conversion observed after 30 min to afford a reasonable molecular-weight polyester (*M*_n_ = 3250 g·mol^−1^). The addition of two different loadings of [PPNCl] had effects on the polymerization. The higher loading ([SA]:[epoxide]: [PPNCl] = [100]:[100]:[1]) dramatically increased the activity, reduced the reaction time by half (15 min), and slightly increased the molecular weight (M_n_ = 3550 g·mol^−1^). The lower loading ([SA]:[epoxide]: [PPNCl] = [500]:[500]:[1]) required the same 30 min reaction time but markedly increased the molecular weight to 4200 g·mol^−1^. As was observed for PA, increasing the scale of the SA/LHOGE ROCOP, from 1.25 mmol to 6.81 mmol epoxide content in the [LHOGE], decreased the molecular weight (Table S9; entry 7, *M*_n_ = 2750 g·mol^−1^ vs. entry5, *M*_n_ = 3550 g·mol^−1^), as would be expected from that observed using the DCAGE substrate. The OH content of the poly(SA–*co*–LHOGE) polyesters was determined to be higher for the two polymerizations containing [PPNCl], decreasing slightly as the molecular weight increased (Table S9; entry 5, *M*_n_ = 3550 g·mol^−1^, 3.46 mmol total OH/g vs. entry 6, *M*_n_ = 4200 g·mol^−1^, 3.05 mmol total OH/g). The OH content was lower for the polymerization with no [PPNCl] despite the molecular weight being the lowest observed via GPC analysis (Table S9; entry 4, *M*_n_ = 3250 g·mol^−1^, 2.55 mmol total OH/g). Unlike the poly(SA–*co*–DCAGE) products, the poly(SA–*co*–LHOGE) products were solids at room temperature. At the 1.25 mmol [LHOGE] scale, the polymer formed without [PPNCl] displayed a higher *T*_g_ than when [PPNCl] was added (Table S9; entry 4, *T*_g_ = 25.8 °C). The *T*_g_ value increased as the *M*_n_ value increased for the two polymerizations using PPNCl (Table S9; entry 5, *T*_g_ = 11.4 °C and entry 6, *T*_g_ = 21.8 °C). With no flushing with nitrogen and conducting the ROCOP with SA in air, a higher-molecular-weight polymer was achieved for both DCAGE and LHOGE but reduced control with broader dispersities in comparison to when flushed with nitrogen (see ESI, Table S10, entries 1 and 2 vs. Table S9, entries 1 and 5); this was extreme for the LHOGE, with a broad dispersity of 3.91 observed. This was attributed to possible side reactions occurring during the polymerization. To summarize, while the ROCOP of LHOGE with PA was successful in air, the reaction with SA required flushing with N_2_ to help control the polymerization. It was not possible to determine quantitative ratios of polyester structures for poly(SA–*co*–DCAGE) and poly(SA–*co*–LHOGE) from Pathway A vs. B, as was tried for poly(PA–*co*–DCAGE) (Figure 4), because of either extensive overlapping of signals to prevent distinguishable integration or the broad nature of the ^1^H NMR signals in the spectra. However, further mechanistic investigations are currently ongoing.

### 2.7. The Application of Lignin-Derived ROCOP Polyesters as Polyols for the Synthesis of Polyurethane Film Materials

As a proof of concept, and to demonstrate that PU material could be produced by employing our functional lignin-derived polyesters as polyols, a range of thermoset PU films was synthesized and evaluated. The polyesters derived from PA, SA, DCAGE, and LHOGE that were selected as polyols are shown in Table 3. Hexamethylene diisocyanate (HDI) was chosen as the diisocyanate reagent to theoretically provide a softer repeating segment, in conjunction with the hard lignin polyester polyol segments across the material. Attempts to prepare PU films using methylene diphenyl diisocyanate (MDI) with a harder repeating segment were unsuccessful and incompatible with the already hard lignin polyester polyol segments (see ESI, Figure S31). Transparent PU film materials were successfully synthesized using HDI, with a [NCO]/[OH] ratio of 1.05 and the lignin-derived polyester as the sole polyol in dry tetrahydrofuran solvent with tin(II)-octanoate initiator (0.8 mol% OH) (see ESI, Figure S32). Complete reactivity, for the cross-linking and thermosetting, was confirmed via the disappearance of the NCO stretch observed from Fourier transform–infra red (FT–IR) spectroscopy (see ESI, Figure S34).

The differences in the mechanical properties of the PU films, produced from different polyesters, was immediately recognizable upon manual inspection. The PU films made from SA were more flexible compared to those from PA, which were more brittle. In particular, there was stark contrast between the poly(SA–*co*–DCAGE) PU film, which was very flexible, and the poly(PA–*co*–DCAGE) PU film, which was very brittle and easy to break by touch. The thermomechanical properties of all the PU film materials were screened and studied using differential scanning calorimetry (DSC) (Table 3) and dynamic mechanical thermal analysis (DMTA) (see ESI, Figure S33). Tensile strength values were calculated from their stress–strain curves (Figure 2). In all cases, DMTA observed a distinct phase transition as the storage modulus (*E’*) shifted from a glassy region, through the *T*_g_, and then plateauing in the rubbery region (see ESI, Figure S33). There was no further decrease in the *E’* and no *T*_m_ observed, confirming the materials were thermosets and that curing was successful, with all the [NCO] moieties reacting to form a highly cross-linked network. Using the peak of the tanδ curve, the average *T*_g_ values of the PU films were determined and found to range from 10.1 to 73.8 °C (Table 3). Specimens of poly(PA–*co*–DCAGE) PU were incredibly challenging to cut and test by DMTA because of brittleness. The poly(PA–*co*–LHOGE) PU film was slightly less brittle in comparison and easier to handle without breaking. PU specimens from the poly(SA–*co*–DCAGE) and poly(SA–*co*–LHOGE) polyols were tougher, more flexible, and easier to handle, particularly poly(SA–*co*–DCAGE). The molecular weight of the polyester polyol was found to influence the brittleness of the poly(PA–*co*–DCAGE) PU film. This is discussed further in the ESI.

**Figure 5:**
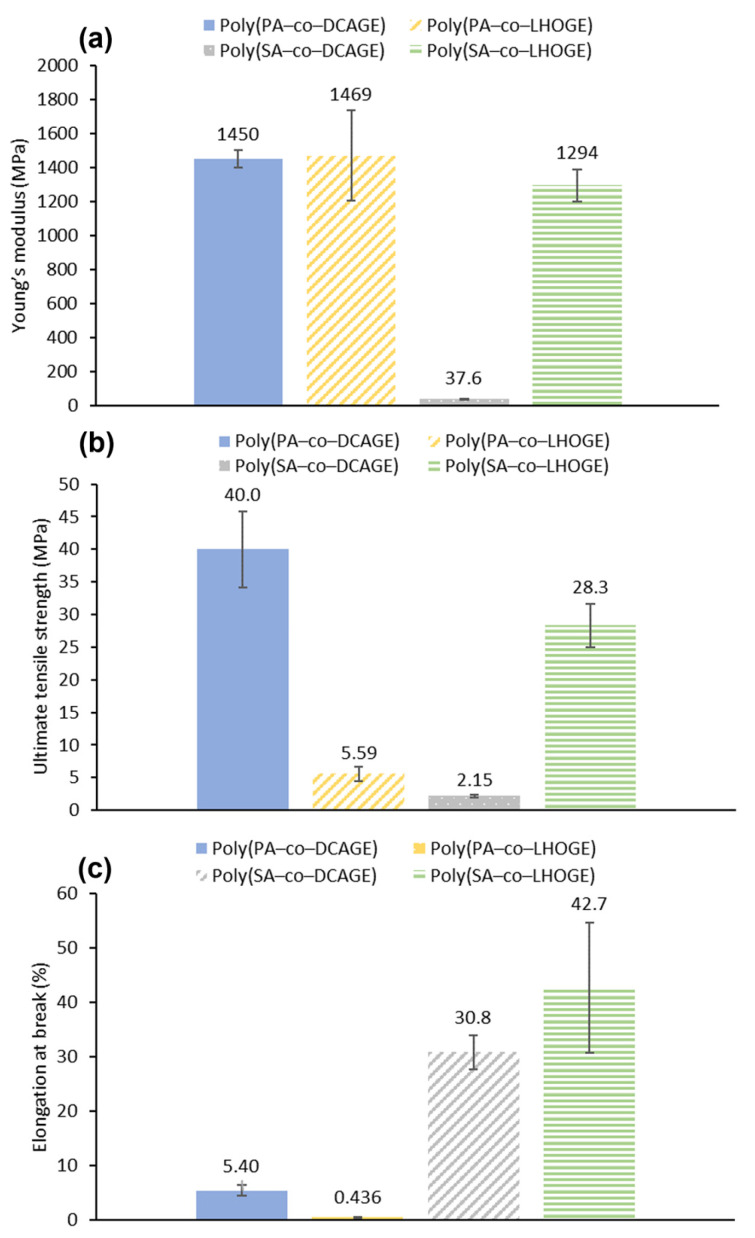
The observed mechanical properties of the different PU films, synthesized from the lignin-derived polyester polyols shown in the legends, measured using tensile stress–strain curves: (**a**) the Young’s modulus, (**b**) the ultimate tensile strength, (**c**) the elongation at break.

The mechanical properties of the PU films measured by DMTA and calculated from stress–strain curves (Figure 5) agreed with the physical observations and general handleability of the samples. All the PU films demonstrated promising properties in at least one of the mechanical parameters (Young’s modulus, ultimate tensile strength, and elongation at break). Furthermore, the results were especially encouraging as a proof of concept, considering the high lignin content ([LHOGE or DCAGE] = 46–63%) and biomass-derived content ([LHOGE or DCAGE] + [anhydride] = 75–79%) in the PU material (Table 3). The brittle nature of the PU films produced using PA was exemplified by the low elongation of break and exacerbated with the use of depolymerized lignin oil (Figure 5c). Although brittle to handle, the films were still reasonably strong, with respectable Young’s modulus and ultimate tensile strength values considering the high lignin or biomass content. However, the PU material containing [LHOGE] was considerably weaker in tensile strength compared to that from [DCAGE] (ultimate tensile strength; poly(PA–*co*–DCAGE) polyol, 40.0 ± 5.8 MPa and poly(PA–*co*–LHOGE), 5.59 ± 1.1 MPa) (Figure 5b), while the Young’s modulus was still consistent at around 1450 MPa for both (Young’s modulus; poly(PA–*co*–DCAGE) polyol, 1450 ± 51 MPa and poly(PA–*co*–LHOGE), 1469 ± 266 MPa) (Figure 5a).

Substituting the aromatic PA with aliphatic SA in the polyester resulted in the PU films becoming less brittle and more elastic as shown by the increase in the elongation at break (poly(SA–*co*–DCAGE) polyol, 30.8 ± 3.2% and poly(SA–*co*–LHOGE), 42.7 ± 12%) (Figure 5c). As a downside, for the PU film from poly(SA–*co*–DCAGE) polyol, this was accompanied by a significant decrease in the ultimate tensile strength (2.15 ± 0.17 MPa) and Young’s modulus (37.6 ± 2.7 MPa) as would be expected for a flexible, elastic material. In contrast, the PU film prepared from the poly(SA–*co*–LHOGE) polyol was found to be the most well-balanced material for the PU films synthesized in this study. Despite having a high bio-based content (79 wt%), this film had a promising combination of mechanical properties (Young’s modulus = 1294 ± 95 MPa, ultimate tensile strength = 28.3 ± 3.3 MPa and elongation at break = 42.7 ± 12%) {Figure 5a–c)}. Overall, this demonstrated how the thermomechanical properties of a resulting PU material can be tuned by varying the chemical structures/backbones of the epoxide and anhydride substrates used in the initial ROCOP reaction. Further investigations are currently ongoing in attempting to improve and optimize the PU synthesis and characterization.

## 3. Conclusions

In this proof-of-concept study, lignin-derived, fully bio-based polyesters were synthesized via a simple metal-free, organo-catalytic [PPNCl], and/or self-promoted (no [PPNCl] added) ROCOP system. This was from both glycidylated lignin models (PGGE, EGE, and DCAGE) and glycidylated depolymerized lignin (LHOGE) sourced from the RCF of native lignin from *Pinus radiata* wood biomass. The polymerizations were conducted using more robust and industrially relevant conditions with no purification of any of the commercially received reagents, catalysts, or solvents, and conducted in the majority of cases in air. This was in a bid towards attempting to make ROCOP more attractive to commercialization by showcasing its inherent advantages. The cyclic anhydrides employed (PA and SA) can be derived from the furanics sourced from the cellulosic and hemicellulosic components from lignocellulosic biomass. Hence, the biorefinery products from both the lignin and the polysaccharide components of lignocellulosic biomass can be coupled and valorized together in one polymerization reaction via ROCOP here to produce fully bio-based polymer materials. After mechanistic and NMR studies, an alternative ROCOP-style mechanism was proposed that contributes to the observed accelerated rates with added [PPNCl] organo-catalyst and ‘self- initiation/self-promoted’ ROCOP reactivity when no external [PPNCl] catalyst was added because of the likely presence of inherent [OH] groups/species in the lignin-derived glycidyl ether monomer. The value and potential of these lignin-derived polyesters was demonstrated by exploiting their characteristic functionality and applying them as intermediate polyols in the successful synthesis of four PU film materials with a high biomass content of 75–79%. A range of thermomechanical properties were observed for the materials, which demonstrated effectively how ROCOP can be used to fine-tune the structure and dramatically affect the resulting properties, of both the functional polyester and PU material, by subtle variations in the epoxide and anhydride substrate combinations employed. These findings help endeavors towards predicting the relationship between chemical structure and material thermomechanical properties and performance, relevant for industrial applications. Key characteristics such as tunability, self-promotion, rapid solvent-free polymerization, and low temperature and purity requirements, coupled with the increasing availability of biomass-derived substrates, all point towards a promising future for ROCOP processes in the preparation of a new generation of bio-based polymers. This study demonstrated for the first time that ROCOP can be successfully used to produce proof of concept functional PU materials from lignocellulosic biomass. Further investigations are needed and currently ongoing in better understanding the ROCOP reaction occurring with the lignin monomer in these studies, improving the purification protocol for polyester products and attempting to improve the PU synthesis and characterization.

## Figures and Tables

**Table 1:** PA/PGGE, EGE, and DCAGE ROCOP in optimized solution conditions for higher molecular weights using [PPNCl] organic catalyst.

Entry	Epoxide	Time (h)	Conv. ^a^ (%)	*M*_n_^b^ (Da)	Ð ^b^	*T*_g_^c^ (°C)
1	PGE	24	87	6850	1.12	43.8
2	PGGE	24	95	3400	1.31	30.2
3	EGE	24	95	3600	1.36	34.2
4	DCAGE	8	96	2700	1.96	33.8

Conditions: flushed with argon, [PA]:[Epoxide]:[PPNCl] = [2000]:[2000]:[1], 110 °C, grounded and vacuum oven dried, commercial PA (1.481 g, 10 mmol), commercial PGE (1.35 mL, 10 mmol) or synthesized PGGE (2.223 g, 10 mmol) or synthesized EGE (2.203 g, 10 mmol) or synthesized DCAGE (2.303 g, 10 mmol), [PPNCl] catalyst (0.05 mol%, 0.005 mmol), anhydrous toluene (3.0 mL). ^a^ Determined by ^1^H NMR spectroscopy (CDCl_3_) ^b^ Determined via GPC (refractive index analysis) in THF solvent. ^c^ Determined via differential scanning calorimetry.

**Table 2:** PA/DCAGE ROCOP in neat conditions and air using [PPNCl] organic catalyst with a future view of using the polyesters as polyols for PU synthesis.

Entry	[PA]:[DCAGE]:[PPNCl]	Time (min)	PA Conv ^f^ (%)	*M*_n_^g^ (Da)	Ð ^g^	Total OH Value ^h^ (mmol/g)	ƒ ^i^	*T*_g_^j^ (°C)
1 ^a^	[500]:[500]:[1]	6	88	2650	1.78	3.11	8.2	37.9
2 ^a^	[500]:[500]:[1]	10	>99	2800	1.78	3.13	8.8	37.0
3 ^b^	[250]:[250]:[1]	5	92	2650	1.79	3.07	8.1	33.0
4 ^c^	[100]:[100]:[1]	5	87	2800	1.51	3.22	9.0	31.2
5 ^d^	[100]:[100]:[1]	5	95	2050	1.52	3.46	7.1	30.5
6 ^e^	[100]:[100]:[1]	11	96	1950	1.58	2.33	4.5	29.5

Conditions in air: 110 °C and neat. ^a^ Grounded and vacuum oven dried, commercial PA (0.370 g, 2.5 mmol), synthesized DCAGE (0.596 g, 2.5 mmol), [PPNCl] catalyst (0.2 mol%, 0.005 mmol). ^b^ [PPNCl] catalyst (0.4 mol%, 0.01 mmol). ^c^ [PPNCl] catalyst (1.0 mol%, 0.025 mmol). ^d^ Grounded and vacuum oven dried, commercial PA (1.008 g, 6.81 mmol), synthesized DCAGE (1.622 g, 6.81 mmol), [PPNCl] catalyst (1.0 mol%, 0.0681 mmol). ^e^ Grounded and vacuum oven dried, commercial PA (3.111 g, 21.0 mmol), synthesized DCAGE (5.005 g, 21.0 mmol), [PPNCl] catalyst (1.0 mol%, 0.21 mmol). ^f^ Determined by ^1^H NMR spectroscopy (CDCl_3_). ^g^ Determined via GPC (refractive index analysis) in THF solvent. ^h^ Determined via phosphitylation and quantitative ^31^P NMR spectroscopy. ^i^ Chemical (OH) functionality, *f*_OH_ = (total OH value (mmol/g) × *M*_n_ of the polymer (g·mol^−1^))/1000. ^j^ Determined via differential scanning calorimetry.

**Table 3:** Summary of the PU film materials synthesized from the functional lignin-derived polyesters produced in this study, with their biomass content and thermal properties.

Polyester Polyol	Poly(PA–*co*–DCAGE)	Poly(PA-*co*-LHOGE)	Poly(SA–*co*–DCAGE)	Poly(SA–*co*–LHOGE)
Polyester sample source	Table 2, entry 4	Table S8, entry 6	Table S9, entry 3	Table S9, entry 7
*M*_n_ (Da)	2800	2450	1100	2750
[DCAGE/LHOGE content] (wt%)	46	57	55	63
[Biomass-derived content] (wt%)	75	77	78	79
*T*_g,tanδ_ (°C)	73.8 ± 0.61	66.9 ± 1.4	10.1 ± 0.80	62.5 ± 1.6

## Data Availability

The data supporting this article have been included as part of the Supplementary Information.

## Supplementary Materials

The following supporting information can be downloaded at: https://www.mdpi.com/article/10.3390/polym17202806/s1, The materials and methods section, supplementary results and discussion related to the main text, experimental section and details, and characterization data such as DSC, GPC, NMR, FT–IR, and DMTA can be found in the supporting information. References [105,106,107,108] are cited in the supplementary materials.

## References

[1] O’Dea R.M., Willie J.A., Epps T.H. (2020). 100th Anniversary of Macromolecular Science Viewpoint: Polymers from Lignocellulosic Biomass. Current Challenges and Future Opportunities. ACS Macro Lett..

[2] Zhu Y., Romain C., Williams C.K. (2016). Sustainable Polymers from Renewable Resources. Nature.

[3] Jones M.D., Payne J. (2021). The Chemical Recycling of Polyesters for a Circular Plastics Economy: Challenges and Emerging Opportunities. ChemSusChem.

[4] Payne J., Mckeown P., Jones M.D. (2019). A Circular Economy Approach to Plastic Waste. Polym. Degrad. Stab..

[5] Ellen MacArthur Foundation (2016). The New Plastics Economy: Rethinking the Future of Plastics.

[6] Rabnawaz M., Wyman I., Auras R., Cheng S. (2017). A Roadmap towards Green Packaging: The Current Status and Future Outlook for Polyesters in the Packaging Industry. Green. Chem..

[7] Sanford M.J., Peña Carrodeguas L., van Zee N.J., Kleij A.W. (2016). Alternating Copolymerization of Propylene Oxide and Cyclohexene Oxide with Tricyclic Anhydrides: Access to Partially Renewable Aliphatic Polyesters with High Glass Transition Temperatures. Macromolecules.

[8] Müller R.J., Kleeberg I., Deckwer W.D. (2001). Biodegradation of Polyesters Containing Aromatic Constituents. J. Biotechnol..

[9] Vert M. (2005). Aliphatic Polyesters: Great Degradable Polymers That Cannot Do Everything. Biomacromolecules.

[10] Brown A.H., Sheares V.V. (2007). Amorphous Unsaturated Aliphatic Polyesters Derived from Dicarboxylic Monomers Synthesized by Diels-Alder Chemistry. Macromolecules.

[11] Olson D.A., Gratton S.E.A., DeSimone J.M., Sheares V.V. (2006). Amorphous Linear Aliphatic Polyesters for the Facile Preparation of Tunable Rapidly Degrading Elastomeric Devices and Delivery Vectors. J. Am. Chem. Soc..

[12] Ryzhakov D., Printz G., Jacques B., Messaoudi S., Dumas F., Dagorne S., Le Bideau F. (2021). Organo-Catalyzed/Initiated Ring Opening Co-Polymerization of Cyclic Anhydrides and Epoxides: An Emerging Story. Polym. Chem..

[13] Longo Julie M., Sanford M.J., Coates G.W. (2016). Ring-Opening Copolymerization of Epoxides and Cyclic Anhydrides with Discrete Metal Complexes: Structure−Property Relationships. Chem. Rev..

[14] Lidston C.A.L., Severson S.M., Abel B.A., Coates G.W. (2022). Multifunctional Catalysts for Ring-Opening Copolymerizations. ACS Catal..

[15] Thomas C.M. (2010). Stereocontrolled Ring-Opening Polymerization of Cyclic Esters: Synthesis of New Polyester Microstructures. Chem. Soc. Rev..

[16] Stanford M.J., Dove A.P. (2010). Stereocontrolled Ring-Opening Polymerisation of Lactide. Chem. Soc. Rev..

[17] Dechy-Cabaret O., Martin-Vaca B., Bourissou D. (2004). Controlled Ring-Opening Polymerization of Lactide and Glycolide. Chem. Rev..

[18] Paul S., Zhu Y., Romain C., Brooks R., Saini P.K., Williams C.K. (2015). Ring-Opening Copolymerization (ROCOP): Synthesis and Properties of Polyesters and Polycarbonates. Chem. Commun..

[19] Gregory G.L., Sulley G.S., Carrodeguas L.P., Chen T.T.D., Santmarti A., Terrill N.J., Lee K.-Y., Williams C.K. (2020). Triblock Polyester Thermoplastic Elastomers with Semi-Aromatic Polymer End Blocks by Ring-Opening Copolymerization. Chem. Sci..

[20] Kummari A., Pappuru S., Chakraborty D. (2018). Fully Alternating and Regioselective Ring-Opening Copolymerization of Phthalic Anhydride with Epoxides Using Highly Active Metal-Free Lewis Pairs as a Catalyst. Polym. Chem..

[21] Brochu S., Prud’homme R.E., Barakat I., Jérôme R. (1995). Stereocomplexation and Morphology of Polylactides. Macromolecules.

[22] Tsuji H. (2005). Poly(Lactide) Stereocomplexes: Formation, Structure, Properties, Degradation, and Applications. Macromol. Biosci..

[23] Garlotta D. (2001). Literature Review of Poly (Lactic Acid). J. Polym. Environ..

[24] Nampoothiri M.K., Nair N.R., John R.P. (2010). An Overview of the Recent Developments in Polylactide (PLA) Research. Bioresour. Technol..

[25] Stolt M., Södergård A. (2002). Properties of Lactic Acid Based Polymers and Their Correlation with Composition. Prog. Polym. Sci..

[26] Rieger J. (1996). The Glass Transition Temperature of Polystyrene. J. Thermal. Anal..

[27] Hosseini Nejad E., Paoniasari A., Koning C.E., Duchateau R. (2012). Semi-Aromatic Polyesters by Alternating Ring-Opening Copolymerisation of Styrene Oxide and Anhydrides. Polym. Chem..

[28] Nejad Elham H., van Melis Carlo G.W., Vermeer T.J., Koning C.E., Duchateau R. (2012). Alternating Ring-Opening Polymerization of Cyclohexene Oxide and Anhydrides: Effect of Catalyst, Cocatalyst, and Anhydride Structure. Macromolecules.

[29] Nejad E.H., Paoniasari A., Van Melis C.G.W., Koning C.E., Duchateau R. (2013). Catalytic Ring-Opening Copolymerization of Limonene Oxide and Phthalic Anhydride: Toward Partially Renewable Polyesters. Macromolecules.

[30] Mundil R., Hošťálek Z., Šeděnková I., Merna J. (2015). Alternating Ring-Opening Copolymerization of Cyclohexene Oxide with Phthalic Anhydride Catalyzed by Iron(III) Salen Complexes. Macromol. Res..

[31] Stößer T., Williams C.K. (2018). Selective Polymerization Catalysis from Monomer Mixtures: Using a Commercial Cr-Salen Catalyst To Access ABA Block Polyesters. Angew. Chem. Int. Ed..

[32] Bester K., Bukowska A., Myśliwiec B., Hus K., Tomczyk D., Urbaniak P., Bukowski W. (2018). Alternating Ring-Opening Copolymerization of Phthalic Anhydride with Epoxides Catalysed by Salophen Chromium(III) Complexes. Eff. Substituents Salophen Ligands. Polym. Chem..

[33] Winkler M., Romain C., Meier M.A.R., Williams C.K. (2015). Renewable Polycarbonates and Polyesters from 1,4-Cyclohexadiene. Green. Chem..

[34] Williams C.K., Hillmyer M.A. (2008). Polymers from Renewable Resources: A Perspective for a Special Issue of Polymer Reviews. Polym. Rev..

[35] Brandolese A., della Monica F., Pericàs M.À., Kleij A.W. (2022). Catalytic Ring-Opening Copolymerization of Fatty Acid Epoxides: Access to Functional Biopolyesters. Macromolecules.

[36] Liu B., Chen J., Liu N., Ding H., Wu X., Dai B., Kim I. (2020). Bio-Based Polyesters Synthesized by Ring-Opening Copolymerizations of Eugenyl Glycidyl Ether and Cyclic Anhydrides Using a Binuclear [OSSO] CrCl Complex. Green. Chem..

[37] Peña Carrodeguas L., Martín C., Kleij A.W. (2017). Semiaromatic Polyesters Derived from Renewable Terpene Oxides with High Glass Transitions. Macromolecules.

[38] Monica F.D., Kleij A.W. (2021). Synthesis and Characterization of Biobased Polyesters with Tunable Tgby ROCOP of Beta-Elemene Oxides and Phthalic Anhydride. ACS Sustain. Chem. Eng..

[39] Chen T.T.D., Carrodeguas L.P., Sulley G.S., Gregory G.L., Williams C.K. (2020). Bio-based and Degradable Block Polyester Pressure-Sensitive Adhesives. Angew. Chem. Int. Ed..

[40] Haslewood M.N.D., Farmer T.J., North M. (2022). Synthesis and Chemoselective Crosslinking of Functionalized Polyesters from Bio-Based Epoxides and Cyclic Anhydrides. J. Polym. Sci..

[41] Pagliaro M., Ciriminna R., Kimura H., Rossi M., Della Pina C. (2007). From Glycerol to Value-Added Products. Angew. Chem. Int. Ed..

[42] Solvay Epicerol® Earns Roundtable on Sustainable Biomaterials Certification. https://www.solvay.com/en/press-release/solvay-epicerol-earns-roundtable-sustainable-biomaterials-certification.

[43] INOVYN Launches World’s First Commercially Available Grade of Bio-Attributed Epichlorohydrin. https://www.ineos.com/businesses/inovyn/news/inovyn-launches-worlds-first-commercially-available-grade-of-bio-attributed-epichlorohydrin/.

[44] Faye I., Decostanzi M., Ecochard Y., Caillol S. (2017). Eugenol Bio-Based Epoxy Thermosets: From Cloves to Applied Materials. Green. Chem..

[45] Morales-Cerrada R., Molina-Gutierrez S., Lacroix-Desmazes P., Caillol S. (2021). Eugenol, a Promising Building Block for Biobased Polymers with Cutting-Edge Properties. Biomacromolecules.

[46] Nghiem N., Kleff S., Schwegmann S. (2017). Succinic Acid: Technology Development and Commercialization. Fermentation.

[47] Putri D.N., Sahlan M., Montastruc L., Meyer M., Negny S., Hermansyah H. (2020). Progress of Fermentation Methods for Bio-Succinic Acid Production Using Agro-Industrial Waste by Actinobacillus Succinogenes. Energy Rep..

[48] Dickson R., Mancini E., Garg N., Woodley J.M., Gernaey K.V., Pinelo M., Liu J., Mansouri S.S. (2021). Sustainable Bio-Succinic Acid Production: Superstructure Optimization, Techno-Economic, and Lifecycle Assessment. Energy Environ. Sci..

[49] Pietraru M., Lentz N., Ponsard L., Nicolas E., Cantat T. (2023). Catalytic Carbonylation of Acrylic Acid to Succinic Anhydride. ChemCatChem.

[50] Driscoll O.J., Stewart J.A., McKeown P., Jones M.D. (2021). Ring-Opening Copolymerization Using Simple Fe(III) Complexes and Metal- and Halide-Free Organic Catalysts. Macromolecules.

[51] Hauenstein O., Reiter M., Agarwal S., Rieger B., Greiner A. (2016). Bio-Based Polycarbonate from Limonene Oxide and CO_2_ with High Molecular Weight, Excellent Thermal Resistance, Hardness and Transparency. Green. Chem..

[52] Hošťálek Z., Trhlíková O., Walterová Z., Martinez T., Peruch F., Cramail H., Merna J. (2017). Alternating Copolymerization of Epoxides with Anhydrides Initiated by Organic Bases. Eur. Polym. J..

[53] Robert C., Ohkawara T., Nozaki K. (2014). Manganese-Corrole Complexes as Versatile Catalysts for the Ring-Opening Homo- and Co-Polymerization of Epoxide. Chem. Eur. J..

[54] Nakano K., Kobayashi K., Ohkawara T., Imoto H., Nozaki K. (2013). Copolymerization of Epoxides with Carbon Dioxide Catalyzed by Iron-Corrole Complexes: Synthesis of a Crystalline Copolymer. J. Am. Chem. Soc..

[55] Saini P.K., Romain C., Zhu Y., Williams C.K. (2014). Di-Magnesium and Zinc Catalysts for the Copolymerization of Phthalic Anhydride and Cyclohexene Oxide. Polym. Chem..

[56] Diciccio A.M., Coates G.W. (2011). Ring-Opening Copolymerization of Maleic Anhydride with Epoxides: A Chain-Growth Approach to Unsaturated Polyesters. J. Am. Chem. Soc..

[57] Hu L., Zhang C., Wu H., Yang J., Liu B., Duan H., Zhang X. (2018). Highly Active Organic Lewis Pairs for the Copolymerization of Epoxides with Cyclic Anhydrides: Metal-Free Access to Well-Defined Aliphatic Polyesters. Macromolecules.

[58] Darensbourg D.J., Poland R.R., Escobedo C. (2012). Kinetic Studies of the Alternating Copolymerization of Cyclic Acid Anhydrides and Epoxides, and the Terpolymerization of Cyclic Acid Anhydrides, Epoxides, and CO2 Catalyzed by (Salen)CrIIICl. Macromolecules.

[59] Shi Z., Jiang Q., Song Z., Wang Z., Gao C. (2018). Dinuclear Iron(III) Complexes Bearing Phenylene—Bridged Bis(Amino Triphenolate) Ligands as Catalysts for the Copolymerization of Cyclohexene Oxide with Carbon Dioxide or Phthalic Anhydride. Polym. Chem..

[60] Bio-Based, Commercial Itaconic Anhydride (Merck Ltd., Product Number: 259926). https://www.sigmaaldrich.com/GB/en/product/aldrich/259926.

[61] Zhao M., Lu X., Zong H., Li J., Zhuge B. (2018). Itaconic Acid Production in Microorganisms. Biotechnol. Lett..

[62] Kolankowski K., Miętus M., Ruśkowski P., Gadomska-Gajadhur A. (2022). Optimisation of Glycerol and Itaconic Anhydride Polycondensation. Molecules.

[63] Shang S., Huang S.J., Weiss R.A. (2009). Synthesis and Characterization of Itaconic Anhydride and Stearyl Methacrylate Copolymers. Polymer.

[64] Okuda T., Ishimoto K., Ohara H., Kobayashi S. (2012). Renewable Biobased Polymeric Materials: Facile Synthesis of Itaconic Anhydride-Based Copolymers with Poly(L-Lactic Acid) Grafts. Macromolecules.

[65] Schutyser W., Renders T., Van Den Bosch S., Koelewijn S.F., Beckham G.T., Sels B.F. (2018). Chemicals from Lignin: An Interplay of Lignocellulose Fractionation, Depolymerisation, and Upgrading. Chem. Soc. Rev..

[66] Liu X., Bouxin F.P., Fan J., Budarin V.L., Hu C., Clark J.H. (2020). Recent Advances in the Catalytic Depolymerization of Lignin towards Phenolic Chemicals: A Review. ChemSusChem.

[67] Torr K.M., Driscoll O.J., van de Pas D.J., Feghali E. (2024). Recent Advances in Thermoset and Thermoplastic Polymeric Materials Produced Using Technical and Depolymerized Native Lignins. Lignin Chemistry.

[68] Sun Z., Fridrich B., de Santi A., Elangovan S., Barta K. (2018). Bright Side of Lignin Depolymerization: Toward New Platform Chemicals. Chem. Rev..

[69] Wang Y.-Y., Meng X., Pu Y., J Ragauskas A. (2020). Recent Advances in the Application of Functionalized Lignin in Value-Added Polymeric Materials. Polymers.

[70] Yu O., Kim K.H. (2020). Lignin to Materials: A Focused Review on Recent Novel Lignin Applications. Appl. Sci..

[71] Feghali E., van de Pas D.J., Torr K.M. (2020). Toward Bio-Based Epoxy Thermoset Polymers from Depolymerized Native Lignins Produced at the Pilot Scale. Biomacromolecules.

[72] Quinsaat J.E.Q., Feghali E., van de Pas D.J., Vendamme R., Torr K.M. (2021). Preparation of Mechanically Robust Bio-Based Polyurethane Foams Using Depolymerized Native Lignin. ACS Appl. Polym. Mater..

[73] Chio C., Sain M., Qin W. (2019). Lignin Utilization: A Review of Lignin Depolymerization from Various Aspects. Renew. Sustain. Energy Rev..

[74] Korányi T.I., Fridrich B., Pineda A., Barta K. (2020). Development of ‘Lignin-First’ Approaches for the Valorization of Lignocellulosic Biomass. Molecules.

[75] Rao N., Guo Y., Li H., Chen Q., Li Y., Huang Q., Zhao Z.K., Xie H. (2024). Synthesis of Multifunctional Lignin Graft Alternating Copolymers of Cyclic Anhydrides and Epoxides Catalyzed by Metal-Free Lewis Pairs. Ind. Crops Prod..

[76] Shao X., Su L., Zhang J., Tian Z., Zhang N., Wang Y., Wang H., Cui X., Hou X., Deng T. (2021). Green Production of Phthalic Anhydride from Biobased Furan and Maleic Anhydride by an Acid Resin Catalyst. ACS Sustain. Chem. Eng..

[77] Mahmoud E., Watson D.A., Lobo R.F. (2014). Renewable Production of Phthalic Anhydride from Biomass-Derived Furan and Maleic Anhydride. Green. Chem..

[78] Lin Z., Ierapetritou M., Nikolakis V. (2015). Phthalic Anhydride Production from Hemicellulose Solutions: Technoeconomic Analysis and Life Cycle Assessment. AIChE J..

[79] Settle A.E., Berstis L., Rorrer N.A., Roman-Leshkóv Y., Beckham G.T., Richards R.M., Vardon D.R. (2017). Heterogeneous Diels–Alder Catalysis for Biomass-Derived Aromatic Compounds. Green. Chem..

[80] (R,R)-N,N′-Bis(3,5-di-tert-butylsalicylidene)-1,2-cyclohexanediaminochromium(III) chloride (Merck Ldt., Product Number: 531944). https://www.sigmaaldrich.com/GB/en/product/aldrich/531944.

[81] Denny J. Biobased Phthalic Anhydride: Can This Key Feedstock Be Green?. https://www.orbichem.com/blog/a-green-future-for-phthalic-anhydride-biomass-boost.

[82] Thiyagarajan S., Genuino H.C., Śliwa M., van der Waal J.C., de Jong E., van Haveren J., Weckhuysen B.M., Bruijnincx P.C.A., van Es D.S. (2015). Substituted Phthalic Anhydrides from Biobased Furanics: A New Approach to Renewable Aromatics. ChemSusChem.

[83] Yu X., Jia J., Xu S., Lao K.U., Sanford M.J., Ramakrishnan R.K., Nazarenko S.I., Hoye T.R., Coates G.W., DiStasio R.A. (2018). Unraveling Substituent Effects on the Glass Transition Temperatures of Biorenewable Polyesters. Nat. Commun..

[84] Van den Bosch S., Schutyser W., Koelewijn S.-F., Renders T., Courtin C.M., Sels B.F. (2015). Tuning the Lignin Oil OH-Content with Ru and Pd Catalysts during Lignin Hydrogenolysis on Birch Wood. Chem. Commun..

[85] Parsell T., Yohe S., Degenstein J., Jarrell T., Klein I., Gencer E., Hewetson B., Hurt M., Kim J.I., Choudhari H. (2015). A Synergistic Biorefinery Based on Catalytic Conversion of Lignin Prior to Cellulose Starting from Lignocellulosic Biomass. Green. Chem..

[86] Zhang J., Wang L., Liu S., Li Z. (2020). Phosphazene/Lewis Acids as Highly Efficient Cooperative Catalyst for Synthesis of High-Molecular-Weight Polyesters by Ring-Opening Alternating Copolymerization of Epoxide and Anhydride. J. Polym. Sci..

[87] Han B., Zhang L., Liu B., Dong X., Kim I., Duan Z., Theato P. (2015). Controllable Synthesis of Stereoregular Polyesters by Organocatalytic Alternating Copolymerizations of Cyclohexene Oxide and Norbornene Anhydrides. Macromolecules.

[88] Ji H.-Y., Chen X.-L., Wang B., Pan L., Li Y.-S. (2018). Metal-Free, Regioselective and Stereoregular Alternating Copolymerization of Monosubstituted Epoxides and Tricyclic Anhydrides. Green. Chem..

[89] Pappuru S., Chakraborty D. (2019). Progress in Metal-Free Cooperative Catalysis for the Ring-Opening Copolymerization of Cyclic Anhydrides and Epoxides. Eur. Polym. J..

[90] Torr K.M., van de Pas D.J., Cazeils E., Suckling I.D. (2011). Mild Hydrogenolysis of In-Situ and Isolated Pinus Radiata Lignins. Bioresour. Technol..

[91] Van den Bosch S., Schutyser W., Vanholme R., Driessen T., Koelewijn S.-F., Renders T., De Meester B., Huijgen W.J.J., Dehaen W., Courtin C.M. (2015). Reductive Lignocellulose Fractionation into Soluble Lignin-Derived Phenolic Monomers and Dimers and Processable Carbohydrate Pulps. Energy Environ. Sci..

[92] Driscoll O.J., Van Hecke K., Vande Velde C.M.L., Blockhuys F., Rubens M., Kuwaba T., van de Pas D.J., Eevers W., Vendamme R., Feghali E. (2024). Solid-State Structures and Properties of Lignin Hydrogenolysis Oil Compounds: Shedding a Unique Light on Lignin Valorization. Int. J. Mol. Sci..

[93] Quinsaat J.E.Q., Falireas P.G., Feghali E., Torr K.M., Vanbroekhoven K., Eevers W., Vendamme R. (2023). Depolymerised Lignin Oil: A Promising Building Block towards Thermoplasticity in Polyurethanes. Ind. Crops Prod..

[94] Wu X., Chen C., Guo Z., North M., Whitwood A.C. (2019). Metal- and Halide-Free Catalyst for the Synthesis of Cyclic Carbonates from Epoxides and Carbon Dioxide. ACS Catal..

[95] Inoue S. (2000). Immortal Polymerization: The Outset, Development, and Application. J. Polym. Sci. A Polym. Chem..

[96] Byers J.A., Biernesser A.B., Delle Chiaie K.R., Kaur A., Kehl J.A., Di Lorenzo M.L., Androsch R. (2018). Catalytic Systems for the Production of Poly (Lactic Acid). Advances in Polymer Science 279: Synthesis, Structure and Properties of Poly(lactic acid).

[97] Shellard E.J.K., Diment W.T., Resendiz-Lara D.A., Fiorentini F., Gregory G.L., Williams C.K. (2024). Al(III)/K(I) Heterodinuclear Polymerization Catalysts Showing Fast Rates and High Selectivity for Polyester Polyols. ACS Catal..

[98] Lidston C.A.L., Abel B.A., Coates G.W. (2020). Bifunctional Catalysis Prevents Inhibition in Reversible-Deactivation Ring-Opening Copolymerizations of Epoxides and Cyclic Anhydrides. J. Am. Chem. Soc..

[99] Hu C., Chen X., Niu M., Zhang Q., Duan R., Pang X. (2022). Immortal Ring-Opening Copolymerization of Epoxide and Isocyanate Using a Commercial Manganese Catalyst. Macromolecules.

[100] Sanford M.J., Van Zee N.J., Coates G.W. (2018). Reversible-Deactivation Anionic Alternating Ring-Opening Copolymerization of Epoxides and Cyclic Anhydrides: Access to Orthogonally Functionalizable Multiblock Aliphatic Polyesters. Chem. Sci..

[101] Vendamme R., Olaerts K., Gomes M., Degens M., Shigematsu T., Eevers W. (2012). Interplay Between Viscoelastic and Chemical Tunings in Fatty-Acid-Based Polyester Adhesives: Engineering Biomass toward Functionalized Step-Growth Polymers and Soft Networks. Biomacromolecules.

[102] Scarica C., Suriano R., Levi M., Turri S., Griffini G. (2018). Lignin Functionalized with Succinic Anhydride as Building Block for Biobased Thermosetting Polyester Coatings. ACS Sustain. Chem. Eng..

[103] Vendamme R., Eevers W. (2013). Sweet Solution for Sticky Problems: Chemoreological Design of Self-Adhesive Gel Materials Derived from Lipid Biofeedstocks and Adhesion Tailoring via Incorporation of Isosorbide. Macromolecules.

[104] Quinsaat J.E.Q., Feghali E., van de Pas D.J., Vendamme R., Torr K.M. (2022). Preparation of Biobased Nonisocyanate Polyurethane/Epoxy Thermoset Materials Using Depolymerized Native Lignin. Biomacromolecules.

[105] van de Pas D.J., Nanayakkara B., Suckling I.D., Torr K.M. (2014). Comparison of Hydrogenolysis with Thioacidolysis for Lignin Structural Analysis. Holzforschung.

[106] Gracia-Vitoria J., Rubens M., Feghali E., Adriaensens P., Vanbroekhoven K., Vendamme R. (2022). Low-Field Benchtop versus High-Field NMR for Routine 31P Analysis of Lignin, a Comparative Study. Ind. Crops Prod..

[107] Granata A., Argyropoulos D.S. (1995). 2-Chloro-4,4,5,5-Tetramethyl-1,3,2-Dioxaphospholane, a Reagent for the Accurate Determination of the Uncondensed and Condensed Phenolic Moieties in Lignins. J. Agric. Food Chem..

[108] Meng X., Crestini C., Ben H., Hao N., Pu Y., Ragauskas A.J., Argyropoulos D.S. (2019). Determination of Hydroxyl Groups in Biorefinery Resources via Quantitative 31P NMR Spectroscopy. Nat. Protoc..

