# Supplementary material for: An Integrated Biorefinery Proof of Concept: The Synthesis of Fully Bio-Based, Functional Lignin Polyester Copolymers of Cyclic Anhydrides and Epoxides Towards Polyol Applications and Tunable Bio-Derived Materials
Source: Polymers (Basel). 2025 Oct 21;17(20):2806. doi: 10.3390/polym17202806 (PMC12567910; doi:10.3390/polym17202806)
Supplement: Supplementary file 1 [file polymers-17-02806-s001.zip › polymers-3904282-supplementary.pdf]

Supplementary Information for

An Integrated Biorefinery Proof of Concept: The Synthesis of Fully Bio-Based, Functional Lignin Polyester Copolymers of Cyclic Anhydrides and Epoxides Towards Polyol Applications and Tunable Bio-Derived Materials

Contents

	Pages
S1. Materials and methods	3–7
S1.1. Materials, methods and general considerations	3–5
S1.2. ROCOP procedures and PU synthesis procedure	5–7
S2. Supplementary results and discussion from the main text (Tables and Figures)	8–41
S2.1. Development of a catalytic ROCOP system for lignin model epoxide compounds and phthalic anhydride (Tables S1–3 and Figure S1–3)	8–11
S2.2. Exploring organo–catalyzed PA / DCAGE ROCOP with views to further material applications (Figures S4–5)	12
S2.3. Blank control ROCOP reactions, chain–transfer reactions, self–catalysis/promoted ROCOP and [OH] species (Tables S4–6)	13–15
S2.4. Mechanistic considerations: Why the increase in rate with the presence of [OH] groups, the role of the [OH] groups and an alternative ROCOP mechanism pro-posed inherent for lignin (Figures S6–14 and Table S7)	16–20
S2.5. Organo–catalyzed ROCOP with PA and LHOGE sourced from the RCF of Pinus radiata biomass and glycidylation (Tables S8)	21–22
S2.6. Organo–catalyzed ROCOP of DCAGE and LHOGE with succinic anhydride (Tables S9–10)	23–25
S2.7. Breakdown of the OH contents determined, using phosphitylation and quantitative ^{31}P NMR spectroscopy, of the polyesters (Table S11)	26–27
S2.8. Further NMR spectroscopic studies for the mechanistic elucidation of the reaction pathway (Figures S22–30 and Tables S12–13)	28–36
S2.9. The application of lignin–derived ROCOP polyesters as polyols for the synthesis of polyurethane film materials (Figures S31–34 and Table S14)	37–41
S3. Characterization data	42–89
S3.1. ROCOP and polyester characterization	42–84
S3.1.1. Crude ^{1}H NMR spectra of ROCOP reaction mixtures and of isolated polymers (Figures S35–66)	42–58
S3.1.2. Phosphitylation and quantitative ^{31}P NMR spectra (Figures 67–74)	58–62
S3.1.3. FT–IR spectra of lignin, lignin-derived polyesters and polyurethane film materials (Figures S75–S93)	62–71
S3.1.4. DSC traces of polyesters and PU films (Figures S94–S111)	72–77
S3.1.5. GPC chromatograms (Figures S112–S139)	78–84
S3.2. Polyurethane material and thermomechanical characterization: DMTAs and tensile profiles of the PU films (Figures S140–S144))	85–89
S3.3. Lignin model compound synthesis and characterization	90
S4. References	91–92

S1. Materials and methods

S1.1. Materials, methods and general considerations

All the chemicals were commercially obtained from Merck® (Sigma–Aldrich®) or VWR® and used as received unless stated otherwise. The phthalic anhydride that was grounded up, dried in a vacuum oven overnight, and stored in a desiccator before use to remove residual water, which was observed *via* NMR spectroscopy. Propyl guaiacol glycidyl ether, eugenyl glycidyl ether, dihydroconiferyl alcohol glycidyl ether and lignin hydrogenolysis oil glycidyl ether, and lignin hydrogenolysis oil were prepared following previously reported protocols for glycidylation.[7,8] As per a standard protocol of purification employed in the literature, for the isolation of purified polymer from the crude ROCOP reaction mixtures, to ensure all residual catalyst, co–catalyst and unreacted reagents were removed, this was achieved by dissolving the crude product in dichloromethane and precipitating the polymer product using acidified methanol (2 M).[1–5] The Fourier transform–infrared spectroscopy (FT–IR) was performed using a Thermo Fischer Nicolet iS10 or Bruker Tensor 27 spectrometer and was measured using attenuated total reflectance mode (ATR–FTIR) under standard atmospheric conditions on a diamond crystal with a spectral range of 400–4000 cm^{-1}. The scan resolution was 4 cm^{-1} with 32 scans per sample, and the spectra were baseline corrected. The Differential Scanning Calorimetry (DSC) analyses were recorded on a TA Instruments Discovery DSC 250 or Discovery DSC instrument with the samples placed in T_{zero} pans with T_{zero} hermetic lids perforated to allow for a nitrogen atmosphere upon measurement. The sample was cooled to -60 °C at 10 °C/min, equilibrated and held at this temperature for 2 minutes, heated to 150 °C at 10 °C/min (1st heating cycle), held at this temperature for 2 minutes, cooled to -60 °C at 10 °C/min, equilibrated and held at this temperature for 2 minutes, and heated to 150 °C at 10 °C/min (2nd heating cycle). The T_g values were determined from the 2nd heating cycle. NMR spectroscopy was recorded on a Bruker AVIII 400 MHz or benchtop Magritek Spinsolve 80 MHz Ultra series Phosphor instrument or Bruker 600 MHz Avance NEO spectrometer equipped (with a dual channel BBO iProbe) and referenced to residual solvent signals such as $CHCl_3$. Phosphitylation and quantitative ^{31}P NMR spectroscopy, using chromium acetylacetone, endo–*N*–hydroxy–5–norbornene–2,3–dicarboximide, pyridine, deuterated

chloroform ($CDCl_3$) and 2–chloro–4,4,5,5–tetramethyl–1,3,2–dioxaphospholane, were conducted and analyzed following the reported literature.[9–12]. The preparation of the LHO by the depolymerization of native *Pinus radiata* lignin, *via* RCF, is reported from earlier work.[8] In this study, the LHO was that denoted as LHO4 in this previous study.[8] The glycidylation of the LHO and preparation of the LHOGE is also reported from earlier work.[7] The epoxide equivalent weight (EEW) and epoxide content of the LHOGE samples were determined in triplicate by potentiometric titration following ASTM Standard D 1652–97 (1997) scaled down to analyse 30–40 mg samples. Dynamic mechanical thermal analysis (DMTA) was conducted using an RSA–G2 TA instrument in tensile mode and rectangular geometry. The ramp rate was 2.0 °C/min with a frequency of 1.0 Hz. Liquid nitrogen cooling was employed in cases to allow for a -20 °C start temperature if required. Each material sample was measured at least three times to obtain an average value and the standard deviation error for the T_g. The DMTA was also employed for the tensile strength testing *via* measuring the stress–strain curves at 23 °C and 50% humidity at a rate of 0.002–0.1 mm/s depending on the brittleness or flexibility of the film sample. Each sample was measured 4–5 times to obtain average values and the standard deviation error for the ultimate tensile strength, Young's modulus, and elongation at break. Samples were cut with the help of an ISO 527–2 / ISO 37–4 metal cutter or die (PIONEER Die–tecs®) with sample dimensions of *ca*. 12 mm length x 2 mm width. The exception being the PU film synthesized from poly(PA–co–DCAGE) (*Table 2*, entry 4) where, due to limited film sample, small, thin rectangular samples, as similar in size as possible, were cut with a scalpel. Despite this, this sample was still tested 4 times to obtain an average value with a low standard deviation error for the tensile parameters. LC–MS was performed on a Thermo Orbitrap Q Exactive coupled with a Dionex UPLC–MS. A reversed phase LC measurement was performed using an Acquity BEH C18 100 x 2.1 mm 1.7 µm column with 10 mMH_4Ac as buffer A and CH_3CN as buffer B. With gradient: t= 0 min: 2% B, t= 2 min: 2% B, t= 10 min 100% B, t = 12 min 100% B, t= 12.1 min: 2% B, t= 15 min: 2% B. The column temperature was 60 °C, and 5 µl was injected. Measurements were performed using ESI in positive and negative ion mode. Gel permeation chromatography (GPC) was performed on a Shimadzu system using a combination of three Styragel columns (guard–HRE4–HRE4 or guard–

HR0.5–HR1) connected to a refractive index (RI) and UV detector. The instrument was calibrated with polystyrene (PS) internal standards (162–204000 g.mol^{-1} for the HRE4–HRE4 columns or 162–12980 g.mol^{-1} for the HR0.5–HR1 columns) and eluted with THF solvent. The molecular weight (M_n) and dispersity (Đ) values were obtained from the RI detector. Characterization assistance for the GPC and LC–MS was carried out through the GOAL department at VITO.

S1.2. ROCOP procedures and PU synthesis procedure

General procedure for the organo–catalyzed ROCOP in air under more robust and industrially relevant conditions

To a 20 mL vial, the chosen epoxide was added, followed by anhydride, commercial *bis*(triphenylphosphoranylidene)iminium chloride and magnetic stirring bar; for amounts, see the relevant table. Either the crude reaction mixture was left neat, solvent–free, or with standard-grade toluene solvent. The vial was sealed with a cap and placed in a preheated heating block. The crude reaction mixture in the vial was stirred at the desired temperature (typically 110 °C; in the neat conditions, this ensures the phthalic anhydride reagent melts) until stirring had stopped, because of an increase in viscosity of the crude reaction mixture, or for the desired reaction time period. After this reaction time, the stirring was stopped, the vial was removed from heating, and an aliquot was taken of the crude product mixture and analyzed *via* ^{1}H NMR spectroscopy to determine the conversion. As a standard protocol of purification employed in the literature [1–5], to purify and isolate the polyester product (particularly to remove any catalyst residue and any unreacted substrate), dichloromethane was added (~3 mL) to form a concentrated solution, followed by acidified methanol (2M, 35 mL) to precipitate a copolymer solid. This was isolated *via* centrifugation (RPM = 3500, 15 minutes). The polymer solid would collect at the bottom of the centrifuge tube, the solvent could be decanted and discarded away, and the polymer redissolved in dichloromethane to transfer all product to another vial. The solvents were removed *in–vacuo* on a rotary evaporator and dried in a vacuum oven at 35 °C overnight.

General procedure for the organo-catalyzed ROCOP in argon

To a 20 mL vial, the chosen epoxide was added, followed by anhydride, commercial *bis*(triphenylphosphoranylidene)iminium chloride, and magnetic stirring bar; for amounts, see the relevant table. The vial was sealed with a septa cap and flushed with argon for ten minutes using a needle. Anhydrous toluene was added (3.0 mL) to the vial through the septa cap. The vial was placed in a preheated heating block. The crude reaction mixture in the vial was stirred at 110 °C. After the reaction time, the stirring was stopped, the vial was removed from heating and opened to air, and an aliquot was taken of the crude product mixture and analyzed *via* ^{1}H NMR spectroscopy to determine the conversion. As a standard protocol of purification employed in the literature [1–5], to purify and isolate the polyester product (particularly to remove any catalyst residue and any unreacted substrate), dichloromethane was added (~3 mL) to form a solution, followed by acidified methanol (2M, 35 mL) to precipitate a copolymer solid. This was isolated *via* centrifugation (RPM = 3500, 15 minutes). The polymer solid would collect at the bottom of the centrifuge tube, the solvent could be decanted and discarded away, and the polymer redissolved in dichloromethane to transfer all product to another vial. The solvents were removed *in–vacuo* on a rotary evaporator and dried in a vacuum oven at 35 °C overnight.

General PU film synthesis

The chosen polyester, polyol (0.200 g or 0.700 g, 1 eq. [OH]), the Sn(II)–octanoate (0.8 mol% [OH]), and dry THF (1.0 mL for the 0.200 g scale or 3.0 mL for the 0.700 g scale) were added to a 20 mL vial (stored in a 105 °C oven to ensure dryness) with a septa cap. The vial was flushed with nitrogen for ~5 mins, placed on a vortex, and stirred at 40 °C for 10 minutes to ensure all components had dissolved in the solvent. Hexamethylene diisocyanate (HDI) ([NCO]/[OH] = 1.05 eq., 11.9 mmol/g [NCO]) was added to another vial (stored in a 105 °C oven to ensure dryness). Dry THF (1.0 mL for the 0.200 g scale or 2.5 mL for the 0.700 g scale) was added to the HDI, and the colorless solution was added to the polyester polyol reaction vial. This vial was briefly degassed (~30 seconds) and vortexed for 10 minutes. The solution (the color dependent on the polyol, ranging from colorless to pale yellow for DCAGE-derived

polyesters to amber for LHOGE-derived polyesters) was then stirred at 40 °C (RPM = 650) until sufficient cloudiness was observed but the solution not too viscous (gel point). The cloudy solution was poured into a mold. The mold was left overnight in the fumehood surrounded by a metal tray with punctured holes to ensure slow evaporation of the solvent and further reaction / cross–linking. The mold was placed in a 105 °C oven for 2 hours and vacuum oven at room temperature overnight to produce the isolated, dry PU film.

S2. Supplementary results and discussion from the main text (Tables and Figures)

S2.1. Development of a catalytic ROCOP system for lignin model epoxide compounds and phthalic anhydride (*Tables S1–3* and *Figure S1–3*)

Table S1. Initial investigations into the ROCOP of PA / PGE, in neat or solution conditions in air, using [Cr] catalyst and [PPNCl] co–catalyst[a].

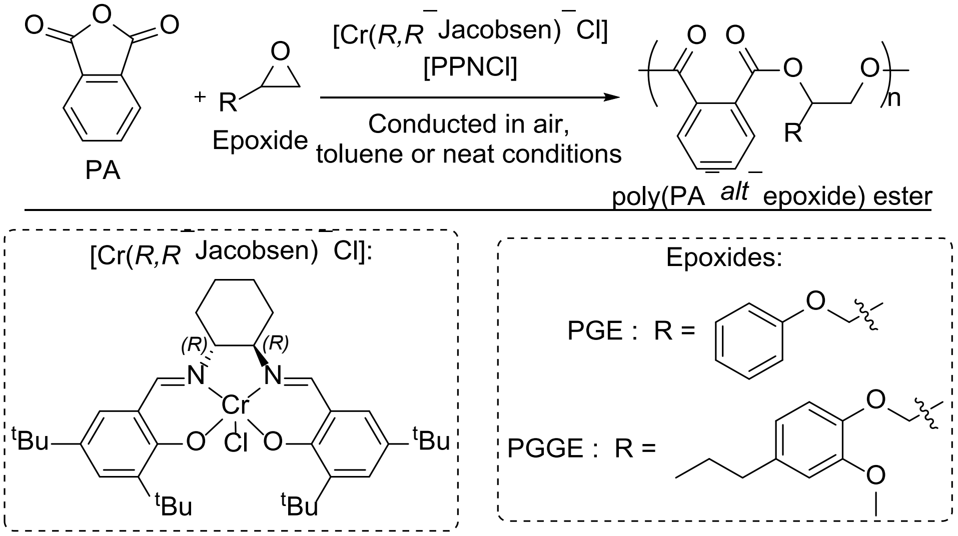

Entry	[PA]:[PGE]:[Cr]:[PPNCl]	Solvent	Temp.	Time	Conv.[b]	TOF[c]	M_n[d]	Đ[d]
1[e]	[250]:[250]:[1]:[1]	Toluene	110	120	100	125	5700	1.58
2[e]	[250]:[250]:[1]:[0]	Toluene	110	120	0	0	–	–
3[e]	[250]:[250]:[1]:[1]	Toluene	110	30	100	500	5150	1.16
4[e]	[250]:[250]:[1]:[1]	Toluene	90	120	100	125	5500	1.18
5	[250]:[250]:[1]:[1]	Toluene	60	120	39	49	2850	1.26
6[e,f]	[500]:[500]:[1]:[1]	Toluene	110	120	100	250	6800	1.16
7[e,g]	[1000]:[1000]:[1]:[1]	Toluene	110	120	65	325	7500	1.15
8[e]	[250]:[250]:[1]:[1]	Neat	110	8	93	1744	3900	1.17
9[e,f]	[500]:[500]:[1]:[1]	Neat	110	18	85	1417	4650	1.15
10[e,g]	[1000]:[1000]:[1]:[1]	Neat	110	54	74	822	5100	1.15

[a] Conditions in air: Commercial PA (0.3703 g, 2.5 mmol), commercial PGE (0.339 mL, 2.5 mmol), commercial [Cr] catalyst (0.4 mol%, 0.01 mmol), commercial [PPNCl] co–catalyst (0.4 mol%, 0.01 mmol), standard toluene (1.0 mL) or neat. [b] Determined by ^{1}H NMR spectroscopy ($CDCl_3$) by integrating the methylene resonance of PGE (δ 3.22–3.56 ppm) and the opened epoxide, methylene resonance in the poly(PA–*co*–PGE) (δ 5.42–5.84 ppm). [c] TOF = [(Conv. (%) / 100) x (100 / mol%)] / time (h)]. [d] Determined *via* GPC (refractive index analysis) in THF solvent. [e] Residual water observed by ^{1}H NMR spectroscopy. [f] Conditions in air: commercial PA (0.7406 g, 5 mmol), commercial PGE (0.677 mL, 5 mmol), [Cr] catalyst (0.2 mol%, 0.01 mmol), [PPNCl] co–catalyst (0.2 mol%, 0.01 mmol), standard toluene (1.0 mL) or neat. [g] Conditions in air: commercial PA (1.4812 g, 10 mmol), commercial PGE (1.354 mL, 10 mmol), [Cr] catalyst (0.1 mol%, 0.01 mmol), [PPNCl] co–catalyst (0.1 mol%, 0.01 mmol), standard toluene (1.0 mL) or neat.

Table S2. Initial investigations into the ROCOP of PA / PGGE, in neat or solution conditions in air, using [Cr] catalyst and [PPNCl] co–catalyst.

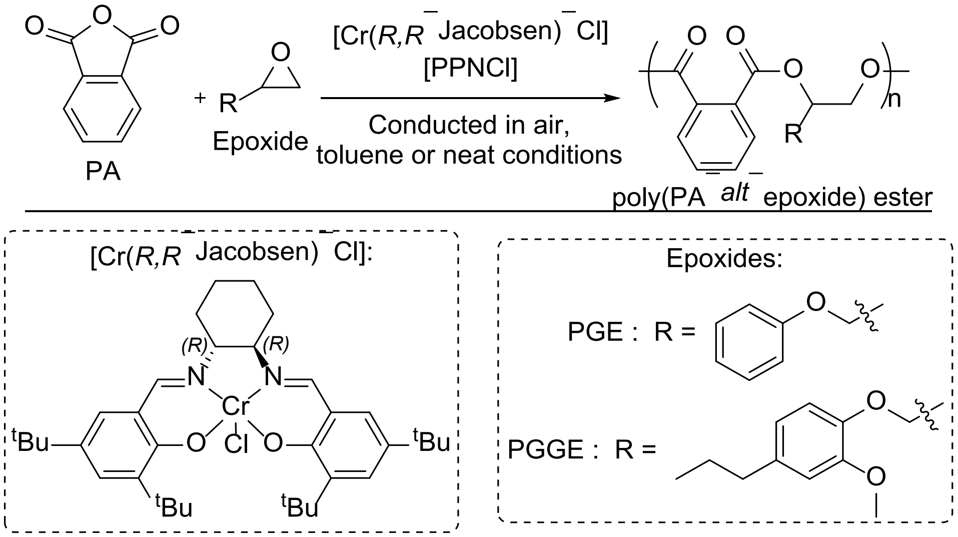

Entry	[PA]:[PGGE]:[Cr]:[PPNCl]	Solvent or neat	Time (mins.)	Conv.[c] (%)	TOF[d] (h^{-1})	M_n[e] (Da)	Đ[e]
1[a]	[250]:[250]:[1]:[1]	Neat	16	100	937	2900	1.24
2[b]	[1000]:[1000]:[1]:[1]	Toluene	240	82	205	3350	1.26

[a] Neat conditions in air: 110 °C, grounded and vacuum oven dried, commercial PA (0.3703 g, 2.5 mmol), synthesized PGGE (0.5557 g, 2.5 mmol), commercial [Cr] catalyst (0.4 mol%, 0.01 mmol), commercial [PPNCl] co–catalyst (0.4 mol%, 0.01 mmol). [b] Solution conditions in air: 110 °C, grounded and vacuum oven dried, commercial PA (0.3703 g, 2.5 mmol), synthesized PGGE (0.5557 g, 2.5 mmol), [Cr] catalyst (0.1 mol%, 0.0025 mmol), [PPNCl] co–catalyst (0.1 mol%, 0.0025 mmol). [c] Determined by ^{1}H NMR spectroscopy ($CDCl_3$) by integrating the resonances of PA (δ 7.86–8.16 ppm) and the aromatic phenylene resonances in the poly(PA–*co*–PGGE) (δ 7.34–7.86 ppm). [d] TOF = [(Conv. (%) / 100) x (100 / mol%)] / time (h)]. [e] Determined *via* GPC (refractive index analysis) in THF solvent.

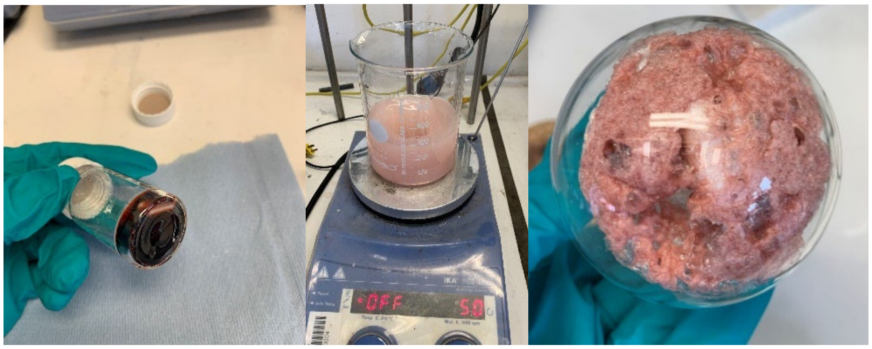

Figure S1. Pictures for *Table S1*, entry 7: *Left*) The deep dark red, crude ROCOP mixture after the reaction time. *Middle*) Despite extensive purification efforts, the pale pink color remained from the [Cr] catalyst residue; here is an example where the color remains despite adding and stirring a concentrated dichloromethane solution of the crude mixture (3.0 mL) into a large excess of acidified methanol (100 mL, 2M HCl). *Right*) After six attempts of repeated precipitations of the polymer solid, the final non-white, pale pink product is shown.

Table S3. PA / PGE, PGGE, and EGE ROCOP in neat or solution conditions in air using [PPNCl] organic catalyst.

Entry	Epoxide	[PA]:[PGE]:[PPNCl]	Solvent or neat	Time (h)	Conv.[e] (%)	TOF[f] (h^{-1})	M_n[g] (Da)	Đ[g]
1[a]	PGE	[500]:[500]:[0]	Neat	2	0	–	–	–
2[b]	PGE	[500]:[500]:[1]	Neat	0.5	0	0	–	–
3[b]	PGE	[500]:[500]:[1]	Neat	2	77	193	4250	1.12
4[b]	PGGE	[500]:[500]:[1]	Neat	2.5	85	170	3200	1.23
5[b]	EGE	[500]:[500]:[1]	Neat	2	80	200	1950	1.34
6[c]	PGE	[1000]:[1000]:[1]	Neat	4	74	185	4300	1.14
7[d]	PGE	[2000]:[2000]:[1]	Neat	8	78	195	5000	1.19
8[a]	PGE	[500]:[500]:[1]	Toluene (1.0 mL)	24	>99	21	3950	1.29
9[c]	PGE	[1000]:[1000]:[1]	Toluene (2.0 mL)	24	>99	42	5250	1.11
10[d]	PGE	[2000]:[2000]:[1]	Toluene (3.0 mL)	24	90	75	5700	1.12

[a] Conditions in air: 110 °C, grounded and vacuum oven dried, commercial PA (0.370 g, 2.5 mmol), commercial PGE (0.339 mL, 2.5 mmol), standard toluene or neat. [b] Commercial PGE (0.339 mL, 2.5 mmol) or synthesized PGGE (0.556 g, 2.5 mmol) or synthesized EGE (0.551 g, 2.5 mmol), [PPNCl] catalyst (0.2 mol%, 0.005 mmol) added. [c] Conditions in air: 110 °C, grounded and vacuum oven dried, commercial PA (0.741 g, 5 mmol), commercial PGE (0.667 mL, 5 mmol), [PPNCl] catalyst (0.1 mol%, 0.005 mmol), standard toluene or neat. [d] Conditions in air: 110 °C, grounded and vacuum oven dried, commercial PA (1.481 g, 10 mmol), commercial PGE (1.354 mL, 10 mmol), [PPNCl] catalyst (0.05 mol%, 0.005 mmol), standard toluene or neat. [e] Determined by ^1H NMR spectroscopy ($CDCl_3$). [f] TOF = [(Conv. (%) / 100) x (100 / mol%)] / time (h)]. [g] Determined *via* GPC (refractive index analysis) in THF solvent.

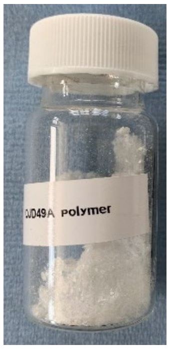

Figure S2. Picture of the purified and isolated white polymeric product, with no metal residue, for entry 2 of *Table S3* from [PPNCl]-mediated PA / PGE ROCOP.

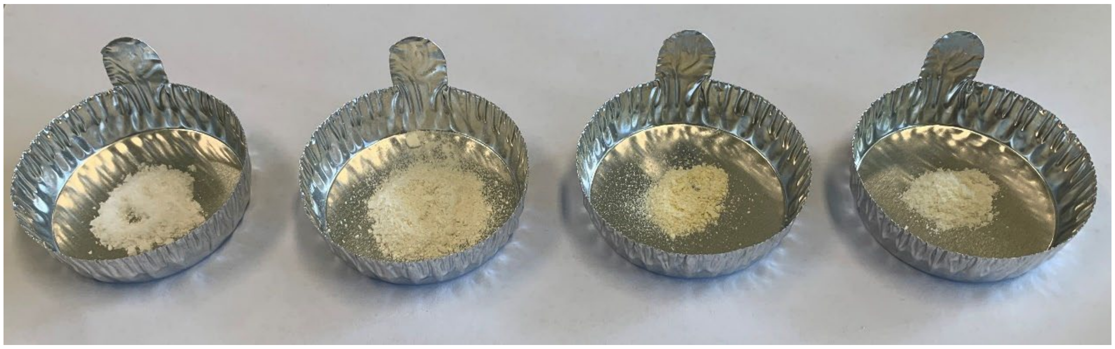

Figure S3. Pictures of the purified, dried, and isolated near-white polymeric products from the [PPNCl]-catalyzed ROCOP of PA with PGE, PGGE, EGE, and DCAGE substrates (*from left to right*), with no metal residue, for entries 1, 2, 3 and 4 of *Table 2*.

S2.2. Exploring organo–catalyzed PA / DCAGE ROCOP with views to further material applications (*Figures S4–5*)

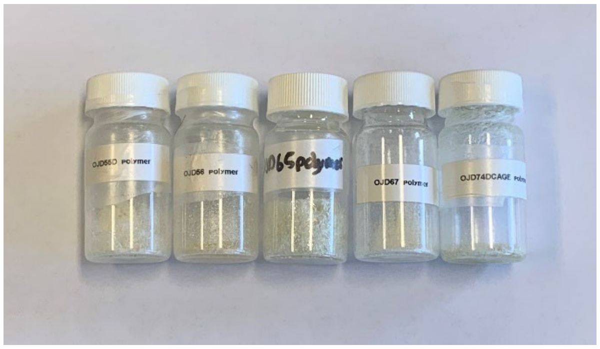

Figure S4. Pictures of purified, dried, and isolated near-white polymeric products from the [PPNCl] catalyzed ROCOP of PA and DCAGE substrates with no metal residue (*from left to right*: entries 1, 2, 3, 4 and 5 of *Table 3*).

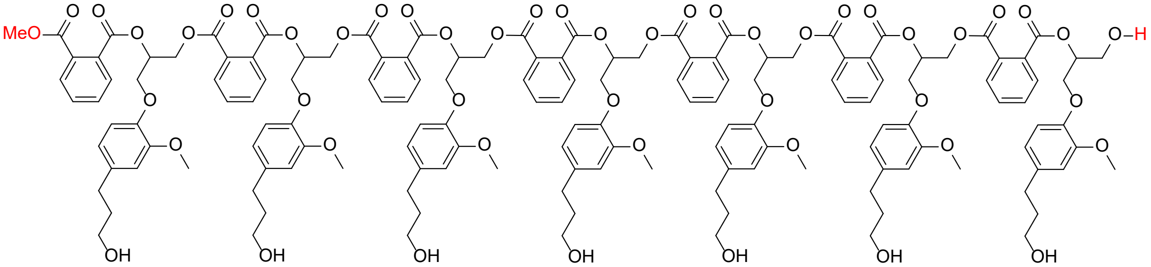

Figure S5. Initially proposed possible structure for the linear, thermoplastic, alternating, and functional poly(PA–*co*–DCAGE) polyester formed with seven aligned aliphatic arms for entry 2 of *Table 2*. A range of end groups are possible with the method of purification; the standard protocol was employed from the literature, one of the most likely is shown here in red.[1–5] See below in the ***2.4. Mechanistic considerations: Why the increase in rate with the presence of [OH] groups, the role of the [OH] groups and an alternative ROCOP mechanism proposed inherent for lignin*** section **for the updated proposed structures**.

S2.3. Blank control ROCOP reactions, chain–transfer reactions, self–catalysis/promoted ROCOP and [OH] species (*Tables S4–6*)

Table S4. Blank or [PPNCl] organo–catalyzed PA / DCAGE, PGGE and EGE ROCOP in neat conditions and air.

Entry	Epoxide	Added [Catalyst]	Time (mins.)	Conv.[f] (%)	M_n[g] (g.mol^{-1})	Đ[g]
1[a]	DCAGE	None	15	88	1250	1.80
2[b]	DCAGE	None	10	97	1950	1.95
3[c]	DCAGE	PPNCl	6	88	2650	1.78
4[c]	DCAGE	PPNCl	10	>99	2800	1.78
5[d]	PGGE	None	60	91	2150	1.22
6[e]	PGGE	PPNCl	35	86	2000	1.21
7[d]	EGE	None	120	47	1300	1.26
8[e]	EGE	PPNCl	120	80	1950	1.34

Conditions in air: 110 °C and neat. [a] Grounded and vacuum oven dried, commercial PA (0.0632 g, 0.427 mmol), synthesized DCAGE (0.1018, 0.427 mmol). [b] Grounded and vacuum oven dried, commercial PA (0.3703 g, 2.5 mmol), synthesized DCAGE (0.5957 g, 2.5 mmol). [c] From *Table 2*, entries 1 and 2: Grounded and vacuum oven dried, commercial PA (0.3703 g, 2.5 mmol), synthesized DCAGE (0.5957 g, 2.5 mmol), [PPNCl] (0.2 mol%, 0.005 mmol). [d] Grounded and vacuum oven dried, commercial PA (0.3703 g, 2.5 mmol), synthesized PGGE (0.5557 g, 2.5 mmol) or synthesized EGE (0.5507 g, 2.5 mmol). [e] Grounded and vacuum oven dried, commercial PA (0.3703 g, 2.5 mmol), synthesized PGGE (0.5557 g, 2.5 mmol) or synthesized EGE (0.5507 g, 2.5 mmol), [PPNCl] catalyst (0.2 mol%, 0.005 mmol), [PA]:[PGGE/EGE]:[PPNCl] = [500]:[500]:[1]. [f] Determined by ^{1}H NMR spectroscopy ($CDCl_3$) by integrating the resonances of PA (δ 7.86–8.16 ppm) and the aromatic phenylene resonances in the poly(PA–*co*–DCAGE/PGGE/EGE) (δ 7.34–7.86 ppm). [g] Determined *via* GPC (refractive index analysis) in THF solvent.

Expanding on the discussion from the ***2.3. Blank control ROCOP reactions, chain–transfer reactions, self–catalysis/promoted ROCOP and [OH] species*** section of the main text, it was also found that, after conducting the other blank ROCOP reactions with synthesized PGGE and EGE, the initiation from OH groups for DCAGE may not have been fully derived from the aliphatic OH side chains. Unexpectedly, slower polymerization was still observed without the presence of these moieties and no structural monomer features capable of opening two ringed substrates, for PGGE and EGE (*Table S3*, entries 5 and 7). This was attributed to traces of [OH] containing impurities from the glycidylation protocol, such as the α–chlorohydrin species; the intermediate before the intramolecular ring closure to form the epoxide *via* dehydrohalogenation.[72,94] These species would be present in the synthesized DCAGE as well, and thus may have contributed to the accelerated reaction times with the many OH side chains. Despite this, in all cases for all synthesized lignin model epoxides, the ROCOP was accelerated with the addition of [PPNCl].

The PGE employed, for the earlier blank PA / PGE ROCOP (*Table S3*, entry 1), was commercially sourced so may explain the zero–reactivity observed. Upon synthesizing the PGE, using the same glycidylation protocol, polymerization was then observed for the blank PA / synthesized PGE ROCOP attributed to be coming from glycidylation impurities as there were no [OH] side chains present (*Table S4*). These glycidylation impurities are challenging to quantify and likely only present at very low concentrations. The different species could not be distinctly identified and quantified using ^{1}H NMR spectroscopy or phosphitylation and ^{31}P NMR spectroscopy. Nonetheless, efforts were not focused on these [OH] impurity species as they did not affect the main goal of this study for lignin material synthesis, however, they were an important consideration and demonstrated how even small quantities of an organic compound, in this case a synthetic impurity, with [OH] content can behave as possible catalysts for ROCOP.[6] With molecular weight dependent on the total [OH], this may contribute for the decreasing M_n values upon increasing the scale of polymerization in *Table 2*. Overall, all these findings with lignin model compounds were important to consider before the application of LHOGE to ROCOP; with DCA, PG, and aliphatic OH side chains being prominent in LHO, the same glycidylation protocol being employed for the LHO resulting in the same trace impurities and the LHO biomass containing a mixture of many [OH] containing monomer, dimer and oligomer species.[7,8]

Table S5. The comparison between commercial and synthesized PGE in blank PA ROCOP in neat conditions.

Entry	[PGE] source	[PA]:[PGE]:[PPNCl]	Time (h)	Conv.[a] (%)	M_n[b] (g.mol^{-1})	Đ[b]
1	Commercial	[1]:[1]:[0]	2	0	–	–
2	Synthesized	[1]:[1]:[0]	2	58	1900	1.19
3	Commercial	[1]:[1]:[0]	4.5	9	–	–
4	Synthesized	[1]:[1]:[0]	4.5	76	1950	1.47

Conditions in air: 110 °C and neat. Grounded and vacuum oven dried, commercial PA (0.3703 g, 2.5 mmol), commercial or synthesized PGE (0.339 mL, 2.5 mmol), no [PPNCl] added. [a] Determined by ^{1}H NMR spectroscopy ($CDCl_3$) by integrating the resonances of PA (δ 7.86–8.16 ppm) and the aromatic phenylene resonances in the poly(PA–*co*–PGE) (δ 7.34–7.86 ppm). [b] Determined *via* GPC (refractive index analysis) in THF solvent.

Table S6. The organocatalyzed ROCOP of different amounts of synthesized DCAGE in commercial PGE with no [PPNCl] added in neat conditions.

	Synthesized DCAGE spiked into commercial PGE (mol% of [DCAGE])								
	0%	0.1%	0.2%	1%	2.5%	5%	10%	25%	100%[h]
[OH arms] (mol%)[a]	0	0.1	0.2	1	2.5	5	10	25	100
$[OH]_{theo}$[b] (mmol/g)	0	0.0034	0.0067	0.033	0.083	0.17	0.33	0.78	2.6
$[OH]_{exp}$[c] (mmol/g)	n.d.	n.d.	n.d.	n.d.	n.d.	0.11	0.25	0.80	2.20
[DCAGE][d] (wt%)	0	0.080	0.16	0.80	2.0	3.9	7.8	19	62
[OH group on arm][e] (wt%)	0	0.0057	0.011	0.057	0.14	0.28	0.55	1.3	4.4
[OH propanol arm][e] (wt%)	0	0.020	0.040	0.20	0.49	0.97	1.9	4.6	15
Total monomer (g)	0.746	0.746	0.746	0.748	0.751	0.757	0.768	0.801	0.966
Time	24 h	28 h	22 h	312 mins	130 mins	70 mins	35 mins	15 mins	10 mins
Conv.[f] (%)	36	69	77	74	79	79	83	87	97
M_n[g] (g.mol^{-1})	–	3800	4250	3750	4000	3500	2800	2250	1950
Đ[g]	–	1.10	1.09	1.10	1.15	1.20	1.26	1.18	1.95

Conditions in air: 110 °C and neat. Grounded and vacuum oven dried, commercial PA (0.3703 g, 2.5 mmol, 100 eq.), commercial PGE and synthesized DCAGE (overall 2.5 mmol, 100 eq.), no [PPNCl] added. Abbreviations: n.d. = not determined, *theo* = theoretical, *exp* = experimental or experimentally measured. [a] mmol or mol% of [DCAGE] = mmol or mol% of [OH arms]. [b] [OH] $(mmol/g)_{theoretical}$ = [DCAGE] (mmol) / total monomer (g). [c] [OH] $(mmol/g)_{experimental}$, determined *via* phosphitylation and quantitative ^{31}P NMR spectroscopy. This was achieved by taking an aliquot of the crude ROCOP product mixture as soon as the reaction was complete. This was not done before the reaction because of concerns with the cyclic anhydride and epoxide opening during the phosphitylation NMR reaction. The value was calculated by subtracting the integral of the observed carboxylic acid OH resonance signals from the integral of the aliphatic OH resonance signals. Theoretically, the carboxylic acid resonance signals reside from the polymer end groups and, thus, are not present before the ROCOP reaction and should not be used in the calculation of mmol [OH]/g. In addition, as there are a statistically equal distribution of polymer end groups, it was assumed there was an equal amount and integral of aliphatic resonance signals residing from the polymer end groups, and hence the subtraction. [d] Mass of [DCAGE] / total monomer (g). [e] (mol of [DCAGE] x M_w of the OH group or propanol arm) / total monomer (g). [f] Determined by ^{1}H NMR spectroscopy ($CDCl_3$) by integrating the resonances of PA (δ 7.86–8.16 ppm) and the aromatic phenylene resonances in the poly(PA–*co*–PGE) (δ 7.34–7.86 ppm). [g] Determined *via* GPC (refractive index analysis) in THF solvent. [h] From *Table S4*, entry 2.

S2.4. Mechanistic considerations: Why the increase in rate with the presence of [OH] groups, the role of the [OH] groups and an alternative ROCOP mechanism proposed inherent for lignin (*Figures S6–14* and *Table S7*)

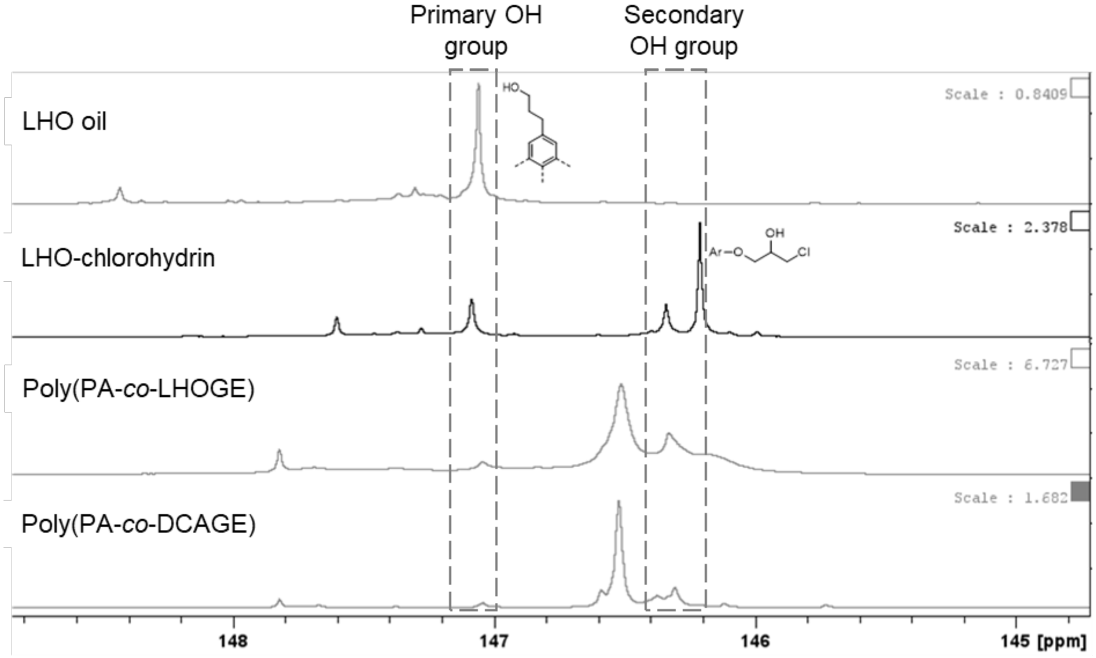

Figure S6. Stacked ^{31}P NMR spectra of LHO oil, the LHO-chlorohydrin (intermediate during the epoxidation protocol), poly(PA–*co*–LHOGE) and poly(PA–*co*–DCAGE) polyester samples focused in on the aliphatic OH region. The two different alcohol environments are highlighted.

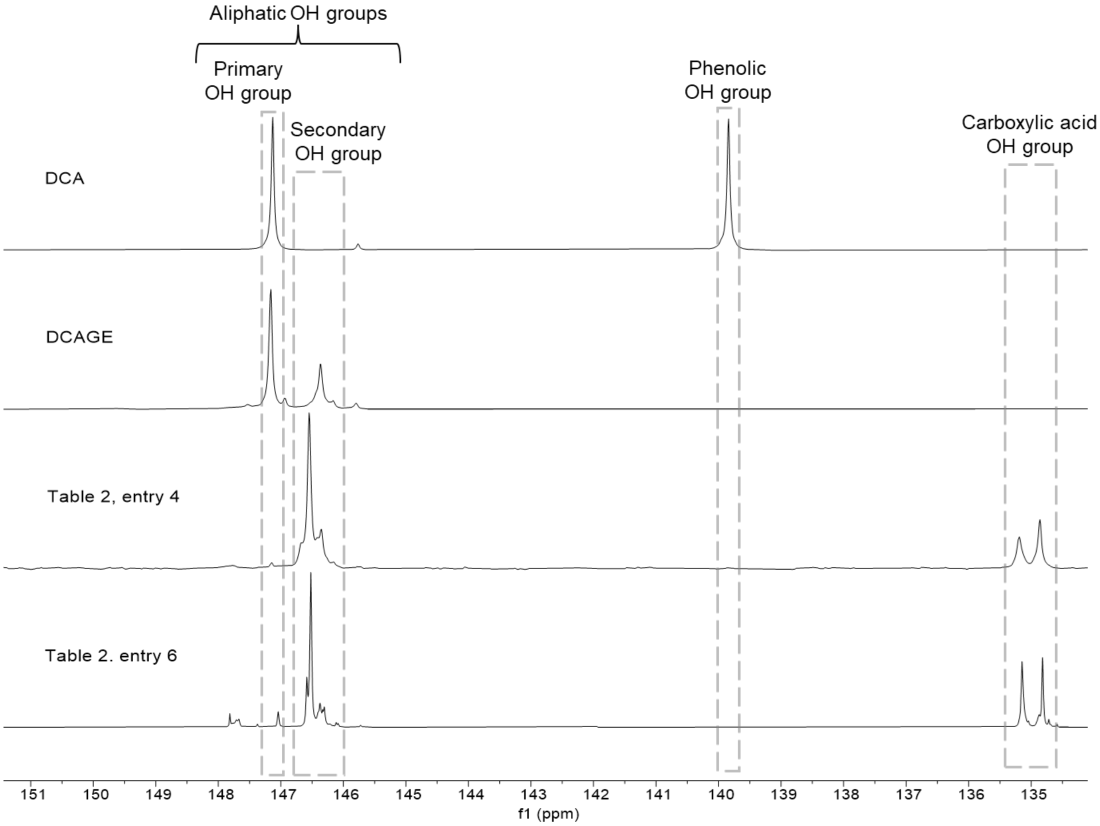

Figure S7. Stacked ^{31}P NMR spectra of DCA, DCAGE and poly(PA–*co*–DCAGE) polyester samples.

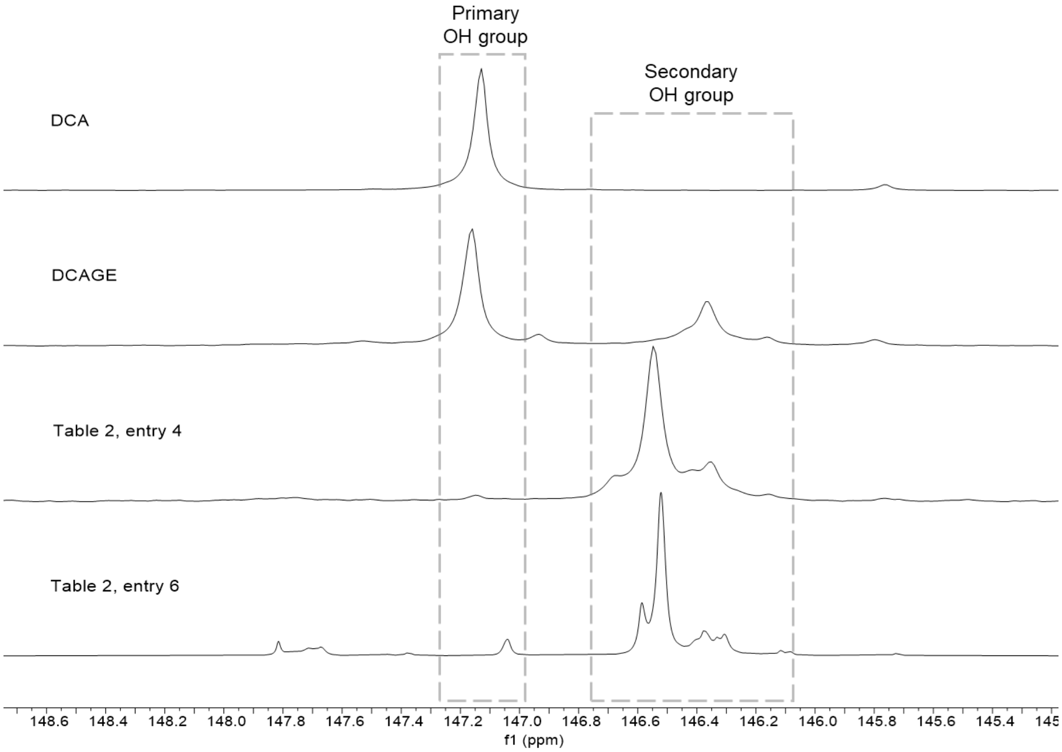

Figure S8. Stacked ^{31}P NMR spectra of DCA, DCAGE and poly(PA–*co*–DCAGE) polyester samples focused in on the aliphatic OH region.

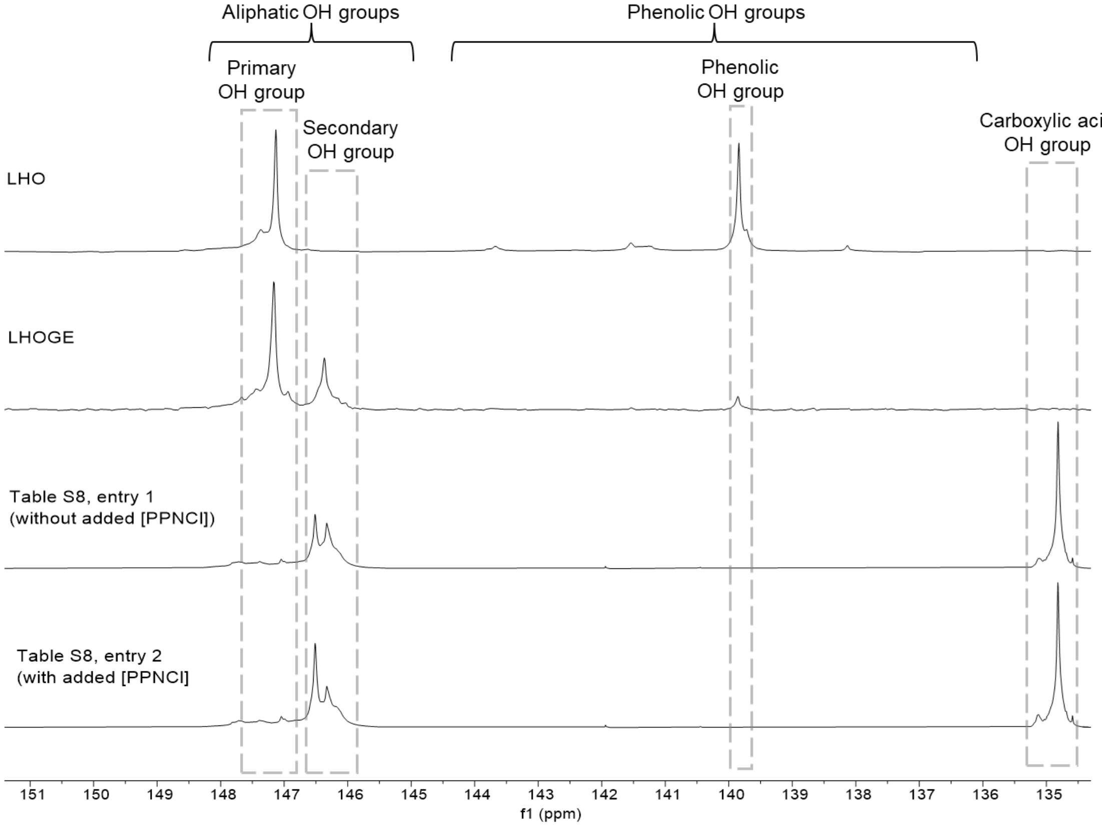

Figure S9. Stacked ^{31}P NMR spectra of LHO, LHOGE and poly(PA–*co*–LHOGE) polyester samples (both with and without [PPNCl] added to the ROCOP).

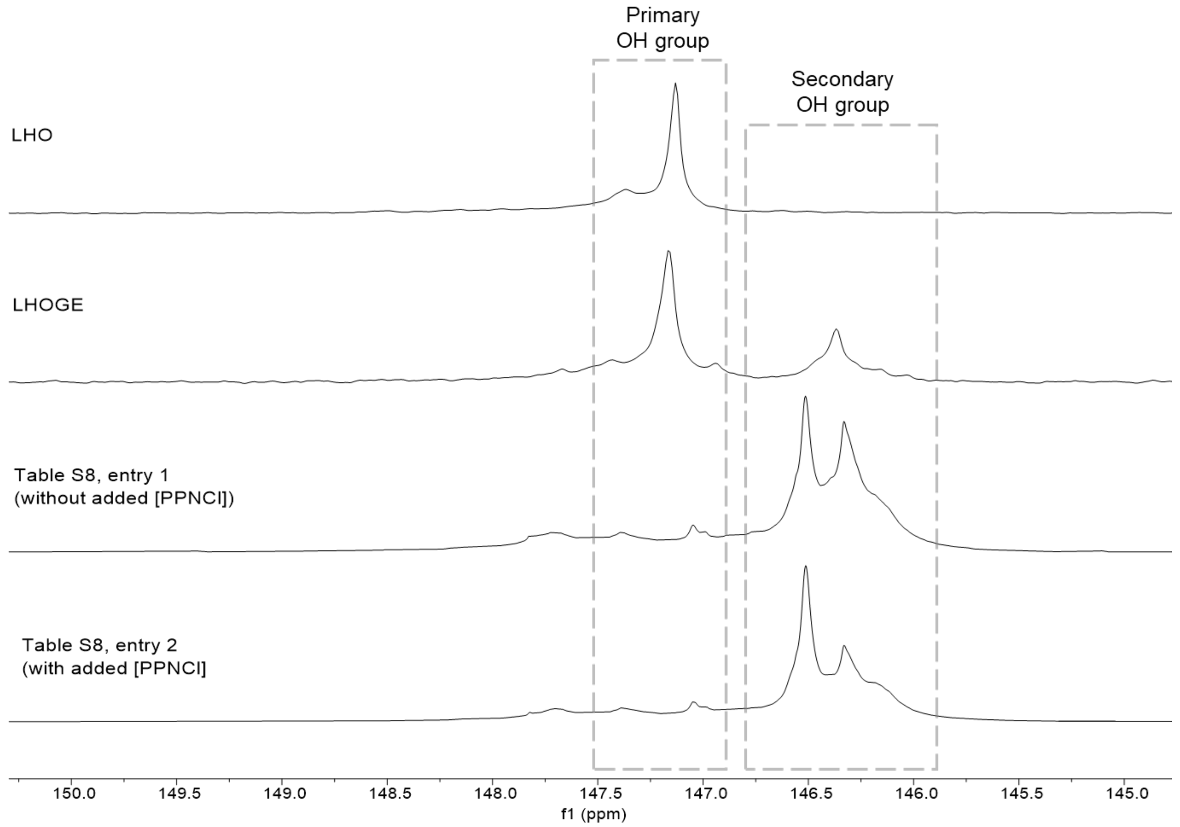

Figure S10. Stacked ^{31}P NMR spectra of LHO, LHOGE and poly(PA–*co*–LHOGE) polyester samples (both with and without [PPNCl] added to the ROCOP) focused in on the aliphatic OH region.

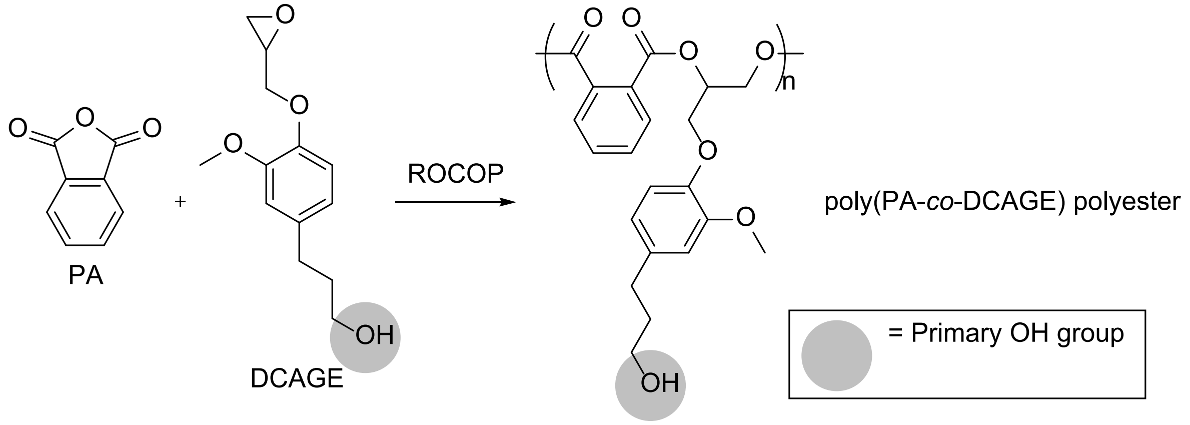

Figure S11. Pathway A: traditional ROCOP pathway to polyester product for PA and DCAGE.

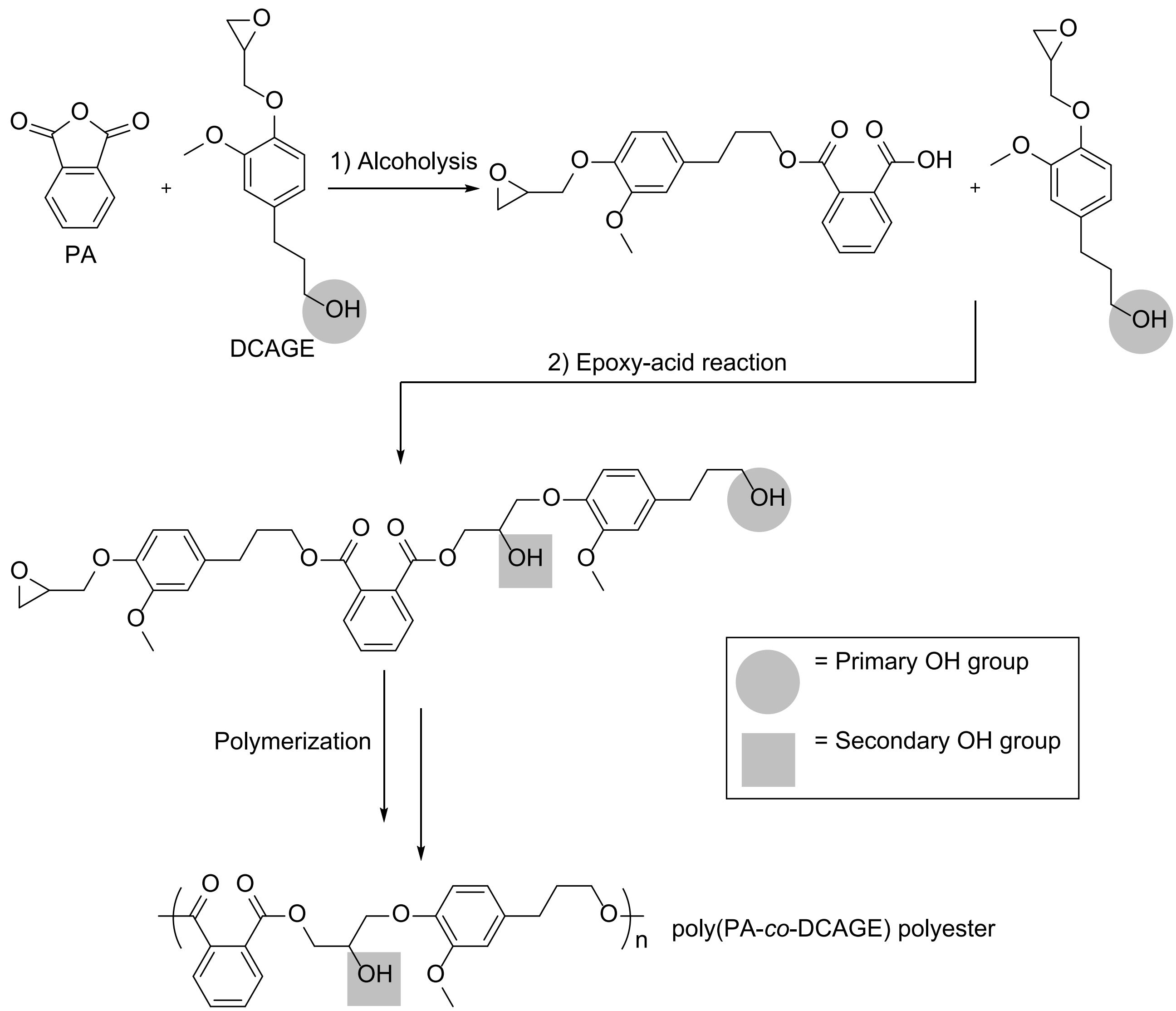

Figure S12. Pathway B: alcoholysis and epoxy–acid pathway to polyester product for PA and DCAGE.

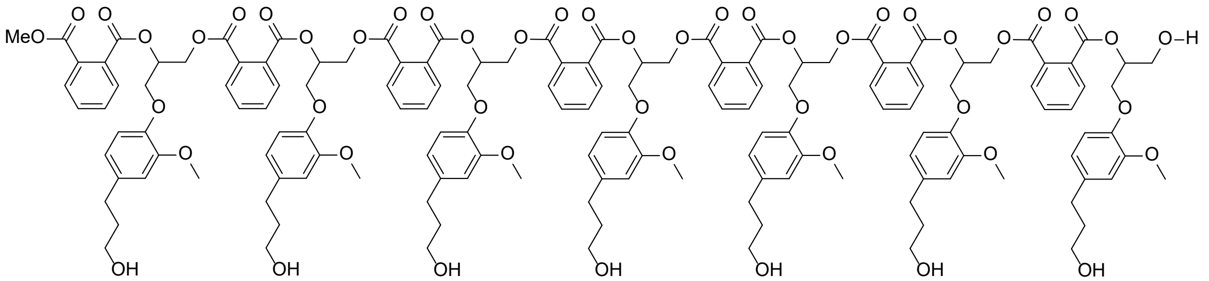

Figure S13. Possible poly(PA-co-DCAGE) polyester structure *via* Pathway A (with possible end groups).

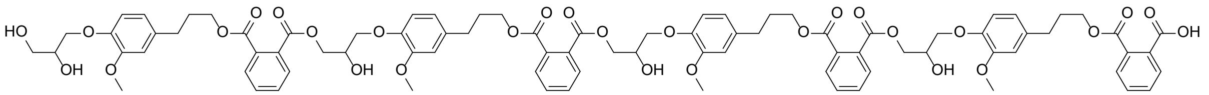

Figure S14. Possible poly(PA-co-DCAGE) polyester structure *via* Pathway B (with possible end groups).

Table S7. Spiking PA / PGE ROCOP with 3–phenyl–1–propanol without [PPNCl] added catalyst.

Entry	[PA]:[PGE]:[PPNCl]:[3–phenyl–1–propanol]	Time (h)	Conv. (%)[d]	M_n (g.mol^{-1})[e]	Đ[e]
1[a]	[1]:[1]:[0]:[0]	2	0	–	
2[b]	[1]:[1]:[0]:[0]	24	36	–	
3[c]	[1]:[1]:[0]:[1]	0.5	73	n.d.	
4[c]	[1]:[1]:[0]:[1]	18	95	n.d.	
5[c]	[1]:[1]:[0]:[1]	24	95	450	1.15

Conditions in air: 110 °C and neat. Grounded and vacuum oven dried, commercial PA (0.3703 g, 2.5 mmol), commercial PGE (0.339 mL, 2.5 mmol), no [PPNCl] added. [a] From *Table S5*, entry 1. [b] From *Table S6*. [c] 3–phenyl–1–propanol (0.340 mL, 2.5 mmol) added. [d] Determined by ^{1}H NMR spectroscopy ($CDCl_3$) by integrating the resonances of PA (δ 7.86–8.16 ppm) and the aromatic phenylene resonances in the poly(PA–*co*–PGE) (δ 7.34–7.86 ppm). [e] Determined *via* GPC (refractive index analysis) in THF solvent.

For the S***2.8. Further NMR spectroscopic studies for the mechanistic elucidation of the reaction pathway*** section, see below page 28.

S2.5. Organo–catalyzed ROCOP with PA and LHOGE sourced from the RCF of *Pinus radiata* biomass and glycidylation (*Tables S8*)

Table S8. PA / LHOGE ROCOP in neat conditions and air with and without [PPNCl] organic catalyst.

Entry	[PA]:[LHOGE]: [PPNCl]	Time (mins)	PA conv.[e] (%)	M_n[f] (Da)	Đ[f]	Total OH value[g] (mmol/g)	f[h]	T_g[i] (°C)
1[a]	[250]:[250]:[0]	15	38	2000	1.92	3.13	6.3	58.6
2[a]	[250]:[250]:[1]	15	63	2600	1.90	3.21	8.3	59.5
3[a]	[250]:[250]:[1]	20	83	2800	1.93	3.01	8.4	59.3
4[b]	[100]:[100]:[1]	15	78	2750	1.92	2.90	7.8	58.1
5[c]	[500]:[500]:[1]	30	79	2950	1.94	3.03	8.9	61.3
6[d]	[100]:[100]:[1]	20	88	2450	1.93	3.31	8.1	57.4

Conditions in air: 110 °C and neat. [a] Grounded and vacuum oven dried, commercial PA (0.187 g, 1.25 mmol), LHOGE (0.421 g, 1.25 mmol [epoxide]), with and without [PPNCl] catalyst (0.4 mol%, 0.005 mmol). [b] [PPNCl] catalyst (1.0 mol%, 0.0125 mmol). [c] [PPNCl] catalyst (0.2 mol%, 0.0025 mmol). [d] Grounded and vacuum oven dried, commercial PA (1.008 g, 6.81 mmol), LHOGE (2.837 g, 6.81 mmol [epoxide]), [PPNCl] catalyst (1.0 mol%, 0.0681 mmol). [e] Determined by ^{1}H NMR spectroscopy ($CDCl_3$). [f] Determined *via* GPC (refractive index analysis) in THF solvent. [g] Determined *via* phosphitylation and quantitative ^{31}P NMR spectroscopy. [h] Chemical (OH) functionality, f_{OH} = (total OH value mmol/g x M_n of the polymer g.mol^{-1}) / 1000. [i] Determined *via* differential scanning calorimetry.

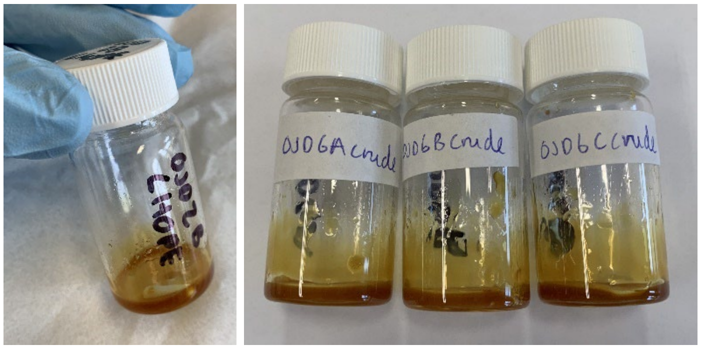

Figure S15. Example pictures of the crude products from the ROCOP of PA and LHOGE substrates for entry 2 (*left*) and entries 3–5 (*right*), *Table S8*.

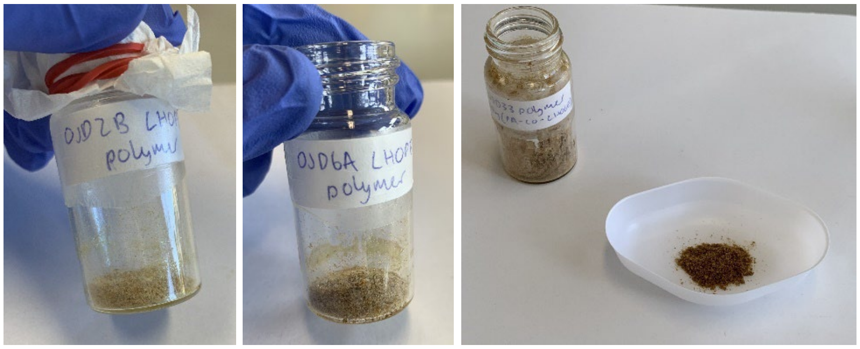

Figure S16. Example pictures of the purified, dried, and isolated products from the ROCOP of PA and LHOGE substrates for entry 2 (*left*), entry 3 (*middle*), and entry 6 (*right*), *Table S8*.

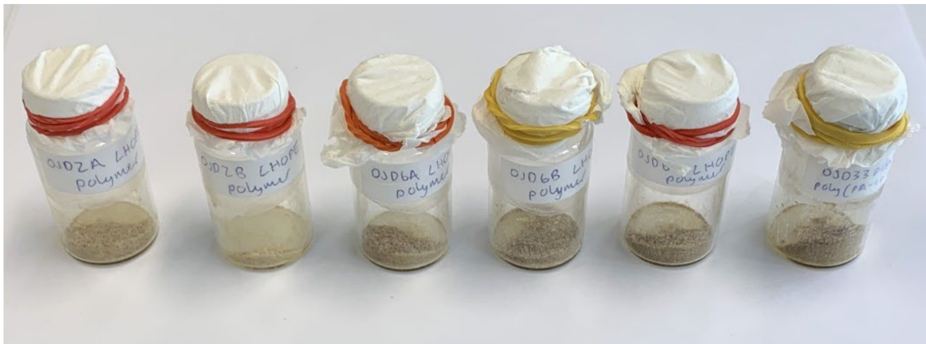

Figure S17. Pictures of all the purified, dried, and isolated products from the ROCOP of PA and LHOGE substrates (*from left to right*: entries 1, 2, 3, 4, 5, and 6 of *Table S8*).

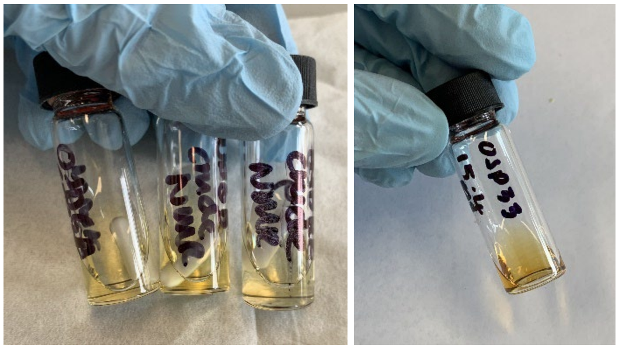

Figure S18. Example pictures of the NMR solutions in $CDCl_3$ for the solubility of the crude products from the ROCOP of PA and LHOGE substrates for entries 3–5 (*left*) and entry 6 (*right*), *Table S8*.

S2.6. Organo–catalyzed ROCOP of DCAGE and LHOGE with succinic anhydride (*Tables S9–10*)

Table S9. SA / DCAGE or LHOGE ROCOP in neat conditions and flushed with nitrogen.

Entry	Epoxide	[SA]:[epoxide]:[PPNCl]	Time (mins)	SA conv.[e] (%)	M_n[f] (Da)	Đ[f]	Total OH value[g] (mmol/g)	f[h]	T_g[i] (°C)
1[a]	DCAGE	[100]:[100]:[1]	15	>99	2350	1.75	2.56	6.0	-1.4
2[a]	DCAGE	[500]:[500]:[1]	15	>99	2450	1.78	2.57	6.3	-2.2
3[b]	DCAGE	[100]:[100]:[1]	15	>99	1100	2.01	3.11	3.4	-12.1
4[c]	LHOGE	[100]:[100]:[0]	30	94	3250	2.39	2.55	8.3	25.8
5[c]	LHOGE	[100]:[100]:[1]	15	98	3550	2.11	3.46	12.3	11.4
6[c]	LHOGE	[500]:[500]:[1]	30	96	4200	1.97	3.05	12.8	21.8
7[d]	LHOGE	[100]:[100]:[1]	20	>99	2750	1.83	3.10	8.5	32.6

Conditions: 110 °C, neat and the reaction vial was briefly flushed with nitrogen just before the polymerization was commenced. [a] Grounded and vacuum oven dried, commercial SA (0.250 g, 2.5 mmol), DCAGE (0.596 g, 2.5 mmol [epoxide]), [PPNCl] catalyst (1.0 mol%, 0.025 mmol for entry 1 or 0.2 mol%, 0.005 mmol for entry 2). [b] Grounded and vacuum oven dried, commercial SA (2.100 g, 21.0 mmol), DCAGE (5.000 g, 21.0 mmol), [PPNCl] catalyst (1.0 mol%, 0.21 mmol). [c] Grounded and vacuum oven dried, commercial SA (0.125 g, 1.25 mmol), LHOGE (0.421 g, 1.25 mmol [epoxide]), with and without [PPNCl] catalyst (1.0 mol%, 0.0125 mmol for entry 5 or 0.2 mol%, 0.0025 mmol for entry 6). [d] Grounded and vacuum oven dried, commercial SA (0.681 g, 6.81 mmol), LHOGE (2.837 g, 6.81 mmol [epoxide]), [PPNCl] catalyst (1.0 mol%, 0.0681 mmol). [e] Determined by ^{1}H NMR spectroscopy ($CDCl_3$). [f] Determined *via* GPC (refractive index analysis) in THF solvent. [g] Determined *via* phosphitylation and quantitative ^{31}P NMR spectroscopy. [h] Chemical (OH) functionality, f_{OH} = (total OH value mmol/g x M_n of the polymer g.mol^{-1}) / 1000. [i] Determined *via* differential scanning calorimetry.

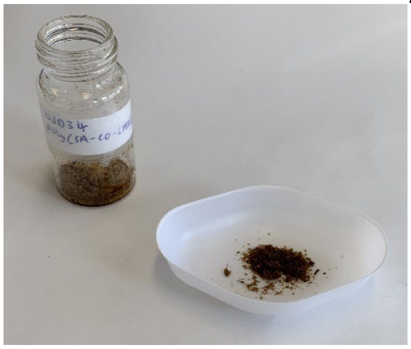

Figure S19. Example picture of the purified, dried, and isolated product from the ROCOP of SA and LHOGE substrates for entry 7, *Table S9*.

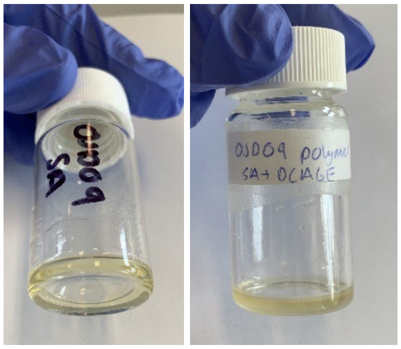

Figure S20. Example pictures of the crude product and the purified, dried, and isolated product from the ROCOP of SA and DCAGE substrates for entry 2, *Table S9*.

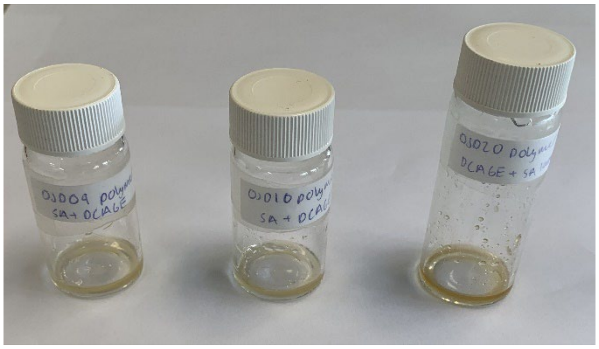

Figure S21. Pictures of all the purified, dried, and isolated products from the ROCOP of SA and DCAGE substrates (*from left to right*: entries 1, 2 and 3 of *Table S8*).

Table S10. SA / DCAGE or LHOGE ROCOP in neat conditions and air.

Entry	Epoxide	[SA]:[epoxide]: [PPNCl]	Time (mins)	SA conv.[c] (%)	M_n[d] (Da)	Đ[d]	Total OH value[e] (mmol/g)	f[f]
1[a]	DCAGE	[100]:[100]:[1]	15	>99	2790	1.83	2.81	7.8
2[b]	LHOGE	[100]:[100]:[1]	15	96	6550	3.91	Did not fully dissolve (crosslinking and thermosetting possibly observed)	–

Conditions: 110 °C, neat and conducted in air. [a] Grounded and vacuum oven dried, commercial SA (0.2502 g, 2.5 mmol), DCAGE (0.5957 g, 2.5 mmol [epoxide]), [PPNCl] catalyst (1.0 mol%, 0.025 mmol). b[f] Grounded and vacuum oven dried, commercial SA (0.1251 g, 1.25 mmol), LHOGE (0.4209 g, 1.25 mmol [epoxide]), with and without [PPNCl] catalyst (1.0 mol%, 0.0125 mmol). [c] Determined by ^{1}H NMR spectroscopy ($CDCl_3$). [d] Determined *via* GPC (refractive index analysis) in THF solvent. [e] Determined *via* phosphitylation and quantitative ^{31}P NMR spectroscopy. [f] Chemical (OH) functionality, f = (total OH value mmol/g x M_n of the polymer g.mol^{-1}) / 1000.

S2.7. Breakdown of the OH contents determined, using phosphitylation and quantitative ^{31}P NMR spectroscopy, of the polyesters (*Table S11*)

Table S11. The OH contents determined, using phosphitylation and quantitative ^{31}P NMR spectroscopy, for the polyesters synthesized in this study; a general reaction sequence for the phosphitylation of lignin is shown with the phosphitylated resonance signals quantified for the different OH environments present in the lignin.

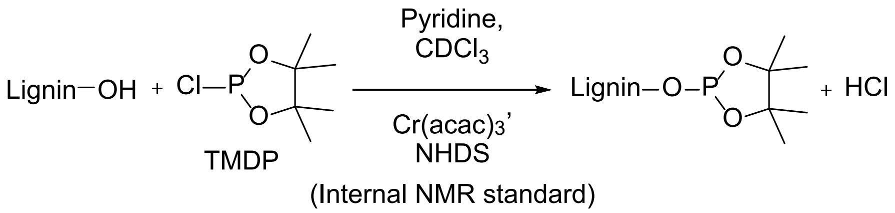

NHDS = endo–N–hydroxy–5–norbornene–2,3–dicarboximide and TMDP = 2–chloro–4,4,5,5–tetramethyl–1,3,2–dioxaphospholane.

Class of polyester	Substrate	Aliphatic OH (mmol/g)	Total phenolic OH (mmol/g)	Carboxylic acid OH (mmol/g)	Total OH value (mmol/g)
Poly(PA–*co*–DCAGE)	Table 2, entry 4	2.07	0	0.99	3.06
	Table 3, entry 1	2.17	0.00	0.94	3.11
	Table 3, entry 2	2.18	0.00	0.95	3.13
	Table 3, entry 3	1.96	0.00	1.11	3.07
	Table 3, entry 4	2.29	0.00	0.93	3.22
	Table 3, entry 5	2.32	0.00	1.14	3.46
	Table 3, entry 6	1.52	0.00	0.82	2.33
Poly(PA–*co*–LHOGE)	Table S8, entry 1	1.88	0.00	1.25	3.13
	Table S8, entry 2	1.98	0.00	1.22	3.21
	Table S8, entry 3	1.92	0.00	1.09	3.01
	Table S8, entry 4	1.91	0.00	0.99	2.90
	Table S8, entry 5	1.86	0.00	1.17	3.03
	Table S8, entry 6	2.32	0.00	1.00	3.31
Poly(SA–*co*–DCAGE)	Table S9, entry 1	2.18	0.00	0.38	2.56
	Table S9, entry 2	2.18	0.00	0.39	2.57
	Table S9, entry 3	2.38	0.00	0.73	3.11

Poly(SA–*co*–LHOGE)	Table S9, entry 4	2.13	0.00	0.42	2.55
	Table S9, entry 5	3.07	0.00	0.39	3.46
	Table S9, entry 6	2.67	0.00	0.38	3.05
	Table S9, entry 7	2.54	0.00	0.55	3.10
Poly(SA–*co*–DCAGE)	Table S10, entry 1	2.14	0.00	0.67	2.81

S2.8. Further NMR spectroscopic studies for the mechanistic elucidation of the reaction pathway (*Figures S22–30* and *Tables S12–13*)

As discussed in the main text under the ***2.4. Mechanistic considerations: Why the increase in rate with the presence of [OH] groups, the role of the [OH] groups and an alternative ROCOP mechanism proposed inherent for lignin*** section, two reaction pathways were proposed (*Figure 3*); Pathway A, where the anhydride reacts solely with the glycidyl ether (classical ROCOP specific), and Pathway B, where the anhydride reacts with both the glycidyl ether and the alcoholic moiety on the side chain. The glycidyl ether of propyl guaiacol (PGGE), also a model compound in hydrogenolysis oil, was used to assist with NMR interpretation. Because PGGE does not contain an alcoholic moiety in the side chain, the reaction mechanism should only proceed via the ROCOP specific Pathway A.

Predictive NMR chemical shifts by ChemDraw® were used to help with peak assignments. Predicted ^{1}H NMR chemical shift assignments of both reaction pathways for PA with DCAGE, and those of pathway A for PA with PGGE are given in *Figure S22*. Predicted ^{13}C NMR chemical shift assignments can be found later in this section (*Figure S26–28*).

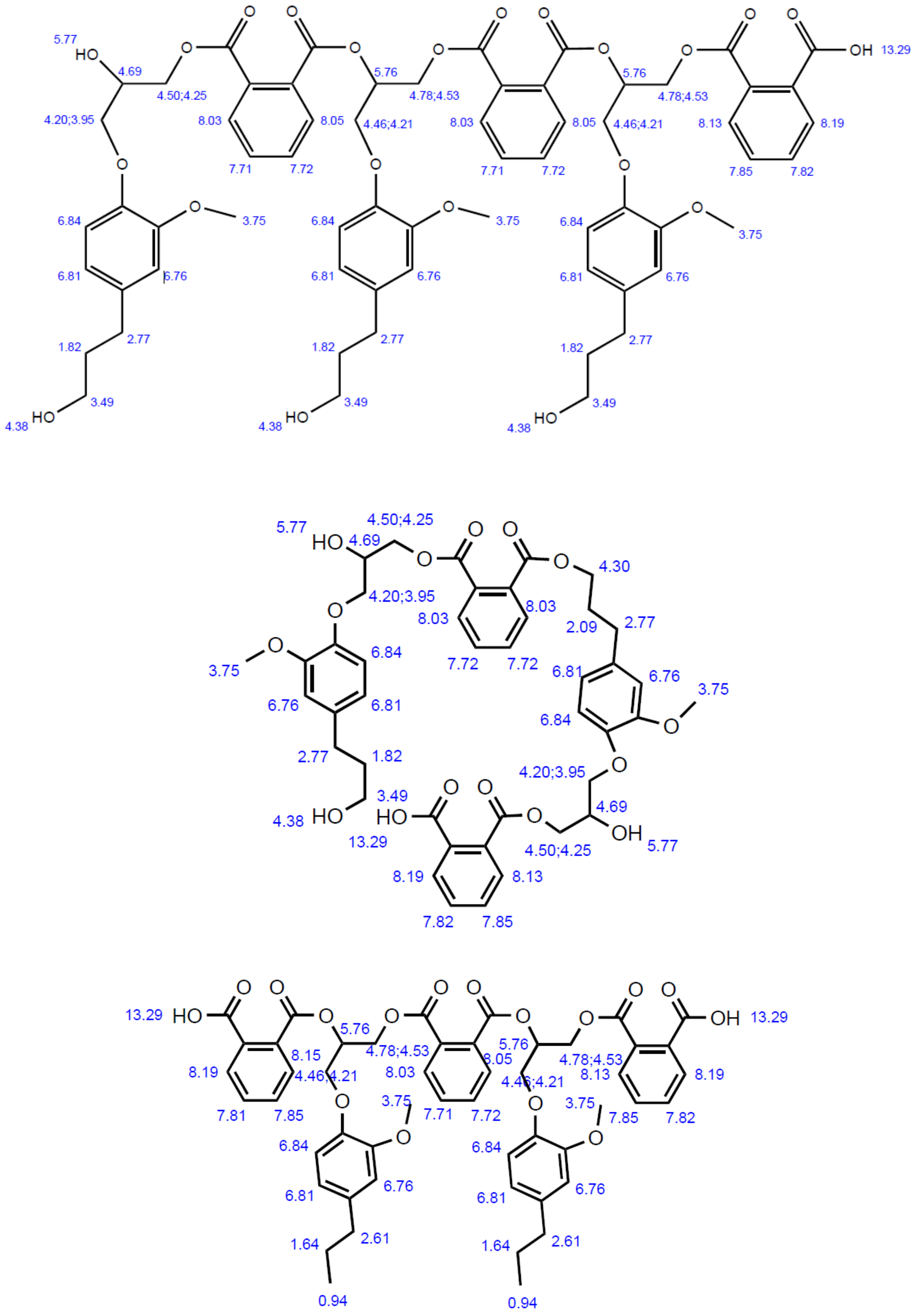

Figure S22. Predicted ^{1}H NMR chemical shift assignments for PA / DCAGE - Pathway A (top) and Pathway B (middle), and for PA / PGGE - Pathway A (bottom).

Standard NMR experiments were performed on selected samples (*Table S12*). In addition, selective Total Correlation Spectroscopy (TOCSY) NMR experiments were performed (*Table S12*) to enhance the NMR signal resolution of the ROCOP products by selectively exciting

specific nuclei within a spin system in a molecule. By focusing on a particular region or group of protons, selective TOCSY helps in distinguishing overlapping signals, providing clearer insights into the structure and connectivity of molecules within a mixture. Note that oxygen atoms and quaternary carbons on the aromatic ring act as barriers and halt further correlations along the chains.

Table S12. Samples analyzed by NMR spectroscopy.

Sample	Polymer	^{1}H	^{13}C	DEPT135	HSQC	HMBC	Sel TOCSY[2]
OJD60A (*Table 1*, entry 1)	PA–*co*–PGE	✓	✓	✓	✓	✓	
OJD60B (*Table 1*, entry 2)	PA–*co*–PGGE	✓	✓	✓	✓	✓	31 (5.69)
OJD60C (*Table 1*, entry 3)	PA–*co*–EGE	✓	✓	✓	✓	✓	
OJD67 (*Table 2*, entry 4)	PA–*co*–DCAGE	✓		Not enough sample			
OJD29 (*Table 2*, entry 6)	PA–*co*–DCAGE	✓	✓	✓	✓	✓	50 (5.68), 61 (2.02), 60 (1.86)
OJD8[1] (*Table S4*, entry 2)	PA–*co*–DCAGE	✓	✓	✓	✓	✓	
OJD33 (*Table S8*, entry 6)	PA–*co*–LHOGE	✓	✓	✓	✓	✓	
OJD2A[1] (*Table S8*, entry 1)	PA–*co*–LHOGE	✓	✓	✓	✓	✓	
OJD20 (*Table S9*, entry 3)	SA–*co*–DCAGE	✓	✓	✓	✓	✓	
OJD34 (*Table S9*, entry 7)	SA–*co*–LHOGE	✓	✓	✓	✓	✓	
OJD11C (*Table S9*, entry 4)	SA–*co*–LHOGE	✓	✓	✓	✓	✓	

[1] without PPNCl. [2] NMR experiment number and chemical shifts used for pulses in ppm.

A unique proton environment in Pathway A is the methine proton at a predicted chemical shift of δ 5.76 ppm. Indeed, a strong signal at δ 5.69 ppm was observed in the ^{1}H NMR spectrum of the PA / PGGE polymer. A selective TOCSY experiment on this polymer was performed by pulsing this signal at δ 5.69 ppm. This gave corresponding signals in the range δ 4.2–4.8 ppm (*Figure S23*) that correlated well to the predicted proton assignments on the adjacent carbons (*Figure S22*), specific to Pathway A.

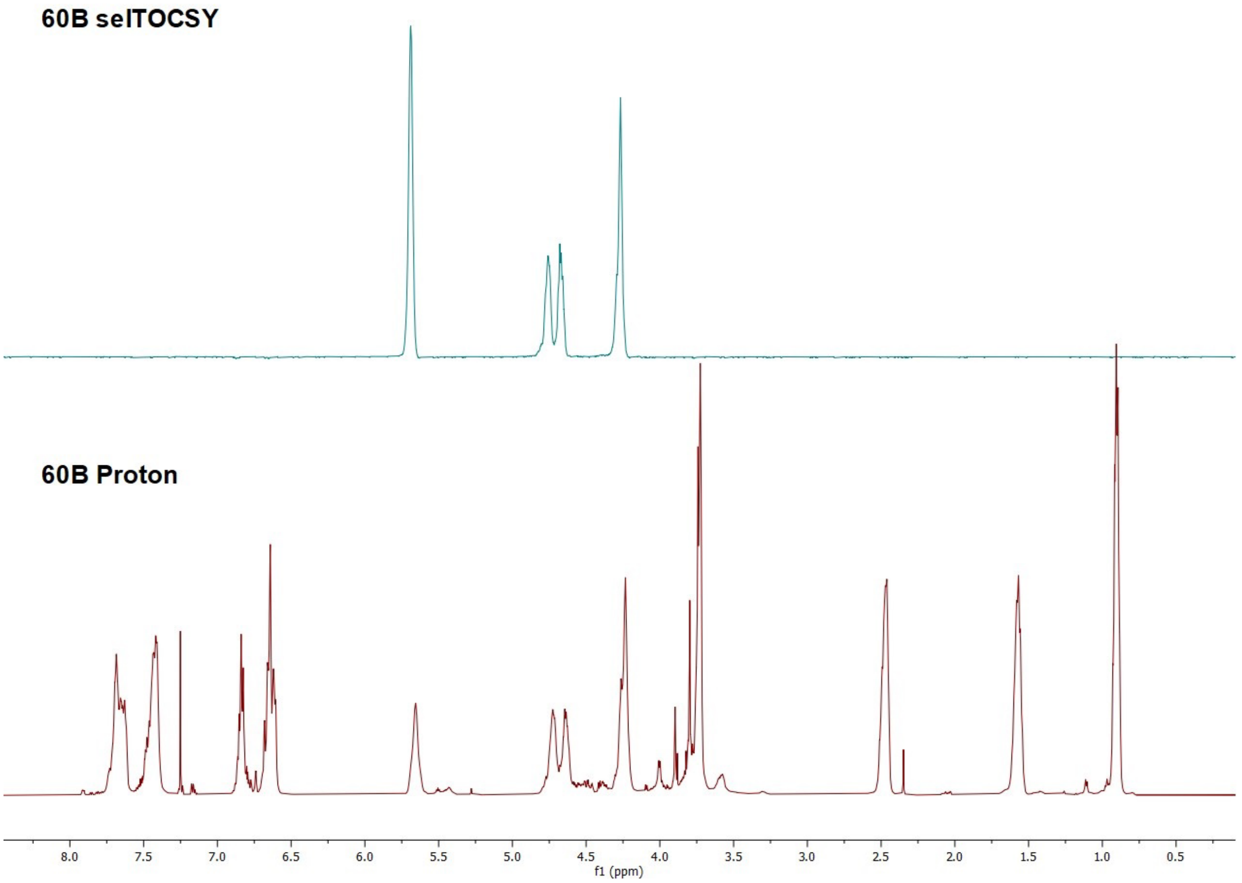

Figure S23. ^{1}H (bottom) and selective TOCSY (top, selective pulse at δ 5.69 ppm) experiments for PA / PGGE (Sample OJD60B, *Table 1*, entry 2).

A methine proton signal at δ 5.68 ppm was also observed in the ^{1}H NMR spectrum of the PA / DCAGE polymer, except that it had a smaller relative intensity compared to the PA / PGGE polymer. A selective TOCSY experiment on the PA / DCAGE polymer was performed by pulsing this signal at δ 5.68 ppm. Like for the PA / PGGE polymer, this gave corresponding signals in the range δ 4.2–4.8 ppm that correlated well to the predicted methylene proton assignments on the adjacent carbons (*Figure S24*), specific to Pathway A. HMBC NMR experiments set up for observing *J*3 coupling show correlation of the methine signal at δ 5.68 ppm to a carboxylate carbon at δ 168 ppm (*Figure S22*), in good agreement with the predicted ^{13}C NMR chemical shift assignment in *Figure S26* and further confirming Pathway A.

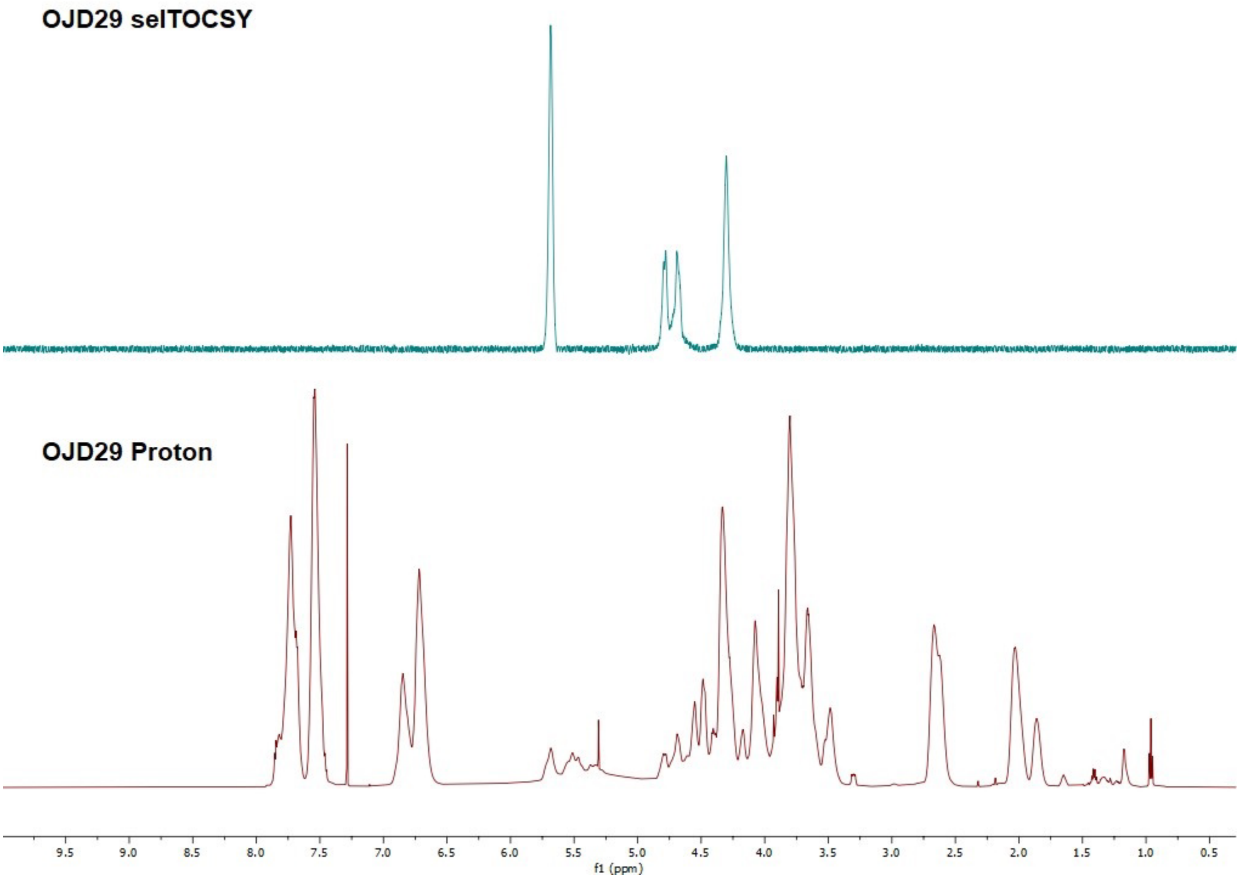

Figure S24. [1]H (bottom) and selective TOCSY (top, selective pulse at δ 5.68 ppm) experiments for PA / DCAGE (Sample OJD29, *Table 2*, entry 6).

A unique proton environment in Pathway B is the methylene protons associated with the carbon at the beta position in the side chain. These protons have a predicted chemical shift of δ 2.09 ppm, which contrasts with the predicted chemical shift of the corresponding protons for Pathway A at δ 1.82 ppm (*Figure S22*). Selective TOCSY experiments on the PA / DCAGE polymer were performed by pulsing the observed signal in the ^{1}H spectrum at δ 2.02 ppm. This gave corresponding signals at δ 2.65 and 4.31 ppm, which correlated well to the predicted proton assignments on the adjacent carbons of the side chain that are specific to Pathway B (*Figure S25*).

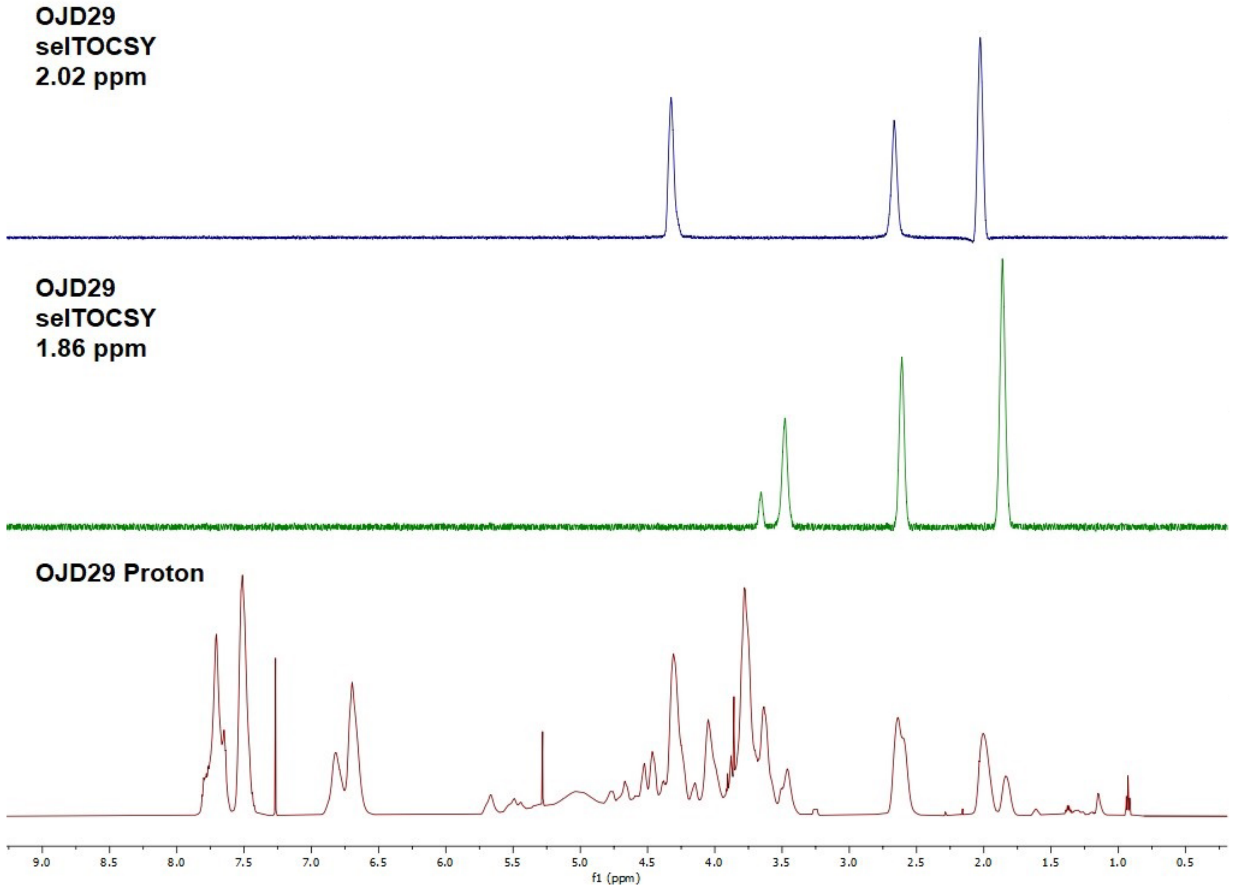

Figure S25. ^{1}H (bottom) and selective TOCSY (top/middle, pulse at δ 2.02 and 1.86 ppm) experiments for PA / DCAGE (Sample OJD29, *Table 2*, entry 6).

A unique proton environment at a predicted chemical shift of δ 1.82 ppm is specific to DCAGE subunits not polymerized at the alcohol group on the side chain, whether as co-polymerized units in Pathway A or as terminal units as part of both Pathway A and Pathway B (*Figure S22*). To confirm this, selective TOCSY experiments on the PA / DCAGE polymer were performed by pulsing the observed signal in the ^{1}H spectrum at δ 1.86 ppm. This gave corresponding signals at δ 2.59 and 3.46 ppm (*Figure S25*), which correlated well to the predicted proton assignments on the adjacent carbons of the side chain that are specific to DCAGE subunits not polymerized at the alcohol (*Figure S22*).

The distinct signals in the ^{1}H NMR spectra at δ 5.66, 2.01, and 1.84 ppm can be used to quantify the different types of co–polymer units for the PA / DCAGE polymers (*Table S13*). It

was not possible to obtain reasonable quantitative values for the SA / DCAGE polymer (Sample OJD20, *Table S9*, entry 3) as the signal for the methine proton (Pathway A) was ill defined, and the signals for the two sets of methylene protons in the side chain overlap more extensively than that for the PA / DCAGE polymers. In addition, the broad nature of the ^{1}H signals in the spectra for the PA / LHOGE and SA / LHOGE polymers made it impossible to determine integrals from any of the signals of interest.

Table S13. Relative proportions of Pathway A, Pathway B, and terminal DCAGE units for PA / DCAGE polymers as discussed in the main text

**Sample**	**Pathway A (δ 5.66 ppm)**	**Pathway B (δ 2.09 ppm)**	**Terminal DCAGE units (δ 1.82 minus 5.66 ppm)**
OJD29 (*Table 2*, entry 6)	1	2.5	≈ 0
OJD8 (*Table S4*, entry 2)	1	3	≈ 0
OJD67 (*Table 2*, entry 4)	1	4.3	0.3

All experiments for these further NMR spectroscopic studies were performed on a Bruker 600 MHz Avance NEO spectrometer equipped with a dual channel BBO iProbe. The spectrometer was operating at 600.23 MHz for ^{1}H and 150.06 MHz for ^{13}C. Standard Bruker pulse sequences were used for 1D ^{1}H, ^{13}C, and selTOCSY experiments, and 2D HSQC and HMBC experiments. ^{1}H spectra were acquired with 64 scans and a relaxation delay of 1 or 5 s. ^{13}C spectra were acquired with 10k scans and a relaxation delay of 1 s. All selTOCSY experiments used an excitation pulse with an 80 ms mixing time. Two-dimensional spectra were acquired with a f1 x f2 dimensions suiting the resolution required for the experiment and were acquired using 50% NUS.

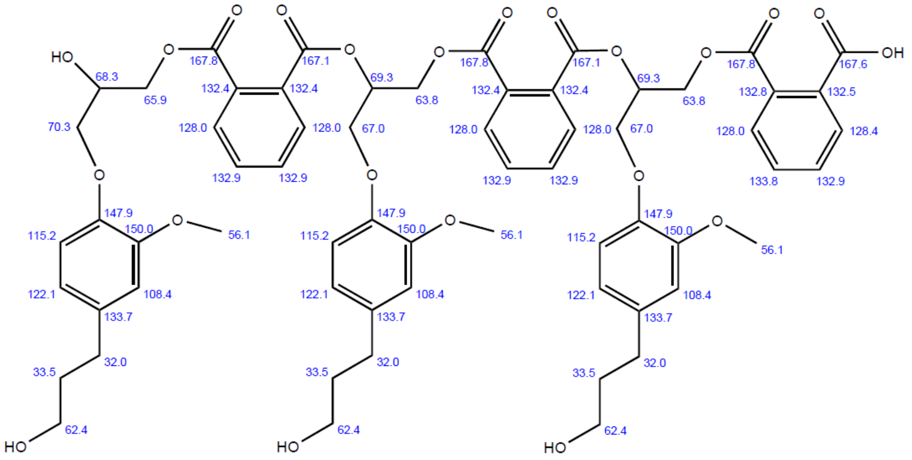

Figure S26. Predicted ^{13}C NMR chemical shift assignments for PA / DCAGE–Pathway A.

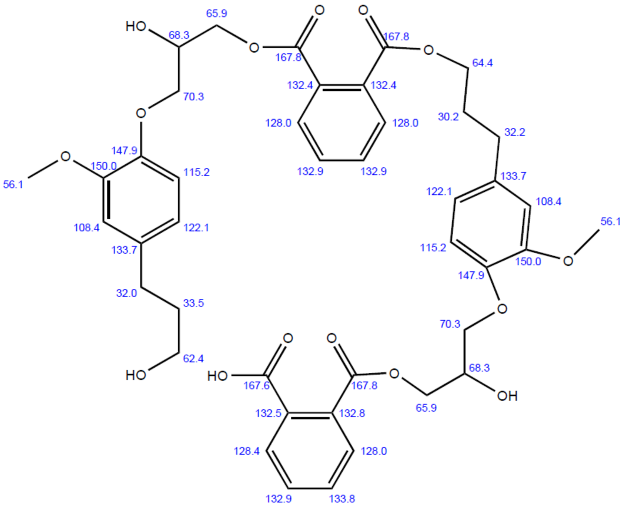

Figure S27. Predicted ^{13}C NMR chemical shift assignments for PA / DCAGE–Pathway A.

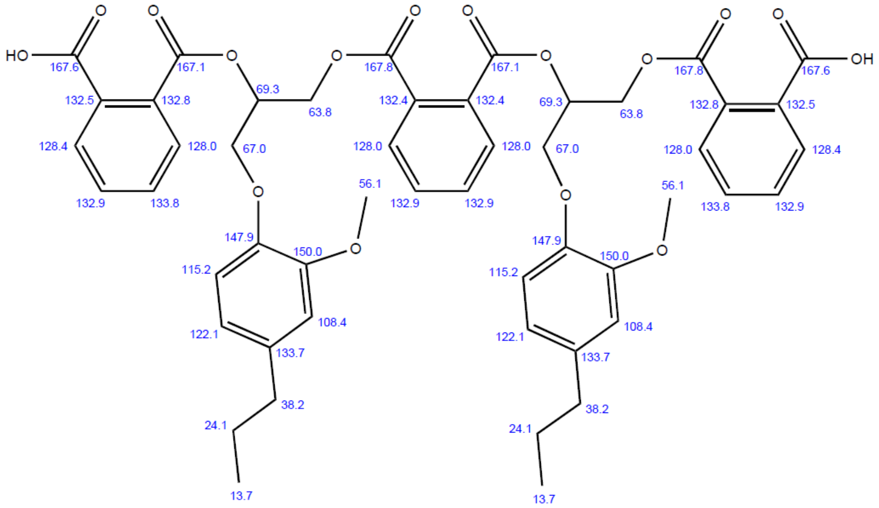

Figure S28. Predicted ^{13}C NMR chemical shift assignments for PA / DCAGE–Pathway A.

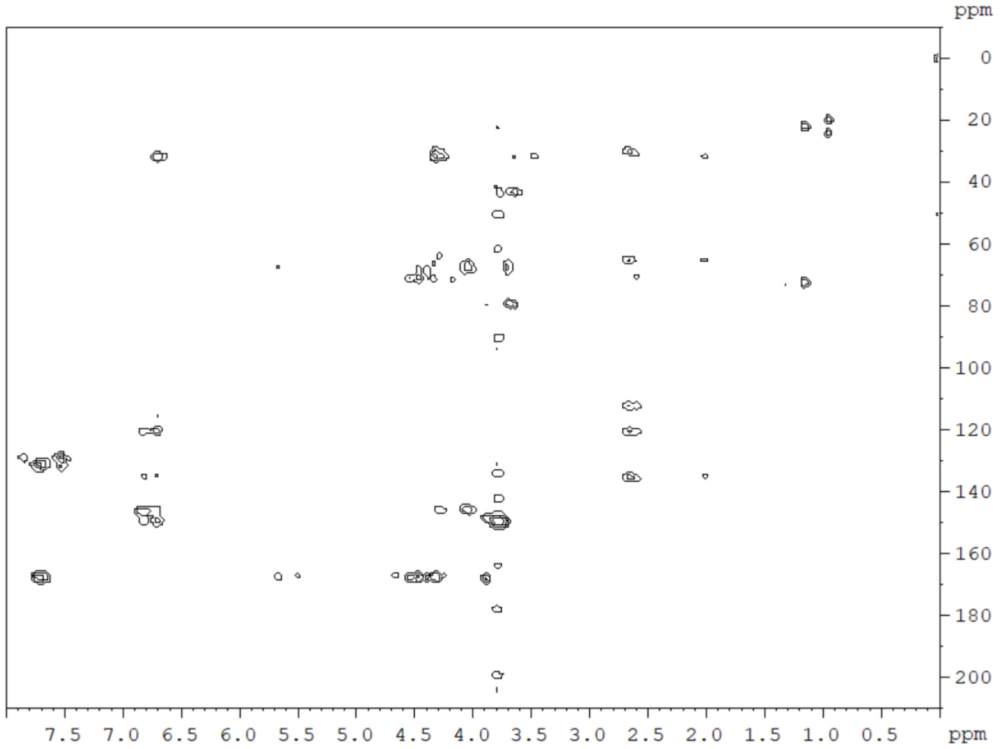

Figure S29. HMBC of PA / DCAGE polymer (Sample OJD29, *Table 2*, entry 6)

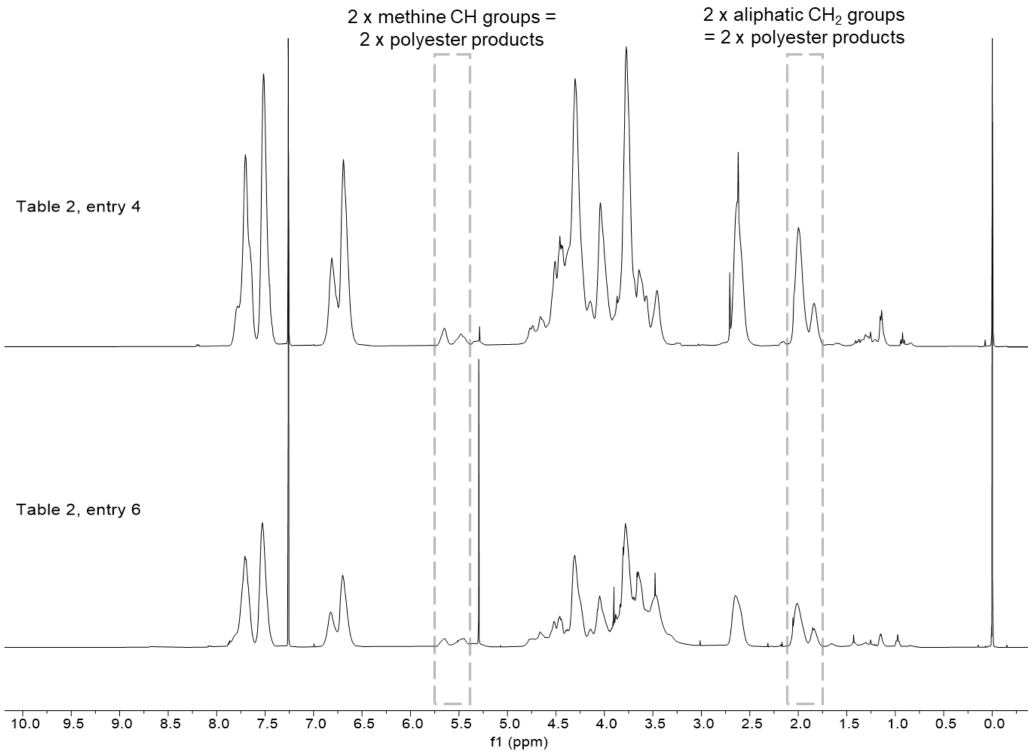

Figure S30. ^{1}H NMR spectra of *Table 2*, entries 4 and 6 for the poly(PA–*co*–DCAGE) polyester samples. Two less crowded regions are highlighted (δ 1.75–2.10 ppm and δ 5.40–5.75 ppm), both containing two resonance peaks, possibly suggesting two polyester products (**Pathway A** and **Pathway B**).

S2.9. The application of lignin–derived ROCOP polyesters as polyols for the synthesis of polyurethane film materials (*Figures S31–34* and *Table S14*)

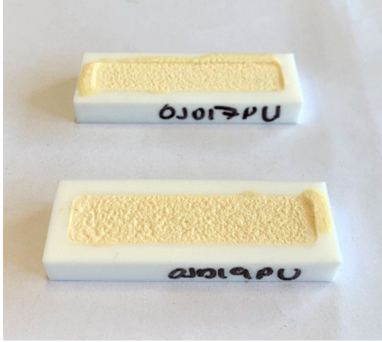

Figure S31. A picture of extremely brittle, unsuccessful PU films synthesized from poly(PA–*co*–DCAGE) polyester polyol and methylene diphenyl diisocyanate.

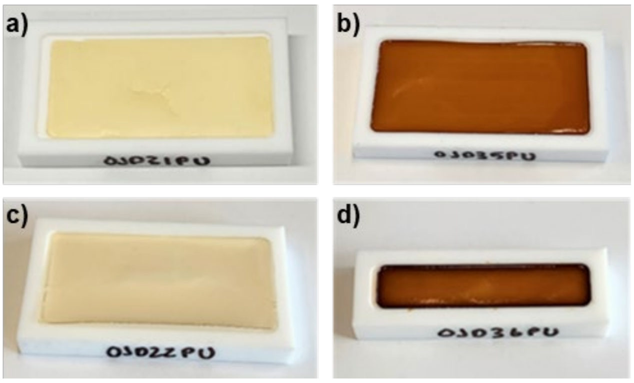

Figure S32. Pictures of example PU thin films obtained from the lignin-derived polyester polyols: a) poly(PA–co–DCAGE) polyol (*Table 2*, entry 4), b) poly(PA–co–LHOGE) polyol (*Table S8*, entry 6), c) poly(SA–co–DCAGE) polyol (*Table S9*, entry 3), d) poly(SA–co–LHOGE) polyol (*Table S9*, entry 7).

Table S14. The overall thermomechanical properties of the PU films, synthesized from lignin-derived polyester polyols, measured using dynamic mechanical thermal analysis and tensile stress–strain curves.

Polyester polyol	Poly(PA–*co*–DCAGE)	Poly(PA–*co*–LHOGE)	Poly(SA–*co*–DCAGE)	Poly(SA–*co*–LHOGE)
Polyester sample source	*Table 2*, entry 4	*Table S8*, entry 6	*Table S9*, entry 3	*Table S9*, entry 7
[DCAGE/LHOGE content] (wt%)	46	57	55	63
[Biomass-derived content] (wt%)	75	77	78	79
$T_{g,\tan\delta}$ (°C)	73.8 ± 0.61	66.9 ± 1.4	10.1 ± 0.80	62.5 ± 1.6
Young's modulus (MPa)	1450 ± 51	1469 ± 266	37.6 ± 2.7	1294 ± 95
Ultimate tensile strength (MPa)	40.0 ± 5.8	5.59 ± 1.1	2.15 ± 0.17	28.3 ± 3.3
Elongation at break (%)	5.40 ± 0.99	0.436 ± 0.12	30.8 ± 3.2	42.7 ± 12

Expanding on the discussion from ***The application of lignin-derived ROCOP polyesters as polyols for the synthesis of polyurethane film materials*** section of the main text, it was mentioned that the molecular weight of the polyester polyol was found to influence the brittleness of the poly(PA–*co*–DCAGE) PU film. It was observed that the extent of brittleness dramatically worsened for the poly(PA–*co*–DCAGE) PU film when the molecular weight of the polyester polyol was lowered. It was challenging to handle the specimens for thermomechanical testing without accidently breaking, but DMTA responses were obtained, and there was a lowering of the T_g from 73.8 °C to 45 °C (*Table 2,* entry 4, M_n = 2800 g.mol^{-1} *vs*. *Table 2*, entry 6, M_n = 1950 g.mol^{-1}) (*Figure S33*; *b*), T_g = 44.48 °C and *c*), T_g = 45.44 °C), agreeing with the physical observations. Furthermore, the tanδ curves were incredibly broad

with possible, noticeable shoulder peaks. This could be indicative of two polymer species present in the PU potentially from the separate poly(PA–*co*–DCAGE) polyester and HDI crosslinking species. To further reinforce the high degree of brittleness, one tensile stress–strain curve was able to be obtained which agreed with these findings (Young's modulus = 765 MPa, ultimate tensile strength = 5.89 MPa, and elongation at break = 0.97%).

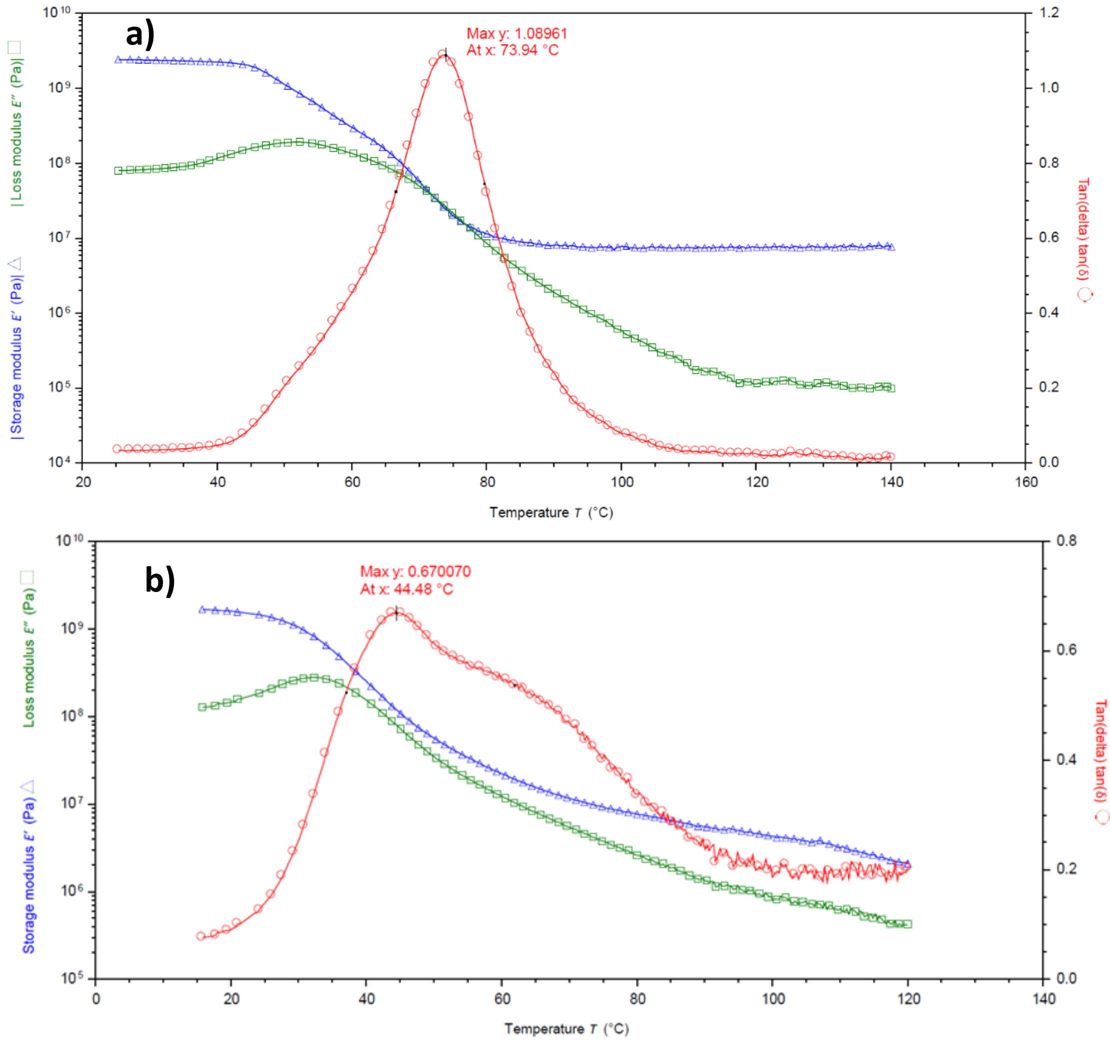

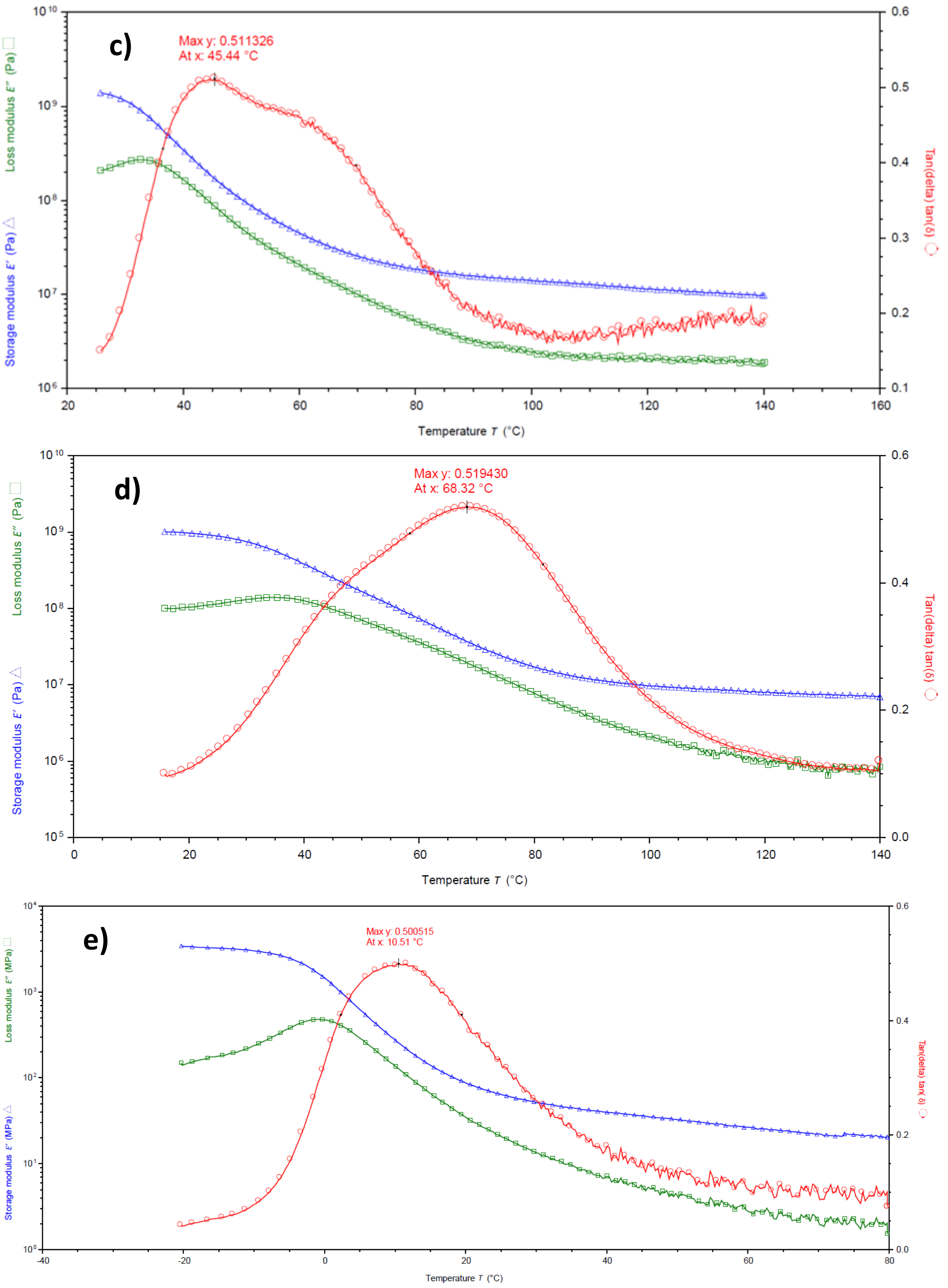

c)
Max y: 0.511326
At x: 45.44 °C
Loss modulus E'' (Pa)
Storage modulus E' (Pa)
Tan(delta) tan(δ)
Temperature T (°C)
d)
Max y: 0.519430
At x: 68.32 °C
Loss modulus E'' (Pa)
Storage modulus E' (Pa)
Tan(delta) tan(δ)
Temperature T (°C)
e)
Max y: 0.500515
At x: 10.51 °C
Loss modulus E'' (MPa)
Storage modulus E' (MPa)
Tan(delta) tan(δ)
Temperature T (°C)

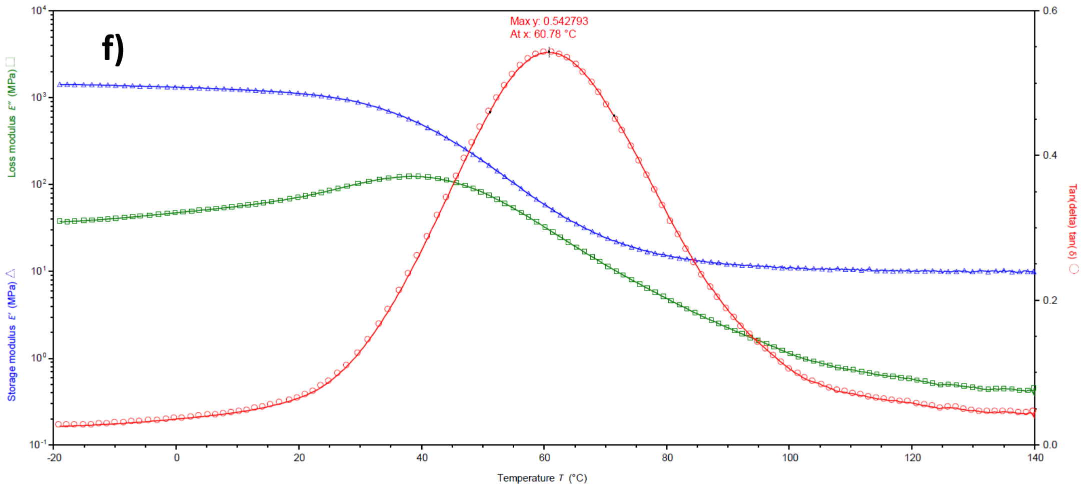

Figure S33. Example dynamic mechanical thermal analysis responses of the PU films synthesized from lignin-derived polyester polyols. *a*) Poly(PA–*co*–DCAGE) polyol (*Table 2, entry 4*). *b*) Poly(PA–*co*–DCAGE) polyol (*Table 2, entry 6*). *c*) Poly(PA–*co*–DCAGE) polyol (*Table 2, entry 4*). *d*) Poly(PA–*co*–LHOGE) polyol (*Table S8, entry 6*). *e*) Poly(SA–*co*–DCAGE) polyol (*Table S9, entry 3*). *f*) Poly(SA–*co*– LHOGE) polyol (*Table S9, entry 7*). Further DMTA responses are shown below.

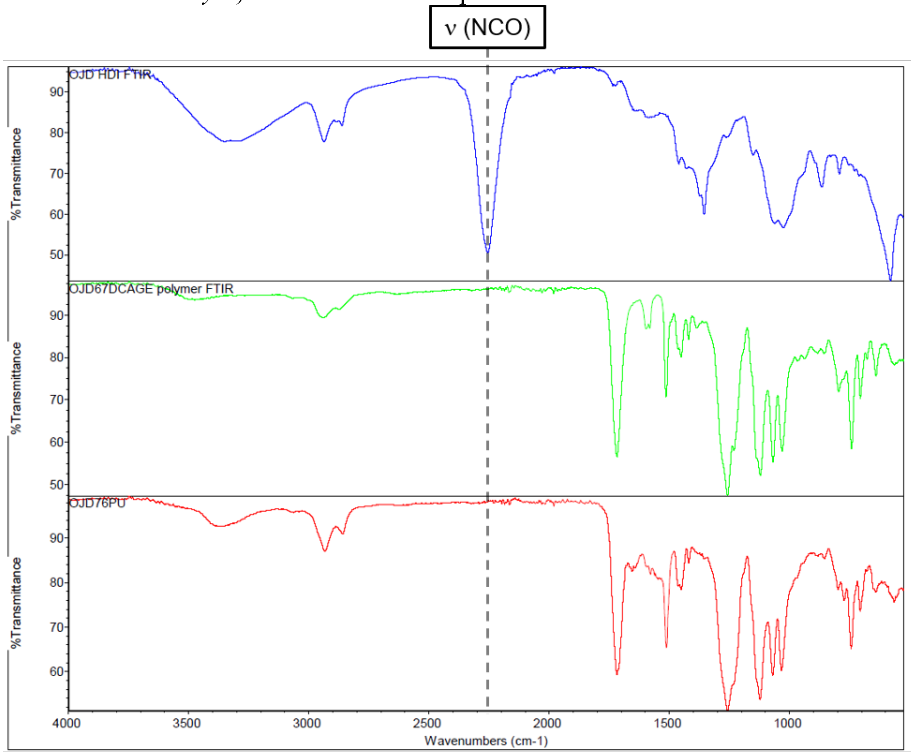

Figure S34. Example stacked FT–IR spectra of a PU thin film product (labelled 'OJD76PU'), compared to the HDI and poly(PA–*co*–DCAGE) polyol (labelled 'OJD67DCAGE') reagents; the disappearance of the NCO stretch (ν) in the PU product can be observed that indicated complete reactivity and conversion to the desired thermoset material.

S3. Characterization data

S3.1. ROCOP and polyester characterization

S3.1.1. Crude ^{1}H NMR spectra of ROCOP reaction mixtures and of isolated polymers (*Figures S35–66*)

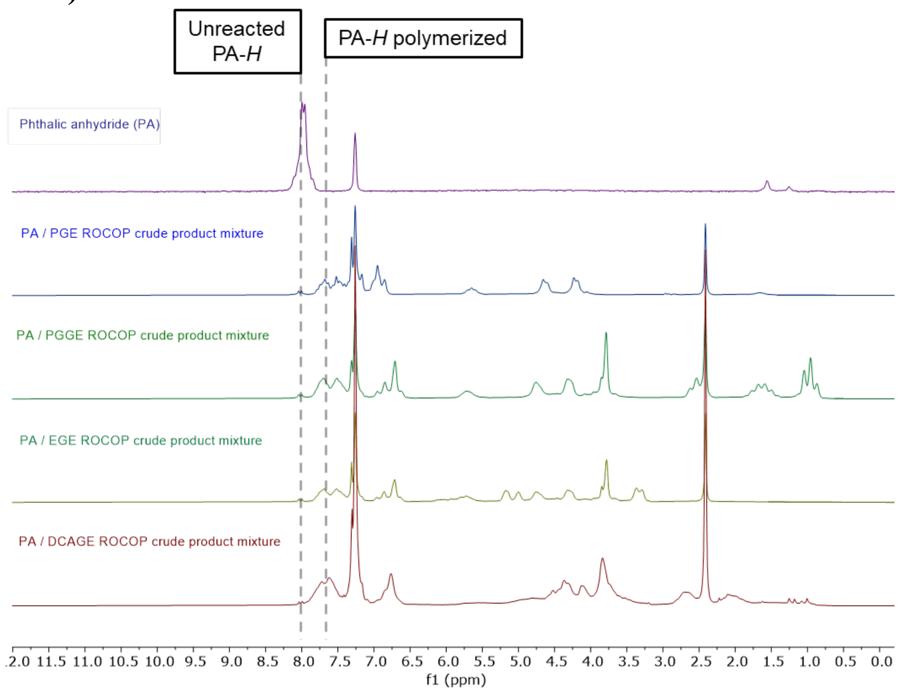

Figure S35. Stacked ^{1}H NMR spectra of PA compared to the solution ROCOP crude reaction mixtures coupling PA with PGE, PGGE, EGE and DCAGE substrates respectively; in all cases, the PA fully reacts with the respective epoxidized lignin model substrates, as highlighted on the figure (*Table 1*, entries 1, 2, 3 and 4).

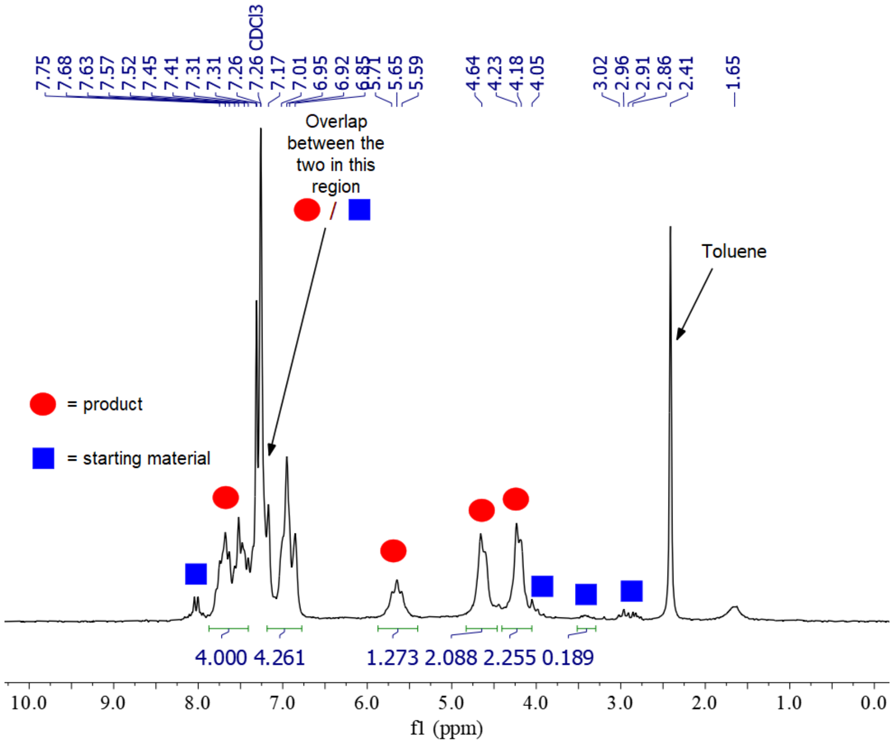

Figure S36. Crude ^{1}H NMR spectrum of the solution PA / PGE ROCOP reaction mixture (*Table 1*, entry 1).

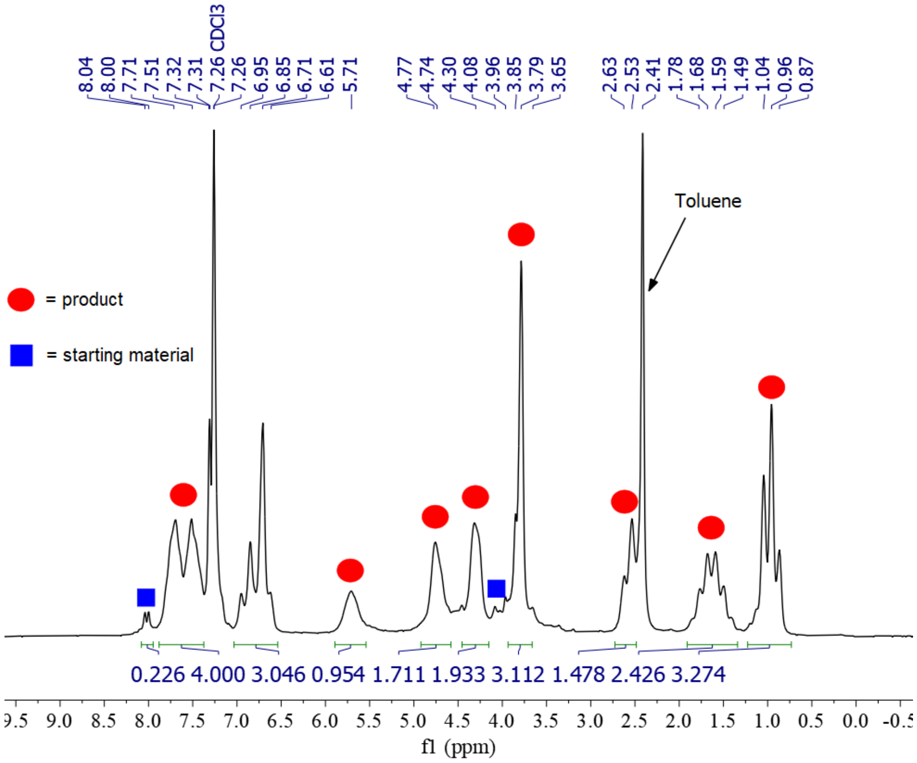

Figure S37. Crude ^{1}H NMR spectrum of the solution PA / PGGE ROCOP reaction mixture (*Table 1*, entry 2).

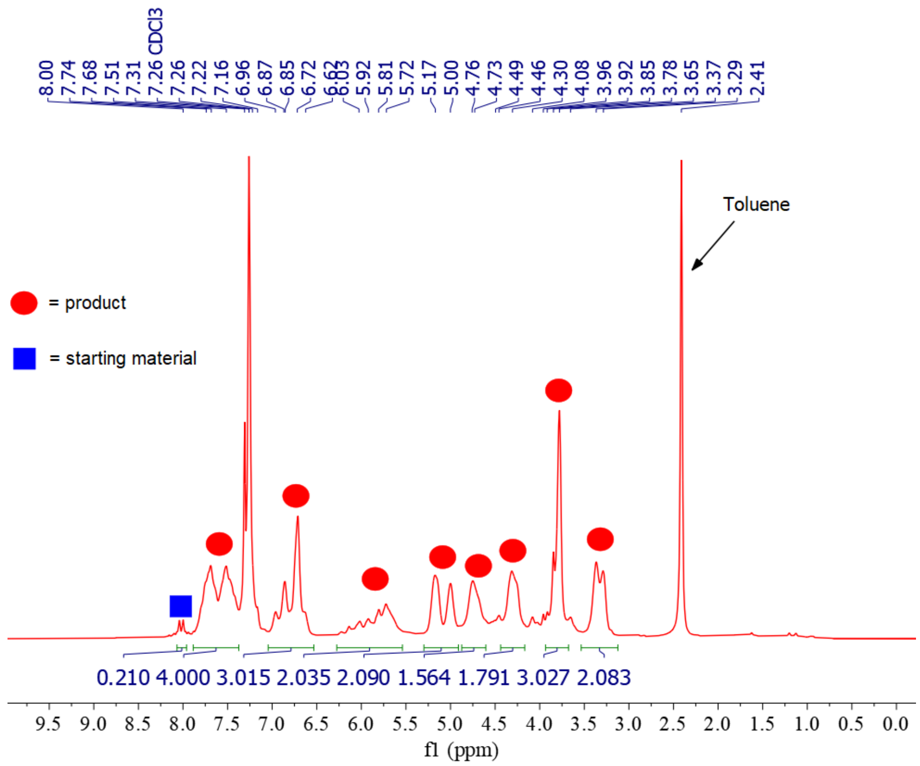

Figure S38. Crude ^{1}H NMR spectrum of the solution PA / EGE ROCOP reaction mixture (*Table 1*, entry 3).

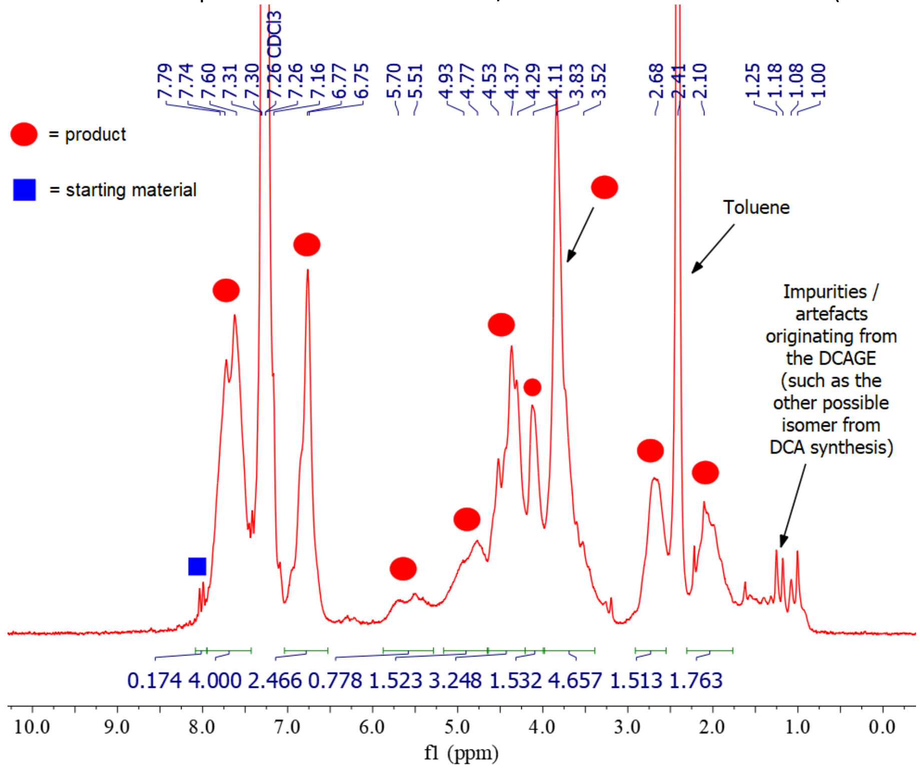

Figure S39. Crude ^{1}H NMR spectrum of the solution PA / DCAGE ROCOP reaction mixture (*Table 1*, entry 4).

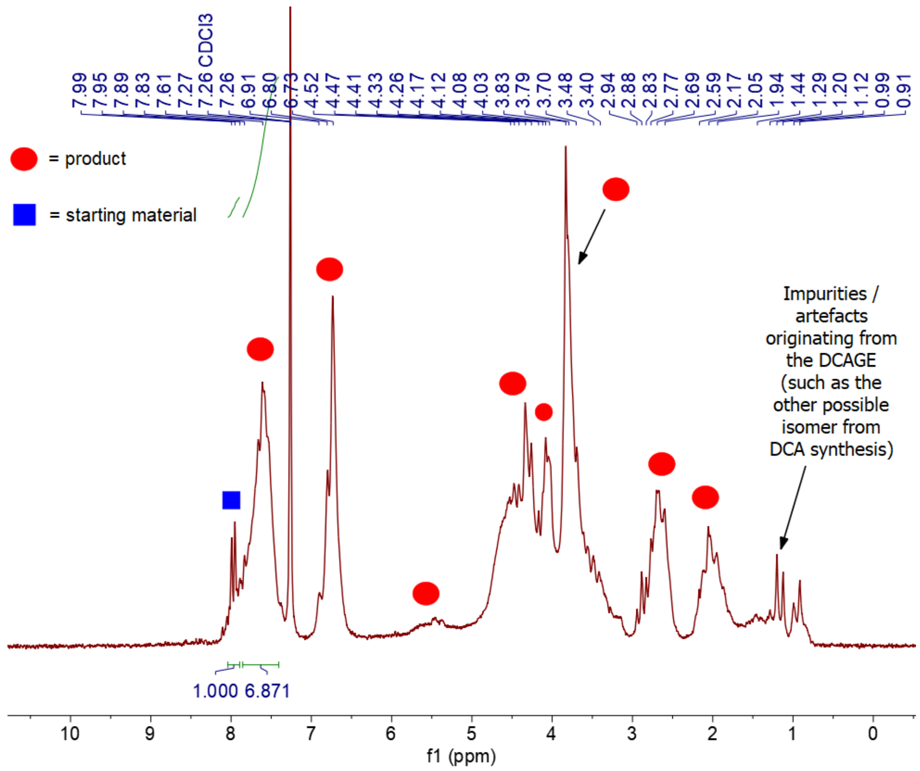

Figure S40. Crude ^{1}H NMR spectrum of the PA / DCAGE ROCOP reaction mixture (*Table 2*, entry 4).

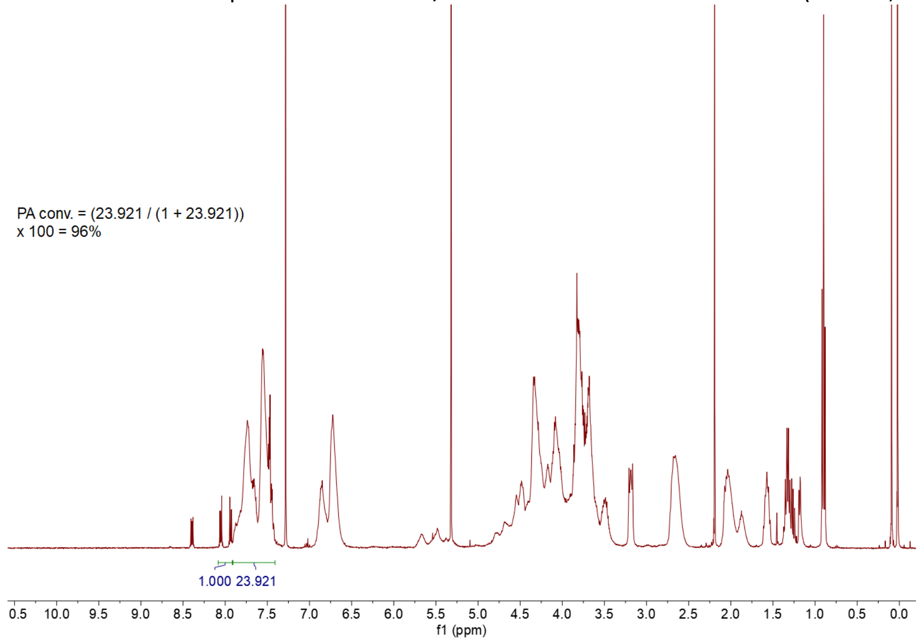

Figure S41. Crude ^{1}H NMR spectrum of the PA / DCAGE ROCOP reaction mixture (*Table 2*, entry 7).

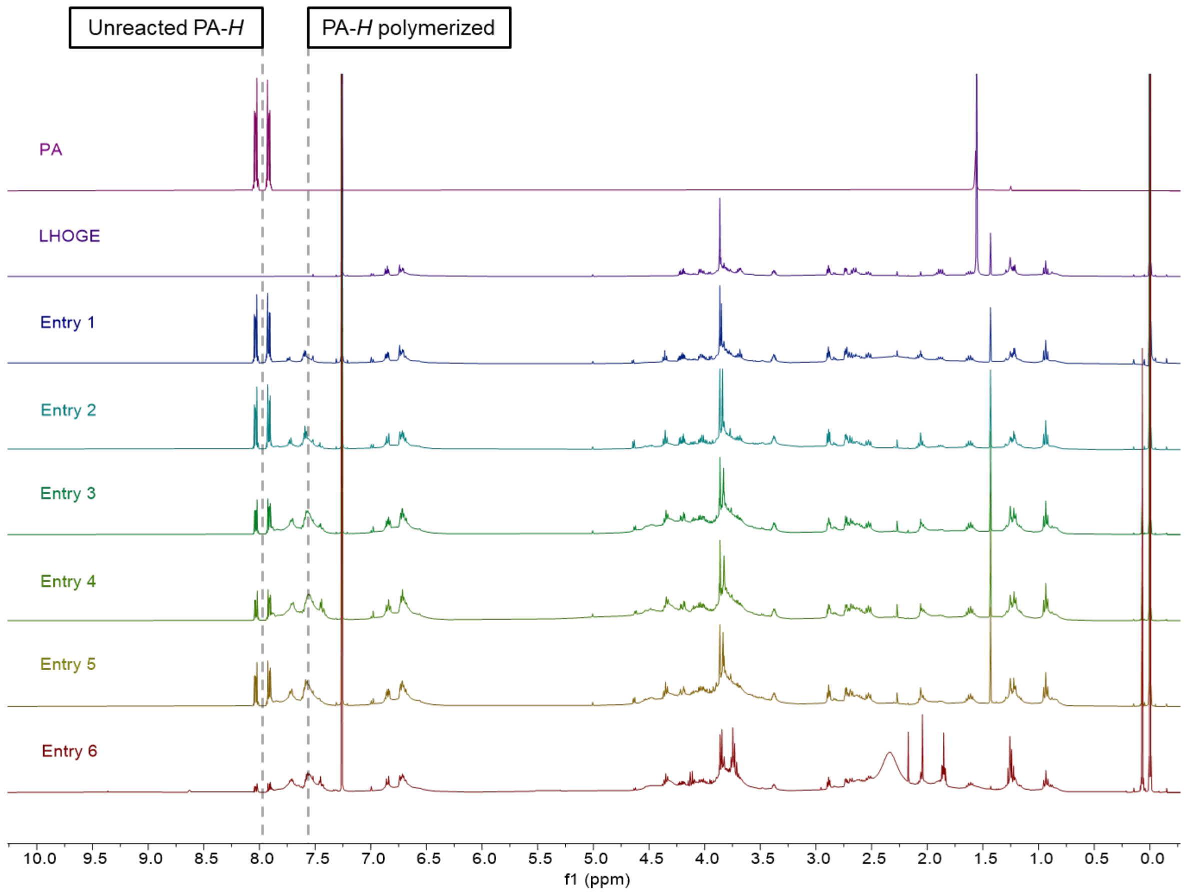

Figure S42. Stacked ^{1}H NMR spectra of PA, LHOGE and PA / LHOGE ROCOP crude reaction mixtures for *Table S8* (entries 1, 2, 3, 4, 5 and 6 respectively).

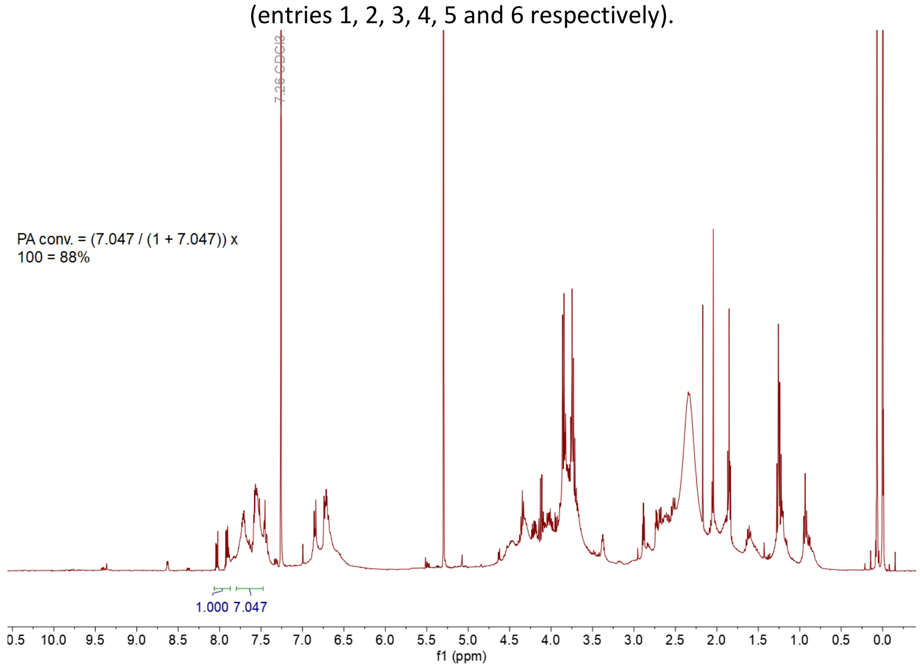

Figure S43. Crude ^{1}H NMR spectrum of the PA / LHOGE ROCOP reaction mixture (*Table S8*, entry 6).

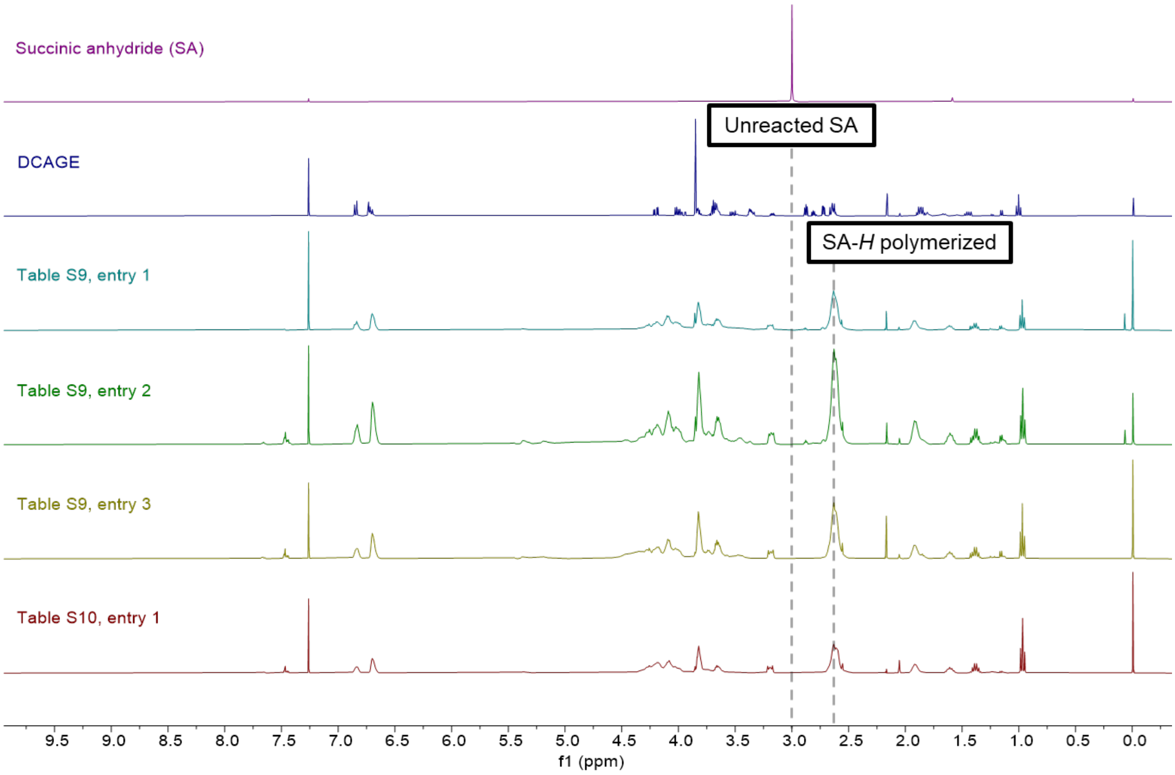

Figure S44. Stacked ¹H NMR spectra of SA, DCAGE, and the SA / DCAGE ROCOP crude reaction mixtures for *Table S9* (entries 1, 2, 3) and *Table S10*, entry 1 respectively; in all cases, the SA fully reacts with the DCAGE to form a new broader resonance at δ 2.63 ppm, as highlighted on the figure.

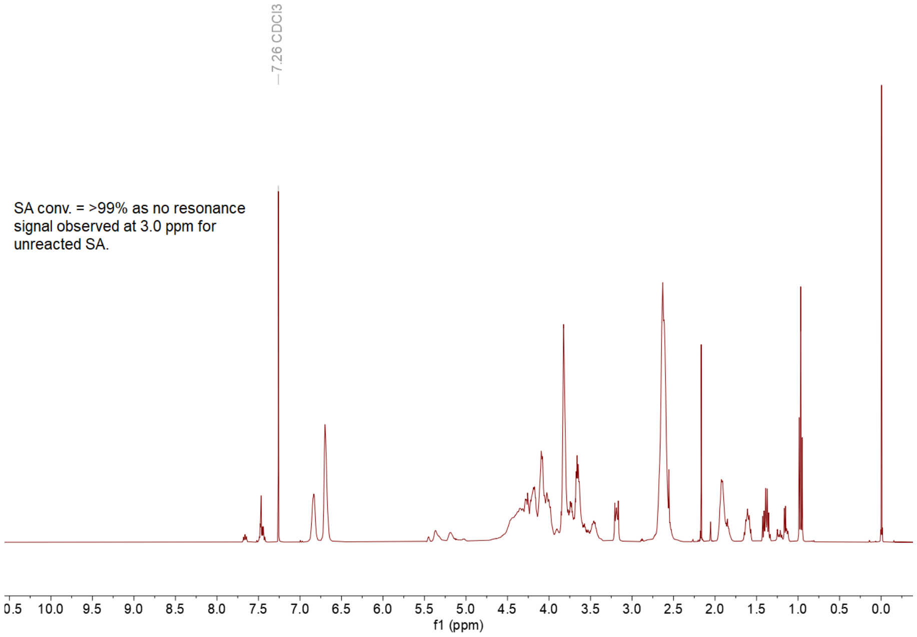

Figure S45. Crude ¹H NMR spectrum of the SA / DCAGE ROCOP reaction mixture (*Table S9*, entry 3).

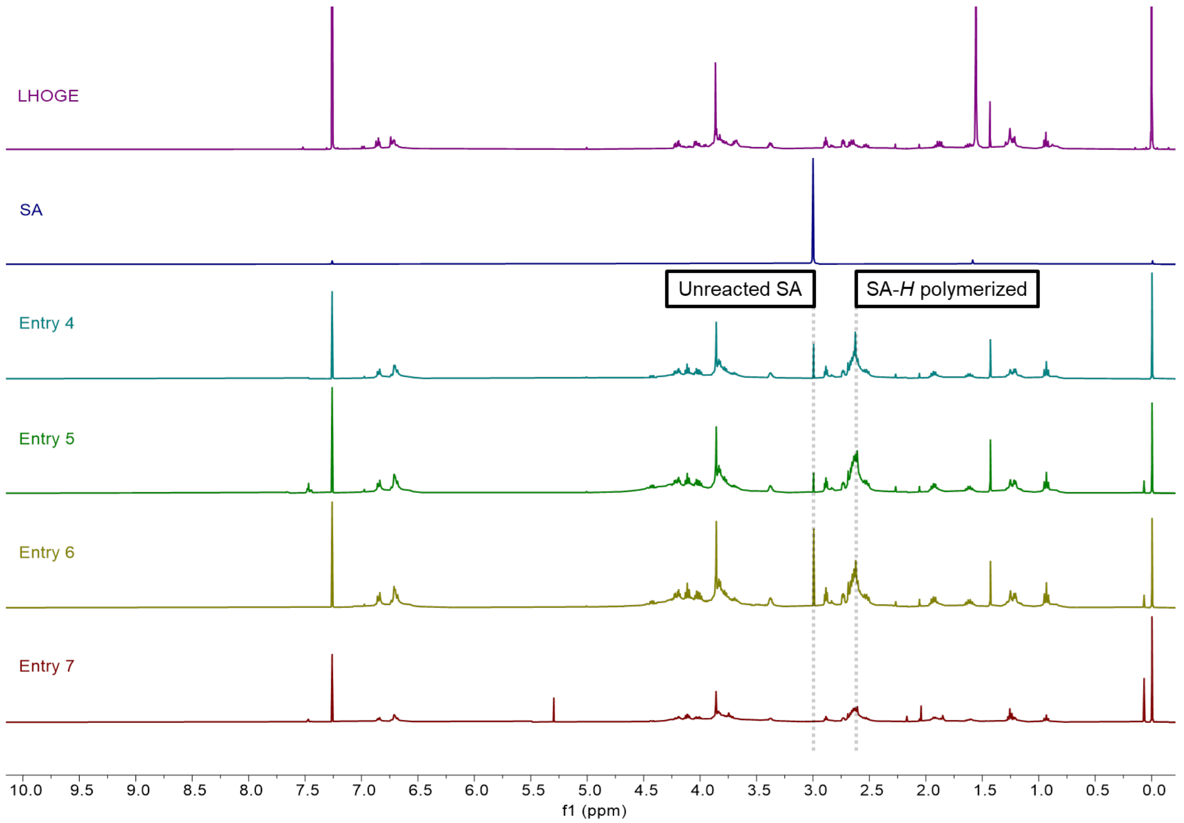

Figure S46. Stacked ^{1}H NMR spectra of SA, LHOGE and SA / LHOGE ROCOP crude reaction mixtures for *Table S9* (entries 4, 5, 6 and 7 respectively).

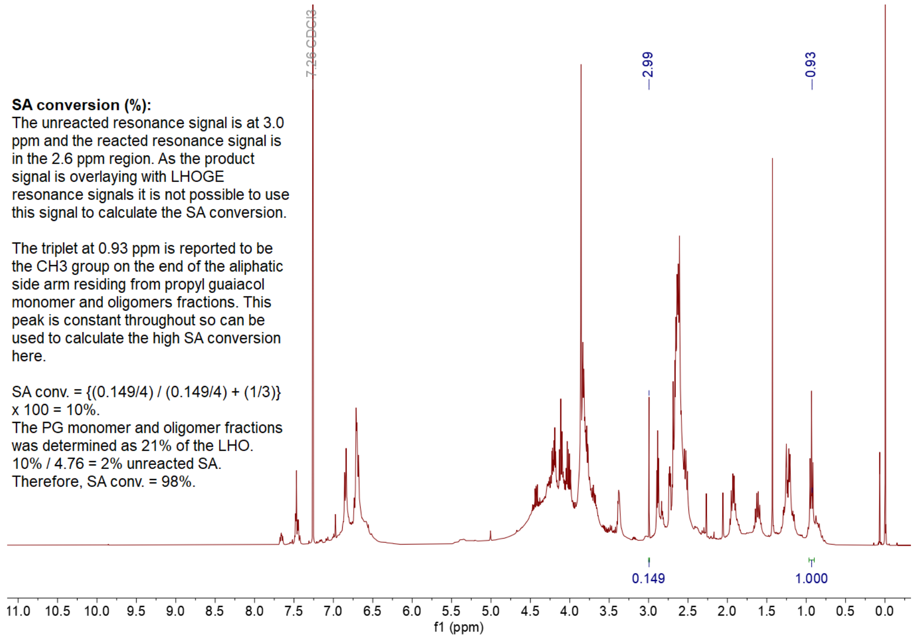

Figure S47. Crude ^{1}H NMR spectrum of the SA / LHOGE ROCOP reaction mixture (*Table S9*, entry 5). The triplet at δ 0.93 ppm, for the CH_3 end group, present in 21% of the species in the LHO (PG monomers and oligomers) was calculated from our previous literature.[8]

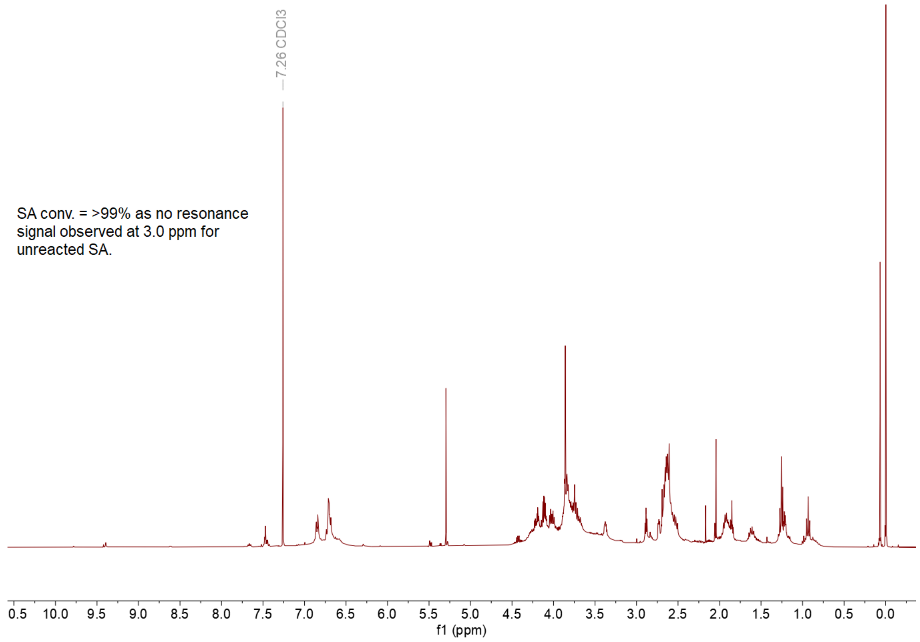

Figure S48. Crude ^{1}H NMR spectrum of the SA / LHOGE ROCOP reaction mixture (*Table S9*, entry 7).

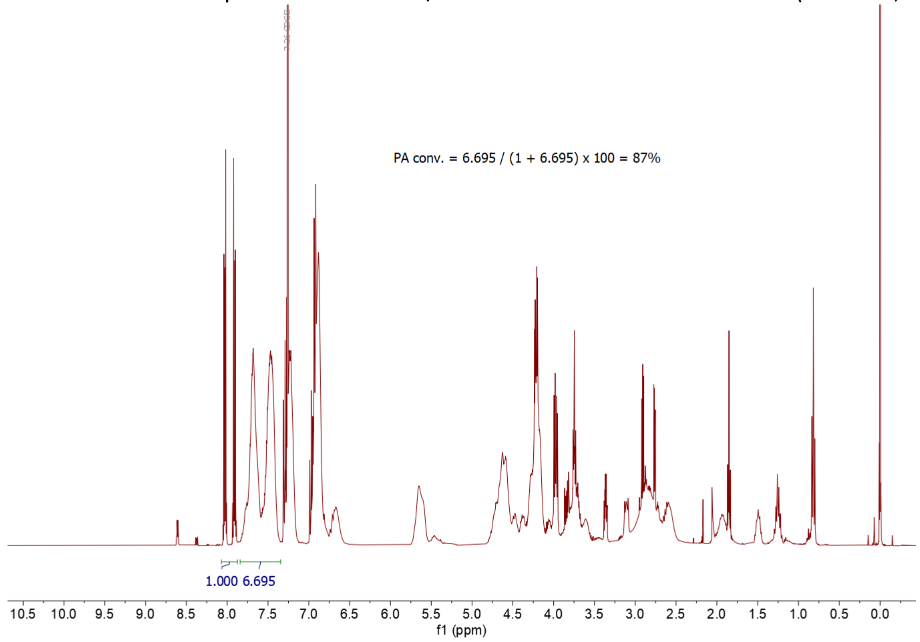

Figure S49. Crude ^{1}H NMR spectrum of the isolated crude product mixture obtained from spiking 25% [DCAGE] into the PA / PGE ROCOP using no PPNCl organic catalyst (*Table S6*).

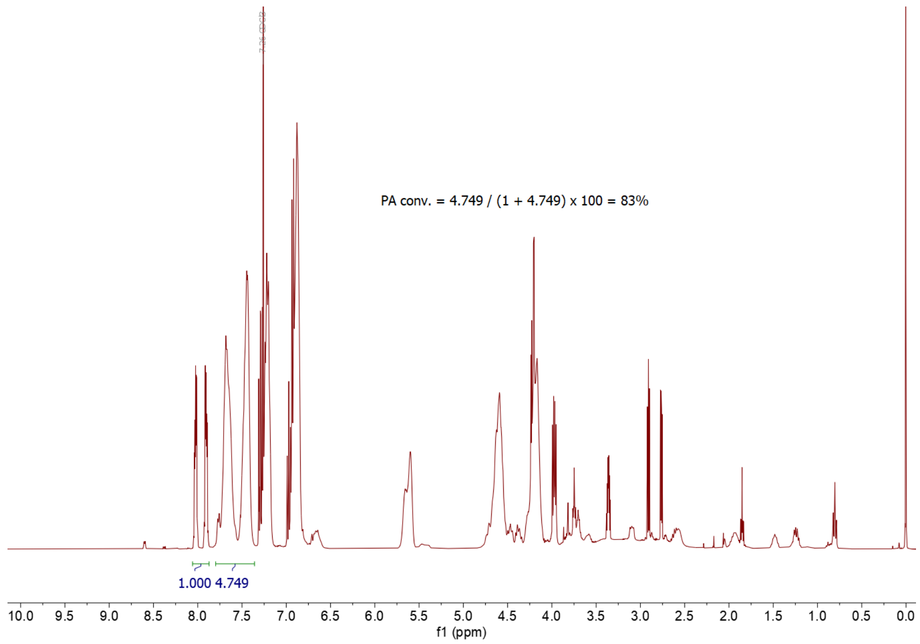

Figure S50. Crude ^{1}H NMR spectrum of the isolated crude product mixture obtained from spiking 10% [DCAGE] into the PA / PGE ROCOP using no PPNCl organic catalyst (*Table S6*).

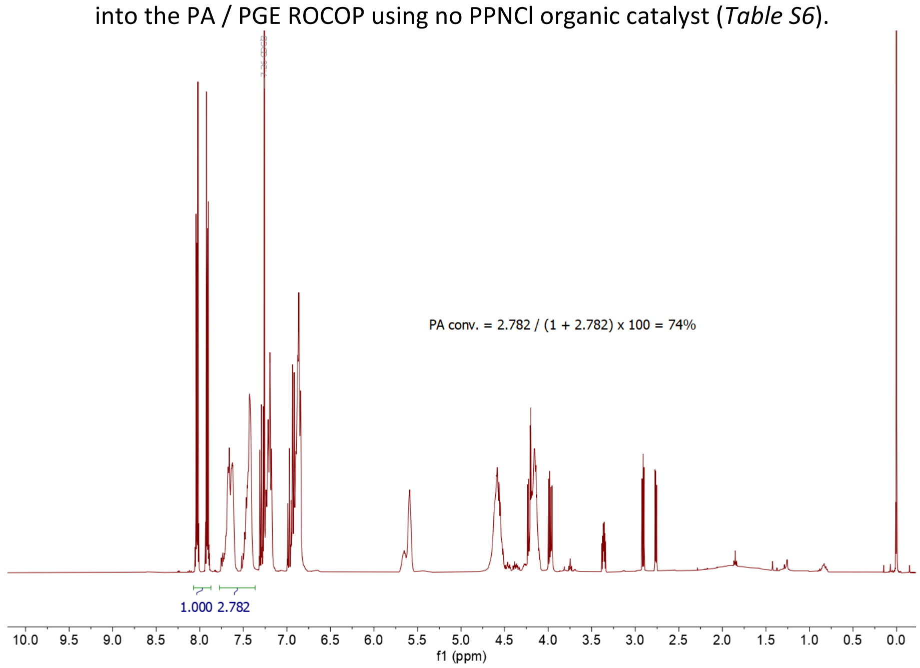

Figure S51. Crude ^{1}H NMR spectrum of the isolated crude product mixture obtained from spiking 1% [DCAGE] into the PA / PGE ROCOP using no PPNCl organic catalyst (*Table S6*).

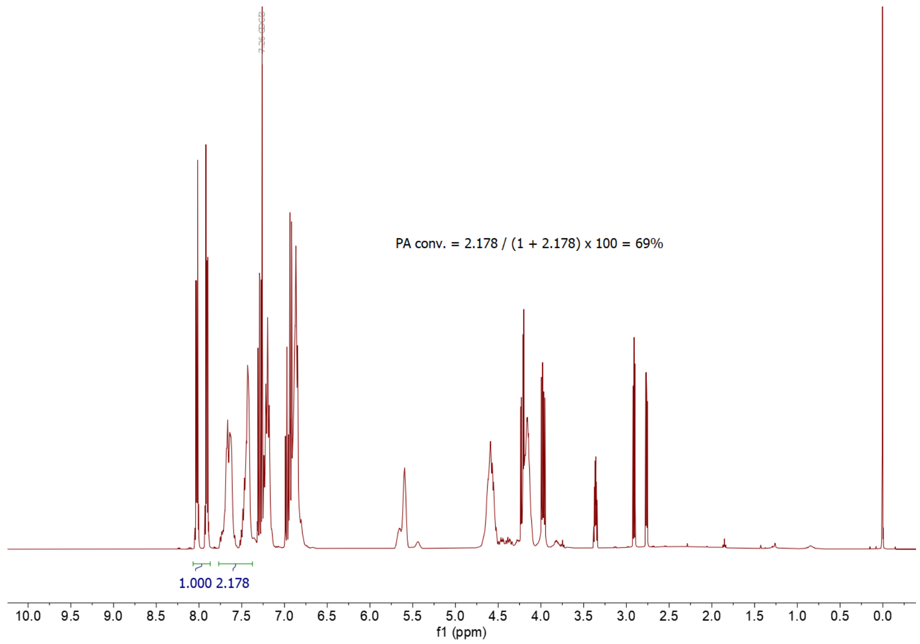

Figure S52. Crude ^{1}H NMR spectrum of the isolated crude product mixture obtained from spiking 0.1% [DCAGE] into the PA / PGE ROCOP using no PPNCl organic catalyst (*Table S6*).

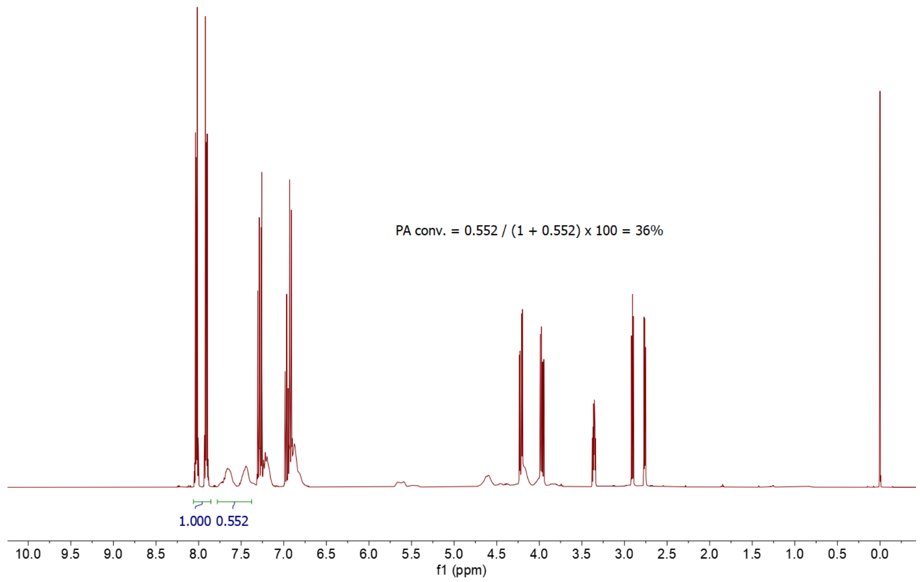

Figure S53. Crude ^{1}H NMR spectrum of the isolated crude product mixture obtained from spiking 0% [DCAGE] into the PA / PGE ROCOP using no PPNCl organic catalyst (*Table S6*).

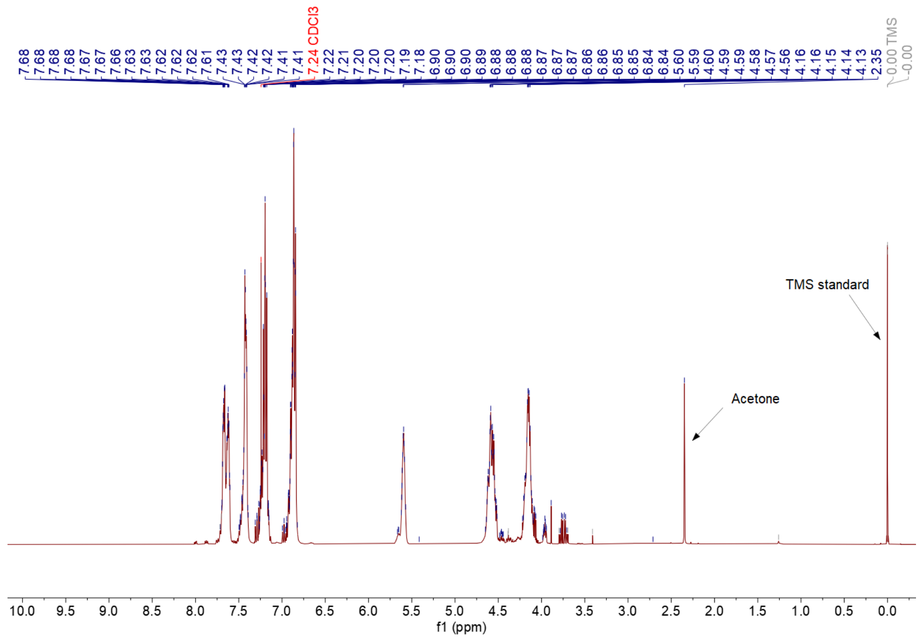

Figure S54. ^{1}H NMR spectrum of the isolated polymer obtained from the PA / PGE ROCOP (*Table 1*, entry 1).

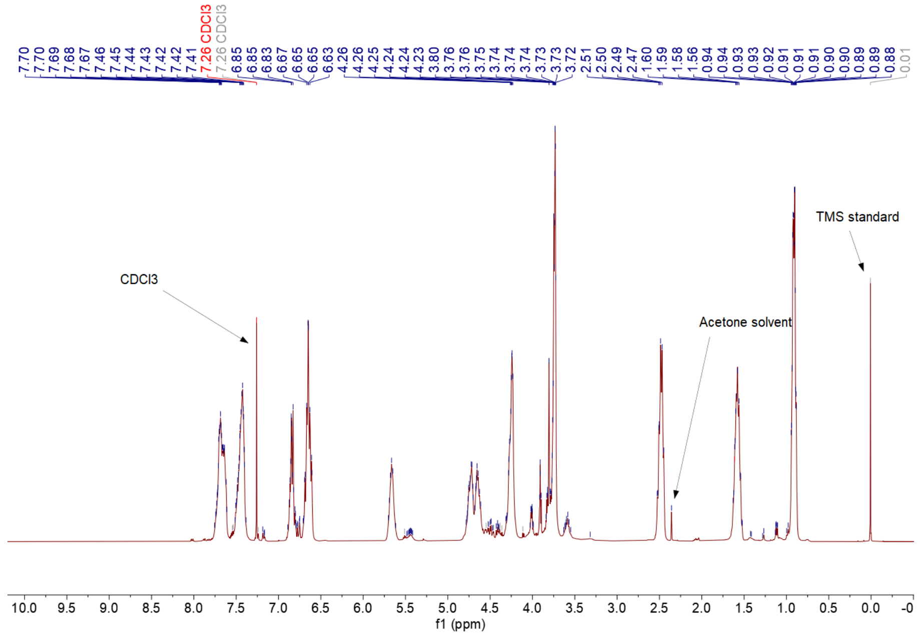

Figure S55. ^{1}H NMR spectrum of the isolated polymer obtained from the PA / PGGE ROCOP (*Table 1*, entry 2).

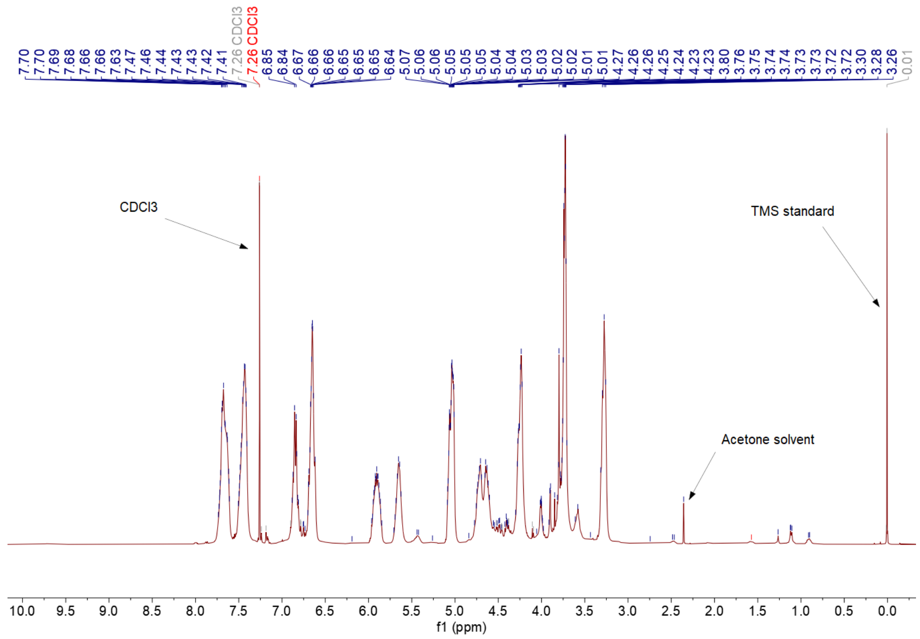

Figure S56. ^{1}H NMR spectrum of the isolated polymer obtained from the PA / EGE ROCOP (*Table 1*, entry 3).

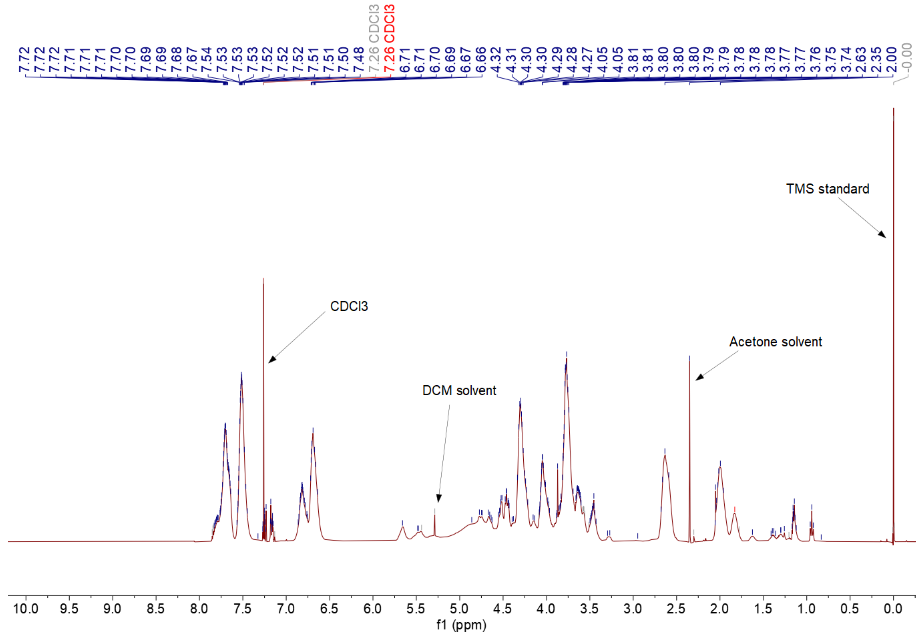

Figure S57. ^{1}H NMR spectrum of the isolated polymer obtained from the PA / DCAGE ROCOP (*Table 1*, entry 4).

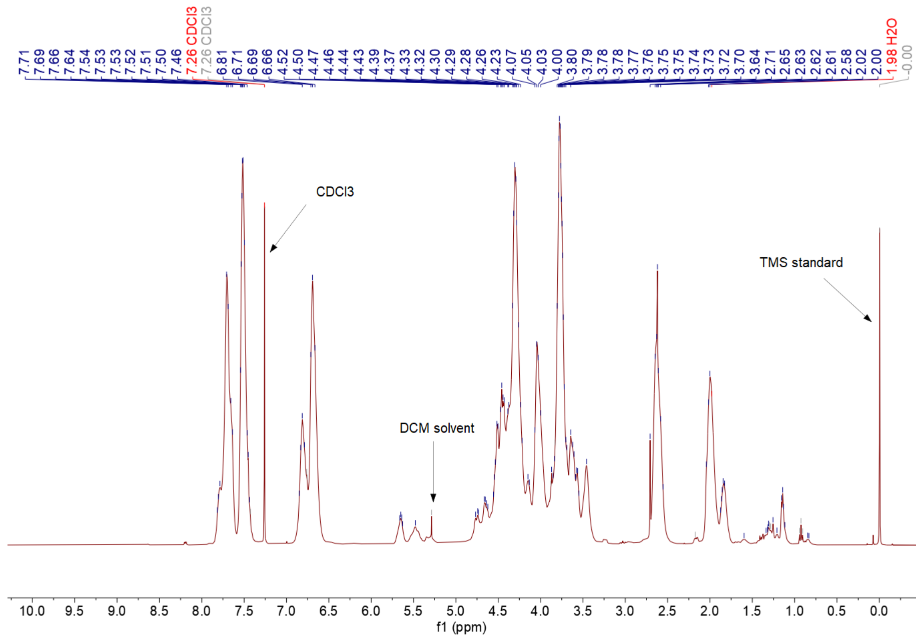

Figure S58. ¹H NMR spectrum of the isolated polymer obtained from the PA / DCAGE ROCOP (*Table 2*, entry 4).

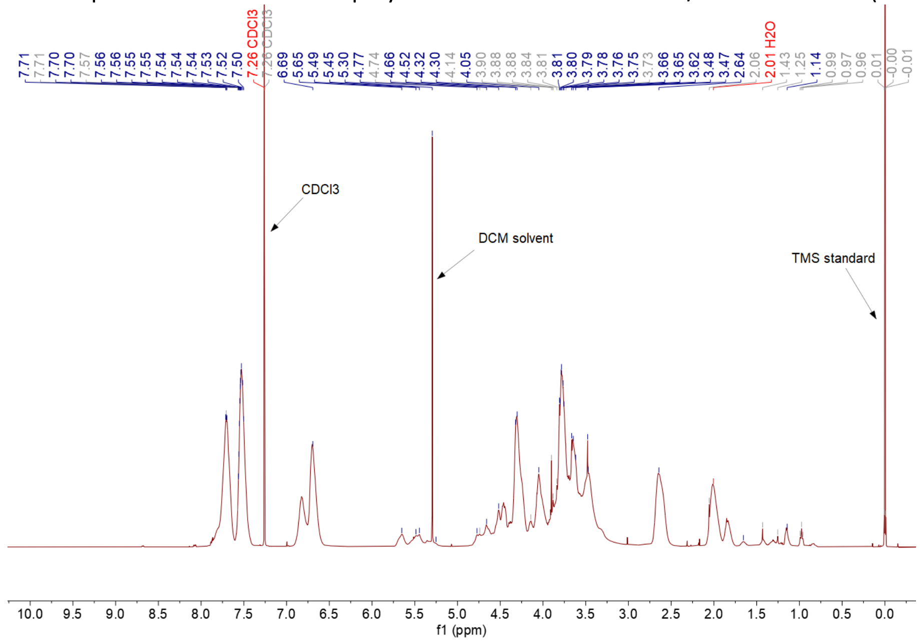

Figure S59. ¹H NMR spectrum of the isolated polymer obtained from the PA / DCAGE ROCOP (*Table 2*, entry 6).

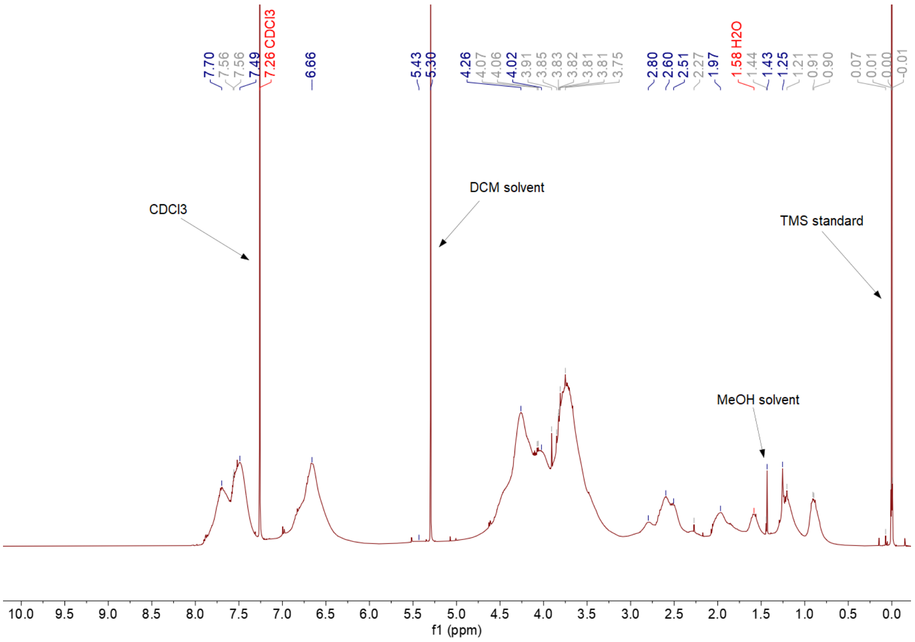

Figure S60. ^{1}H NMR spectrum of the isolated polymer obtained from the PA / LHOGE ROCOP (*Table S8*, entry 1).

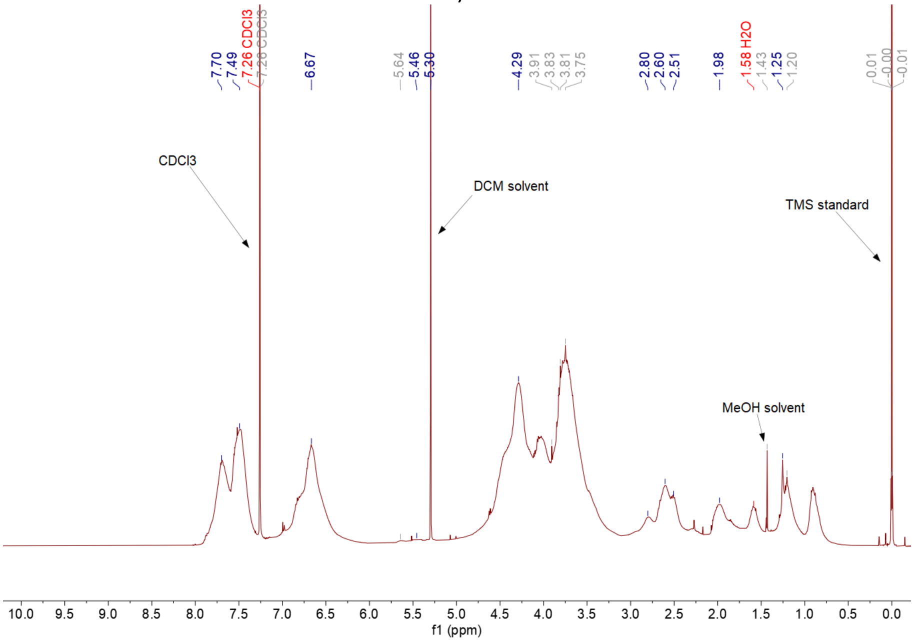

Figure S61. ^{1}H NMR spectrum of the isolated polymer obtained from the PA / LHOGE ROCOP (*Table S8*, entry 2).

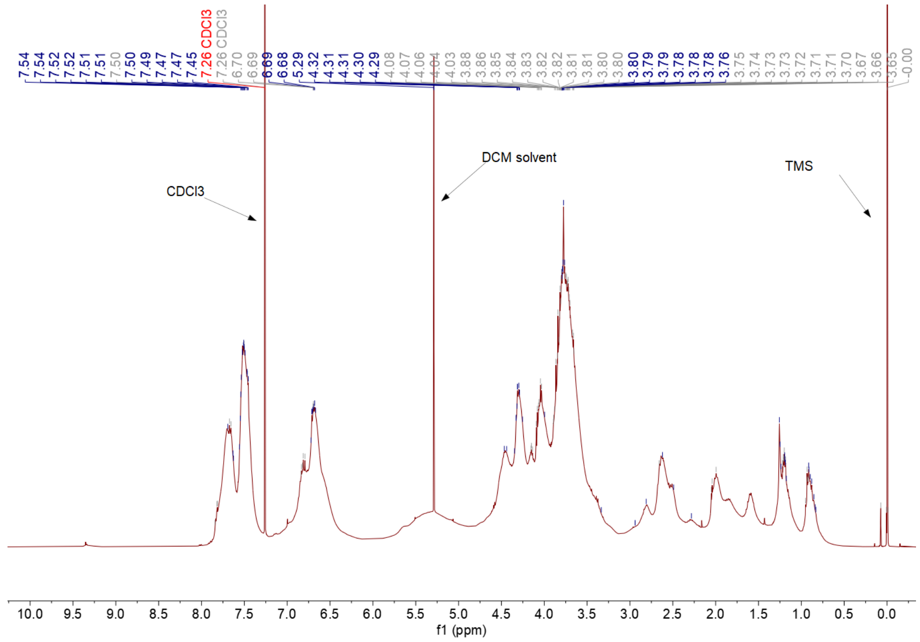

Figure S62. ^{1}H NMR spectrum of the isolated polymer obtained from the PA / LHOGE ROCOP (*Table S8*, entry 6).

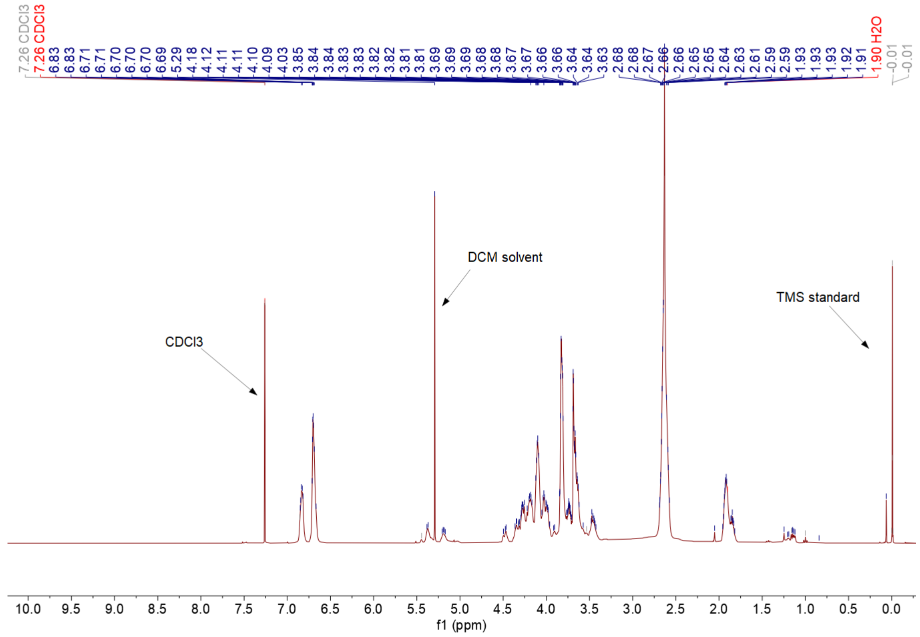

Figure S63. ^{1}H NMR spectrum of the isolated polymer obtained from the SA / DCAGE ROCOP (*Table S9*, entry 1).

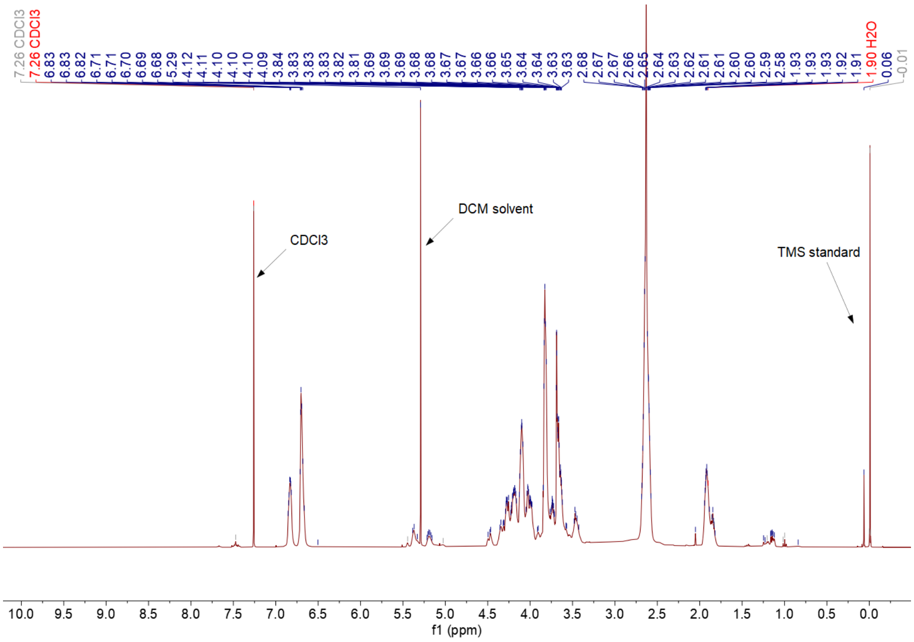

Figure S64. ^{1}H NMR spectrum of the isolated polymer obtained from the SA / DCAGE ROCOP (*Table S9*, entry 2).

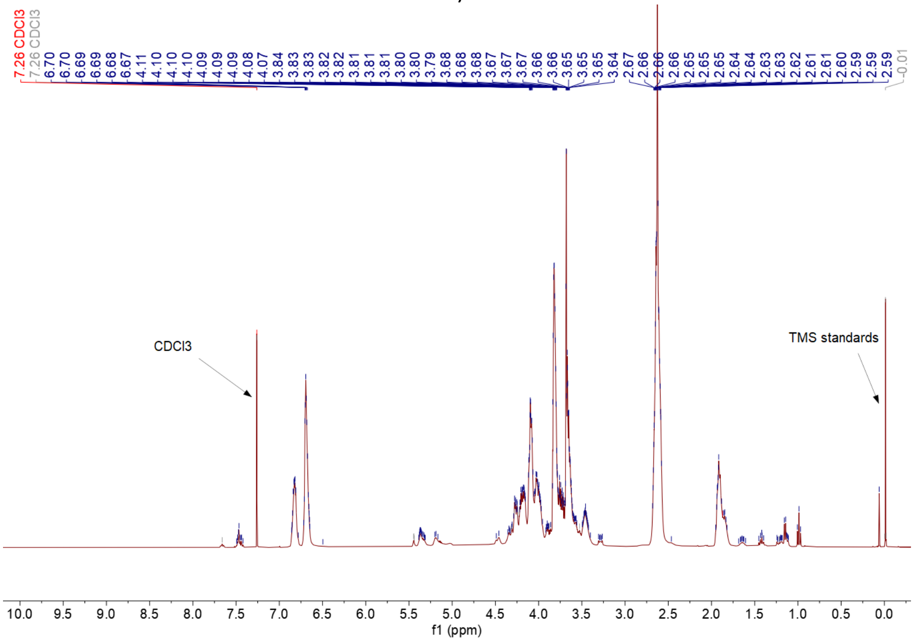

Figure S65. ^{1}H NMR spectrum of the isolated polymer obtained from the SA / DCAGE ROCOP (*Table S9*, entry 3).

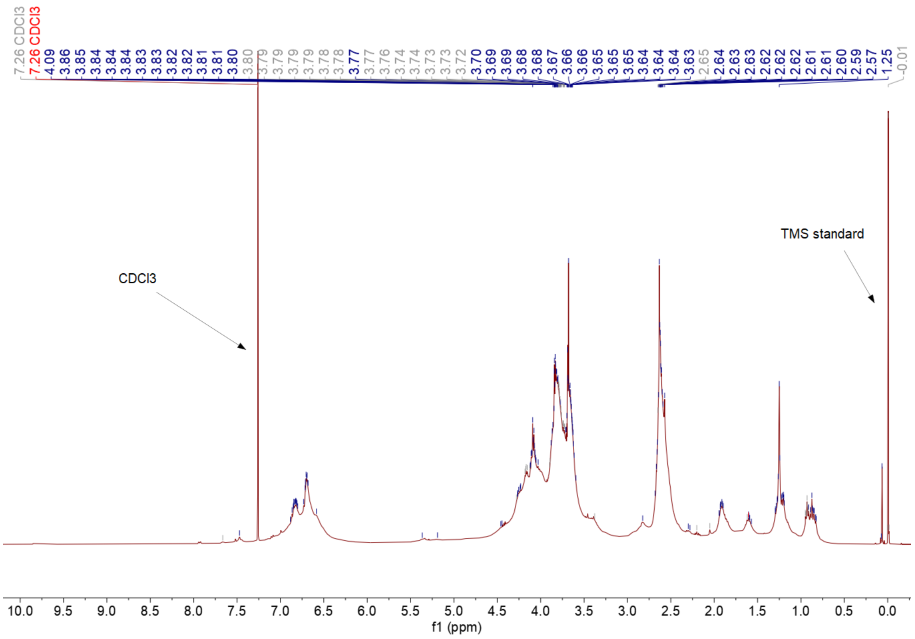

Figure S66. ^{1}H NMR spectrum of the isolated polymer obtained from the SA / LHOGE ROCOP (*Table S9*, entry 7).

S3.1.2. Phosphitylation and quantitative ^{31}P NMR spectra (*Figures 67–74*)

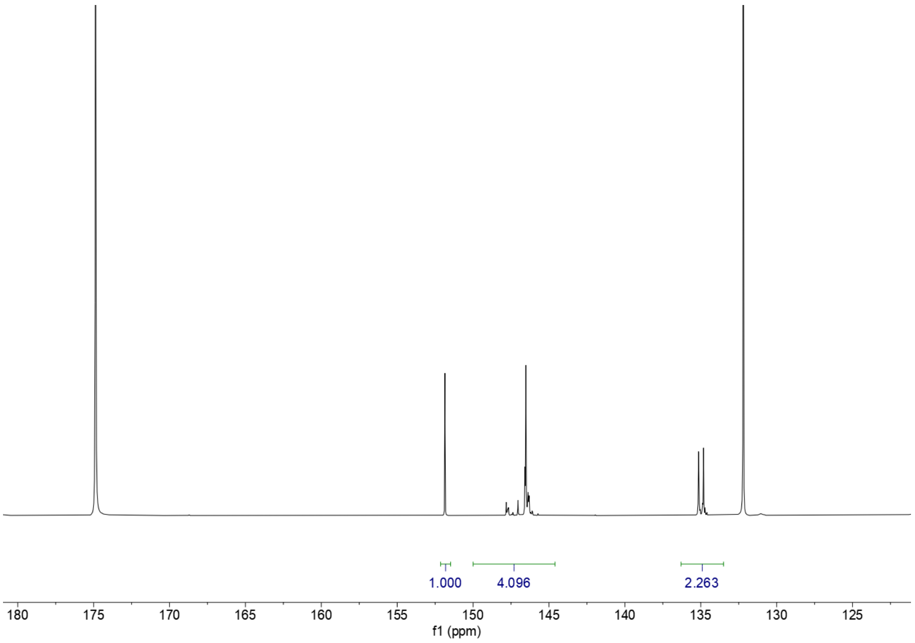

Figure S67. ^{31}P NMR spectrum of phosphitylated poly(PA–*co*–DCAGE) polyester (*Table 2*, entry 7).

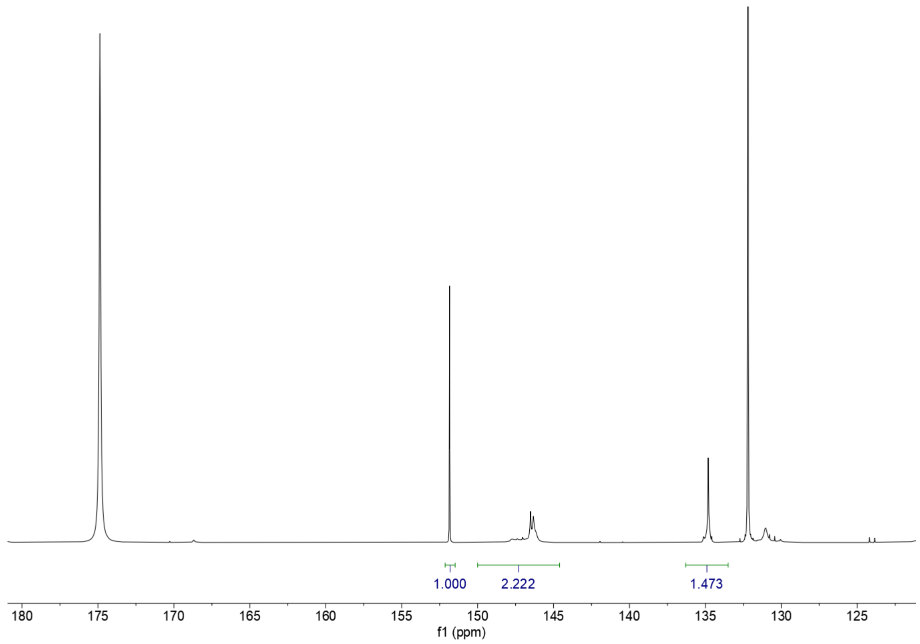

Figure S68. ^{31}P NMR spectrum of phosphitylated poly(PA–*co*–LHOGE) polyester (*Table S8*, entry 1).

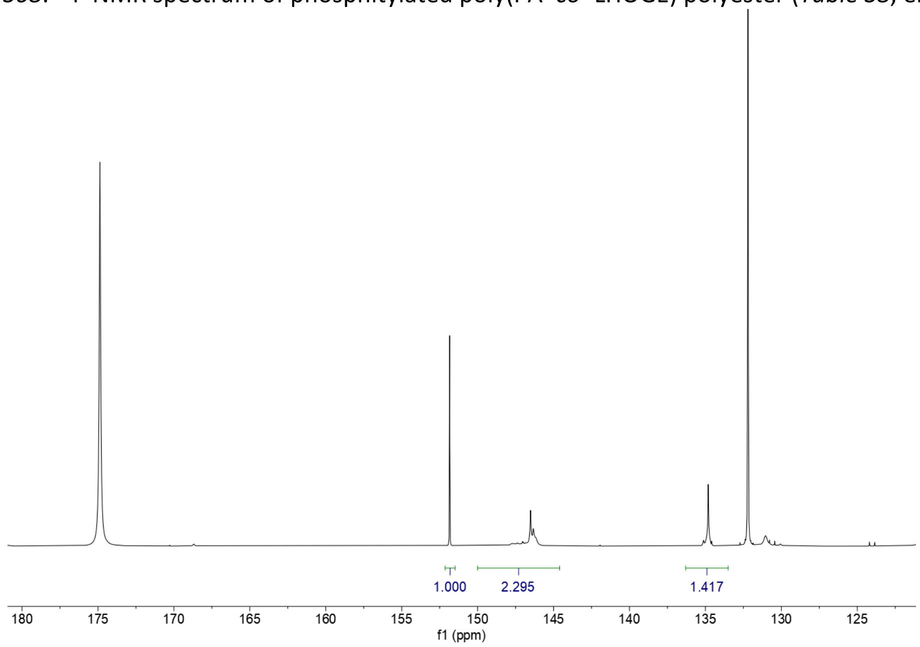

Figure S69. ^{31}P NMR spectrum of phosphitylated poly(PA–*co*–LHOGE) polyester (*Table S8*, entry 2).

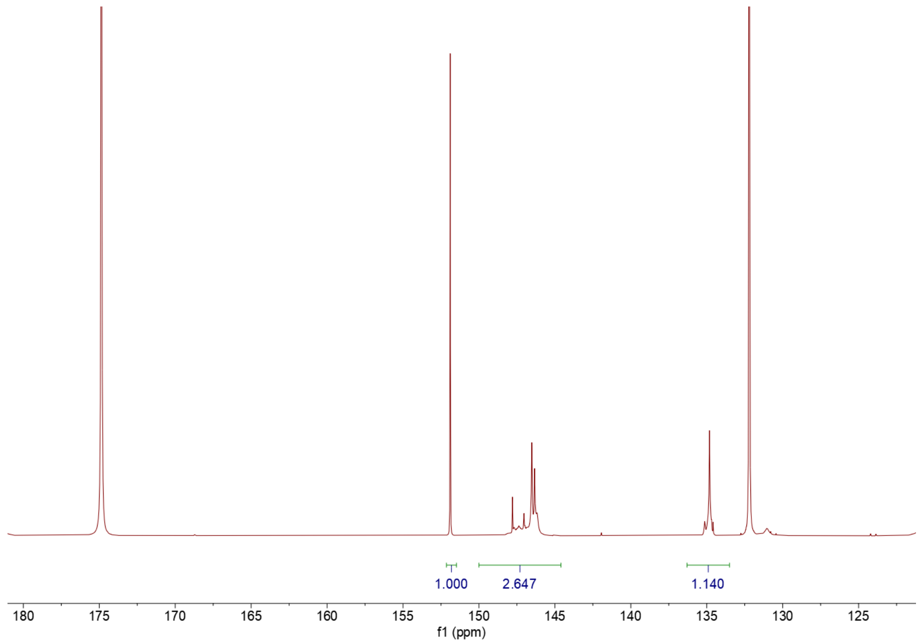

Figure S70. ^{31}P NMR spectrum of phosphitylated poly(PA–*co*–LHOGE) polyester (*Table S8*, entry 6).

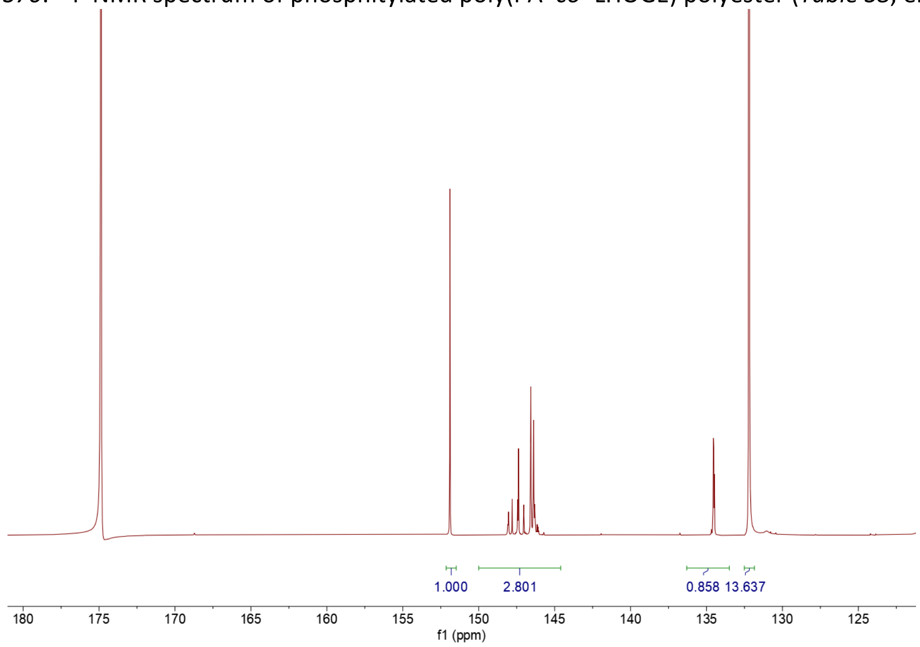

Figure S71. ^{31}P NMR spectrum of phosphitylated poly(SA–*co*–DCAGE) polyester (*Table S9*, entry 3).

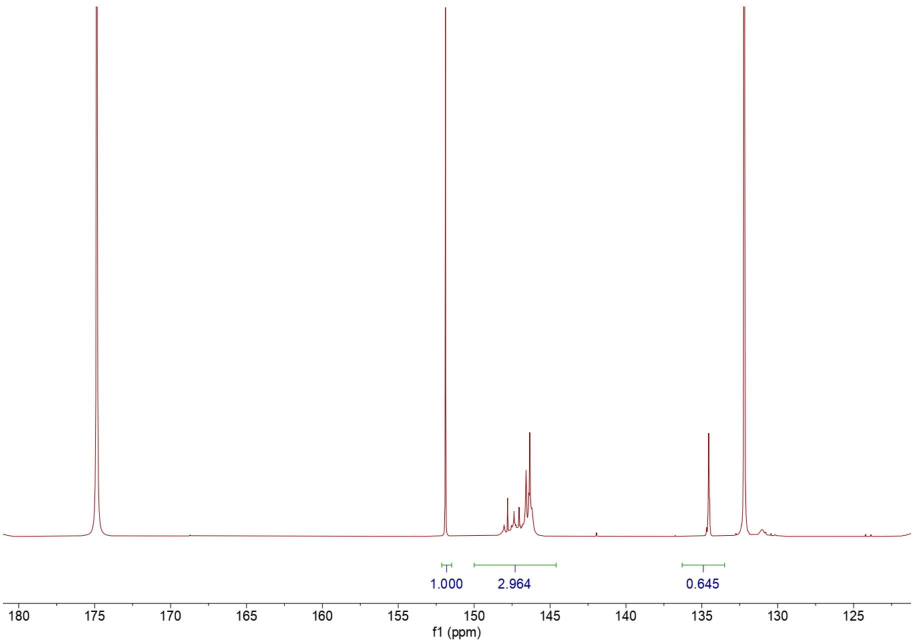

Figure S72. ^{31}P NMR spectrum of phosphitylated poly(SA–*co*–LHOGE) polyester (*Table S9*, entry 7).

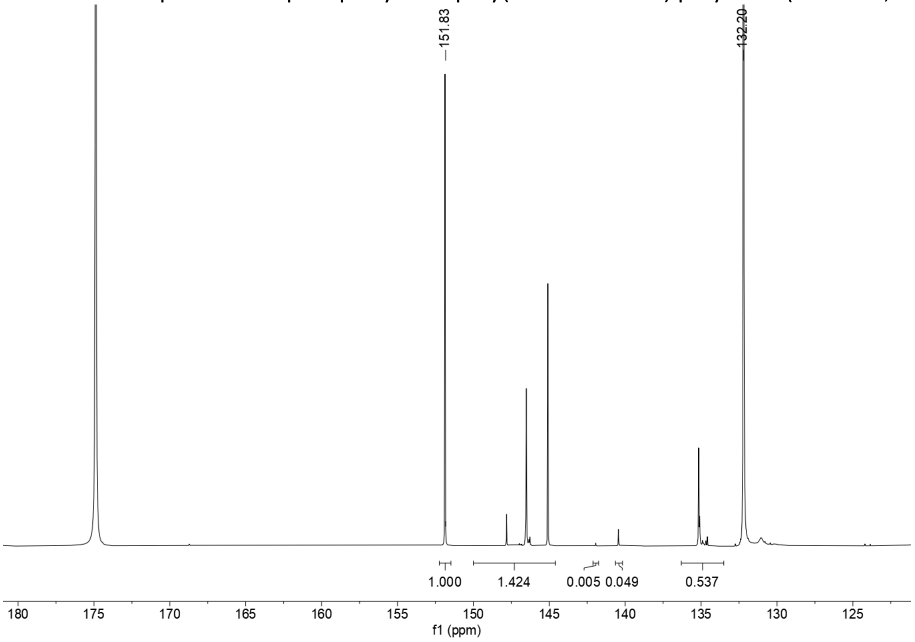

Figure S73. ^{31}P NMR spectrum of the phosphitylated crude product mixture obtained from spiking 25% [DCAGE] into the PA / PGE ROCOP using no PPNCl organic catalyst (*Table S6*).

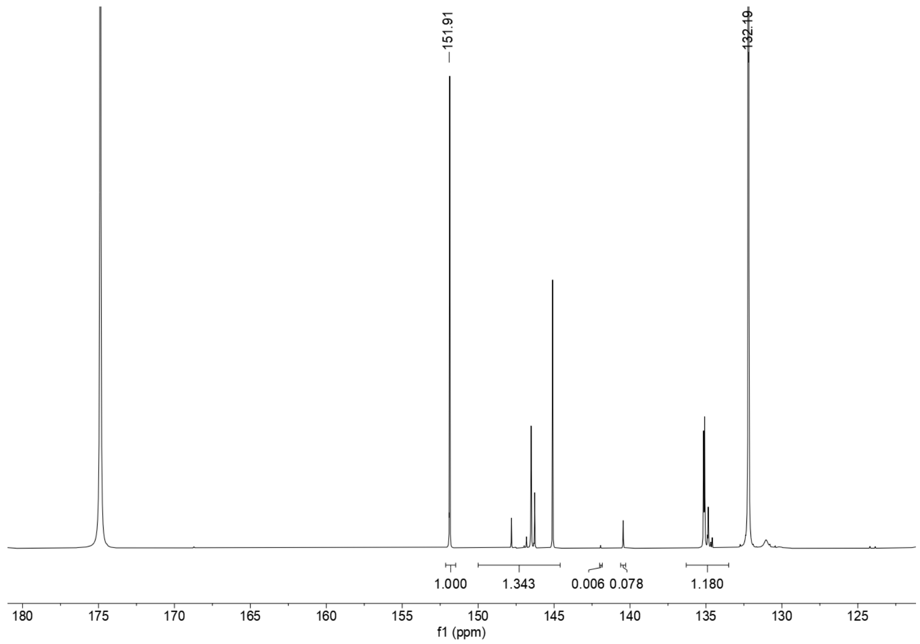

Figure S74. ^{31}P NMR spectrum of the phosphitylated crude product mixture obtained from spiking 5% [DCAGE] into the PA / PGE ROCOP using no PPNCl organic catalyst (*Table S6*).

S3.1.3. FT–IR spectra of lignin, lignin-derived polyesters, and polyurethane film materials (*Figures S75–S93*)

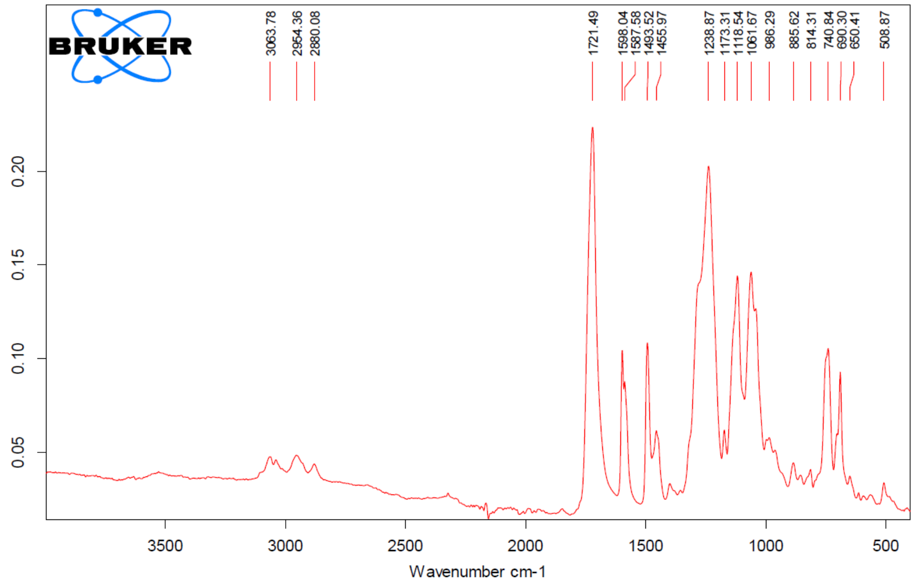

Figure S75. FT–IR spectrum of the isolated polymer obtained from the PA / PGE ROCOP using PPNCl organic catalyst (*Table 1*, entry 1).

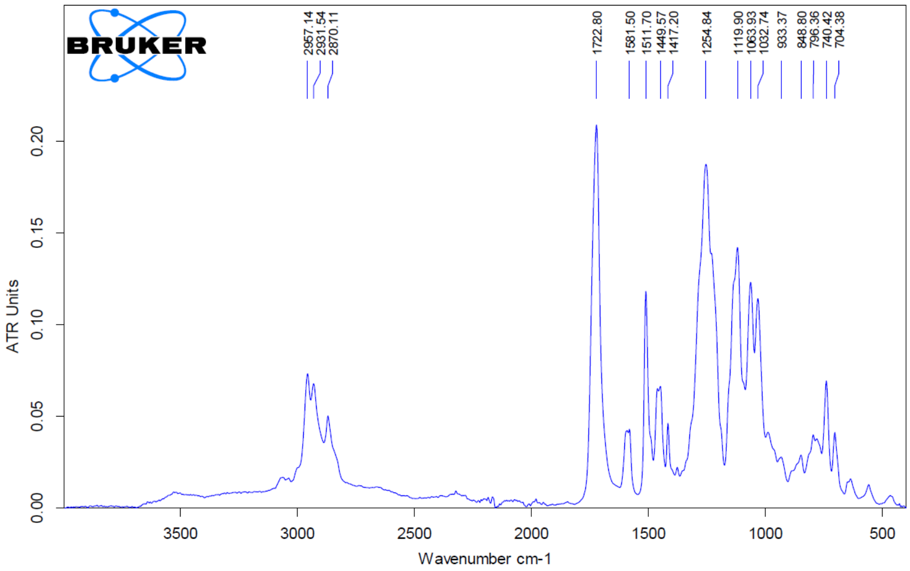

Figure S76. FT–IR spectrum of the isolated polymer obtained from the PA / PGGE ROCOP using PPNCl organic catalyst (*Table 1*, entry 2).

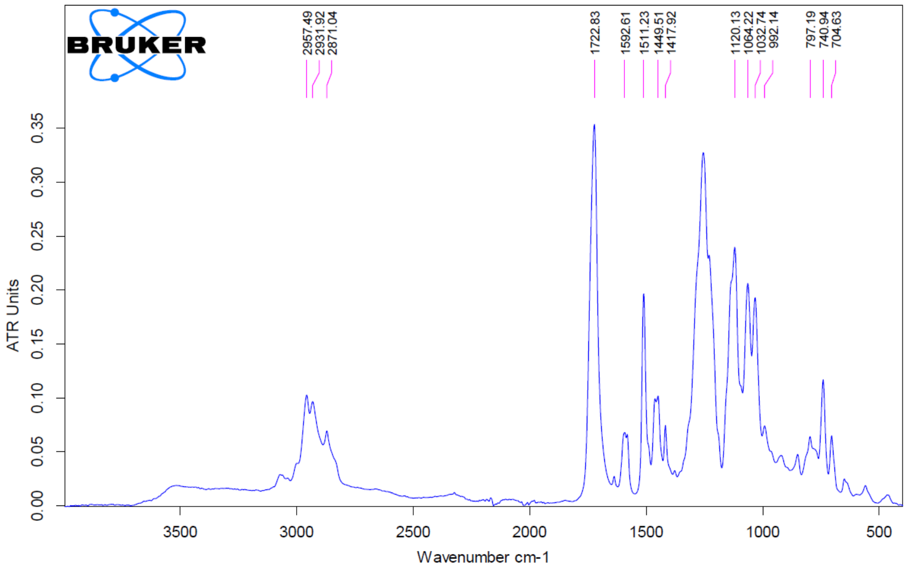

Figure S77. FT–IR spectrum of the isolated polymer obtained from the PA / EGE ROCOP using PPNCl organic catalyst (*Table 1*, entry 3).

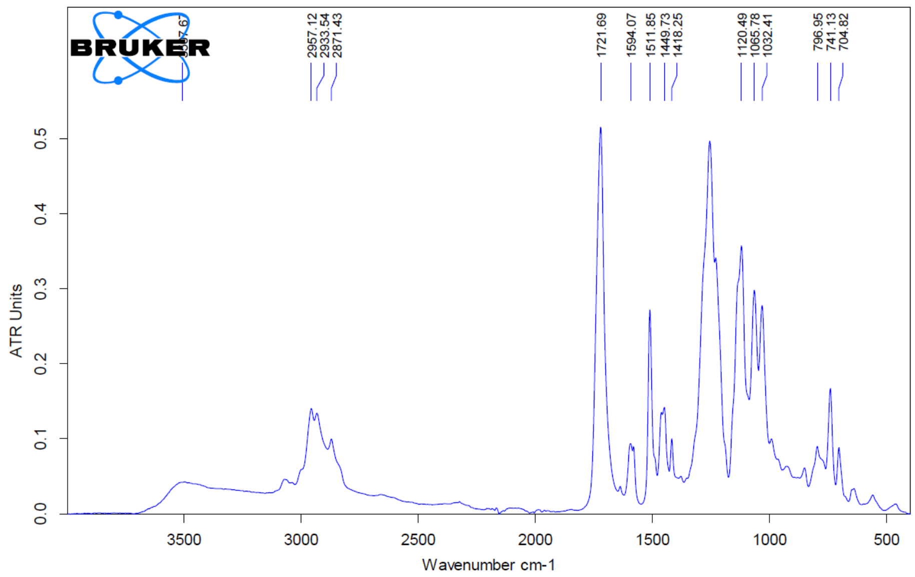

Figure S78. FT–IR spectrum of the isolated polymer obtained from the PA / DCAGE ROCOP using PPNCl organic catalyst (*Table 1*, entry 4).

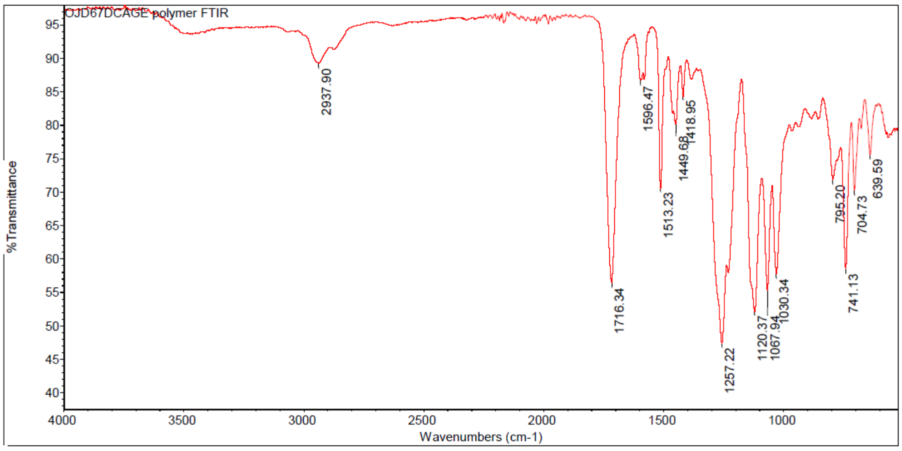

Figure S79. FT–IR spectrum of the isolated polymer obtained from the PA / DCAGE ROCOP using PPNCl organic catalyst (*Table 2*, entry 4).

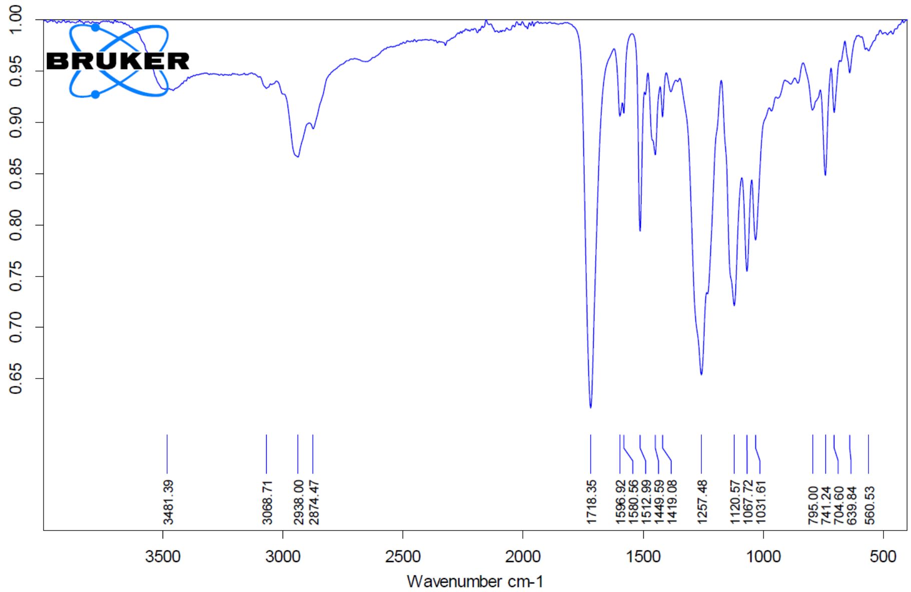

Figure S80. FT–IR spectrum of the isolated polymer obtained from the PA / DCAGE ROCOP using PPNCl organic catalyst (*Table 2*, entry 6).

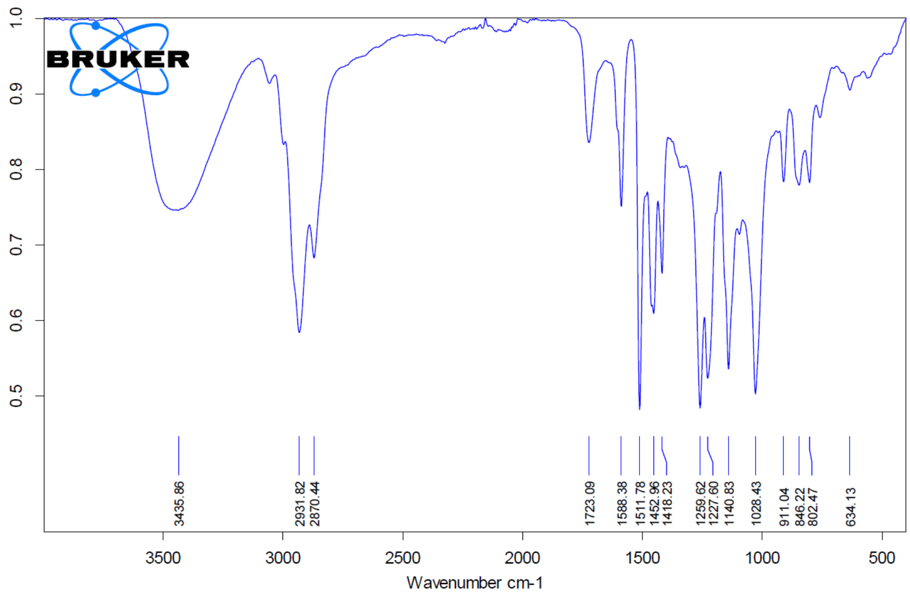

Figure S81. FT–IR spectrum of the LHOGE monomer used for ROCOP.

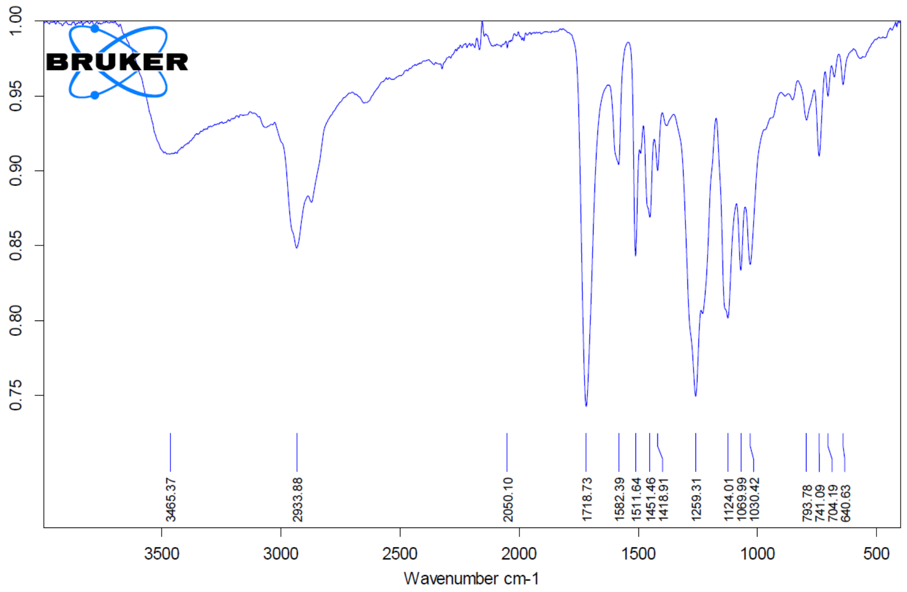

Figure S82. FT–IR spectrum of the isolated polymer obtained from the PA / LHOGE ROCOP without using PPNCl organic catalyst (*Table S8*, entry 1).

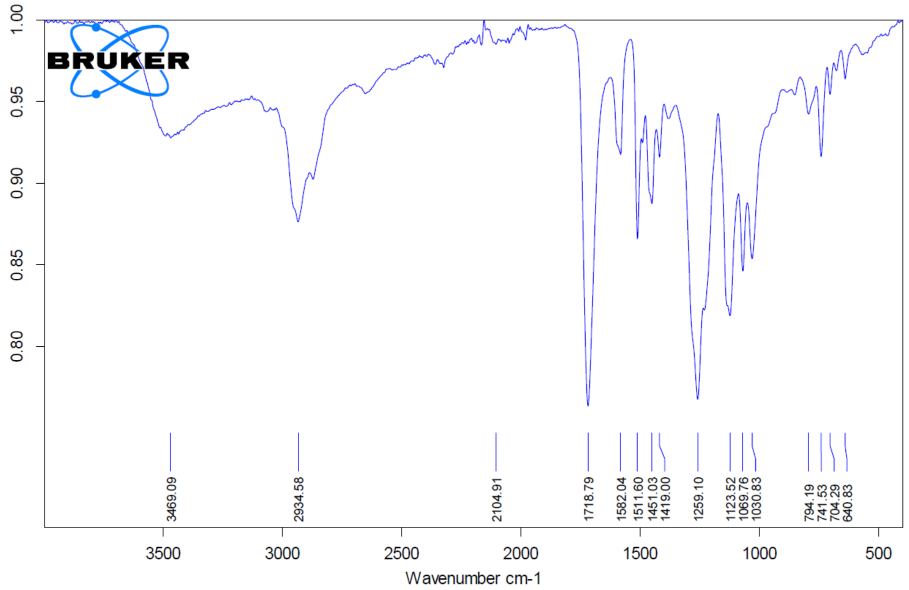

Figure S83. FT–IR spectrum of the isolated polymer obtained from the PA / LHOGE ROCOP using PPNCl organic catalyst (*Table S8*, entry 2).

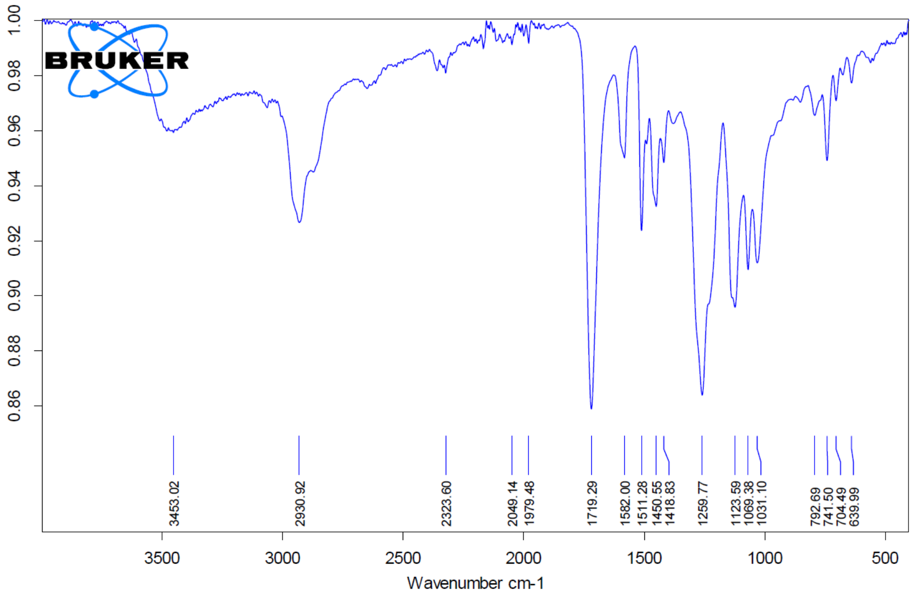

Figure S84. FT–IR spectrum of the isolated polymer obtained from the PA / LHOGE ROCOP using PPNCl organic catalyst (*Table S8*, entry 6).

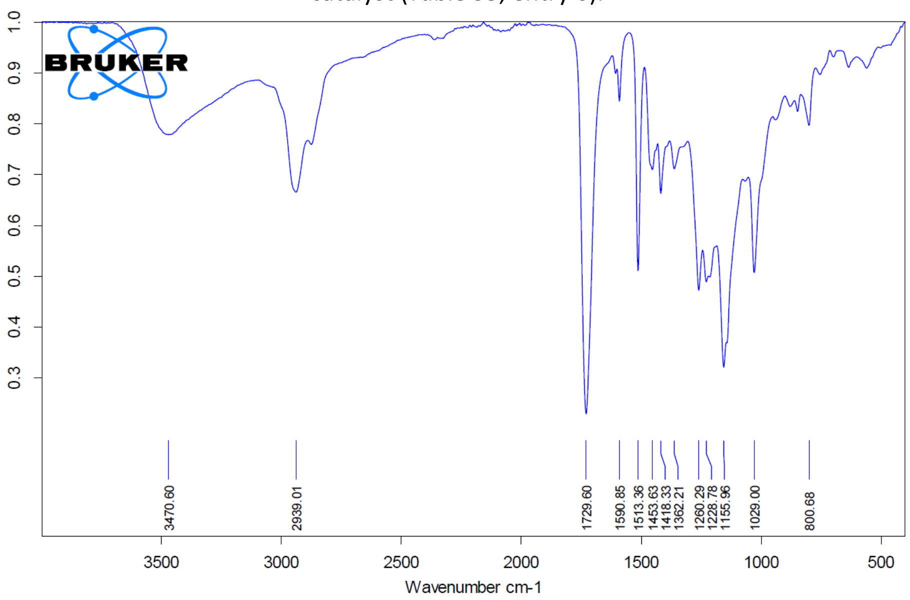

Figure S85. FT–IR spectrum of the isolated polymer obtained from the SA / DCAGE ROCOP using PPNCl organic catalyst (*Table S9*, entry 3).

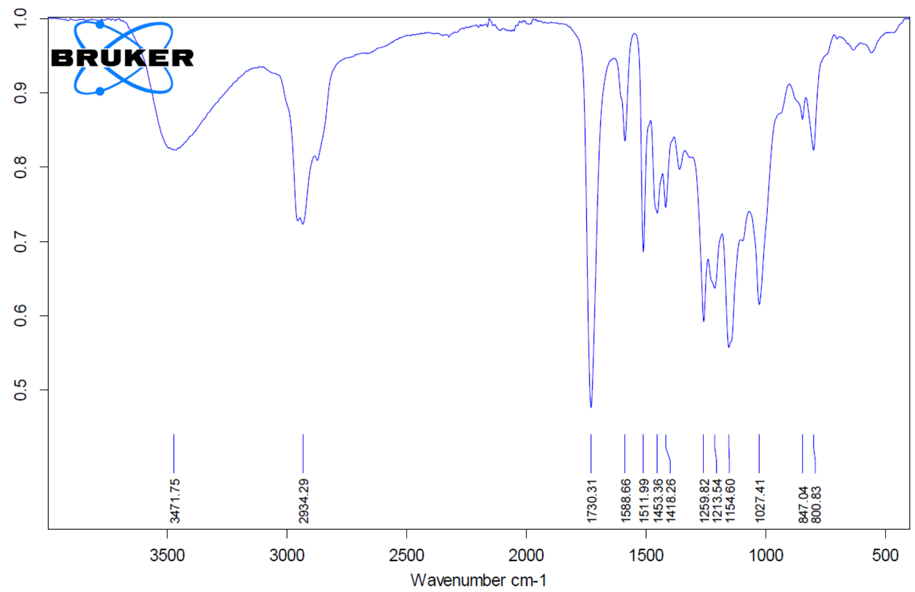

Figure S86. FT–IR spectrum of the isolated polymer obtained from the SA / LHOGE ROCOP without PPNCl organic catalyst (*Table S9*, entry 4).

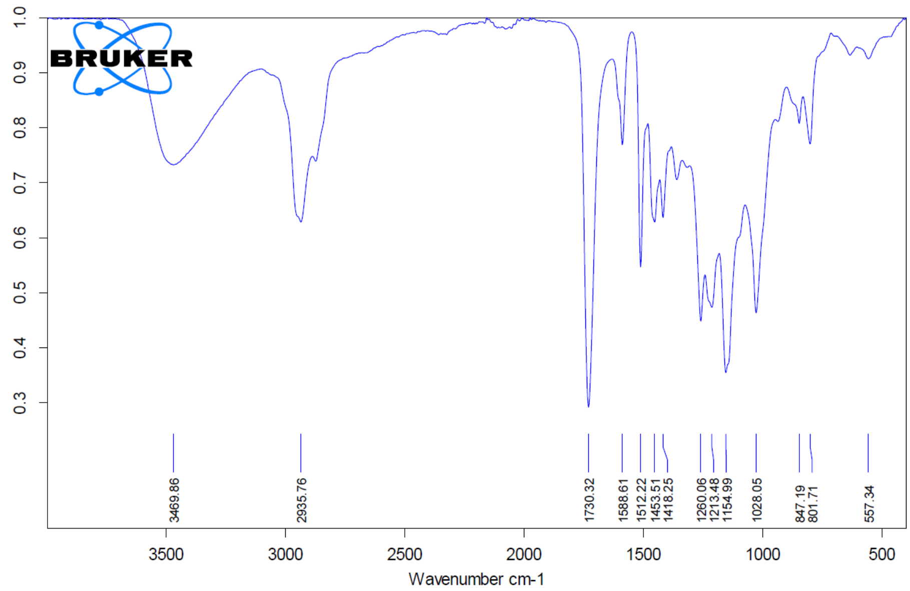

Figure S87. FT–IR spectrum of the isolated polymer obtained from the SA / LHOGE ROCOP using PPNCl organic catalyst (*Table S9*, entry 5).

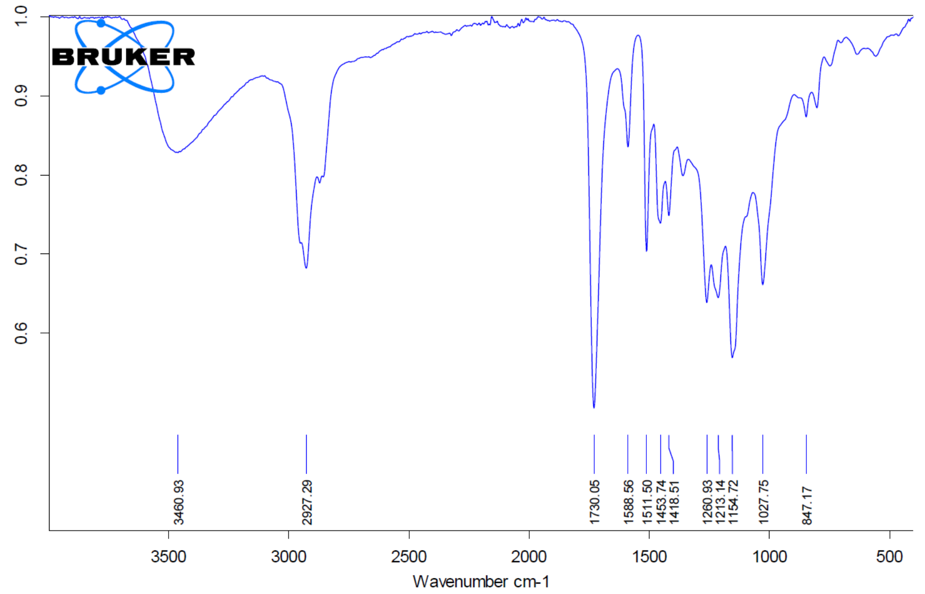

Figure S88. FT–IR spectrum of the isolated polymer obtained from the SA / LHOGE ROCOP using PPNCl organic catalyst (*Table S9*, entry 7).

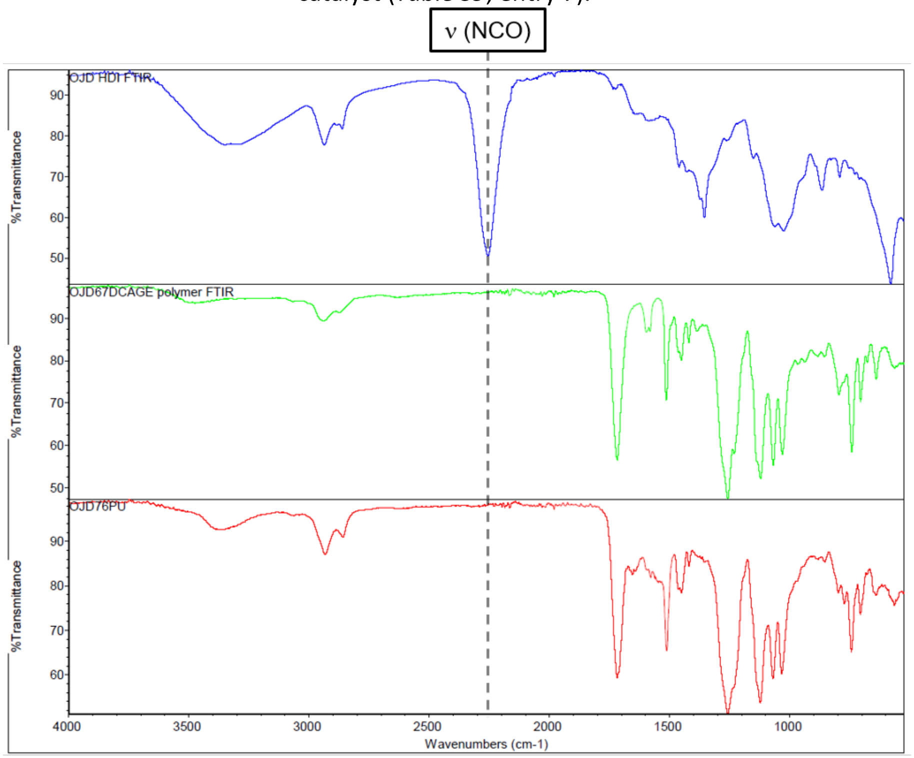

Figure S89. Stacked FT–IR spectra of the PU thin film product (labelled 'OJD76PU'), compared to the HDI and DCAGE polyol (labelled 'OJD67DCAGE', *Table 2*, entry 4) reagents; the disappearance of the NCO stretch (ν) in the PU product can be observed that indicated complete reactivity and conversion to the desired thermoset material.

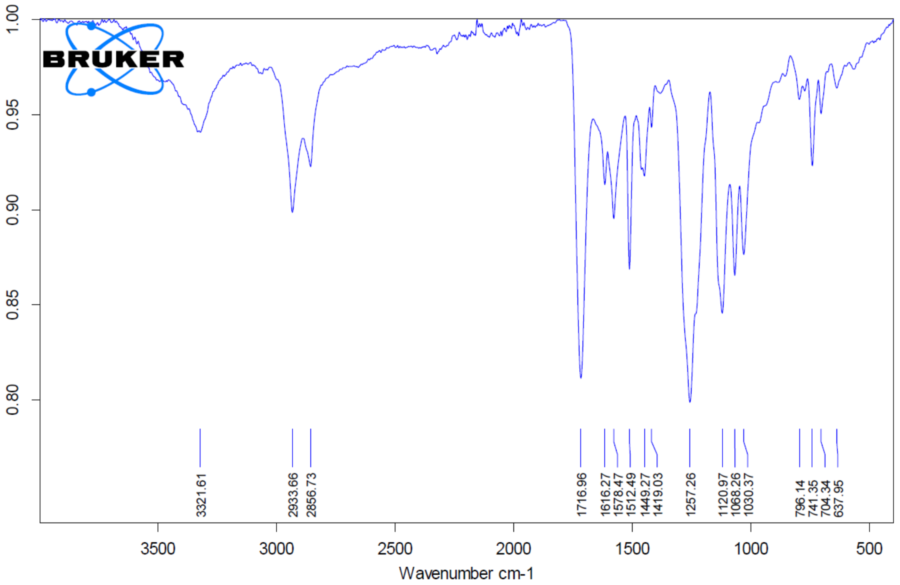

Figure S90. FT–IR spectrum of the isolated PU film product obtained from using poly(PA–*co*–DCAGE) (*Table 2*, entry 2).

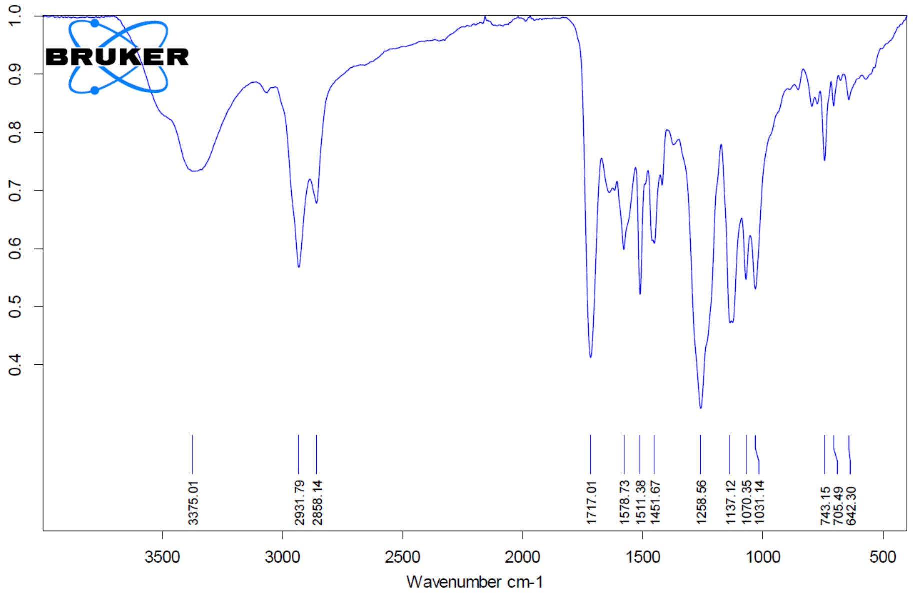

Figure S91. FT–IR spectrum of the isolated PU film product obtained from using poly(PA–*co*–LHOGE) (*Table S8*, entry 6).

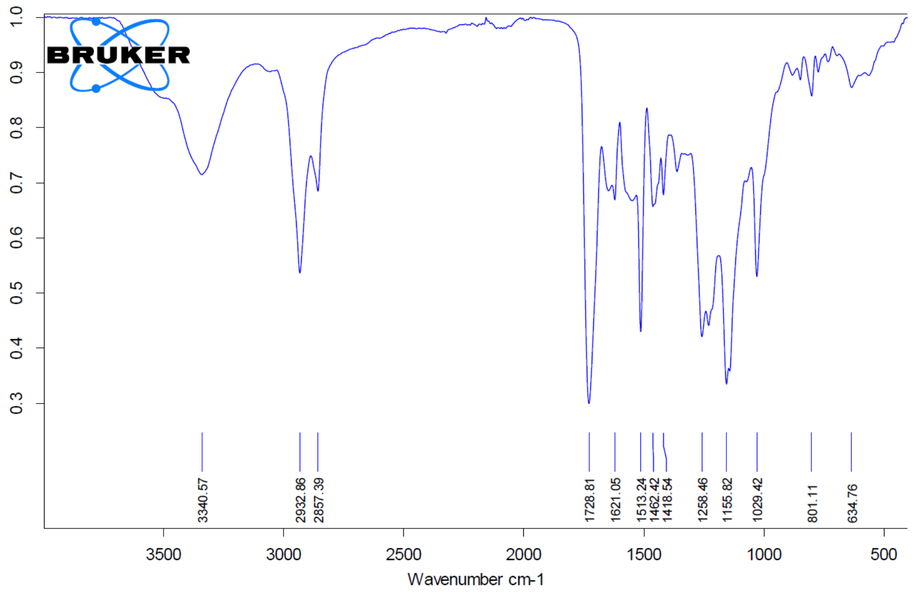

Figure S92. FT–IR spectrum of the isolated PU film product obtained from using poly(SA–*co*–DCAGE) (*Table S9*, entry 3).

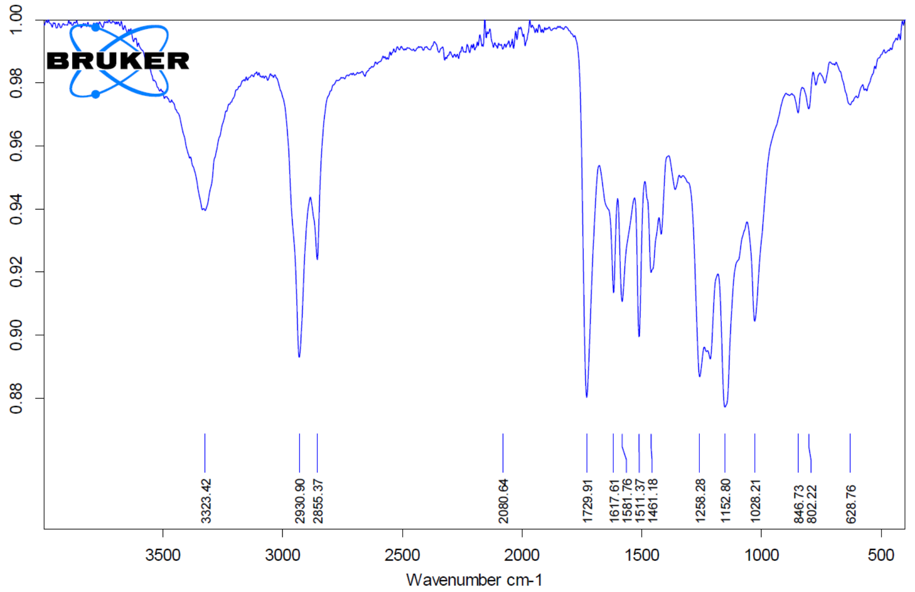

Figure S93. FT–IR spectrum of the isolated PU film product obtained from using poly(SA–*co*–LHOGE) (*Table S9*, entry 7).

S3.1.4. DSC traces of polyesters and PU films (*Figures S94–S111*)

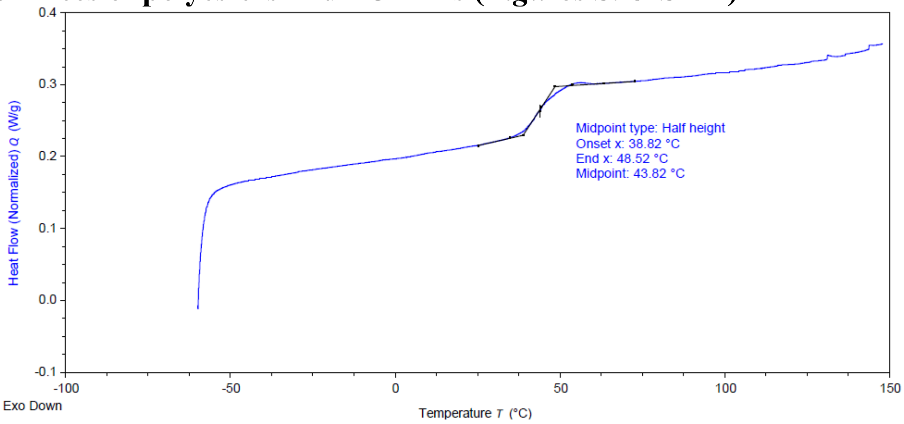

Figure S94. DSC 2nd heating cycle curve of the isolated polymer attained from the PA / PGE ROCOP (*Table 1*, entry 1).

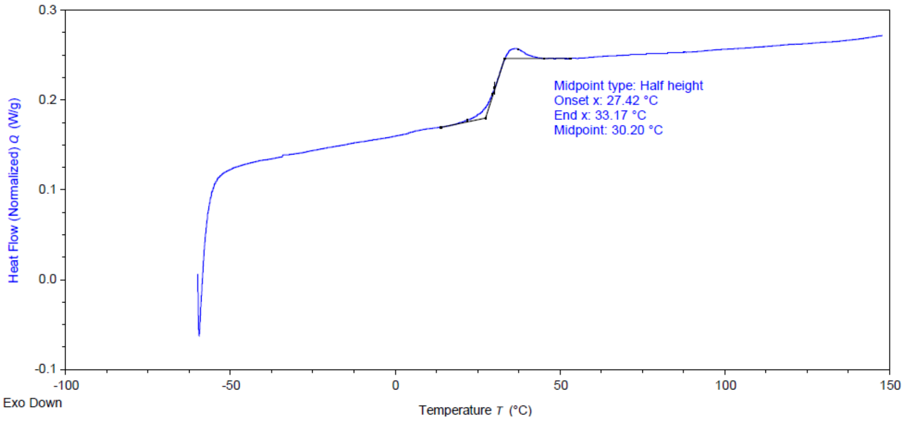

Figure S95. DSC 2nd heating cycle curve of the isolated polymer attained from the PA / PGGE ROCOP (*Table 1*, entry 2).

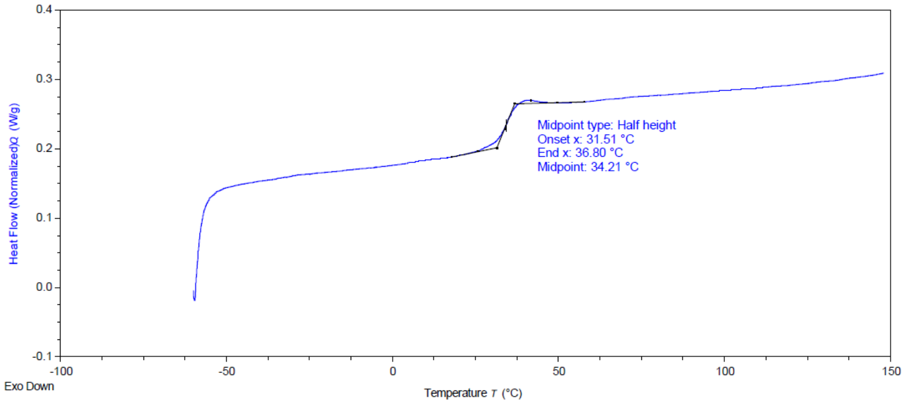

Figure S96. DSC 2nd heating cycle curve of the isolated polymer attained from the PA / EGE ROCOP (*Table 1* entry 3).

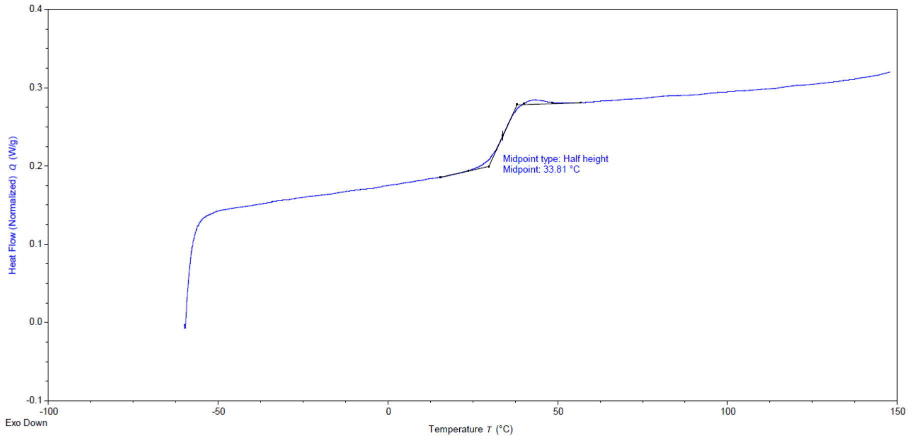

Figure S97. DSC 2nd heating cycle curve of the isolated polymer attained from PA / DCAGE ROCOP (*Table 1*, entry 4).

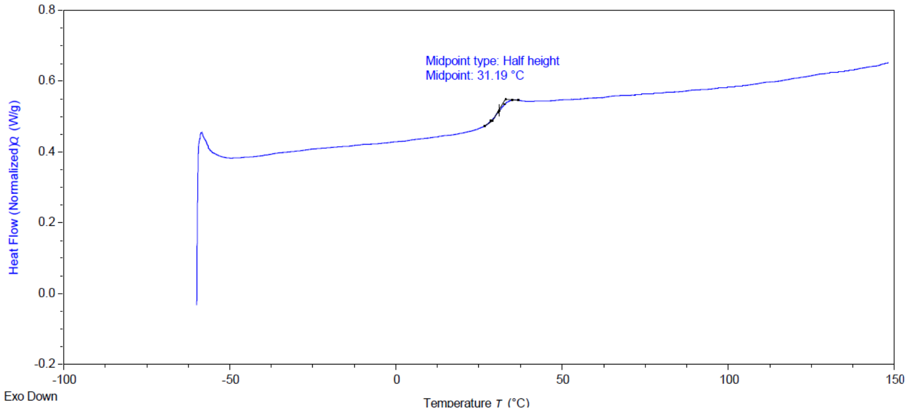

Figure S98. DSC 2nd heating cycle curve of the isolated polymer attained from PA / DCAGE ROCOP (*Table 2*, entry 4).

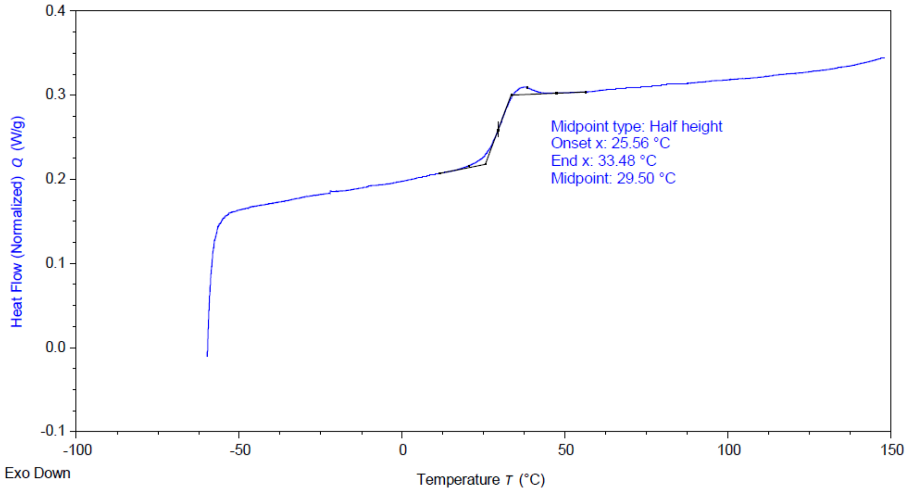

Figure S99. DSC 2nd heating cycle curve of the isolated polymer attained from PA / DCAGE ROCOP (*Table 2*, entry 6).

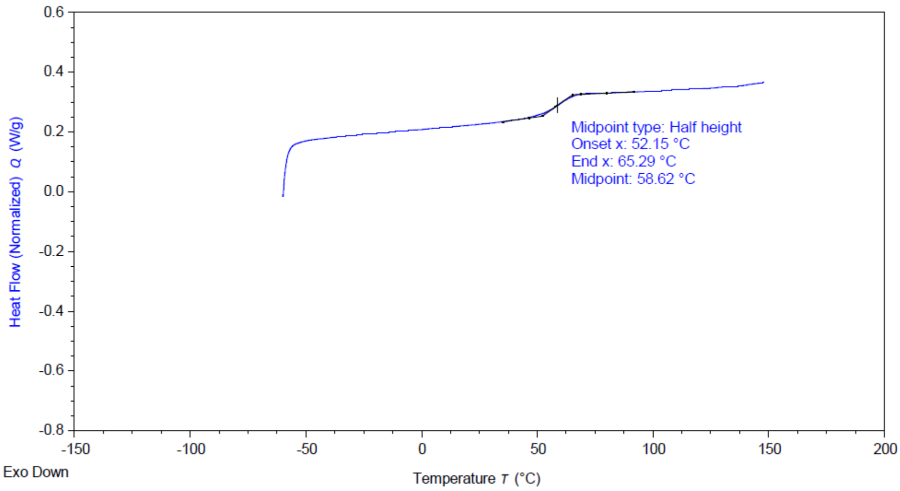

Figure S100. DSC 2nd heating cycle curve of the isolated polymer attained from PA / LHOGE ROCOP (*Table S8*, entry 1).

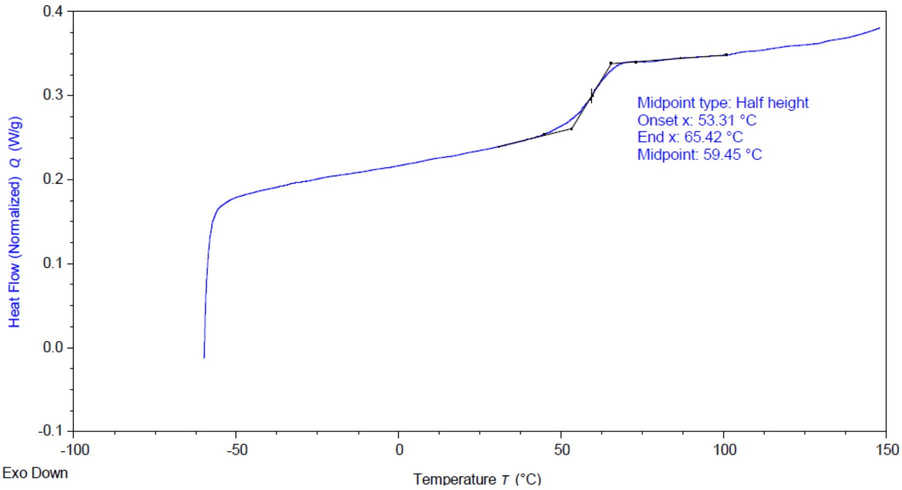

Figure S101. DSC 2nd heating cycle curve of the isolated polymer attained from PA / LHOGE ROCOP (*Table S8*, entry 2).

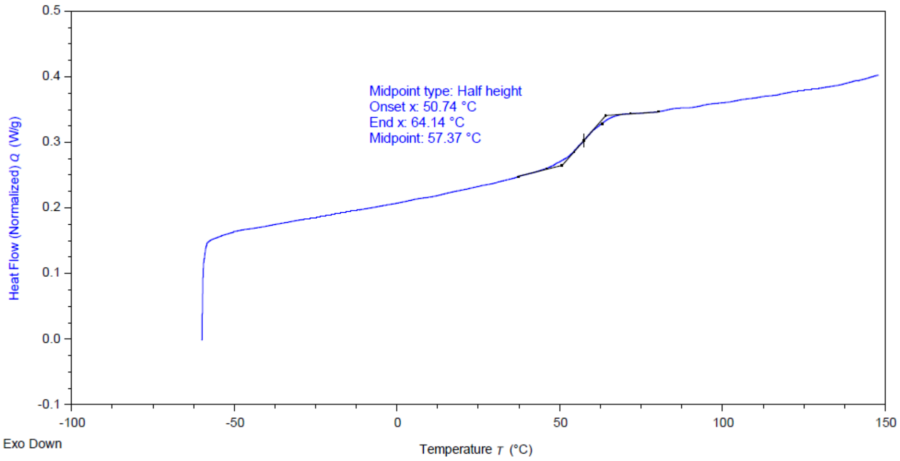

Figure S102. DSC 2nd heating cycle curve of the isolated polymer attained from PA / LHOGE ROCOP (*Table S8*, entry 6).

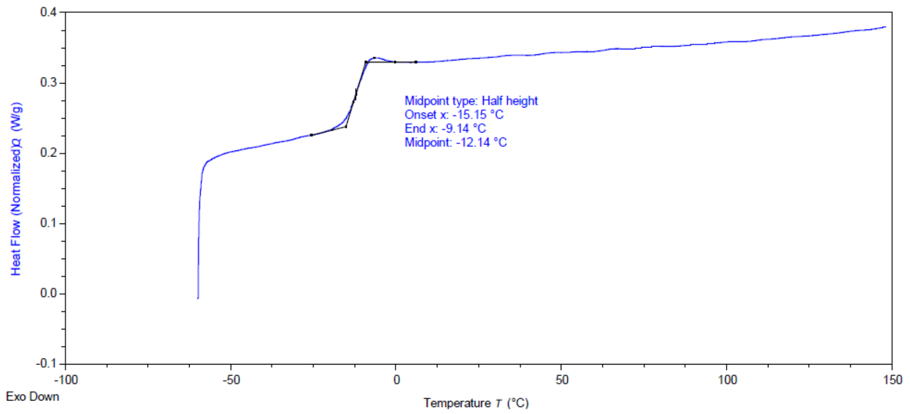

Figure S103. DSC 2nd heating cycle curve of the isolated polymer attained from SA / DCAGE ROCOP (*Table S9*, entry 3).

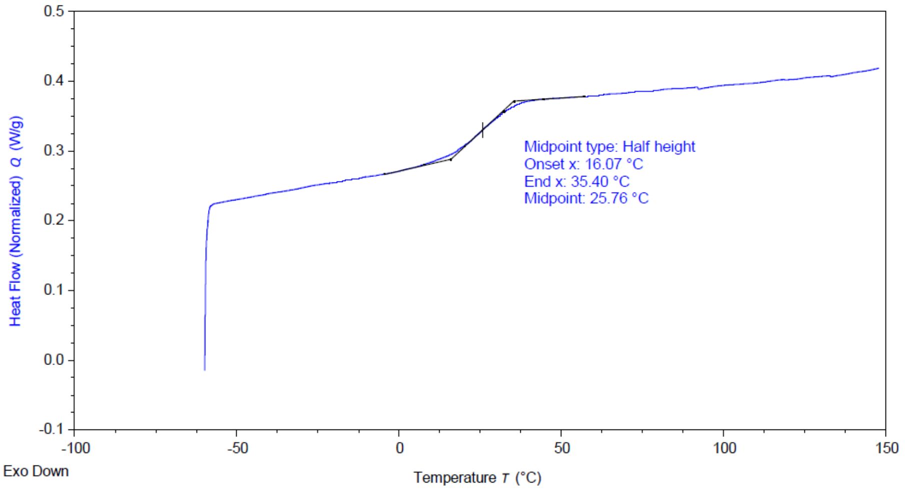

Figure S104. DSC 2nd heating cycle curve of the isolated polymer attained from SA / LHOGE ROCOP (*Table S9*, entry 4).

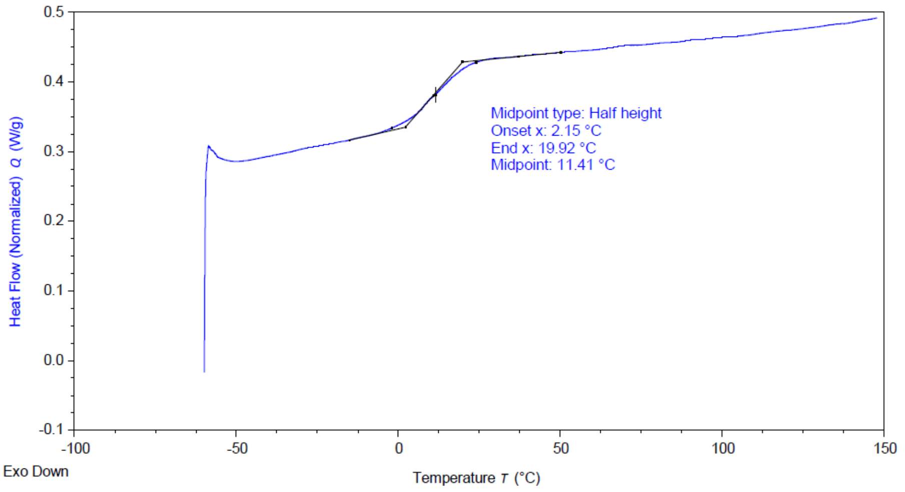

Figure S105. DSC 2nd heating cycle curve of the isolated polymer attained from SA / LHOGE ROCOP (*Table S9*, entry 5).

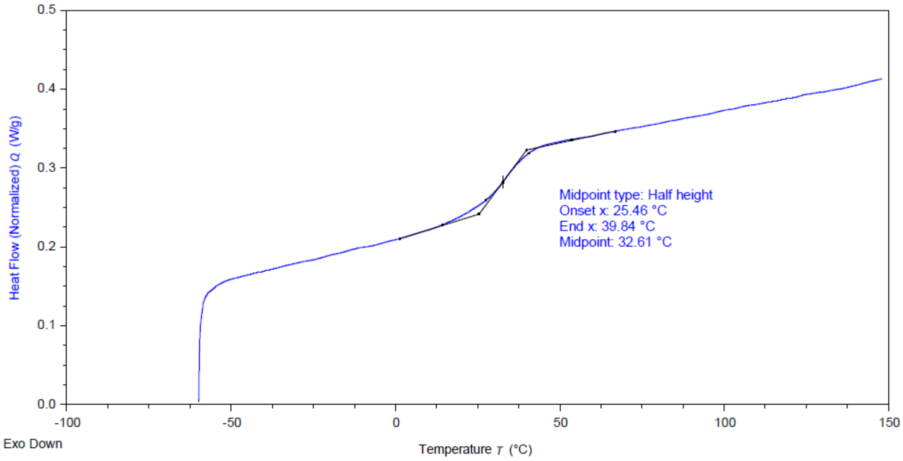

Figure S106. DSC 2nd heating cycle curve of the isolated polymer attained from SA / LHOGE ROCOP (*Table S9*, entry 7).

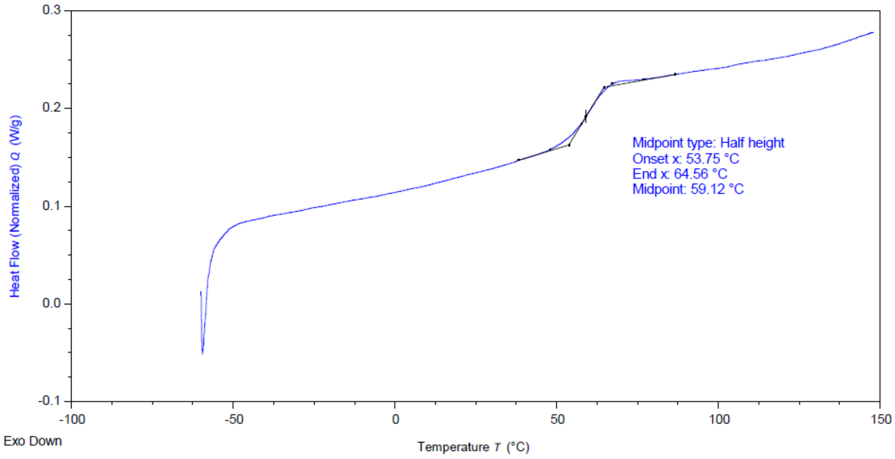

Figure S107. DSC 2nd heating cycle curve of the isolated PU film product obtained from using poly(PA–*co*–DCAGE) (*Table 2*, entry 4).

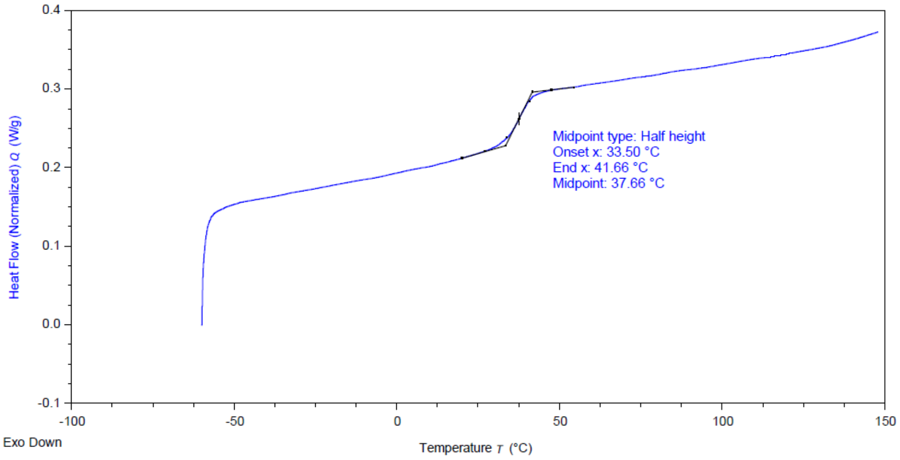

Figure S108. DSC 2nd heating cycle curve of the isolated PU film product obtained from using poly(PA–*co*–DCAGE) (*Table 2*, entry 6).

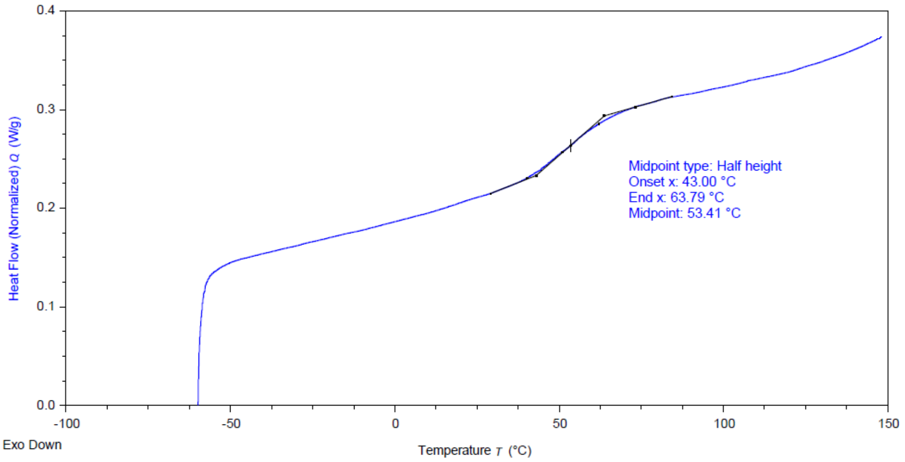

Figure S109. DSC 2nd heating cycle curve of the isolated PU film product obtained from using poly(PA–*co*–LHOGE) (*Table S8*, entry 6).

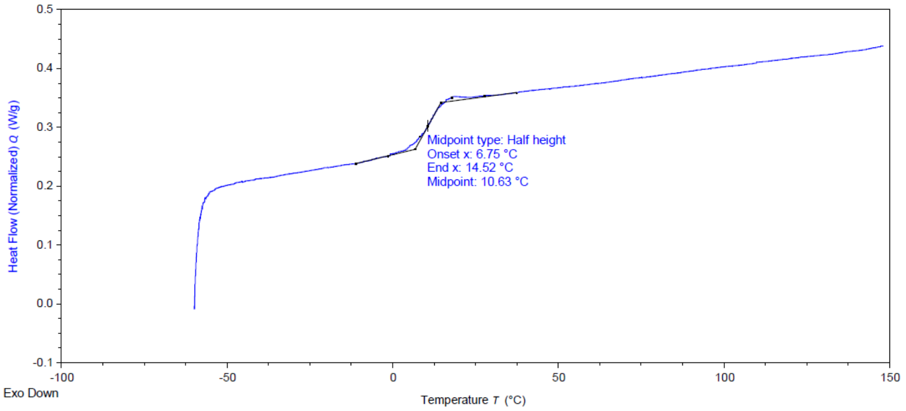

Figure S110. DSC 2nd heating cycle curve of the isolated PU film product obtained from using poly(SA–*co*–DCAGE) (*Table S9*, entry 3).

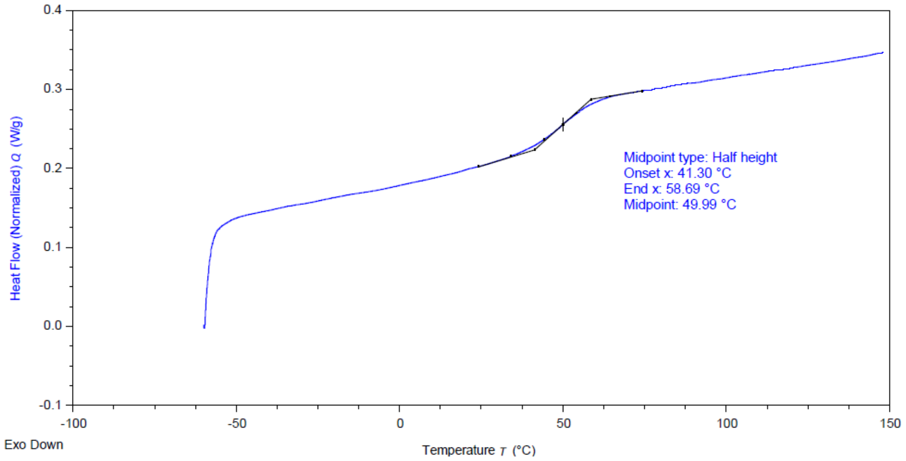

Figure S111 DSC 2nd heating cycle curve of the isolated PU film product obtained from using poly(SA–*co*–LHOGE) (*Table S9*, entry 7).

S3.1.5. GPC chromatograms (*Figures S112–S139*)

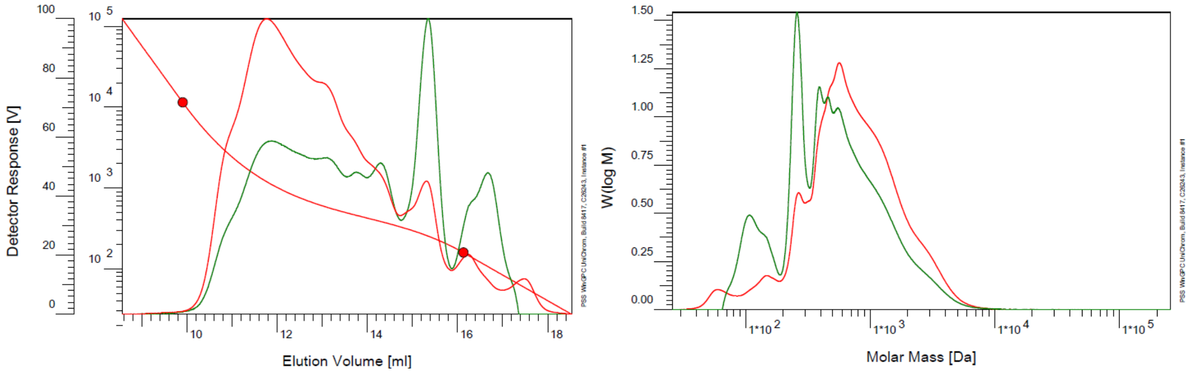

Figure S112. GPC chromatograms of the LHOGE employed for ROCOP (both elution volume and molar mass). See previously reported literature.[7,8,13]

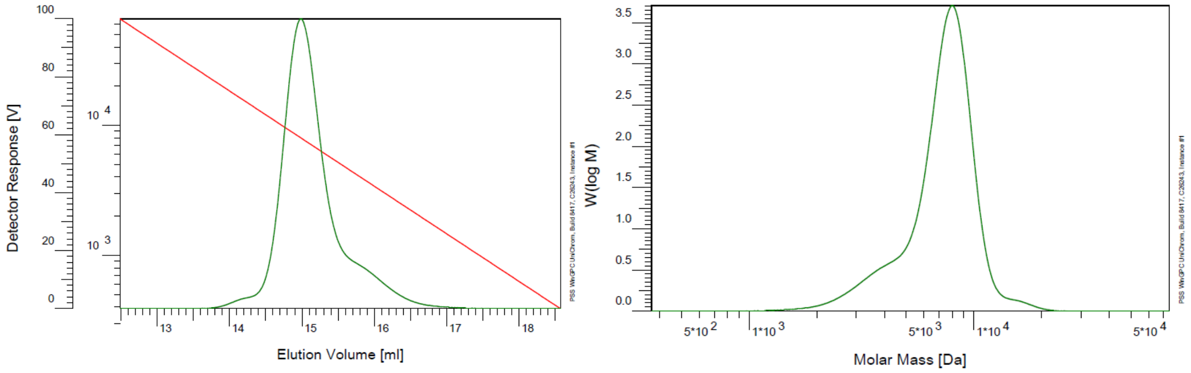

Figure S113. GPC chromatograms (elution volume and molar mass) of the isolated polymer obtained from the PA / PGE ROCOP using PPNCl organic catalyst (*Table 1*, entry 1).

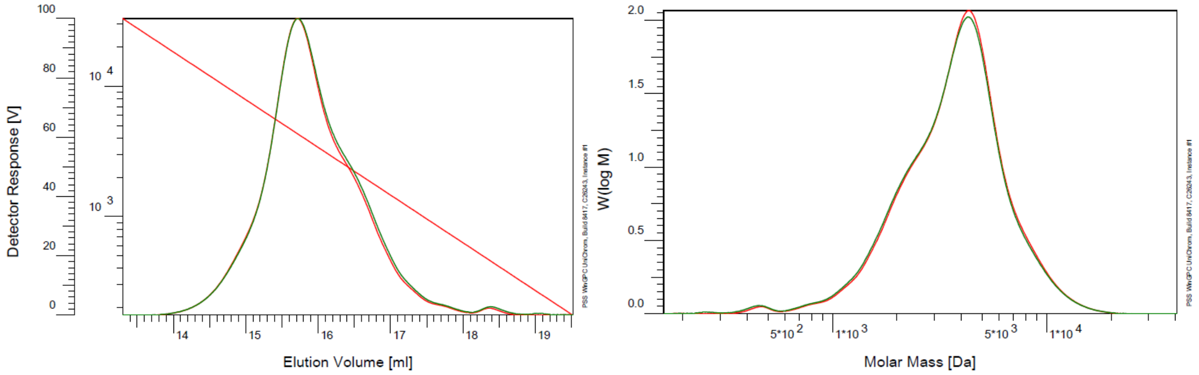

Figure S114. GPC chromatograms (elution volume and molar mass) of the isolated polymer obtained from the PA / PGGE ROCOP using PPNCl organic catalyst (*Table 1*, entry 2).

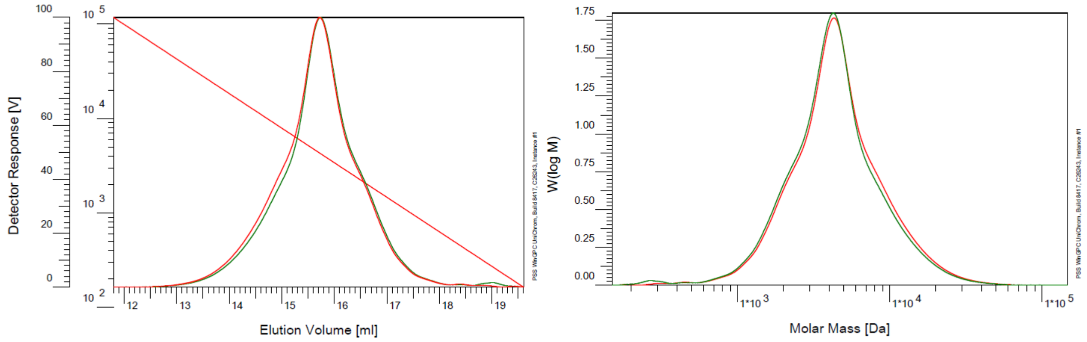

Figure S115. GPC chromatograms (elution volume and molar mass) of the isolated polymer obtained from the PA / EGE ROCOP using PPNCl organic catalyst (*Table 1*, entry 3).

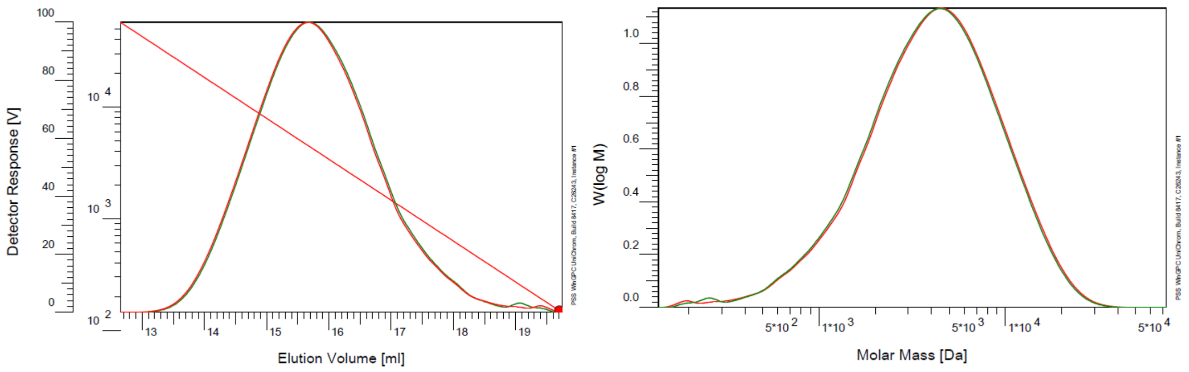

Figure S116. GPC chromatograms (elution volume and molar mass) of the isolated polymer obtained from the PA / DCAGE ROCOP using PPNCl organic catalyst (*Table 1*, entry 4).

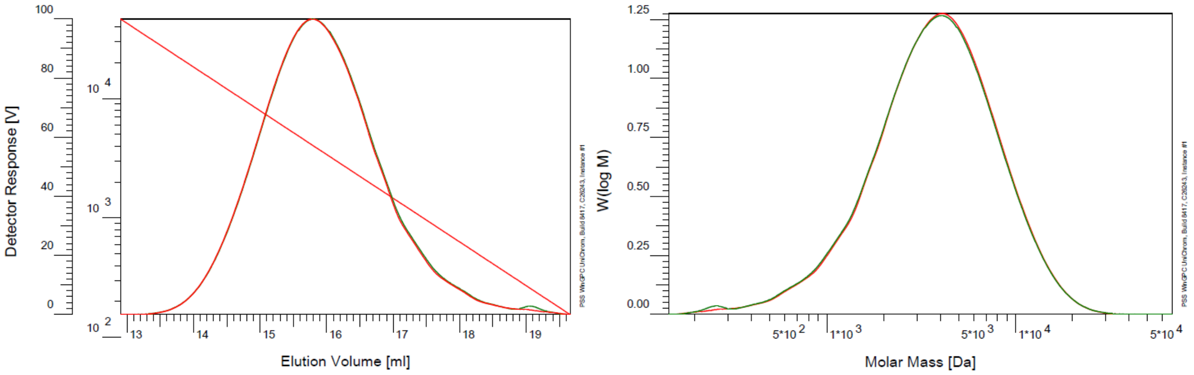

Figure S117. GPC chromatograms (elution volume and molar mass) of the isolated polymer obtained from the PA / DCAGE ROCOP using PPNCl organic catalyst (*Table 2*, entry 3).

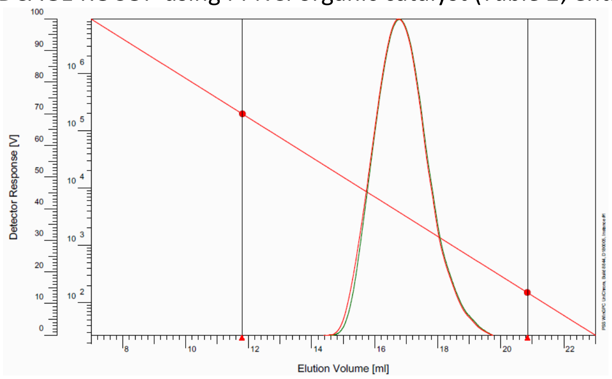

Figure S118. GPC chromatogram of the isolated polymer obtained from the PA / DCAGE ROCOP using PPNCl organic catalyst (*Table 2*, entry 4).

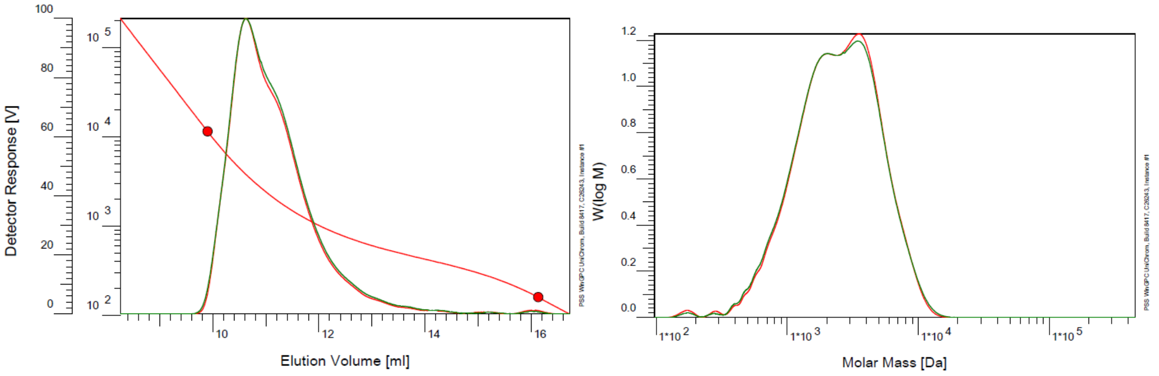

Figure S119. GPC chromatograms (elution volume and molar mass) of the isolated polymer obtained from the PA / DCAGE ROCOP using PPNCl organic catalyst (*Table 2*, entry 6).

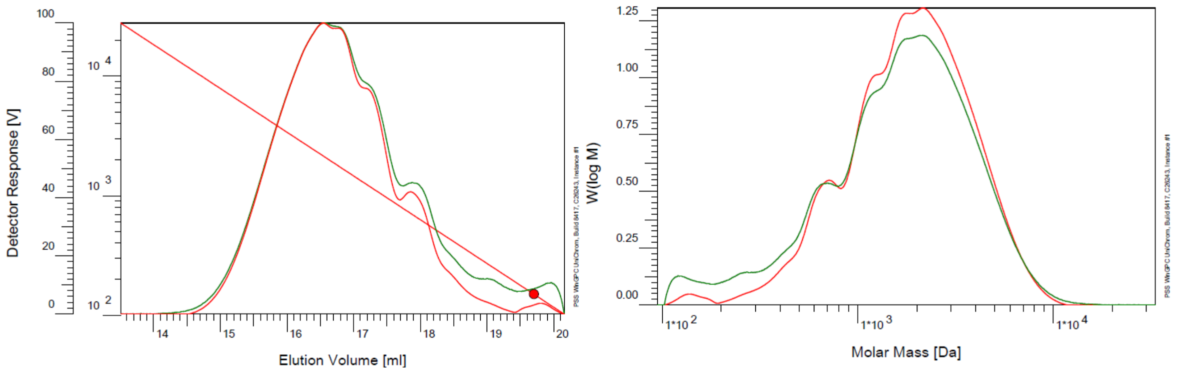

Figure S120. Crude GPC chromatograms (elution volume and molar mass) of the product mixture obtained from the PA / DCAGE ROCOP (*Table S4*, entry 1).

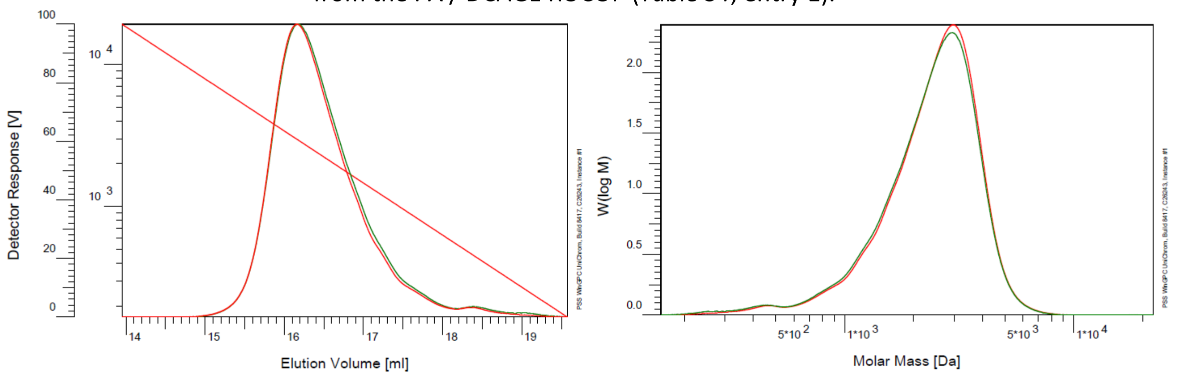

Figure S121. Crude GPC chromatograms (elution volume and molar mass) of the product mixture obtained from the PA / PGGE ROCOP (*Table S4*, entry 5).

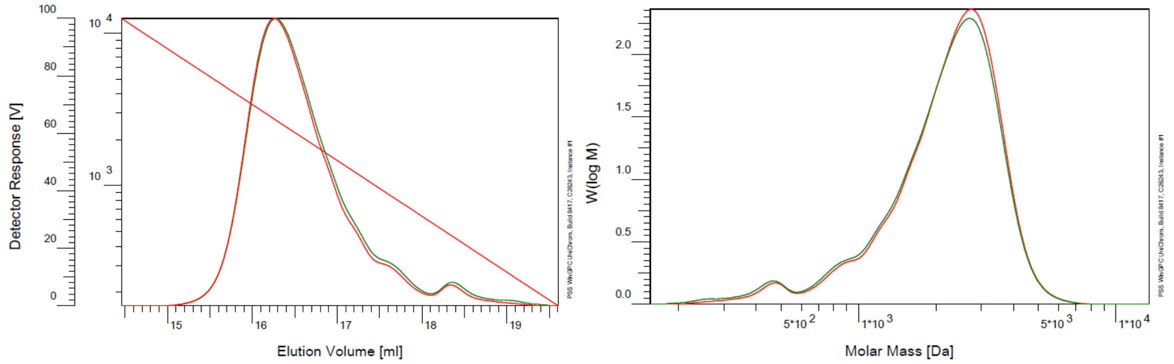

Figure S122. Crude GPC chromatograms (elution volume and molar mass) of the product mixture obtained from the PA / PGGE ROCOP using PPNCl organic catalyst (*Table S4*, entry 6).

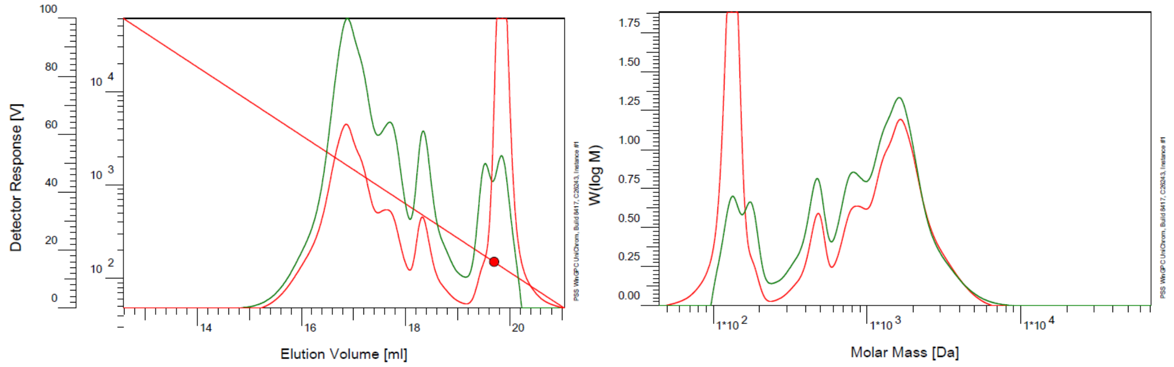

Figure S123. Crude GPC chromatograms (elution volume and molar mass) of the product mixture obtained from the PA / EGE ROCOP (*Table S4*, entry 7).

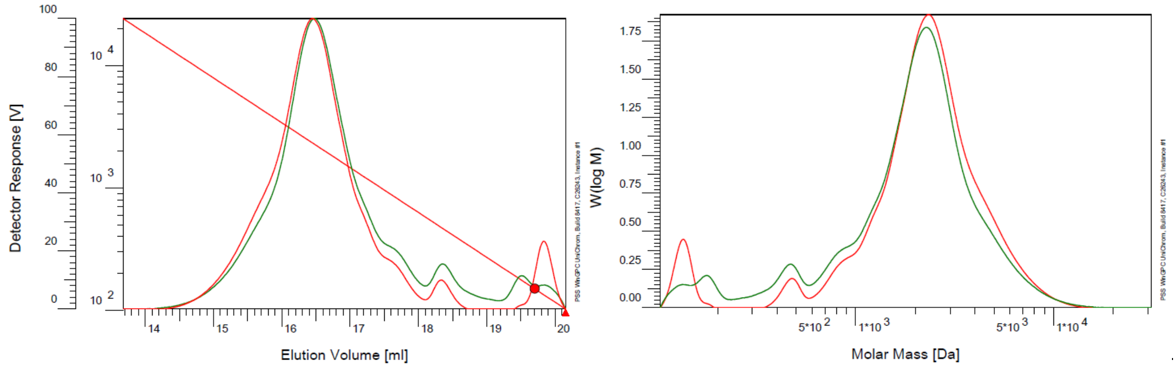

Figure S124. Crude GPC chromatograms (elution volume and molar mass) of the product mixture obtained from the PA / EGE ROCOP using PPNCl organic catalyst (*Table S4*, entry 8).

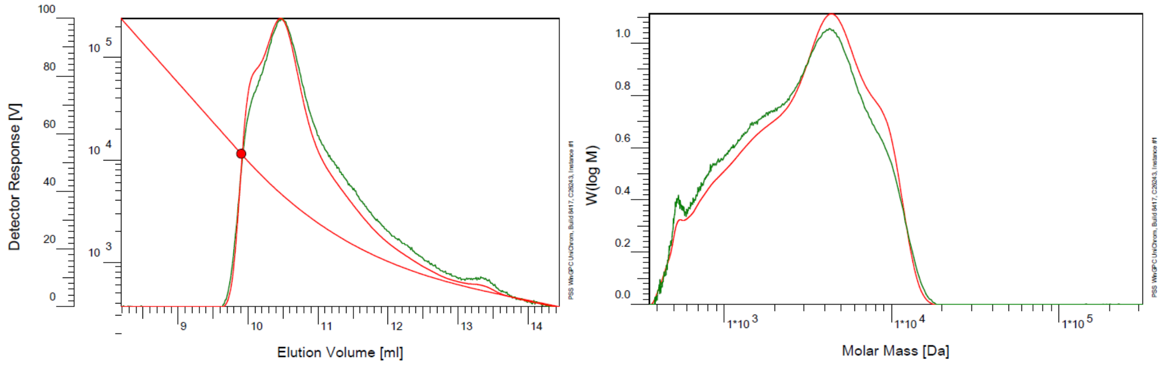

Figure S125. GPC chromatograms (elution volume and molar mass) of the isolated polymer obtained from the PA / LHOGE ROCOP (*Table S8*, entry 1).

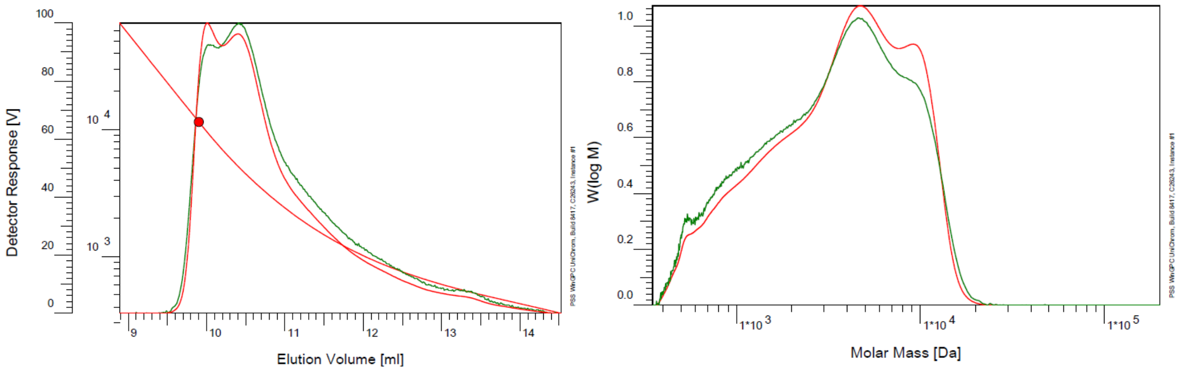

Figure S126. GPC chromatograms (elution volume and molar mass) of the isolated polymer obtained from the PA / LHOGE ROCOP using PPNCl organic catalyst (*Table S8*, entry 2).

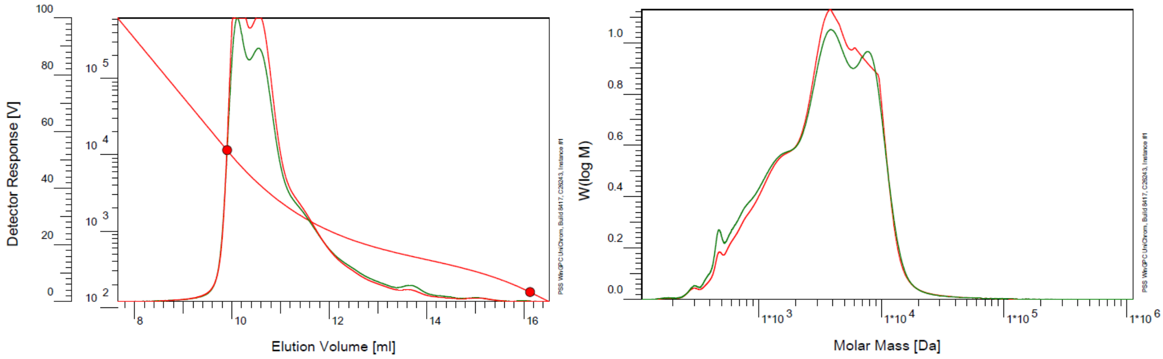

Figure S127. GPC chromatograms (elution volume and molar mass) of the isolated polymer obtained from the PA / LHOGE ROCOP using PPNCl organic catalyst (*Table S8*, entry 6).

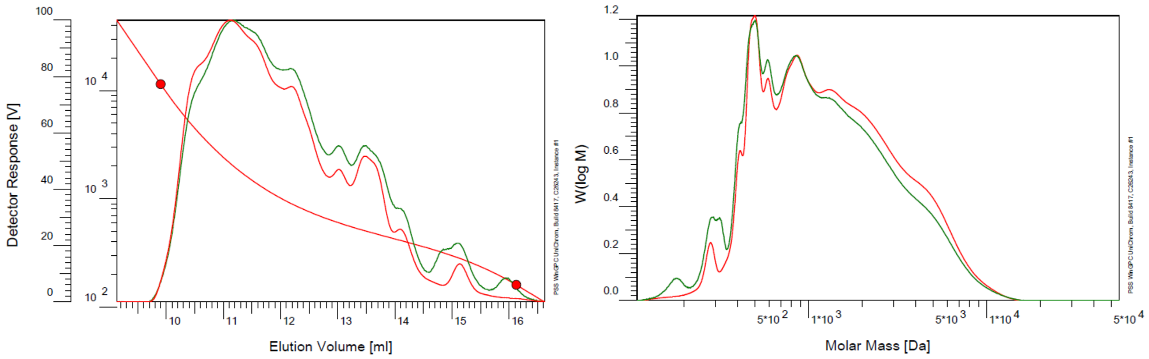

Figure S128. GPC chromatograms (elution volume and molar mass) of the isolated polymer obtained from the SA / DCAGE ROCOP using PPNCl organic catalyst (*Table S9*, entry 3).

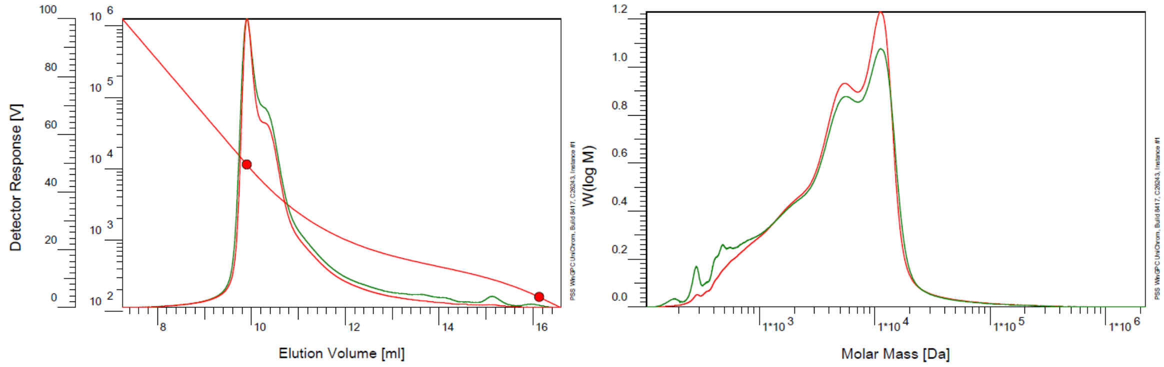

Figure S129. GPC chromatograms (elution volume and molar mass) of the isolated polymer obtained from the SA / LHOGE ROCOP (*Table S9*, entry 4).

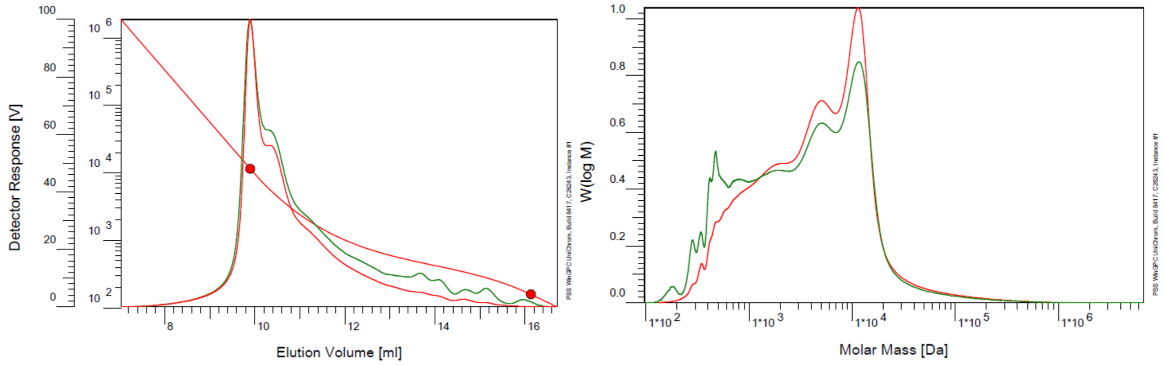

Figure S130. GPC chromatograms (elution volume and molar mass) of the isolated polymer obtained from the SA / LHOGE ROCOP using PPNCl organic catalyst (*Table S9*, entry 5).

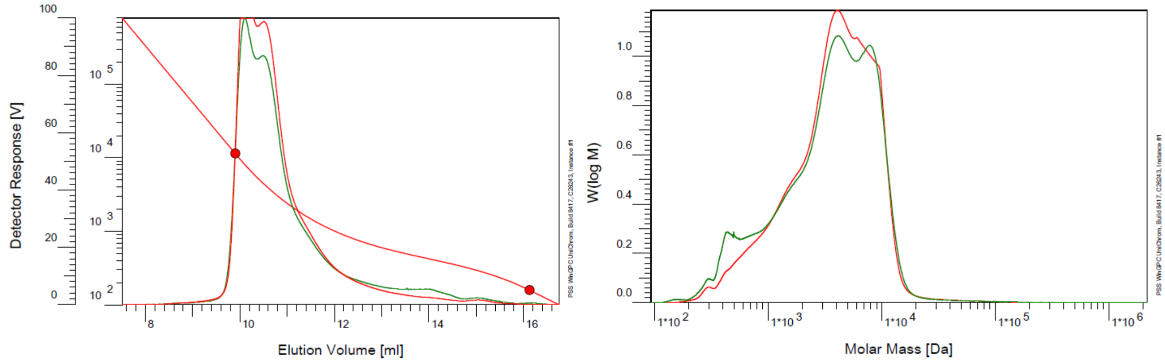

Figure S131. GPC chromatograms (elution volume and molar mass) of the isolated polymer obtained from the SA / LHOGE ROCOP using PPNCl organic catalyst (*Table S9*, entry 7).

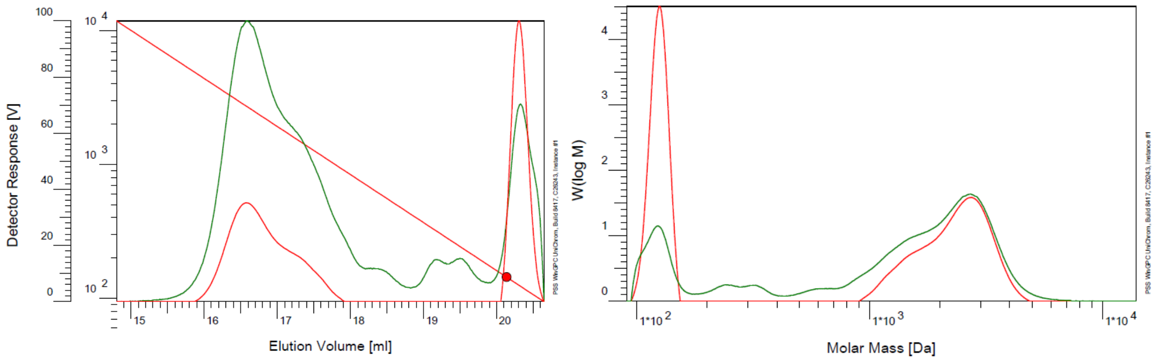

Figure S132. Crude GPC chromatograms (elution volume and molar mass) of the isolated crude product mixture obtained from the PA / synthesized PGE ROCOP using no PPNCl organic catalyst (*Table S5*, entry 2).

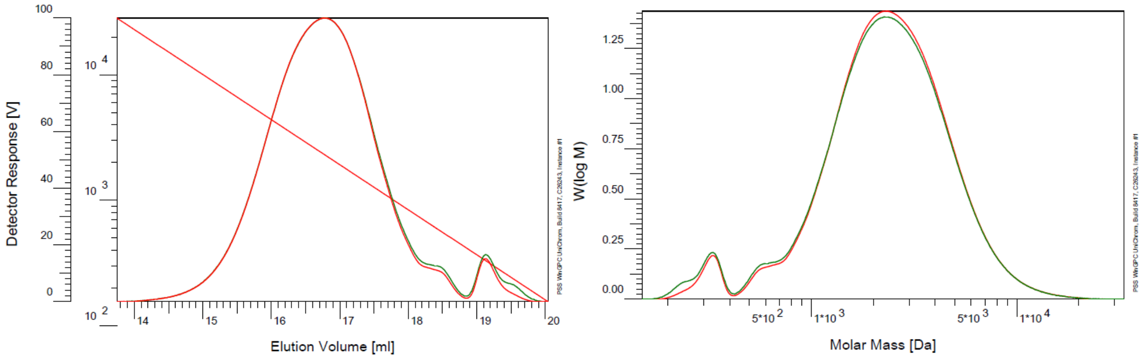

Figure S133. Crude GPC chromatograms (elution volume and molar mass) of the isolated crude product mixture obtained from the PA / synthesized PGE ROCOP using no PPNCl organic catalyst (*Table S5*, entry 4).

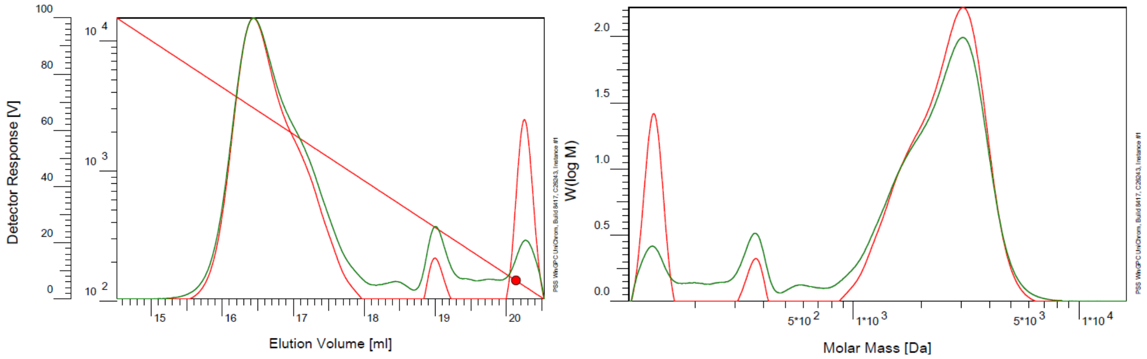

Figure S134. Crude GPC chromatograms (elution volume and molar mass) of the isolated crude product mixture obtained from spiking 25% [DCAGE] into the PA / PGE ROCOP using no PPNCl organic catalyst (*Table S6*).

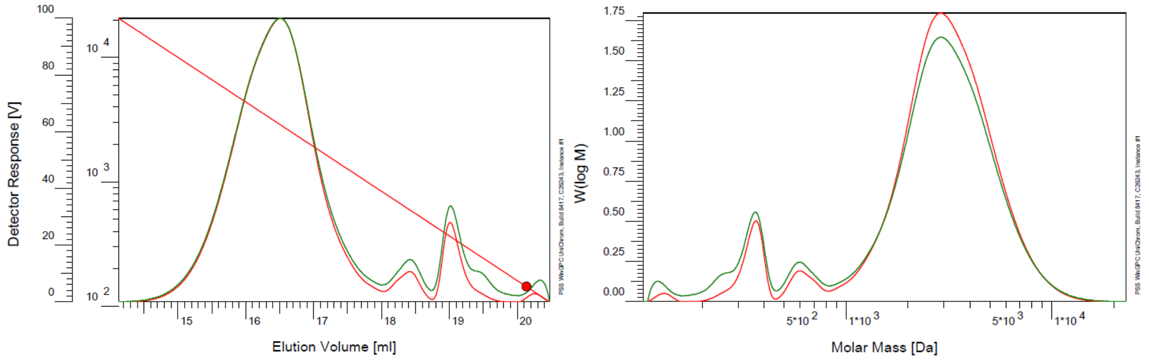

Figure S135. Crude GPC chromatograms (elution volume and molar mass) of the isolated crude product mixture obtained from spiking 10% [DCAGE] into the PA / PGE ROCOP using no PPNCl organic catalyst (*Table S6*).

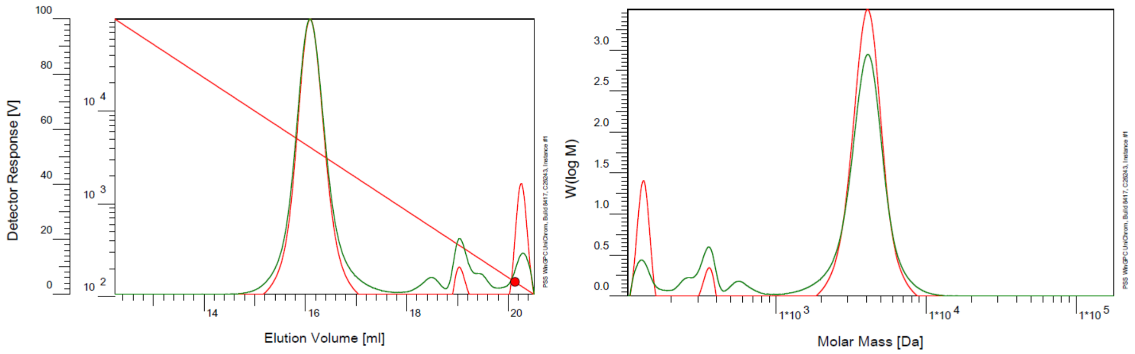

Figure S136. Crude GPC chromatograms (elution volume and molar mass) of the isolated crude product mixture obtained from spiking 1% [DCAGE] into the PA / PGE ROCOP using no PPNCl organic catalyst (*Table S6*).

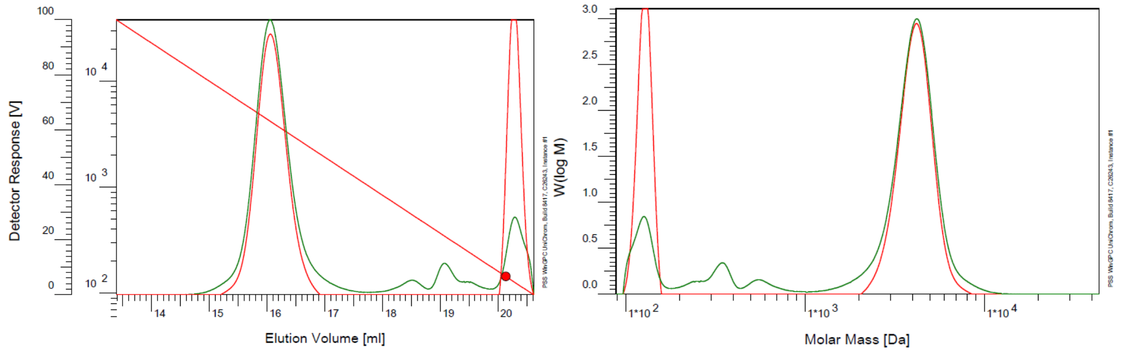

Figure S137. Crude GPC chromatograms (elution volume and molar mass) of the isolated crude product mixture obtained from spiking 0.1% [DCAGE] into the PA / PGE ROCOP using no PPNCl organic catalyst (*Table S6*).

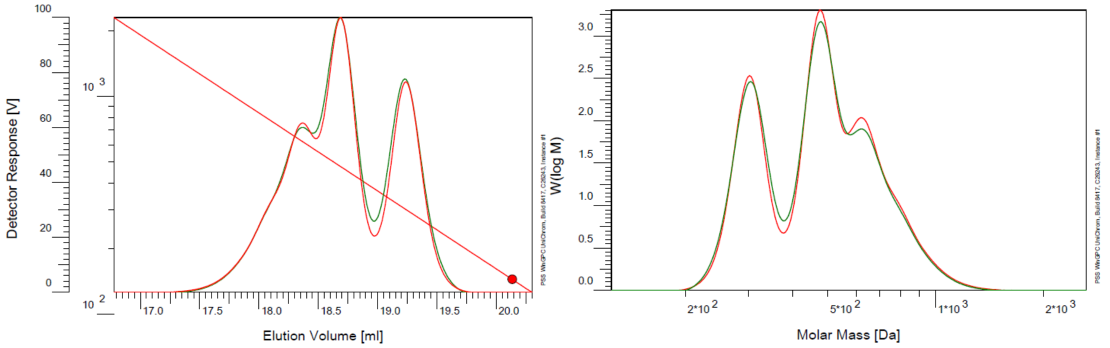

Figure S138. Crude GPC chromatograms (elution volume and molar mass) of the isolated crude product mixture obtained from spiking 3–phenyl–1–propanol into the PA / PGE ROCOP using no PPNCl organic catalyst (*Table S7*, entry 5).

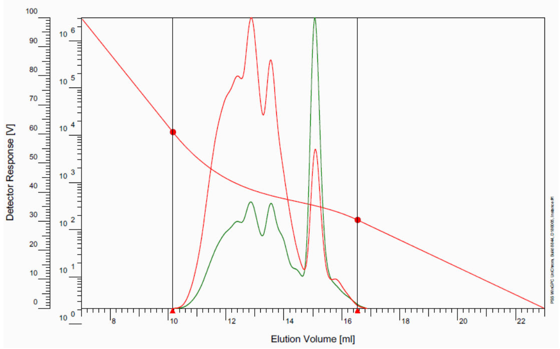

Figure S139. GPC chromatogram of the isolated LHO obtained from the hydrogenolysis of *Pinus radiata* (M_n = 479 g.mol^{-1}, M_w = 707 g.mol^{-1}, Đ = 1.48). See previously literature.[7,8,13]

S3.2. Polyurethane material and thermomechanical characterization

DMTAs and tensile profiles of the PU films (*Figures S140–S144*)

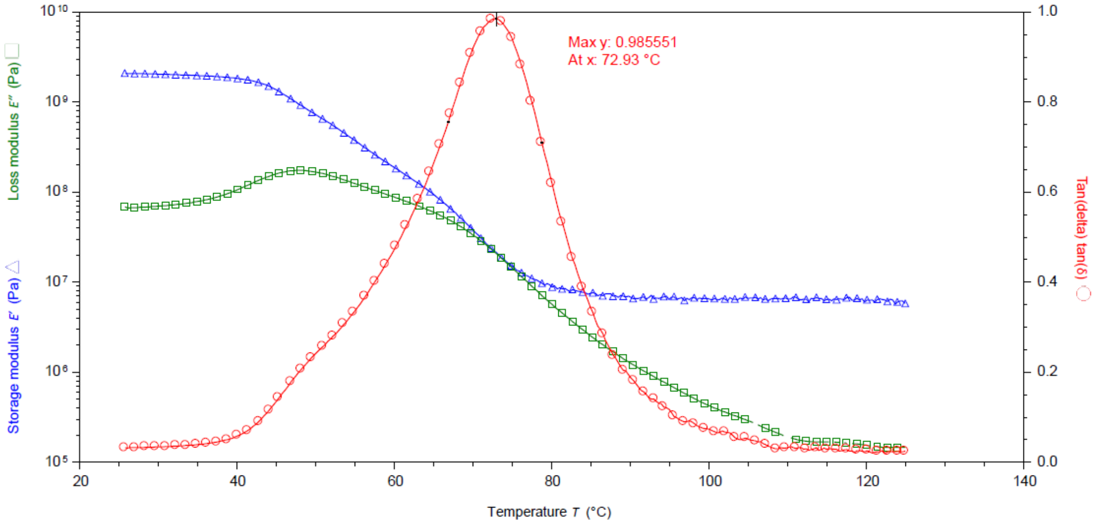

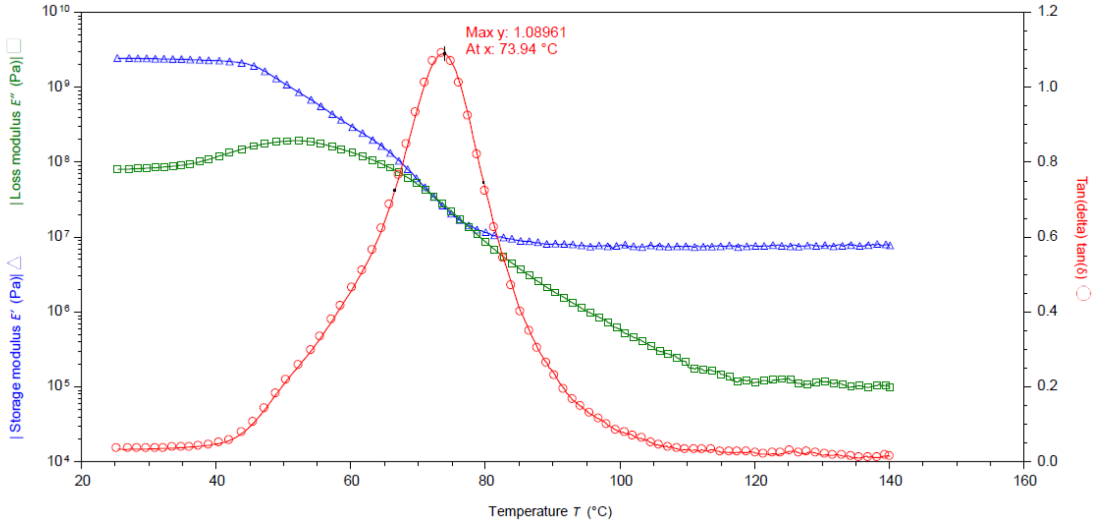

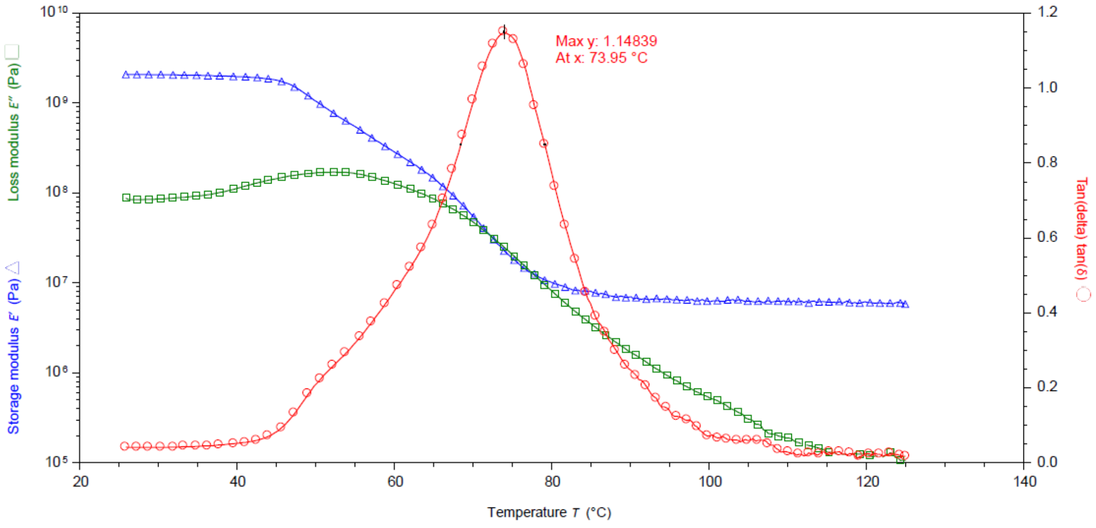

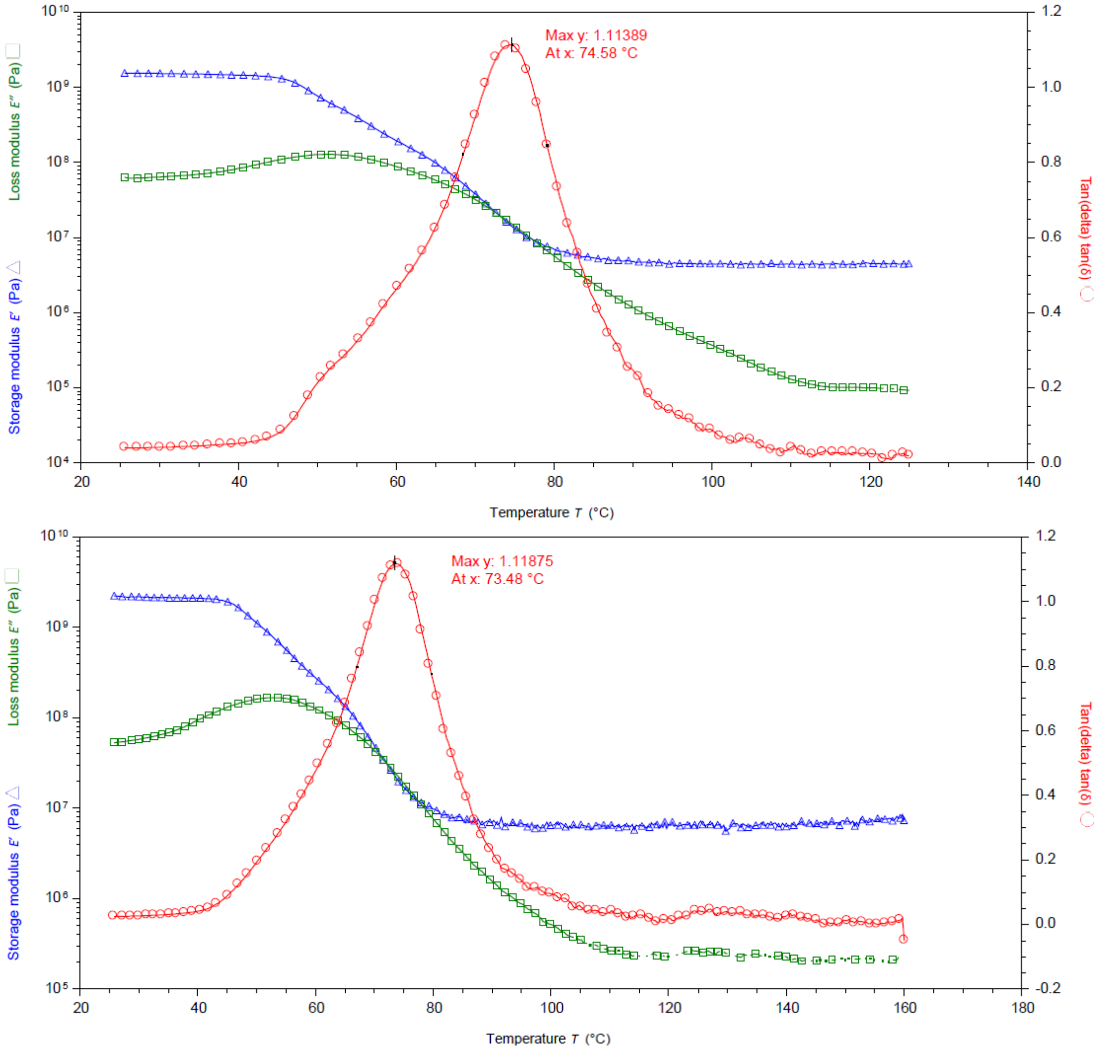

Figure S140. DMTA curves for the isolated PU film product obtained from using poly(PA–*co*–DCAGE) (*Table 2*, entry 4).

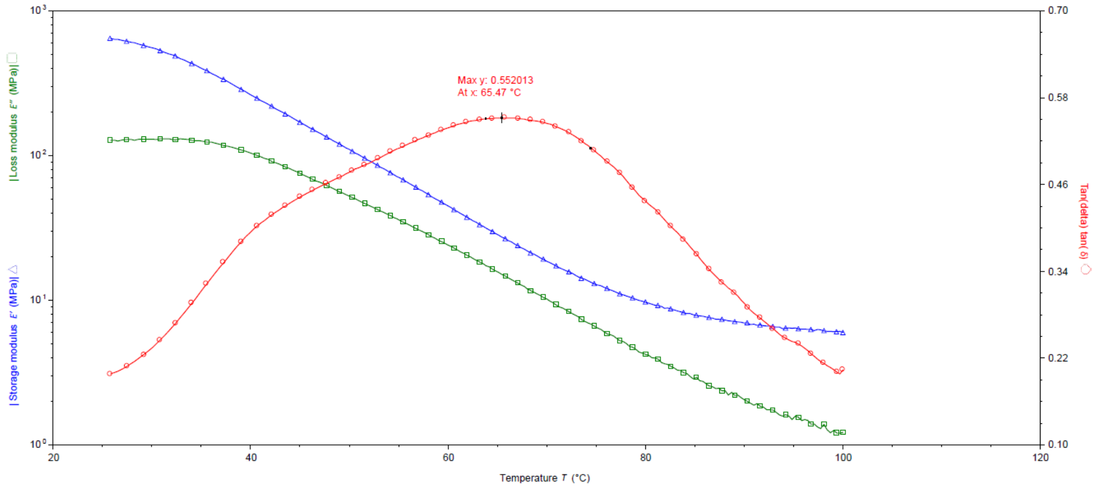

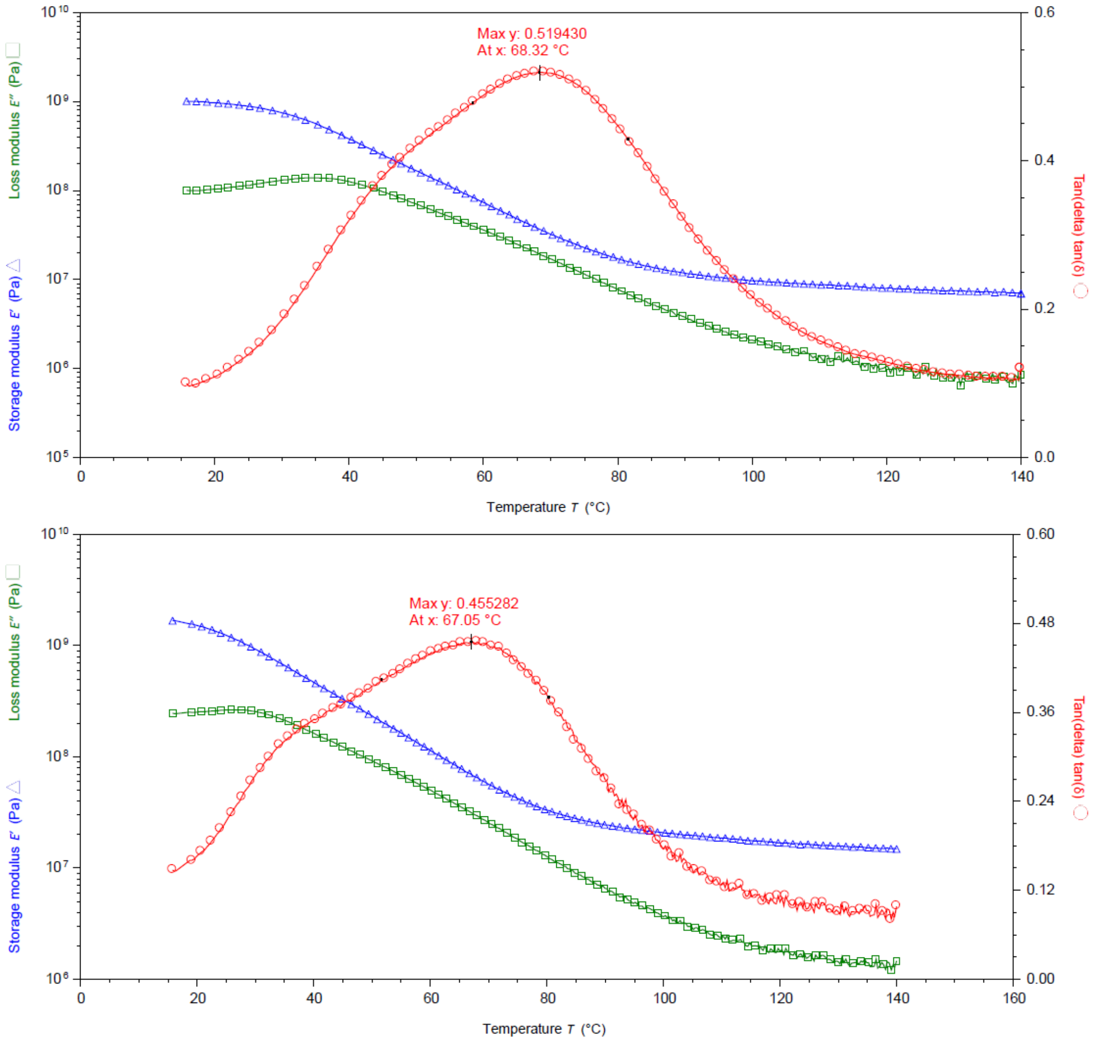

Figure S141. DMTA curves for the isolated PU film product obtained from using poly(PA–*co*–LHOGE) (*Table S8*, entry 6).

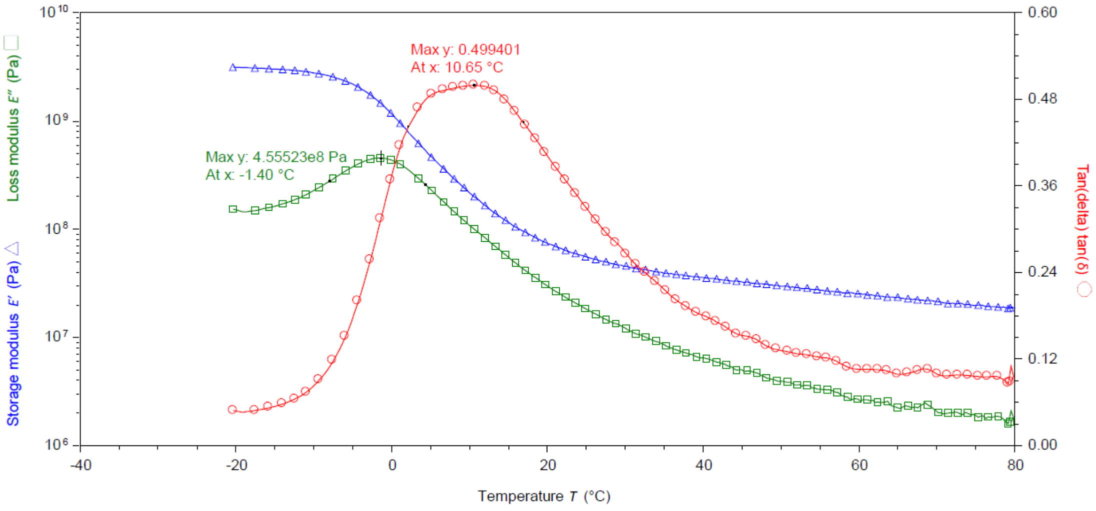

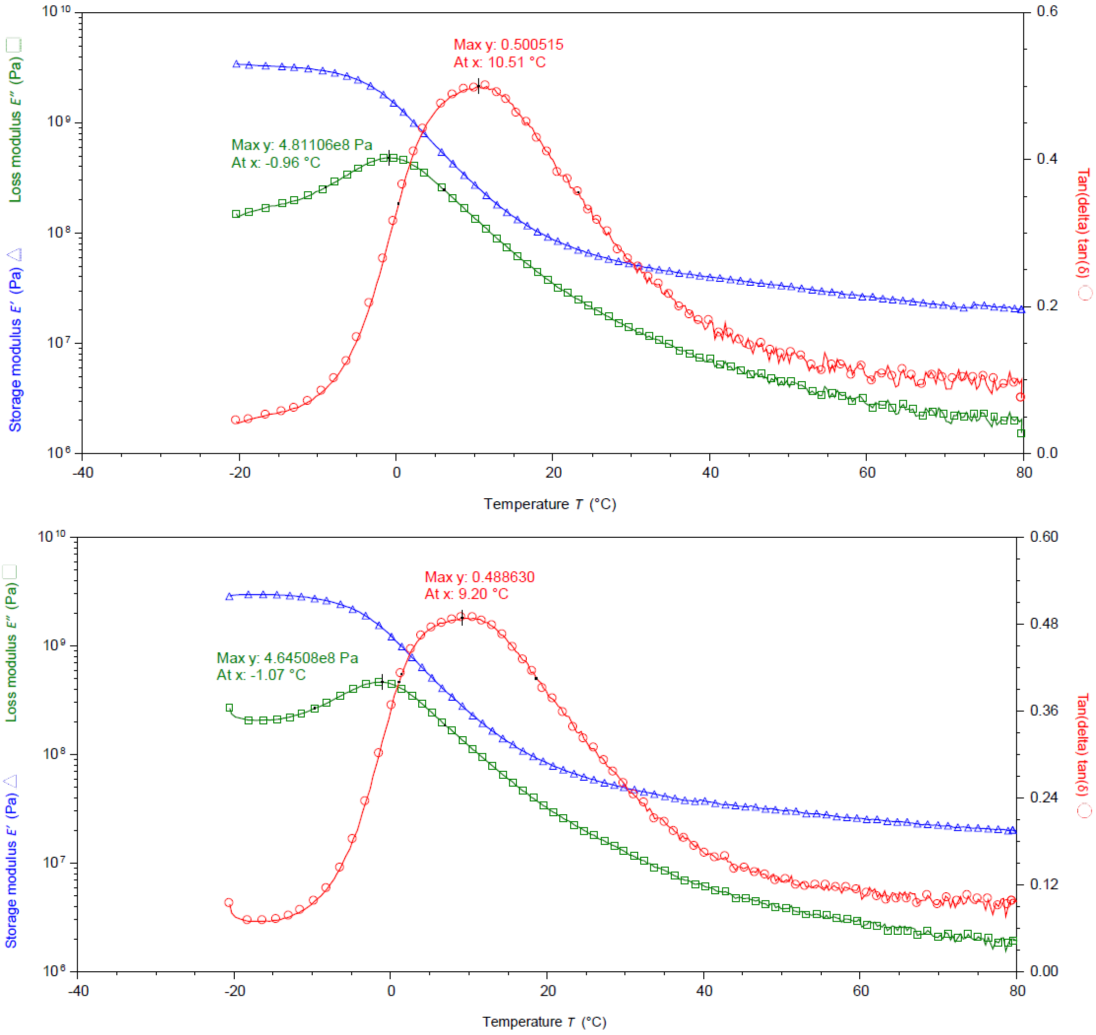

Figure S142. DMTA curves for the isolated PU film product obtained from using poly(SA–*co*–DCAGE) (*Table S9*, entry 3).

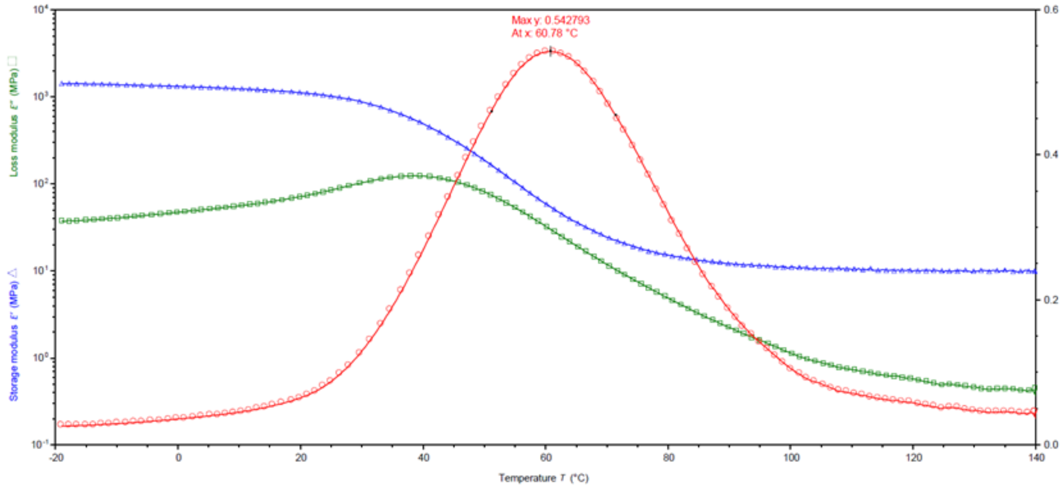

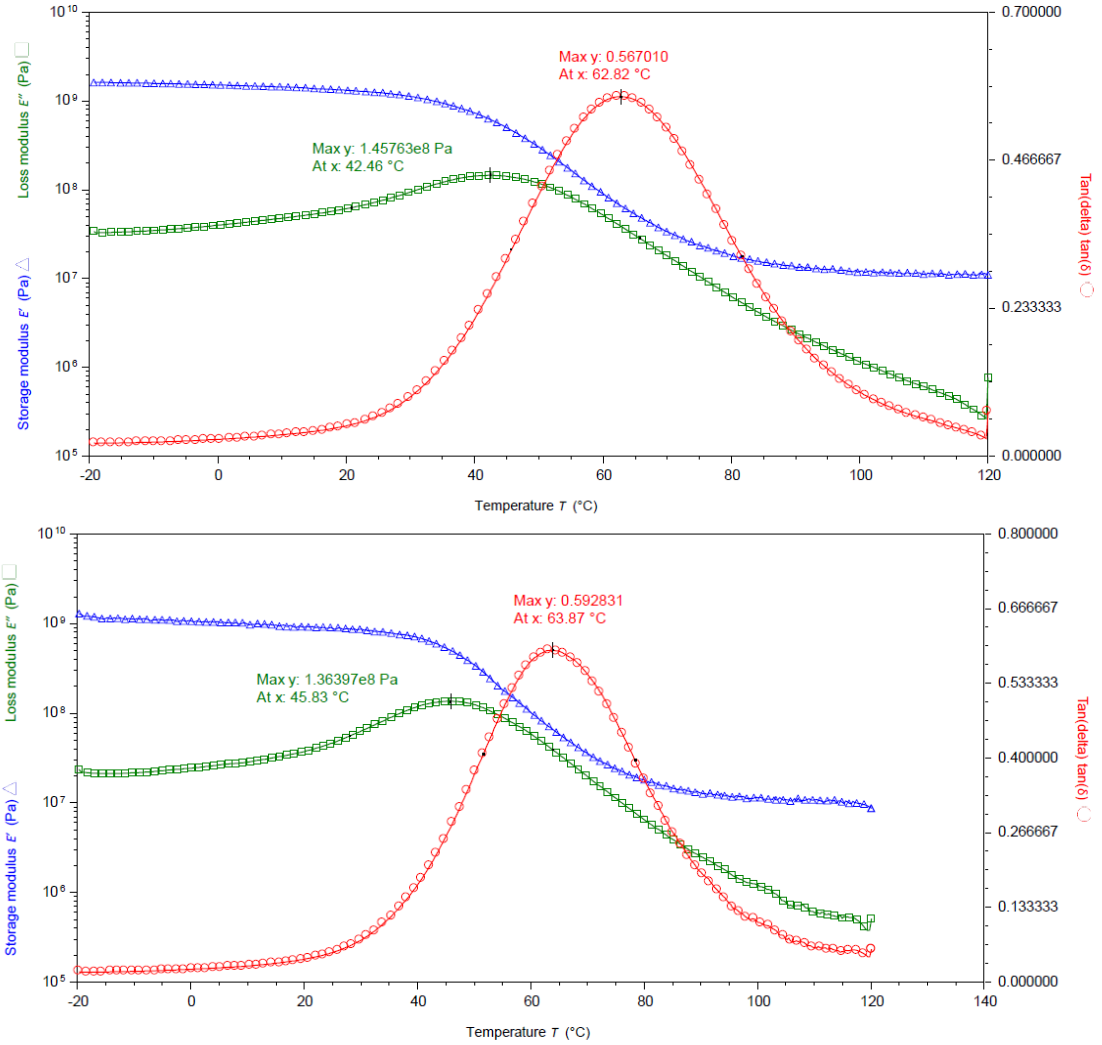

Figure S143. DMTA curves for the isolated PU film product obtained from using poly(SA–*co*–LHOGE) (*Table S9*, entry 7).

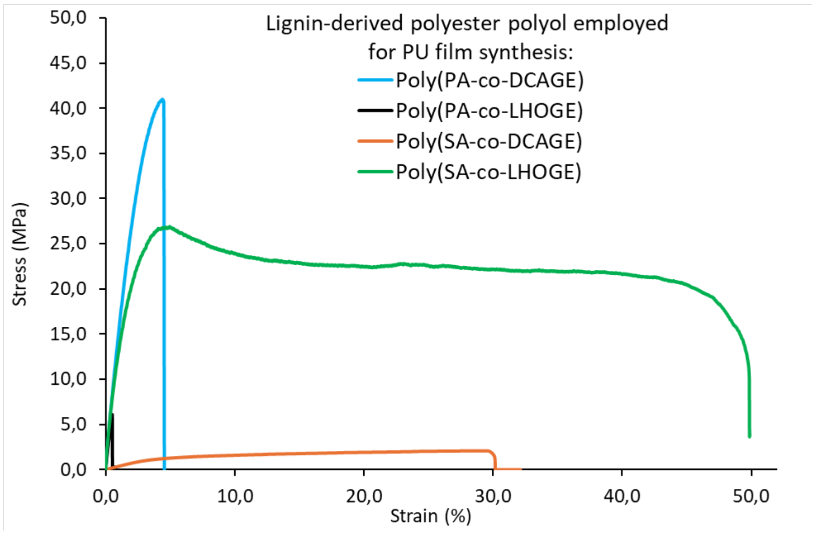

Figure S144. Stress–strain curves of representative samples of the synthesized, tested PU films to show the varying tensile profiles and properties from the different lignin-derived polyesters as polyols related to the differences in the monomer substrate chemical structures utilized.

S3.3. Lignin model compound synthesis and characterization

All synthesized model compounds, including DCA, PGGE, EGE, and DCAGE, have been fully characterized and reported by us previously in the literature.[14]

S4. References

(1) Liu, B.; Chen, J.; Liu, N.; Ding, H.; Wu, X.; Dai, B.; Kim, I. Bio-Based Polyesters Synthesized by Ring-Opening Copolymerizations of Eugenyl Glycidyl Ether and Cyclic Anhydrides Using a Binuclear [OSSO]CrCl Complex. *Green Chemistry* **2020**, *22* (17), 5742–5750. https://doi.org/10.1039/d0gc00469c.

(2) Haslewood, M. N. D.; Farmer, T. J.; North, M. Synthesis and Chemoselective Crosslinking of Functionalized Polyesters from Bio-Based Epoxides and Cyclic Anhydrides. *Journal of Polymer Science* **2022**. https://doi.org/10.1002/pol.20220552.

(3) Saini, P. K.; Romain, C.; Zhu, Y.; Williams, C. K. Di-Magnesium and Zinc Catalysts for the Copolymerization of Phthalic Anhydride and Cyclohexene Oxide. *Polym Chem* **2014**, *5*, 6068–6075.

(4) Driscoll, O. J.; Stewart, J. A.; McKeown, P.; Jones, M. D. Ring-Opening Copolymerization Using Simple Fe(III) Complexes and Metal- and Halide-Free Organic Catalysts. *Macromolecules* **2021**, *54* (18), 8443–8452. https://doi.org/10.1021/acs.macromol.1c01211.

(5) Bester, K.; Bukowska, A.; Myśliwiec, B.; Hus, K.; Tomczyk, D.; Urbaniak, P.; Bukowski, W. Alternating Ring-Opening Copolymerization of Phthalic Anhydride with Epoxides Catalysed by Salophen Chromium(III) Complexes. An Effect of Substituents in Salophen Ligands. *Polym Chem* **2018**, *9* (16), 2147–2156.

(6) Ryzhakov, D.; Printz, G.; Jacques, B.; Messaoudi, S.; Dumas, F.; Dagorne, S.; Le Bideau, F. Organo-Catalyzed/Initiated Ring Opening Co-Polymerization of Cyclic Anhydrides and Epoxides: An Emerging Story. *Polym Chem* **2021**, *12*, 2932–2946.

(7) Quinsaat, J. E. Q.; Feghali, E.; van de Pas, D. J.; Vendamme, R.; Torr, K. M. Preparation of Biobased Nonisocyanate Polyurethane/Epoxy Thermoset Materials Using Depolymerized Native Lignin. *Biomacromolecules* **2022**, *23* (11), 4562–4573. https://doi.org/10.1021/acs.biomac.2c00706.

(8) Feghali, E.; van de Pas, D. J.; Torr, K. M. Toward Bio-Based Epoxy Thermoset Polymers from Depolymerized Native Lignins Produced at the Pilot Scale. *Biomacromolecules* **2020**, *21* (4), 1548–1559. https://doi.org/10.1021/acs.biomac.0c00108.

(9) van de Pas, D. J.; Nanayakkara, B.; Suckling, I. D.; Torr, K. M. Comparison of Hydrogenolysis with Thioacidolysis for Lignin Structural Analysis. *Holzforschung* **2014**, *68* (2), 151–155. https://doi.org/10.1515/hf-2013-0075.

(10) Gracia-Vitoria, J.; Rubens, M.; Feghali, E.; Adriaensens, P.; Vanbroekhoven, K.; Vendamme, R. Low-Field Benchtop versus High-Field NMR for Routine 31P Analysis of Lignin, a Comparative Study. *Ind Crops Prod* **2022**, *176*, 114405. https://doi.org/10.1016/j.indcrop.2021.114405.

(11) Granata, A.; Argyropoulos, D. S. 2-Chloro-4,4,5,5-Tetramethyl-1,3,2-Dioxaphospholane, a Reagent for the Accurate Determination of the Uncondensed and Condensed Phenolic

Moieties in Lignins. *J Agric Food Chem* **1995**, *43* (6), 1538–1544. https://doi.org/10.1021/jf00054a023.

(12) Meng, X.; Crestini, C.; Ben, H.; Hao, N.; Pu, Y.; Ragauskas, A. J.; Argyropoulos, D. S. Determination of Hydroxyl Groups in Biorefinery Resources via Quantitative 31P NMR Spectroscopy. *Nat Protoc* **2019**, *14* (9), 2627–2647. https://doi.org/10.1038/s41596-019-0191-1.

(13) Quinsaat, J. E. Q.; Feghali, E.; van de Pas, D. J.; Vendamme, R.; Torr, K. M. Preparation of Mechanically Robust Bio-Based Polyurethane Foams Using Depolymerized Native Lignin. *ACS Appl Polym Mater* **2021**, *3* (11), 5845–5856. https://doi.org/10.1021/acsapm.1c01081.

(14) Driscoll, O. J.; Van Hecke, K.; Vande Velde, C. M. L.; Blockhuys, F.; Rubens, M.; Kuwaba, T.; van de Pas, D. J.; Eevers, W.; Vendamme, R.; Feghali, E. Solid-State Structures and Properties of Lignin Hydrogenolysis Oil Compounds: Shedding a Unique Light on Lignin Valorization. *Int J Mol Sci* **2024**, *25* (19), 10810. https://doi.org/10.3390/ijms251910810.
